# Deprotonative Single-Electron Oxidation as a General and Controllably Selective Platform for Benzylic C–H Functionalization

**DOI:** 10.1021/jacs.6c04370

**Published:** 2026-05-28

**Authors:** Nicholas J. Coradi, Jeffrey S. Bandar

**Affiliations:** Department of Chemistry, 3447 Colorado State University, Fort Collins, Colorado 80523, United States

## Abstract

We propose a new strategy for benzyl radical generation through a consecutive deprotonation and single-electron oxidation mechanism. The use of an HMDS base generates a low concentration of a benzyl carbanionic intermediate that is oxidized and subsequently captured using two equivalents of the persistent aminoxyl radical reagent TEMPO. Compared to oxidation reactions of stoichiometrically prepared carbanionic species, endergonic deprotonation ensures selective radical coupling over dimerization and enables a major improvement in functional group tolerance and site-selectivity control. This approach is guided by C–H acidity and carbanion oxidation trends and therefore exhibits distinct scope and selectivity over hydrogen atom abstraction and direct single-electron oxidation-based methods that are instead guided by alkylarene C–H bond strengths or oxidation potentials, respectively. As such, this protocol is applicable to alkylarenes with high oxidation potentials and tolerates the presence of weak C–H bonds and oxidatively sensitive functional groups. These features enable TEMPO installation on densely functionalized compounds, including pharmaceutical and *N*-heteroaryl substrates. This approach also introduces the prospect of exploiting the inverse correlation between C–H acidity and the oxidation potential of its deprotonated intermediate such that either property could interchangeably dictate site-selectivity for arenes with multiple benzylic positions. Thus, simple adjustments to the base strength are proposed to initiate a Curtin–Hammett-regulated oxidation pathway that enables a switch in selectivity from the most to least acidic benzylic position. We anticipate that these collective features will enhance benzylic C–H diversification efforts given the downstream synthetic versatility of benzyl–OTMP units.

## Graphical abstract

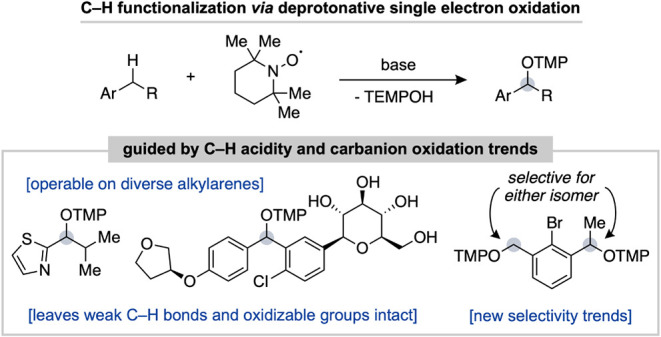

## Introduction

The generation of carbon-centered radicals from C–H bonds serves as a key step in many valuable synthetic transformations.^1^ This process transforms abundant and typically inert C–H bonds into reactive intermediates that engage in diverse reactions such as oxidation, coupling, and cyclization.^2^ Accordingly, these reactions are utilized in many contexts, including the valorization of simple hydrocarbons, the direct functionalization of commercial building blocks without the need for preinstalled functional groups, and late-stage diversification of complex molecules.^3^ The functionalization of benzylic C–H bonds via radical generation is particularly well-developed and commonly used given the ubiquity of benzylic substructures in medicinal, agrochemical, and other commercial contexts (Figure 1a).^2–4^ Benzylic C–H bonds are well-positioned to be activated toward radical formation via three main established mechanistic strategies. First, hydrogen atom abstraction (HAA)-based protocols are the most widely developed, as typified by the classic Wohl–Ziegler bromination method, a process primarily guided by C–H bond strengths, as well as polar effects and the properties of the abstraction reagent.^1,5–7^ Second, single-electron oxidation of an alkylarene acidifies the benzylic C–H bond and readily leads to radical formation, a process regulated mainly by substrate oxidation potential.^8^ Third, concerted proton-coupled electron transfer (PCET) has been introduced recently via the tandem use of strong oxidants and weak bases, with initial reports showing selectivity for the weakest benzylic C–H bond.^9^ In this article, we outline a distinct benzylic radical generation strategy that is proposed to operate via consecutive C–H deprotonation and single-electron oxidation, which consequently offers a new set of scope and selectivity guidelines for C–H oxidation.

**Figure 1:**
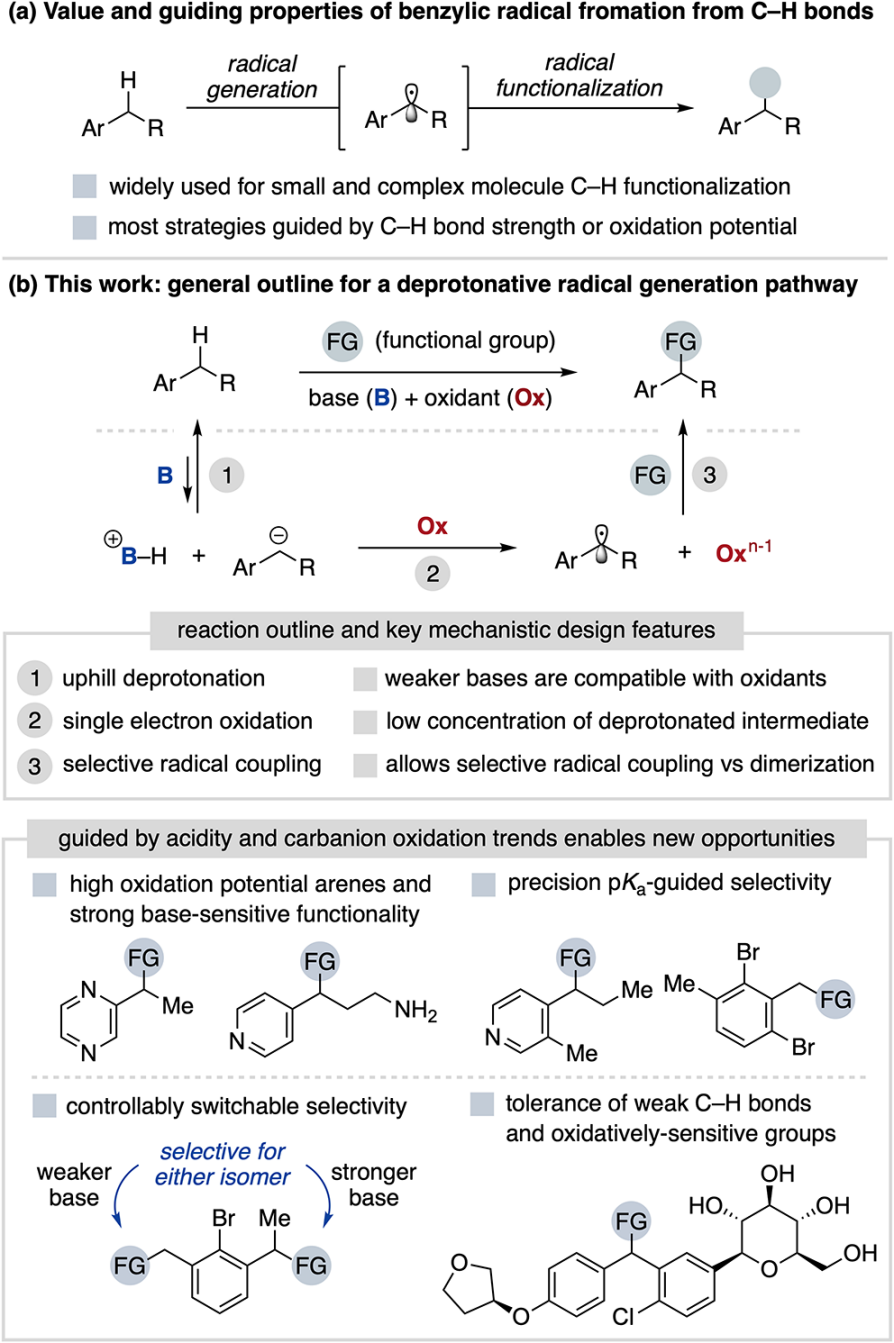
An outline and utility of a deprotonation/single-electron oxidation approach to benzylic C–H functionalization.

We proposed that an initial energetically uphill deprotonation step could be used to overcome the traditional challenges associated with single-electron oxidation of carbanionic species (Figure 1b).^10^ By generating only a low concentration of a deprotonated intermediate, we hypothesized that, upon oxidation, the resulting low radical intermediate concentration would enable selective coupling over dimerization. This is distinct from most prior studies on single-electron oxidation of stoichiometrically pregenerated carbanionic (or organometallic) species that lead to dimerization, a process commonly used for the homocoupling of enolates, arenes, and benzylic compounds.^11,12^ Additionally, relatively mild bases are more compatible with single-electron oxidants than the stronger bases traditionally used for stoichiometric metalation of weakly acidic C–H bonds (e.g., alkyllithium bases).^13,14^ Finally, we anticipated that these conditions could allow for the incorporation of diverse radical coupling partners, with our initial goal being to identify a process that could transform a C–H bond into a diversifiable functional handle.

In addition to being driven by simple and practical reagents, successful implementation of this activation strategy would introduce a range of new synthetic capabilities over established radical generation methods (Figure 1b, bottom). For example, a deprotonative oxidation process could operate on electron-deficient alkyl­(hetero)­arenes that are difficult to oxidize in their neutral forms via chemical or photo/electrochemical approaches.^8,15^ An anionic oxidation approach could also tolerate functional groups that are sensitive to classical oxidants or HAA reagents (e.g., weak C–H bonds, π-nucleophilic alkenes/arenes, and S/N-containing groups), as well as those that coordinate to metals.^5,16^ For compounds with multiple potential sites for C–H functionalization, the site-selectivity could be guided by relative kinetic C–H acidity. This is not only distinct from HAA-based method, but also from traditional exergonic metalation protocols that use very strong bases (e.g., superbase mixtures of TMPH/KO-*t*-Bu/*n*-BuLi) and are typically unselective for similar Csp^3^–H positions.^5,14^ Furthermore, we hypothesized that by separating the deprotonation and oxidation events, either step could be rendered selectivity-determining. As demonstrated herein, simple condition variation allows for highly predictable and switchable selectivity between 1° and 2° benzylic C–H bonds.

This report describes our initial development of this approach using aminoxyl reagents as both the oxidant and the coupling partner (Figure 2a).^17,18^ This reaction design was inspired by the fact that TEMPO is a relatively weak oxidant that is known to promote single electron oxidation of organometallic reagents.^19,20^ For example, alkyllithium reagents (e.g., *n*-BuLi) undergo TEMPO-promoted oxidation to generate mixtures of alkyl-TEMPO, alkyl-dimerized, alkyl-protonated, and other alkyl-oxygenated products.^21^ We therefore proposed that the engagement of an aminoxyl reagent in a transient deprotonation strategy could overcome the challenges of deprotonative single-electron oxidation and unlock its synthetic advantages.^22^ Furthermore, this method translates these advantages in the context of transforming C–H bonds into an aminoxyl unit, which serves as a useful functional handle through reductive and oxidative activation protocols.^17,23^ Such transformations could thereby accomplish formal C–H conversion to diverse substructures, including oxygenated, C–heteroatom, and C–C coupled derivatives (Figure 2b). This deprotonative activation process thus offers novel opportunities compared to the recent innovative use of HMDS bases with oxoammonium salts by the Lin Group for frustrated radical pair chemistry, which installs −OTMP groups via an HAA-based mechanism (Figure 2c).^24^ During the writing of this manuscript, we note that a related KO-*t*-Bu-catalyzed TEMPO C–H coupling protocol was reported by Kondoh and Terada that is suitable primarily for diarylmethanes and allylbenzenes.^25^

**Figure 2:**
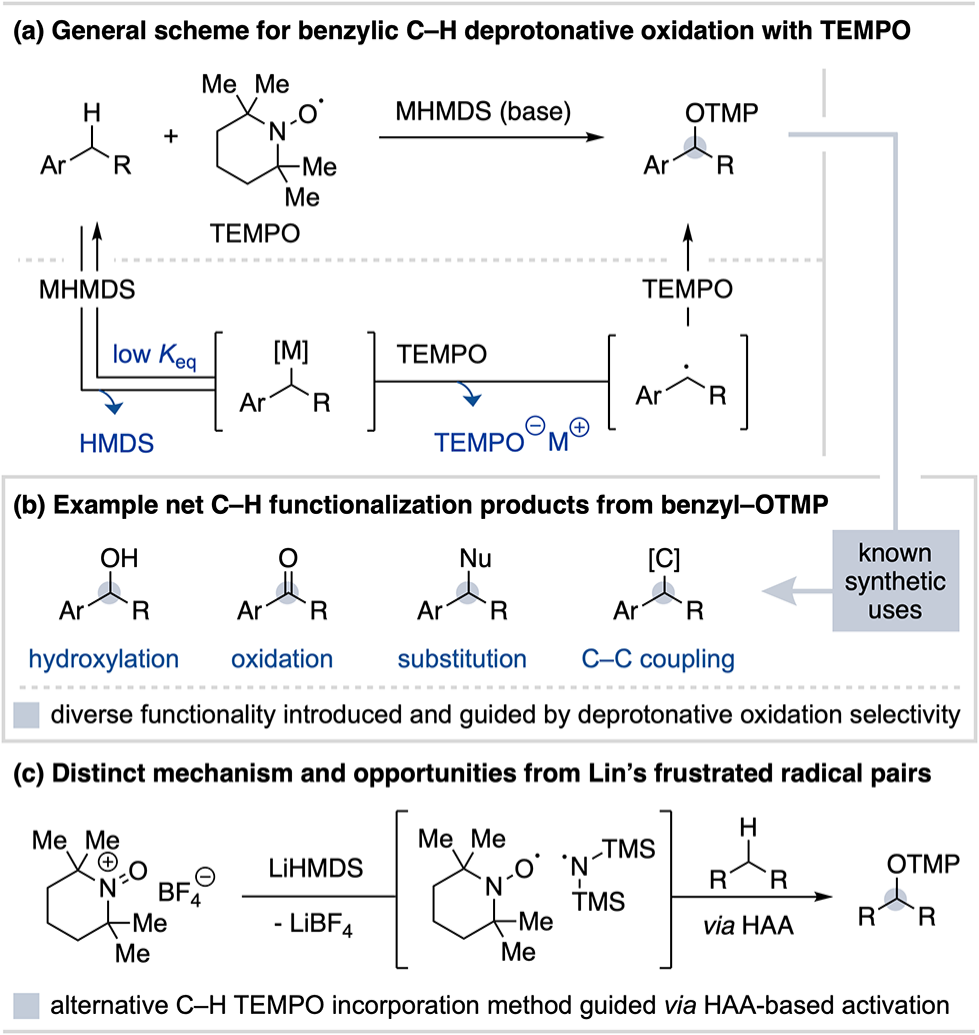
Reaction design and utility for a new benzylic C–H deprotonative oxidation process.

## Results and Discussion

### Reaction Optimization

It was initially unknown what type of alkylarene could engage in the proposed mechanism since a successful reaction requires both sufficient deprotonation and oxidation, two reactivity trends that are typically inversely correlated for a given C–H bond.^22,26^ For this reason, we selected three alkylarene substrates that span a wide p*K*_a_ range (**1**-**3**, approximately 30 to 44 in DMSO) to initially investigate.^27^ We further anticipated that if conditions for these substrates could be identified, it would suggest that this method is suitable for a broad scope of alkylarenes. Key parameters that were identified are shown in Figure 3, while more condition variation information is provided in the Supporting Information.

**Figure 3:**
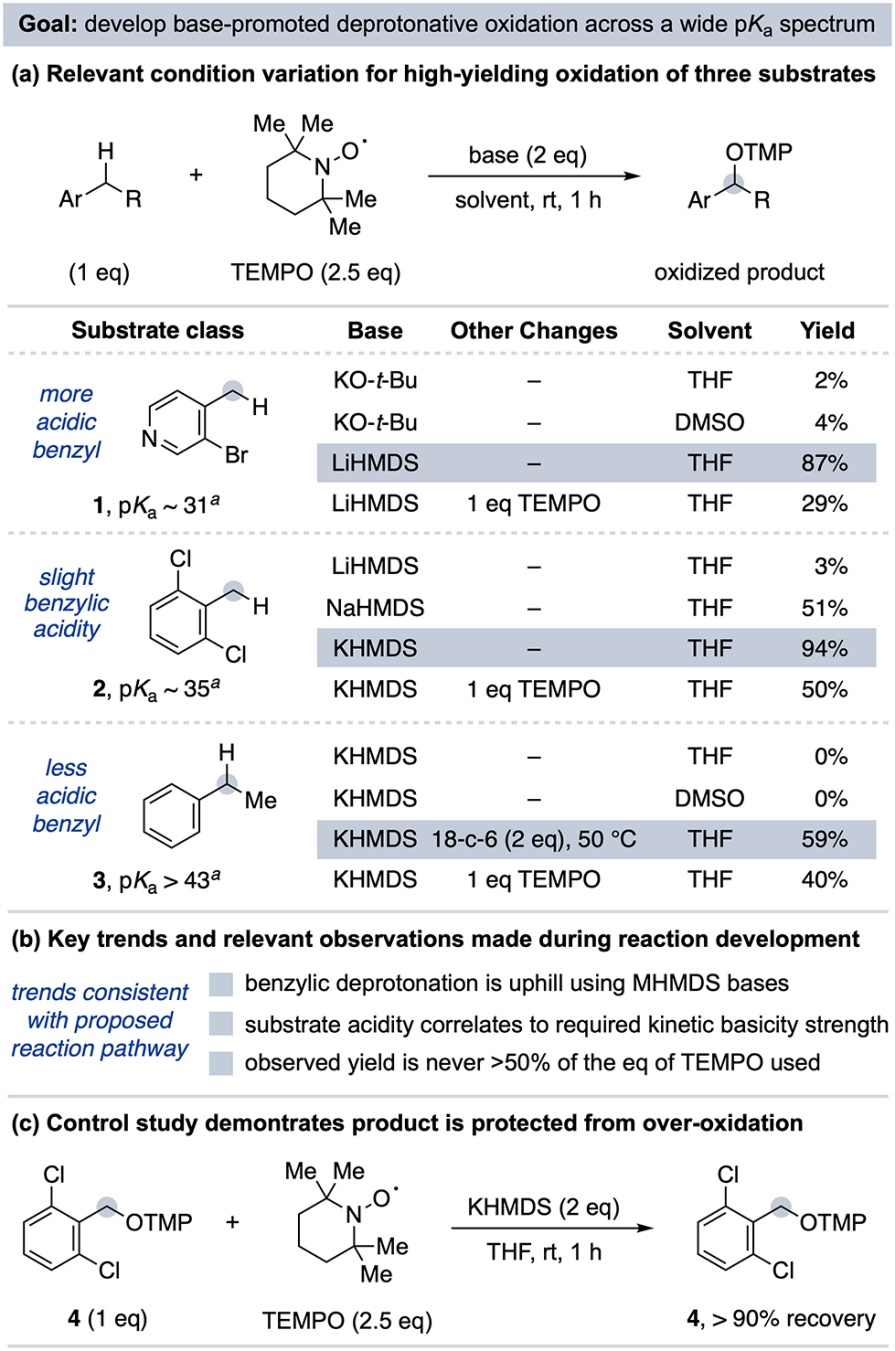
Condition optimization for base-promoted benzylic C–H oxidation across a wide p*K*_a_ spectrum. Yields obtained using ^1^H NMR spectroscopy. *^a^* p*K*_a_ values in DMSO estimated from ref 27.

The relatively acidic 3-bromo-4-methylpyridine (**1**) was first studied using KO-*t*-Bu as a base in various solvents, with minimal yield of the desired product observed. We initially suspected that the relatively stabilized carbanionic intermediate may not be suitable for reaction with TEMPO. However, when more kinetically active hexamethyldisilazide (HMDS) bases were examined in THF, including LiHMDS, a substantial yield increase to 87% was observed.^28^ Similarly, for the moderately acidic 2,6-dichlorotoluene (**2**), we found that oxidation yields continuously increase as the HMDS base countercation is changed from Li, Na, to K (up to 94% for KHMDS). For ethylbenzene (**3**), a substantially less acidic substrate, we found that the use of KHMDS with 18-crown-6 additive and a temperature increase to 50 °C is needed to achieve a 59% yield. A control experiment shows that the monooxidized alkylarene products are protected from overoxidation under these reaction conditions (Figure 3c), likely due to steric hindrance from the large −OTMP group.^29^

Several observations and trends from the reaction optimization studies are consistent with TEMPO serving as an oxidant for a transiently generated benzyl carbanionic intermediate (Figure 3b). First, although HMDS bases (H–N­(TMS)_2_ p*K*_a_ ∼ 26 in DMSO)^30^ are too weak to stoichiometrically deprotonate most benzylic C–H bonds, the correlation of higher kinetic basicity requirements for less acidic substrates (i.e., use of potassium countercation or ligating agents) suggests deprotonation is a key step for substrate activation.^31,32^ Accordingly, deuterium exchange control studies show that the basic conditions developed in Figure 3a readily deprotonate substrates **1**–**3** but with a low equilibrium constant.^33^ It is also notable that THF is used as a solvent and does not react at its weak α-C–H bond.^34^ This fact, in addition to the site-selectivity studies described below, is inconsistent with an operable HAA-based activation mechanism.^35^ Finally, in no case was a yield greater than half the amount of TEMPO used observed, implying a 2:1 reaction stoichiometry for TEMPO:alkylarene.^36^ Experimental and computational studies are ongoing to elucidate the intermediates and transition states involved in this reaction, including the nature of the deprotonated species and the potential involvement of metal coordination or H-bonding for TEMPO-promoted oxidation.^9a,37–39^

### Substrate Scope

We next investigated the generality of this method using the conditions identified from the three model substrate classes (designated as Conditions A, B, and C). The substrates shown in Table 1a show the electronic, steric, and functional group tolerance on a range of building block alkylarenes. Primary, secondary, and tertiary benzylic C–H bonds are readily oxidized for a series of *N*-heteroarenes, including pyridine, pyrazine, and thiazole derivatives (**4**–**8**). Primary and secondary C–H bonds on electron-poor to electron-rich alkylbenzene derivatives are also oxidized, including biaryl, benzothiophene, indole, benzoxadiazole, and benzothiadiazole variants (**9**–**16**). Numerous base-sensitive functionalities are also tolerated, including nitriles (**16**), trifluoromethylthioethers (**17**), elimination-prone amino groups (**18**), unprotected primary amines (**19**, **20**), and electron-deficient aryl bromides (**22**). We also note that substrate **21** provides a meaningful yield (24%) with significant mass balance retained, indicating the tolerance of aryl pinacol boronic ester groups.

**Table 1:**
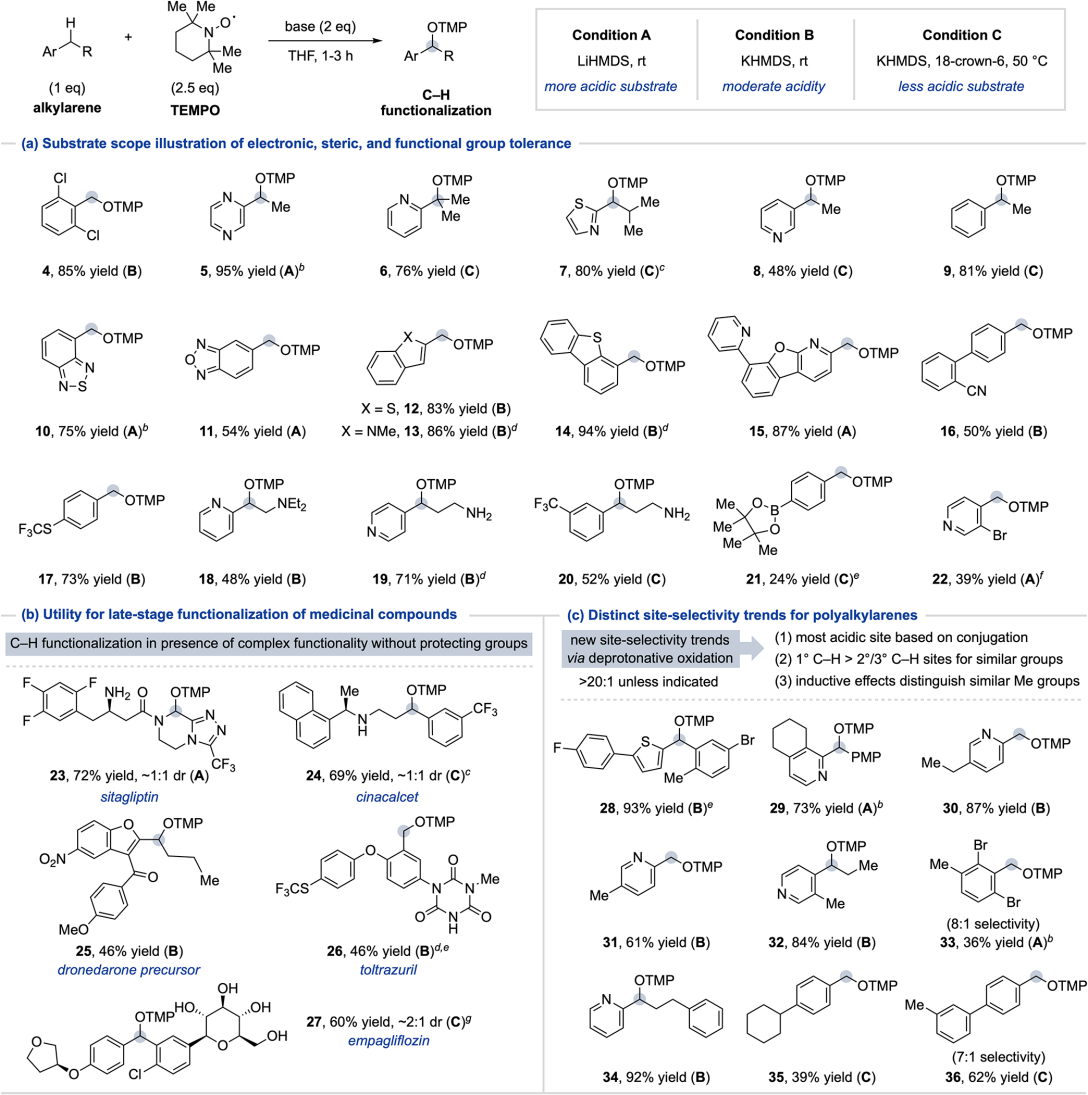
Substrate Scope and Applications of Deprotonative Benzylic C–H Oxidative Coupling with TEMPO^a^

a Isolated yields from 1 mmol scale reaction of alkylarene.

b NaHMDS was used.

c Reaction at rt.

d Reaction at 50 °C.

e Yield determined by ^1^H NMR spectroscopy: 45% isolated for **28**.

f Isolated yield lower than Figure 3a due to coelution with TEMPOH.

g An additional equivalent of base used in DMF.

The broad functional group tolerance of this process suggested that it could be suitable for the late-stage installation of the −OTMP group onto complex molecules without the use of protecting groups. Table 1b illustrates this utility on pharmaceuticals and other medicinally relevant compounds. This includes the direct functionalization of sitagliptin (**23**), cinacalcet (**24**), toltrazuril (**26**), empagliflozin (**27**), and a 2-alkyl-3-acylbenzofuran (**25**) drug substructure (e.g., dronedarone). These compounds illustrate that unprotected benzyl amines, β-amino amides, polyfluoroarenes, isocyanuric acid N–H, nitroarenes, diarylketones, and alcohols are well tolerated. The functionalization of empagliflozin (**27**) is especially notable as its glucosyl unit possesses four unprotected alcohols, and the product can be purified by simple precipitation.^40^

The collection of substrates in Table 1a and b demonstrates the unique features of a transient deprotonation/oxidation approach to radical generation. First, the tolerance of many base-sensitive and acidic functional groups contrasts with traditional stoichiometric metalation chemistry, which relies on very strong bases and is not typically suitable for complex or less-acidic alkylarenes.^14^ Second, the functionalization of *N*-heteroarenes and electron-deficient benzenes overcomes a common limitation of single-electron oxidation-based activation strategies as these substrates often possess prohibitively high oxidation potentials.^8,15^ Third, compared to HAA approaches, this reaction selectively operates in the presence of weak C–H bonds, such as tertiary variants and those adjacent to alcohols, ethers, and amines.^5,7^ Finally, we note that numerous oxidatively sensitive groups that are often incompatible with strong oxidants do not interfere in this process, including electron-rich arenes, various *N*-containing groups, primary alcohols, thioethers, and boronic esters.^16^

Another distinguishing feature of this method is the new set of site-selectivity trends in the functionalization of polyalkylarenes. As evidenced by the substrates in Table 1c (**28**–**36**), this reaction proceeds with good selectivity for the most acidic benzylic position, even in cases with only minor electronic differences (see also Scheme 1). Thus, using the initially developed conditions, selective mono-oxidation occurs at positions in conjugation with resonance-stabilizing groups or is otherwise guided by minor inductive differences (i.e., 1° over 2°/3° positions and proximity to electronegative groups). Alternative approaches to radical generation from C–H bonds typically either have opposing selectivity (e.g., 2° over 1°) or, in many cases, give regioisomeric mixtures.^1^ This is especially true for commonly used halogenation methods where the differences in C–H bond strengths and polar effects are insufficient for site-selective reactivity; these reactions also often generate undesired polyhalogenated products.^6,35,41^ We note that selective HAA has recently been achieved for *N*-heteroaryl secondary benzylic positions through a transient metal coordination process to produce aryl ketone products.^42^

**Scheme 1:**
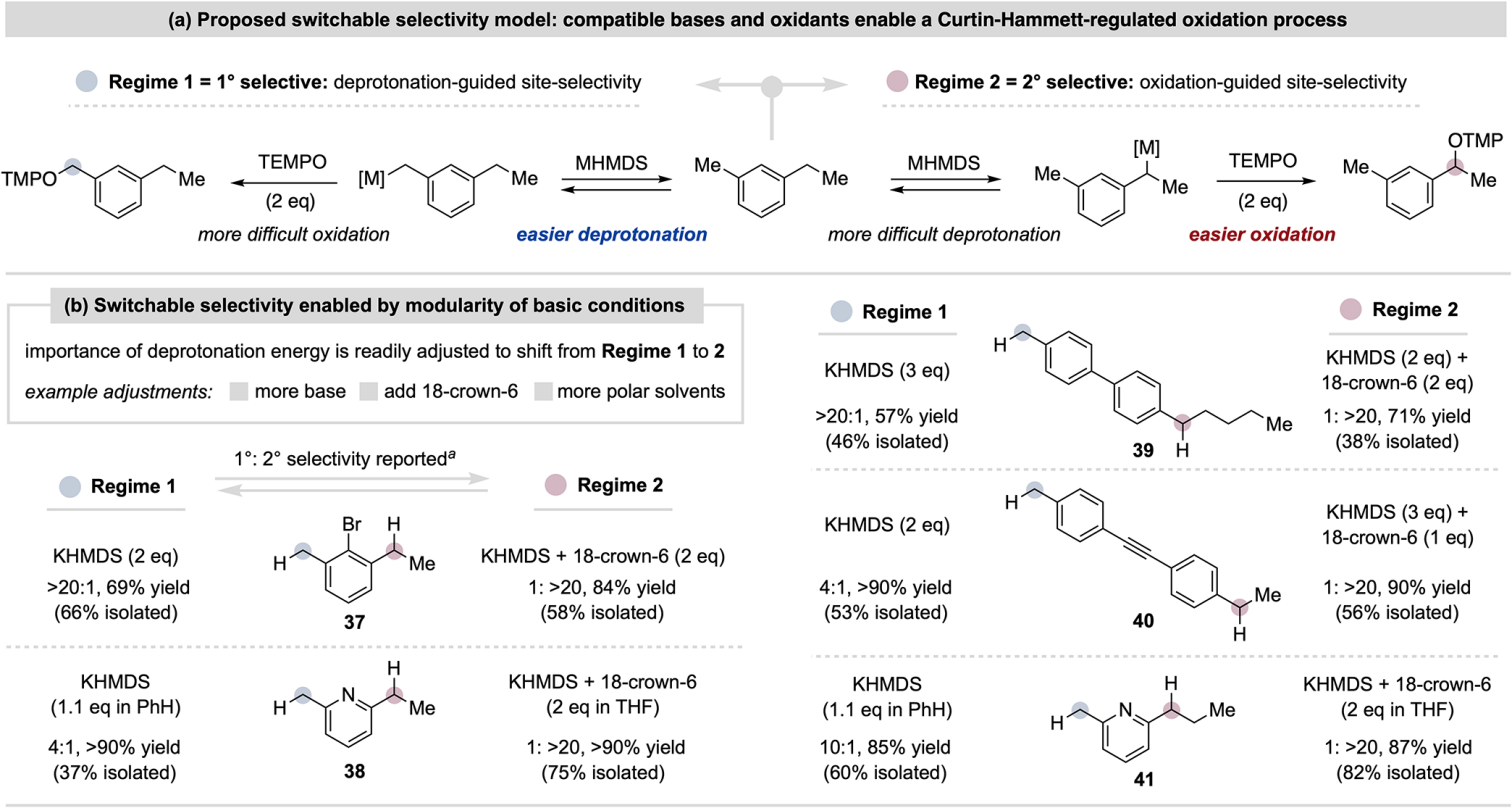
Demonstration of Switchable Selectivity Enabled by Simple Condition Variation.^a^

a Reactions run under Table 1 conditions; yields and selectivities assessed by ^1^H NMR spectroscopy of the crude reaction mixtures, with isolated yields in parentheses.

More broadly, the ability to control and switch positional selectivity on unbiased polyalkylarene substrates using simple reagents is a largely unsolved problem.^1,7^ We rationalized that the *in situ* combination of bases and single-electron oxidants could allow for modular and independent adjustments to be made to the barriers for deprotonation and oxidation, thereby allowing either step to dictate selectivity (Scheme 1a). The prospect for switchable selectivity is supported by the trend that, in general, the more easily a C–H bond is deprotonated, the harder the resulting carbanionic intermediate is to oxidize, and vice versa.^22,26^ However, in practice, this would require deprotonation and oxidation to be similar enough in energy such that either step could be made selectivity-determining. We therefore tested this proposal on a variety of polyalkylarene substrates, especially those with positions that are less electronically biased than the substrates shown in Table 1c. Notably, these substrates cannot be stoichiometrically metalated with positional selectivity, indicating that this approach is also distinguished from traditional deprotonative activation.^14^

We discovered that simple adjustments to the base strength or reaction conditions can be used to invert the selectivity from the most acidic to the least acidic alkyl group in dialkylarenes (Scheme 1b).^43^ In particular, a switch from primary (typically using Condition B) to secondary positions can be accomplished through the addition of 18-crown-6 or an increase in solvent polarity. This was first observed in 1-bromo-2-ethyl-6-methylbenzene (**37**) where the addition of 18-crown-6 completely inverts C–H oxidation to the ethyl group. We next studied 2-ethyl-6-methylpyridine (**38**), a substrate we anticipated to be more challenging to control because both alkyl groups are relatively acidic. We found that the use of nonpolar benzene as a solvent primarily functionalizes the methyl group (4:1 selectivity), while the use of 18-crown-6 in THF gives >20:1 ethyl selectivity. Thus, controllable selectivity is possible across a wide acidity spectrum, and the 1°-to-2° switchable generality is demonstrated by additional examples of -ethyl (**40**), -propyl (**41**), and -*n*-pentyl (**39**) functionalization. We hypothesize that these changes effectively lower the deprotonation energy such that the TEMPO-promoted oxidation step becomes selectivity-determining, although studies are ongoing to understand the full origin of selectivity. From a mechanistic perspective, the observation that a change in base conditions switches selectivity is most consistent with deprotonation and oxidation operating as distinct steps, rather than a concerted process.^9,37^ We expect that these and related condition adjustments will be an enabling and broadly applicable capability for targeting various positions of polyalkylarenes as desired.

## Conclusions

In summary, the combination of uphill deprotonation with single-electron oxidation by a weak oxidant, such as TEMPO, serves as a new platform for benzylic C–H oxidation that is guided by C–H acidity and carbanion oxidation trends. This work demonstrates how this approach provides valuable synthetic opportunities over traditional means of radical generation, namely in new site-selectivity trends and regulation, as well as alternative substrate and functional group tolerances. These features are derived from simple and abundantly available reagents used under practical conditions. Given the wide variety of basic reagents and single-electron oxidants that could be strategically combined, we anticipate that this approach can serve as a general and controllable C–H activation strategy. In this regard, our group is currently studying the details of this method in an effort to expand deprotonative single-electron oxidation to a broader range of C–H bond types and coupling reactions.

## Supplementary Material



## Notes

Detailed experimental procedures, spectral, and characterization data of all new compounds (PDF)
