# Supplementary material for: Deprotonative Single-Electron Oxidation as a General and Controllably Selective Platform for Benzylic C–H Functionalization
Source: J Am Chem Soc. 2026 May 28;148(22):22962–70. doi: 10.1021/jacs.6c04370 (PMC13266974; doi:10.1021/jacs.6c04370)
Supplement: Supplementary file 1 [file ja6c04370_si_001.pdf]

Supporting Information

Deprotonative Single Electron Oxidation as a General and Controllably Selective Platform for Benzylic C–H Functionalization

Nicholas J. Coradi and Jeffrey S. Bandar

Department of Chemistry, Colorado State University

Fort Collins, Colorado 80523, United States

Email: jeff.bandar@colostate.edu

Table of Contents

I.	General Information	S2
II	Experimental Optimization for Base-Promoted C–H TEMPO Coupling	S3
III.	Experimental General Procedures for Base-Promoted C–H TEMPO Coupling	S10
IV.	Characterization Data of Products	S11
V.	Determination of Site Selectivity	S28
VI.	Mechanistic Studies and Control Reactions Experimental Details	S32
VII.	References	S57
VIII.	NMR Spectra	S58

I. General Information

General Reagent Information: All reactions were conducted under nitrogen gas unless otherwise noted. 2,2,6,6-Tetramethylpiperidine 1-oxyl (TEMPO, ChemScene, catalog # CS-W001187) was purchased and used as received. Potassium bis(trimethylsilyl)amide (KHMDS, MilliporeSigma, catalog #324671) was purchased as a 95% pure powder, sodium bis(trimethylsilyl)amide (NaHMDS, MilliporeSigma, catalog # 235083) was purchased as a 95% pure powder, lithium bis(trimethylsilyl)amide (LiHMDS, MilliporeSigma, catalog # 324620) was purchased as a 97% pure powder and were used as received. Potassium bis(trimethylsilyl)amide (1M in THF, TCI, catalog #P3032), sodium bis(trimethylsilyl)amide solution (1M in THF, MilliporeSigma, catalog #245585), and lithium bis(trimethylsilyl)amide solution (1M in THF, MilliporeSigma, catalog #225770) were stored in a desiccator at room temperature. Tetrahydrofuran (THF, anhydrous) was deoxygenated and dried by passage over packed columns of neutral alumina and copper (II) oxide under positive pressure of N_2. *N*,*N*-Dimethylformamide (DMF, anhydrous, MilliporeSigma, catalog #227056) was stored at room temperature (rt) inside a N_2 filled glovebox and was used as received. All other solvents and reagents were purchased from Millipore Sigma, Combi-Blocks, TCI, Acros Organics, Matrix Scientific, AlfaAesar, or ChemScene and were used as received, unless otherwise noted. All solid bases and 1,4,7,10,13,16- hexaoxacyclooctadecane-6 (18-crown-6, Chem-Impex catalog #03901) were stored at rt inside a N_2 filled glovebox and used immediately if brought outside the glovebox. TEMPO was stored in a N_2 filled glovebox freezer at -30 °C. TEMPO aliquots removed from the glovebox were stored in a 20 mL, parafilm wrapped, flame-dried scintillation under a headspace of N_2 at -32 °C in a freezer. TEMPO removed from the glovebox was used within one week of removal. Within 10 minutes of each use, the scintillation vial containing TEMPO was flushed with N_2 gas from a manifold Schlenk line, sealed with a screwcap, wrapped in parafilm, and stored in a -32 °C freezer. We found that KHMDS (1M solution in THF, MilliporeSigma, catalog # 324671) contains toluene, and while an effective base for this protocol, undesired toluene functionalization can occur. Additionally, we note that KHMDS (1M solution in THF, TCI, catalog #P3032) contains impurities that are observable *via* ^{1}H NMR spectroscopy (4.5 ppm – 6.4 ppm); however, the performance of this base solution is similar to the use of pure, solid base. Flash column chromatography was conducted with 40-63 µm silica gel (SiliaFlash® F60 from Silicycle). Preparative thin-layer chromatography (PTLC) was conducted with silica gel 60 Å F254 plates (20 x 20 cm, 1000 µm, SiliaPlate from Silicycle, #TLG-R10011B-341) and visualized with UV light (254 nm). Celite® 545 (Product #CX0574-3) was purchased from Millipore Sigma. Automated flash column chromatography was conducted on a Biotage® Selekt Enkel (Biotage, SEL-ESV).

General Analytical Information: All reported compounds were characterized by ^{1}H, ^{13}C, and ^{19}F (if appropriate) NMR spectroscopy, FTIR spectroscopy, and high-resolution mass spectrometry. Melting point analysis was conducted if the compound was solid. ^{1}H NMR, ^{13}C NMR, and ^{19}F NMR spectra were obtained on a Bruker NEO400, Bruker US400, Bruker Ascend 400, or Bruker AVANCE NEO 400 spectrometer. ^{1}H NMR spectroscopic data is reported as follows: chemical shift (δ ppm), multiplicity (if applicable, s = singlet, br s = broad singlet, d =doublet, t = triplet, q = quartet, p = pentet, h = hextet, hept = heptet, dd = doublet of doublets, ddd = doublet of doublet of doublets, dddd = doublet of doublet of doublet of doublets, dt = doublet of triplets, td = triplet of doublets, dq = doublet of quartets, qd = quartet of doublets, ddt = doublet of doublet of triplets, tt = triplet of triplets, dtd, doublet of triplet of doublets, m = multiplet), coupling constant (Hz), and integration. All ^{1}H NMR spectrum signals are reported as chemical shifts (δ ppm) relative to residual $CDCl_3$ at 7.26 ppm or tetramethylsilane (TMS) at 0.00 ppm. ^{13}C NMR spectroscopic data is reported as follows: chemical shift (δ ppm), multiplicity (if applicable, d = doublet, t = triplet, q = quartet, quin = quintet, dq = doublet of quartets, qd = quartet of doublets, m = multiplet), and coupling constant (Hz). ^{13}C NMR signals are reported as chemical shifts (δ ppm) relative to $CDCl_3$ at 77.16 ppm or CD_3CN at 118.26 ppm.[1] Chemical shifts (δ ppm) for ^{19}F NMR are reported in terms of chemical

shift in reference to an internal standard (1,4-difluorobenzene set to δ -119.7 ppm).[2] ^{19}F NMR spectroscopic data is reported as follows: chemical shift (δ ppm), multiplicity (d = doublet, t = triplet, dt = doublet of triplet, br s = broad singlet, m = multiplet), and coupling constant (Hz). High resolution mass spectra (HRMS) were recorded on an Agilent 6230 LC-MS B-TOF equipped with a dual ESI source provided by the Colorado State University Analytical Resource Core – Molecular and Materials Analysis Center. IR spectra were recorded using a Thermo Scientific Nicolet iS-50 FTIR Spectrometer and reported as frequency of absorption (cm^{-1}). Melting point analyses were conducted using a MelTemp capillary melting point apparatus. Thin-layer chromatography analysis was conducted with silica 60 Å F254 plates (250 μm, SiliaPlate from Silicycle, #TLG-R10014B323) and interpreted using UV light (254 nm) or $KMnO_4$ stain.

Nomenclature Note: The names provided for the structures in this document were obtained from ChemDraw Professional 23.1.1.3.

II. Experimental Optimization for Base-Promoted C–H TEMPO Coupling

a) Evaluation of changes in base identity and equivalents, TEMPO equivalents, and solvent for model substrate 1 (3-bromo-4-methylpyridine).

Discussion. Experiments varying base and solvent indicated that TEMPO and LiHMDS promotes the coupling of **1** with TEMPO in THF at rt. A variety of condition and reagent variations are shown in Table S1 to inform readers of their impacts on the reaction yield.

General procedure for condition variation: 3-Bromo-4-methylpyridine (**1**) (17.2 mg, 0.1 mmol, 1.0 equiv) was added *via* pipette to a 4 mL, oven-dried dram vial (KIMBLE®, #60910-1) containing a stir bar. The vial was sealed with a screw cap (Thermo Fisher Scientific, #C4015-1A) lined with a PTFE septum (Thermo Fisher Scientific, #B7995-13) and was brought into a N_2 filled glovebox. The vial was unsealed and THF (0.25 mL, 0.4 M) was added *via* micropipette. TEMPO (39.1 mg, 0.25 mmol, 2.5 equiv) was then added followed by solid LiHMDS (33.5 mg, 2.0 mmol, 0.2 mmol). The vial was resealed with the screwcap, removed from the glovebox, placed into an aluminum reaction block, and was stirred at rt for 1 hour. At this time the vial was unsealed and methanol (MeOH, 0.1 mL, 2.47 mmol, 24.7 equiv) was added *via* syringe. 1,3,5-Trimethoxybenzene (TMB) was then added to the quenched crude reaction mixture. The mass of TMB added to the vial for each experiment was weighed and recorded separately. A small aliquot from the crude reaction mixture was removed, charged into an NMR tube, and constituted in $CDCl_3$ (0.5 mL). ^{1}H NMR spectroscopy (400 MHz, $CDCl_3$) was used to determine the yield of **22**. The aromatic proton signal of TMB (s, 3H) at 6.09 ppm was integrated against the benzylic proton signal of **22** at 4.87 ppm (s, 2H) to determine the yield. The results from these experiments are summarized in Table S1 below. A representative ^{1}H NMR spectrum for this reaction is provided in Figure S1 below. While optimized conditions for 3-bromo-4-methylpyridine gave optimal yield of **22** after 1 hour, all preparative scale reactions were conducted for 2 hours (unless otherwise noted) to account for potential variation in reaction time for each individual alkylarene substrate.

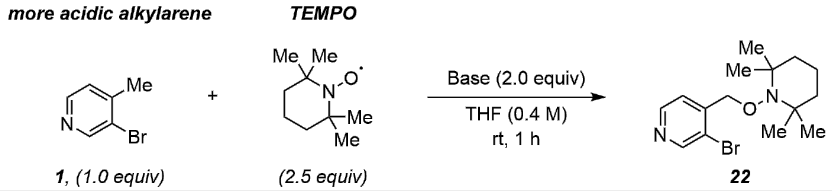

Entry	Conditions and Changes From Above Scheme	Yield
1	KHMDS	0%
2	NaHMDS	36%
**3**	**LiHMDS**	**87%**
4	LiHMDS (1.5 eq)	79%
5	LiHMDS, 2.0 eq TEMPO	80%
6	LiHMDS, 2.2 eq TEMPO	84%
7	LiHMDS (0.2 M instead of 0.4 M)	85%
8	KOH	0%
9	NaOTMS	0%
10	KO-*t*-Bu	2%
11	(LiHMDS) DMF instead of THF	0%
12	(LiHMDS) DMI instead of THF	1%
13	(LiHMDS) DME instead of THF	24%
14	(LiHMDS) Benzene instead of THF	7%
15	KO-*t*-Bu, DMSO instead of THF	4%
16	LiHMDS, 1.0 eq of TEMPO instead of 2.5 eq	29%

Table S1: Condition variation for coupling of 3-bromo-4-methylpyridine (**1**) with TEMPO.

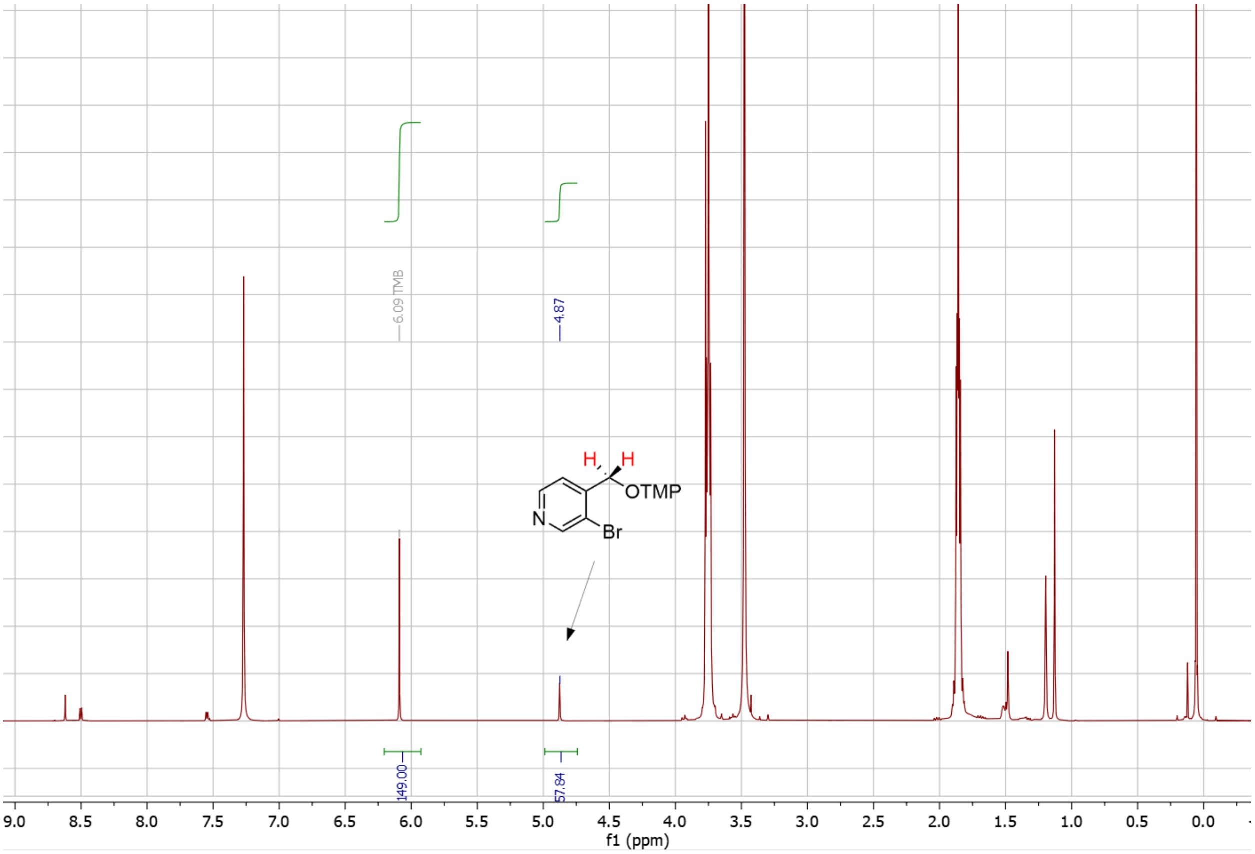

Figure S1: Representative ^1H NMR spectrum of the crude reaction solution from condition variation studies. The reaction was conducted using LiHMDS (31.3 mg, 0.2 mmol, 2.0 equiv) and TEMPO (39.1 mg, 0.25 mmol, 2.5 equiv) (Table S1 entry 3). TMB (25.0 mg, 149 µmol, signal at 6.09 ppm) was used to determine the yield of **22** (86.8 µmol, 87% yield).

b) Optimization of TEMPO adduct formation: Evaluation of changes in base identity and equivalents, TEMPO equivalents, and solvent for model substrate 2 (1,3-dichloro-2-methylbenzene).

Discussion. Experiments varying base and solvent indicated that TEMPO and KHMDS promotes the coupling of 1,3-dichloro-2-methylbenzene (**2**) with TEMPO in THF at rt in optimal yield. A variety of condition and reagent variations are shown in Table S2 to inform readers of their impacts on the reaction yield.

General procedure for condition variation: the general procedure described above for alkylarene **1** was followed with 1,3-dichloro-2-methylbenzene (**2**) (16.1 mg, 0.1 mmol, 1.0 equiv) used in place of 3-bromo-4-methylpyridine (**1**). A representative ^1H NMR spectrum for this reaction is provided in Figure S2 below to show how reaction yield was assessed.

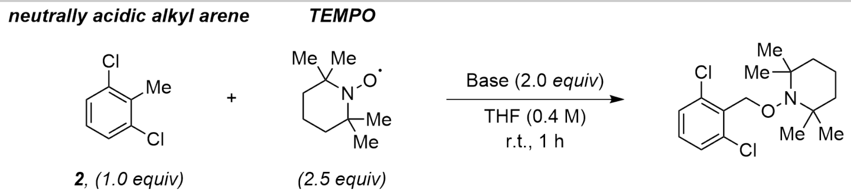

Entry	Conditions and Changes From Above Scheme	Yield
1	LiHMDS	3%
2	NaHMDS	51%
3	NaHMDS (17 h)	92%
**4**	**KHMDS**	**94%**
5	1.0 equiv KHMDS	59%
6	1.5 equiv KHMDS	88%
7	(KHMDS) 2.0 equiv TEMPO	81%
8	(KHMDS) 1.0 equiv TEMPO	50%
9	KO-*t*-Bu	0%
10	(KHMDS) DMF instead of THF	34%
11	(KHMDS) DME instead of THF	82%
12	(KHMDS) Benzene instead of THF	59%
13	(KHMDS) DMI instead of THF	40%
14	KHMDS solution (1.0 mmol scale)	95%
15	reaction run under ambient atmosphere instead of under N_2	87%

Table S2: Condition variation for coupling of 1,3-dichloro-2-methylbenzene (**2**) with TEMPO.

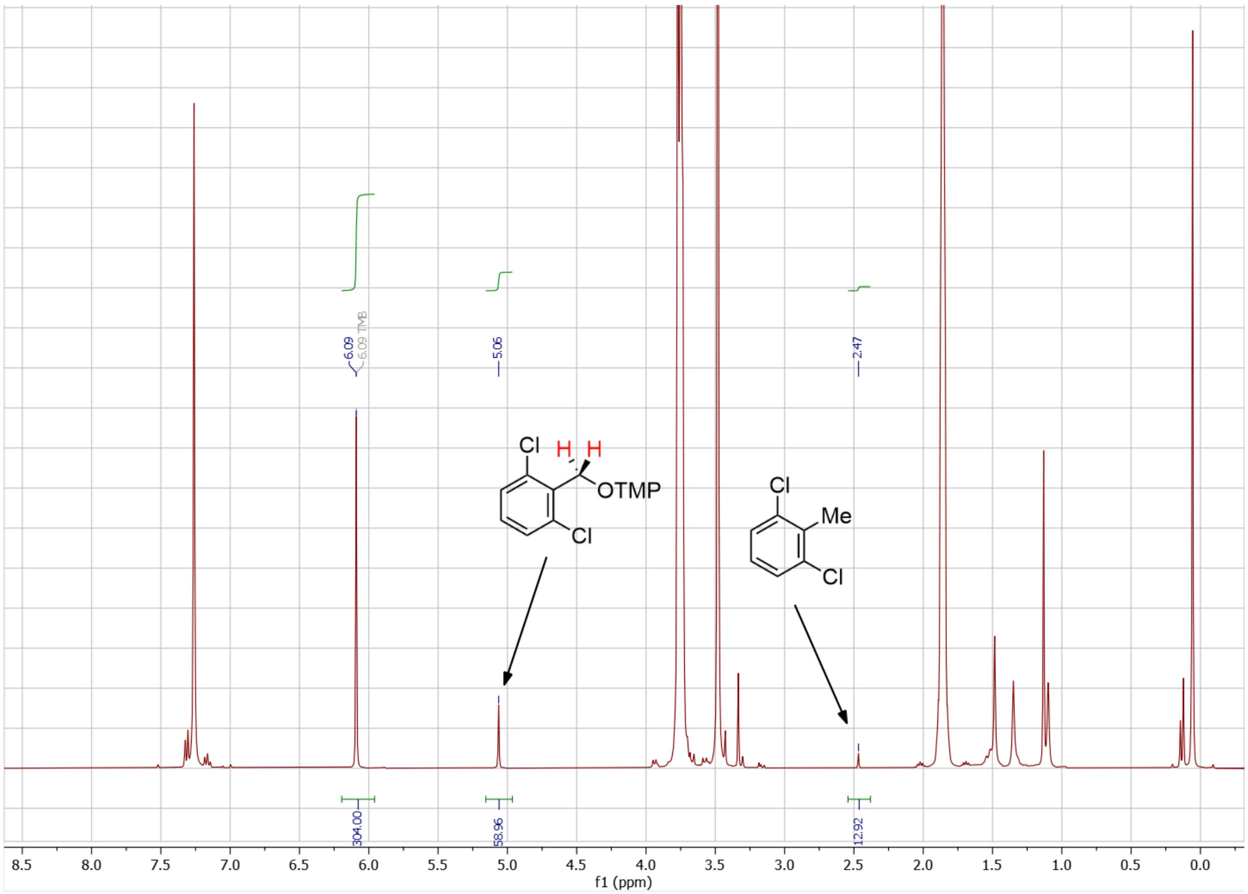

Figure S2: Representative ^{1}H NMR spectrum of the crude reaction solution from condition variation studies. The reaction was conducted using KHMDS (29.9 mg, 0.15 mmol, 1.5 equiv) and TEMPO (39.1 mg, 0.25 mmol, 2.5 equiv) (Table S2 entry 6), to show both starting material and product. TMB (51.2 mg, 304 µmol, signal at 6.09 ppm) was used to determine the yield of **4** (88.4 µmol, 88% yield).

c) Evaluation of changes in base identity and equivalents, TEMPO equivalents, solvent, reaction time, and temperature for model substrate 3 (ethylbenzene).

Discussion. Experiments varying base and solvent indicated that TEMPO and KHMDS with 18-crown-6 additive promotes the coupling of ethylbenzene (**3**) with TEMPO in THF at 50 °C. A variety of condition and reagent variations are shown in Table S3 to inform readers of their impacts on the reaction yield.

General procedure for condition variation: the general procedure described above was followed with ethylbenzene (**3**, 10.6 mg, 0.1 mmol, 1.0 equiv) used in place of 3-bromo-4-methylpyridine (**1**). Additionally, after addition of THF (0.25 mL, 0.4 M), 18-crown-6 (18c6, 52.9 mg, 0.2 mmol, 2.0 equiv) was added. Furthermore, after the vial was removed from the glovebox, it was placed in a reaction block preheated to 50 °C with stirring. A representative ^{1}H NMR spectrum for this reaction is provided in Figure S3 below to show how product analysis was performed.

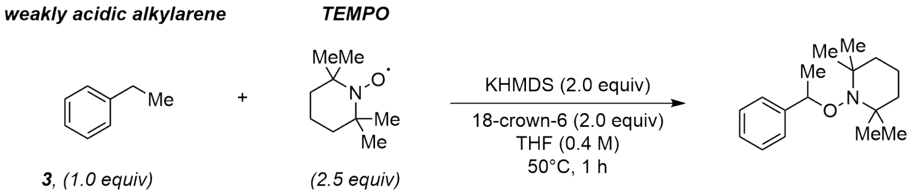

Entry	Conditions and Changes From Above Conditions	Yield
1	None	59%
2	3.0 equiv KHMDS instead of 2.0 equiv	63%
3	1.0 equiv 18-crown-6 instead of 2 equiv	23%
4	3 h instead of 1 h	80%
5	no 18c6	0%
6	r.t. instead of 50°C (3h)	36%
7	1.0 eq TEMPO instead of 2.5 equiv	40%
8	24 h instead of 1 h	89%
9	DMF instead of THF (3h)	32%
10	Dioxane instead of THF (3 h)	55%
11	Benzene instead of THF (3 h)	71%
12	DMPU instead of THF (3 h)	71%

Table S3: Condition variation for coupling of ethylbenzene (**3**) with TEMPO.

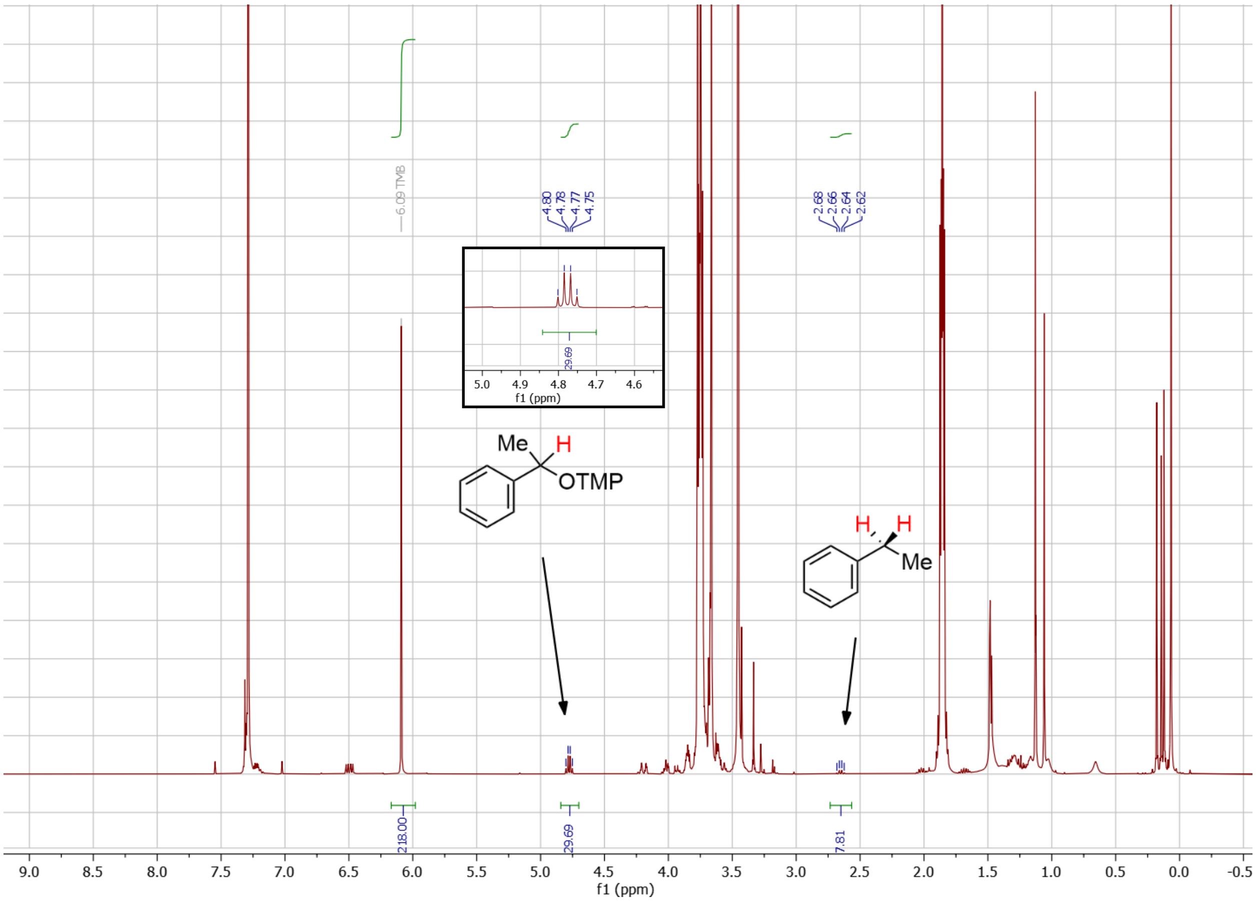

Figure S3: Representative ^{1}H NMR spectrum of the crude reaction solution from condition variation studies. The reaction was conducted using KHMDS (39.9 mg, 0.2 mmol, 2.0 equiv), TEMPO (39.1 mg, 0.25 mmol, 2.5 equiv), and 18-crown-6 (52.9 mg, 0.2 mmol, 2.0 equiv) for 24 hours (Table S3 entry 8). TMB (36.7 mg, 218 µmol, signal at 6.09 ppm) was used to determine the yield of **9** (89.1 µmol, 89% yield) and quantify remaining starting material (ethylbenzene, 11.7 µmol, 12% remaining).

d) Selection of conditions for alkylarenes in Table 1 and selection of conditions for switchable selectivity in Scheme 1

Discussion: Following optimization of each representative alkylarene, we began exploring the generality of this protocol for a wide range of substrates with the goal of demonstrating tolerance of diverse functionality and electronic characteristics. Conditions were selected with the following considerations in mind: for more acidic substrates (e.g., those with electron-withdrawing groups in conjugation with the benzyl anion) **Conditions A** were tested. If those conditions resulted in low yield and high mass balance, **Conditions B** and **Conditions A** with NaHMDS (stronger base conditions) were tested. For moderately acidic alkylarenes (e.g., those bearing minor inductive EWGs, those bearing EWGs out of conjugation with the carbanion, or relatively electron-neutral heteroarenes), **Conditions B** were selected. If those conditions resulted in moderate yield and high mass balance **Conditions B** at 50 °C were tested. If low yield and high mass balance was observed, **Conditions C** were tested. If **Conditions B** resulted in moderate / low yield and poor mass balance, **Conditions A** (LiHMDS and NaHMDS) were tested. **Conditions C** were selected

for electron-neutral arenes with no withdrawing groups. To identify conditions for switchable selectivity, **Conditions B** were first employed. If the result of **Conditions B** resulted in a mixture, addition of 18-crown-6 (**Conditions C** at rt for 2 h) enriched the selectivity for the secondary position. To switch the selectivity to 1° if **Conditions B** resulted in a mixture, KHMDS (1.1 equiv) in toluene was selected. In all observed cases, 2° selectivity is favored with stronger base conditions (e.g., chelating additives or more polar solvents) and 1° selectivity is favored with weaker base conditions in non-polar solvents.

III. Experimental General Procedures for Base-Promoted C–H TEMPO Coupling

General Procedure 1 (GP1) for alkylarene C–H coupling with TEMPO (more acidic to moderately acidic alkylarenes): To an oven-dried, 7.5 mL dram vial (Fisher, #14-955-326) with a stir bar, TEMPO (390.8 mg, 2.5 mmol, 2.5 equiv) was added. If the alkylarene (1.0 mmol, 1.0 equiv) is a solid, it was also added to the vial at this time. **Note:** we found that the solid mixture of alkylarene and TEMPO can form a liquid mixture when combined and note that formation of this mixture does not impact subsequent reactivity. The vial was sealed with a screwcap lined with a PTFE septum and using a needle connected to a Schlenk line manifold, the vial was evacuated and backfilled three times with anhydrous nitrogen gas and left under a positive pressure of nitrogen gas. Anhydrous THF (0.5 mL) was added *via* syringe and the solution was stirred. At this time, if the alkylarene (1.0 mmol, 1.0 equiv) is a liquid, it was added to the vial *via* syringe. KHMDS, NaHMDS, or LiHMDS (1M solution in THF, 2.0 mL, 2.0 mmol, 2.0 equiv) was then added *via* syringe. The needle connected to the Schlenk line was removed and the reaction mixture was stirred at rt (unless otherwise noted) for 2 hours. **Note:** If the reaction is conducted at 50 °C, the screwcap and septum were wrapped in electrical tape and parafilm before placing the vial in a preheated reaction block. After 2 h, the vial was unsealed and MeOH (1.0 mL, 24.7 mmol, 24.7 equiv) was added *via* syringe to quench the reaction mixture. 4-(Trifluoromethyl)pyridine standard was then measured into the crude solution *via* pipette and the mass of standard added was recorded. A small aliquot of the reaction mixture was charged into an NMR tube *via* pipette and constituted in $CDCl_3$ (~0.5 mL). ^1H NMR spectroscopy (400 MHz) was used to determine the yield of the product. The NMR sample and crude reaction mixed were combined in a 125 mL separatory funnel with ethyl acetate (EtOAc, 10 mL). H_2O (50 mL) was added and the mixture was extracted with EtOAc (3 x 50 mL). The combined organic layers were dried over Na_2SO_4, filtered, and concentrated *in vacuo*. The resulting crude residue was purified *via* silica flash column chromatography according to eluent conditions provided. **Note:** A 10 second relaxation delay was used to acquire quantitative ^1H NMR data.

General Procedure 2 (GP2) for alkylarene coupling with TEMPO (less acidic alkylarenes): GP1 was followed except that after TEMPO addition, 18-crown-6 (528.6 mg, 2.0 mmol, 2.0 equiv) was also added. The reaction mixture was stirred for 3 hours at 50 °C instead of 2 hours at rt unless otherwise noted.

General Procedure 3 (GP3) for alkylarene coupling with TEMPO (for reactions that use benzene as a solvent): To a 7.5 mL, oven-dried dram vial (vial A) with a stir bar, KHMDS (219.5 mg, 1.1 mmol, 1.1 equiv) was added in a N_2 filled glovebox. To the vial, benzene (2 mL) was added *via* micropipette to give a turbid slurry. The vial was sealed with a screwcap lined with a PTFE septum, removed from the glovebox and the solution was stirred over a stir plate. Vial A was then connected to a manifold Schlenk line *via* a needle and was left under a positive pressure of nitrogen gas. In a separate 7.5 mL, oven-dried dram vial, (vial B), TEMPO (390.8 mg, 2.5 mmol, 2.5 equiv) was added. If the alkylarene (1.0 mmol, 1.0 equiv) is a solid, it was also added at this time. Vial B was then sealed with a screwcap and septum, and using a needle connected to a Schlenk line manifold, the vial was evacuated and backfilled three times with anhydrous

nitrogen gas and left under a positive pressure of nitrogen gas. To vial B, benzene (0.25 mL) was added *via* syringe. If the alkylarene (1.0 mmol, 1.0 equiv) is a liquid, it was added at this time to vial B *via* syringe. The solution in vial B solution was taken up into a syringe. Vial B was then rinsed with benzene (0.25 mL *via* syringe) and the solution was taken up into the syringe containing the TEMPO and alkylarene solution. This solution was then added to vial A at once to give a homogenous reaction mixture. The reaction mixture was stirred for 2 hours at room temperature. The reaction assessment and isolation procedure is identical to **GP1**.

IV. Characterization Data of Products

1-((2,6-dichlorobenzyl)oxy)-2,2,6,6-tetramethylpiperidine (4)

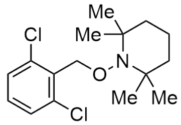

The title product was prepared according to **GP1** using 1,3-dichloro-2-methylbenzene (**1**) (161.0 mg, 1.0 mmol), TEMPO (390.8 mg, 2.5 mmol, 2.5 equiv), KHMDS (1 M solution in THF, 2.0 mL, 2.0 mmol, 2.0 equiv), and anhydrous THF (0.5 mL) for 1 hour. The crude residue was filtered through silica with 2% diethyl ether (Et_2O) in hexanes to afford the product as a white solid (270.1 mg, 0.85 mmol, 85% yield). **MP:** 69-70 °C. **^{1}H NMR** (400 MHz, $CDCl_3$) δ 7.31 (d, J = 8.0 Hz, 2H), 7.15 (dd, J = 8.5, 7.5 Hz, 1H), 5.06 (s, 2H), 1.68 – 1.42 (m, 5H), 1.68 – 1.42 (m, 7H), 1.10 (s, 6H). **^{13}C NMR** (101 MHz, $CDCl_3$) δ 136.9, 133.9, 129.6, 128.6, 73.4, 60.2, 40.3, 33.5, 20.2, 17.3. **IR** (neat, cm^{-1}) 3006, 2920, 2847, 2361, 2341, 1581, 1562, 1435, 1376, 1361, 1352, 1153, 1025, 988, 857, 780, 763. **HRMS** (ESI) $[M+H]^+$ calcd. for $[C_{16}H_{24}Cl_2NO]^+$ = 316.1230, 316.1256 found.

2-(1-((2,2,6,6-tetramethylpiperidin-1-yl)oxy)ethyl)pyrazine (5)

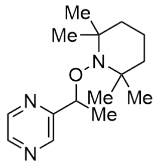

The title product was prepared according to **GP1** using 2-ethylpyrazine (108.1 mg, 1.0 mmol), TEMPO (390.8 mg, 2.5 mmol, 2.5 equiv), NaHMDS (1 M solution in THF, 2.0 mL, 2.0 mmol, 2.0 equiv), and anhydrous THF (0.5 mL). The product was purified *via* silica flash column chromatography with the eluent conditions 10% EtOAc → 20% EtOAc in hexanes to afford the product as a bronze oil (250.5 mg, 0.95 mmol, 95% yield). **^{1}H NMR** (400 MHz, $CDCl_3$) δ 8.73 (d, J = 1.5 Hz, 1H), 8.52 (dd, J = 2.6, 1.5 Hz, 1H), 8.45 (d, J = 2.5 Hz, 1H), 4.99 (q, J = 6.8 Hz, 1H), 1.57 (d, J = 6.8 Hz, 3H), 1.54 – 0.83 (m, 15H), 0.51 (s, 3H). **^{13}C NMR** (101 MHz, $CDCl_3$) δ 158.8, 143.6, 143.1, 142.6, 81.7, 59.5, 59.1, 39.7, 33.8, 33.4, 20.6, 19.8, 16.6. **IR** (neat, cm^{-1}) 2974, 2930, 2871, 1471, 1453, 1400, 1375, 1360, 1258, 1242, 1155, 1132, 1063, 1016, 935, 849, 715. **HRMS** (ESI) $[M+H]^+$ calcd. for $[C_{15}H_{26}N_3O]^+$ = 264.2071, 264.2076 found.

2-(2-((2,2,6,6-tetramethylpiperidin-1-yl)oxy)propan-2-yl)pyridine (6)

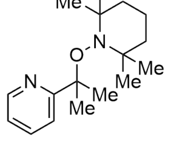

The title product was prepared according to **GP2** using 2-isopropylpyridine (121.2 mg, 1.0 mmol), TEMPO (390.8 mg, 2.5 mmol, 2.5 equiv), 18-crown-6 (528.6 mg, 2.0 mmol, 2.0 equiv), KHMDS (1 M solution in THF, 2.0 mL, 2.0 mmol, 2.0 equiv), and anhydrous THF (0.5 mL). The product was purified *via* preparatory thin layer chromatography, as the product and residual TEMPO exhibit poor resolution on silica. The crude residue was loaded onto a preparatory TLC plate and developed twice with 10% EtOAC in hexanes to afford the product

as a white solid (209.6 mg, 0.76 mmol, 76% yield). **MP:** 48-49 °C. **^{1}H NMR** (401 MHz, $CDCl_3$) δ 8.48 (ddd, *J* = 4.9, 1.9, 1.0 Hz, 1H), 7.74 – 7.59 (m, 2H), 7.09 (ddd, *J* = 7.3, 4.9, 1.3 Hz, 1H), 1.64 (s, 6H), 1.60 – 1.33 (m, 6H), 1.27 (dq, *J* = 12.4, 3.3 Hz, 6H), 1.11 (s, 7H), 0.82 (s, 6H). **^{13}C NMR** (101 MHz, $CDCl_3$) δ 168.7, 147.5, 135.8, 121.5, 121.3, 82.0, 59.4, 40.8, 33.7, 27.2, 20.6, 17.1. **IR** (neat, cm^{-1}) 3007, 2972, 2928, 1590, 1470, 1425, 1371, 1357, 1135, 1122, 926, 789, 750. **HRMS** (ESI) $[M+H]^+$ calcd. for $[C_{17}H_{29}N_2O]^+$ = 277.2275, 277.2289 found.

2-(2-methyl-1-((2,2,6,6-tetramethylpiperidin-1-yl)oxy)propyl)thiazole (7)

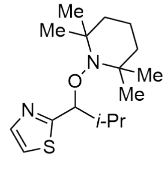

The title product was prepared according to **GP2** using 2-isobutylthiazole (141.2 mg, 1.0 mmol), TEMPO (390.8 mg, 2.5 mmol, 2.5 equiv), 18-crown-6 (528.6 mg, 2.0 mmol, 2.0 equiv), KHMDS (1 M solution in THF, 2.0 mL, 2.0 mmol, 2.0 equiv), and anhydrous THF (0.5 mL) at rt for 2 hours. The crude residue was filtered through silica with DCM (800 mL, discarded) followed by a flush with 2% MeOH in DCM (collected, concentrated *in vacuo*) to afford the product as an off-white solid (237.1 mg, 0.8 mmol, 80% yield). **MP:** 43-44 °C. **^{1}H NMR** (401 MHz, $CDCl_3$) δ 7.76 (d, *J* = 3.2 Hz, 1H), 7.28 (d, *J* = 3.3 Hz, 1H), 4.92 (d, *J* = 6.0 Hz, 1H), 2.76 – 2.47 (m, *J* = 6.8 Hz, 1H), 1.83 – 1.04 (m, 15H), 1.02 (d, *J* = 6.9 Hz, 3H), 0.89 (d, *J* = 6.8 Hz, 3H), 0.56 (s, 3H). **^{13}C NMR** (101 MHz, $CDCl_3$) δ 170.3, 141.3, 118.4, 87.5, 59.8, 40.1, 34.3, 32.4, 31.0, 20.2, 19.8, 19.1, 16.8, 16.6. **IR** (neat, cm^{-1}) 3096, 3080, 3010, 2995, 2964, 2928, 2875, 1491, 1461, 1384, 1363, 1318, 1261, 991, 979, 958, 913, 802, 753, 689, 623. **HRMS** (ESI) $[M+H]^+$ calcd. for $[C_{16}H_{29}N_2OS]^+$ = 297.1996, 297.2004 found.

3-(1-((2,2,6,6-tetramethylpiperidin-1-yl)oxy)ethyl)pyridine (8)

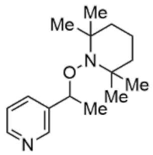

The title product was prepared according to **GP2** using 3-ethylpyridine (107.2 mg, 1.0 mmol), TEMPO (390.8 mg, 2.5 mmol, 2.5 equiv), 18-crown-6 (528.6 mg, 2.0 mmol, 2.0 equiv), KHMDS (1 M solution in THF, 2.0 mL, 2.0 mmol, 2.0 equiv), and anhydrous THF (0.5 mL) for 2 h. The product was purified *via* silica flash column chromatography with eluent conditions 20% EtOAc in hexanes. The fractions containing product were combined and concentrated *in vacuo*. TEMPO persisted and the combined concentrated fractions were filtered through silica with 5% EtOAC in hexanes (200 mL, discarded) followed by a flush with EtOAc (collected, concentrated *in vacuo*) to afford the product as a white solid (125.5 mg, 0.48 mmol, 48% yield). **MP:** 34-35 °C. **^{1}H NMR** (400 MHz, $CDCl_3$) δ 8.53 (s, 1H), 8.46 (d, *J* = 3.7 Hz, 1H), 7.62 (dt, *J* = 7.8, 2.0 Hz, 1H), 7.25 – 7.18 (m, 1H), 4.80 (q, *J* = 6.7 Hz, 1H), 1.47 (d, *J* = 6.7 Hz, 6H), 1.40 – 0.86 (m, 12H), 0.57 (s, 3H). **^{13}C NMR** (101 MHz, $CDCl_3$) δ 148.6, 148.5, 140.7, 134.2, 123.3, 80.8, 59.9, 59.7, 40.3, 34.5, 23.1, 20.4, 17.2. **IR** (neat, cm^{-1}) 2998, 2971, 2936, 2923, 2875, 1574, 1470, 1443, 1360, 1311, 972, 934, 881, 814, 719, 710. **HRMS** (ESI) $[M+H]^+$ calcd. for $[C_{16}H_{27}N_2O]^+$ = 263.2118, 263.2161 found. The spectroscopic data is consistent with a previous report.[3]

2,2,6,6-tetramethyl-1-(1-phenylethoxy)piperidine (9)

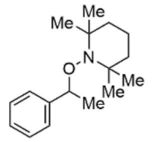

The title product was prepared according to **GP2** using ethylbenzene (**3**) (106.1 mg, 1.0 mmol), TEMPO (390.8 mg, 2.5 mmol, 2.5 equiv), 18-crown-6 (528.6 mg, 2.0 mmol, 2.0 equiv), KHMDS (1 M solution in THF, 2.0 mL, 2.0 mmol, 2.0 equiv), and anhydrous THF (0.5 mL). The crude residue was filtered through silica with 5% EtOAc in hexanes to afford the product as a white solid (211.0 mg, 0.81 mmol, 81% yield). **MP:** 40-41 °C. **^{1}H NMR** (400 MHz, $CDCl_3$) δ 7.35 – 7.26 (m, 4H), 7.26 – 7.16 (m, 1H), 4.78 (q, *J* = 6.7 Hz, 1H), 1.48 (d, *J* = 6.7 Hz, 6H), 1.41 – 0.93 (m, 12H), 0.66 (br s, 3H). **^{13}C NMR** (101 MHz, $CDCl_3$) δ 145.4, 127.6, 126.3, 126.2, 82.7, 59.2, 39.9, 34.0, 33.7, 23.1, 19.9, 16.8. **IR** (neat, cm^{-1}) 2971, 2926, 1493, 1452, 1374, 1361, 1258, 1242,1132, 1060, 935, 882, 759, 697. **HRMS** (ESI) $[M+H]^+$ calcd. for $[C_{17}H_{28}NO]^+$ = 262.2166, 262.2193 found. The spectroscopic data is consistent with a previous report.[4]

4-(((2,2,6,6-tetramethylpiperidin-1-yl)oxy)methyl)benzo[*c*][1,2,5]thiadiazole (10)

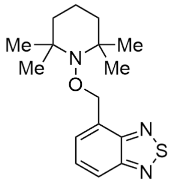

The title product was prepared according to **GP1** using 4-methylbenzo[*c*][1,2,5]thiadiazole (150.2 mg, 1.0 mmol), TEMPO (390.8 mg, 2.5 mmol, 2.5 equiv), NaHMDS (1 M solution in THF, 2.0 mL, 2.0 mmol, 2.0 equiv), and anhydrous THF (0.5 mL). The product was purified *via* silica flash column chromatography with eluent conditions 2% EtOAc → 5% EtOAc in hexanes to afford the product as a white solid (228.9 mg, 0.75 mmol, 75% yield). **MP:** 74-75 °C. **^{1}H NMR** (401 MHz, $CDCl_3$) δ 7.91 (dd, *J* = 8.9, 1.0 Hz, 1H), 7.71 (dq, *J* = 6.8, 1.3 Hz, 1H), 7.60 (dd, *J* = 8.8, 6.8 Hz, 1H), 5.37 (s, 2H), 1.74 – 1.45 (m, 5H), 1.31 (s, 7H), 1.20 (s, 6H). **^{13}C NMR** (101 MHz, $CDCl_3$) δ 155.1, 153.4, 132.0, 129.7, 126.3, 120.1, 74.6, 60.2, 39.9, 33.1, 20.5, 17.3. **IR** (neat, cm^{-1}) 3009, 2967, 2930, 2890, 2869, 1472, 1361, 1244, 1130, 1030, 839, 816, 757, 712. **HRMS** (ESI) $[M+H]^+$ calcd. for $[C_{16}H_{24}N_3OS]^+$ = 306.1635, 306.1688 found.

5-(((2,2,6,6-tetramethylpiperidin-1-yl)oxy)methyl)benzo[*c*][1,2,5]oxadiazole (**11**)

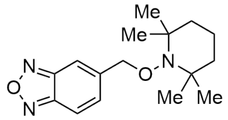

The title product was prepared according to **GP1** using 5-methylbenzo[*c*][1,2,5]oxadiazole (134.1 mg, 1.0 mmol), TEMPO (390.8 mg, 2.5 mmol, 2.5 equiv), LiHMDS (1 M solution in THF, 2.0 mL, 2.0 mmol, 2.0 equiv), and anhydrous THF (0.5 mL). The crude residue was filtered through silica with DCM to afford a crude yellow oil. The product was then purified *via* silica flash column chromatography with eluent conditions 5% EtOAc in hexanes to afford the product as an off-white solid (155.0 mg, 0.54 mmol, 54% yield). **MP:** 38-40 °C. **^{1}H NMR** (400 MHz, $CDCl_3$) δ 7.87 – 7.74 (m, 2H), 7.29 (d, *J* = 9.2 Hz, 1H), 4.92 (s, 2H), 1.77 – 1.31 (m, 6H), 1.21 (d, *J* = 9.7 Hz, 12H). **^{13}C NMR** (101 MHz, $CDCl_3$) δ 149.2, 148.7, 142.3, 131.4, 116.0, 111.9, 77.5, 60.1, 39.6, 32.9, 20.2, 16.9. **IR** (neat, cm^{-1}) 3005, 2981, 2963, 2860, 1537, 1468, 1372, 1349, 1262, 1245, 1133, 1043, 1004, 994, 958, 876, 800, 749, 659. **HRMS** (ESI) $[M+H]^+$ calcd. for $[C_{16}H_{24}N_3O_2]^+$ = 290.1864, 290.1890 found.

1-(benzo[*b*]thiophen-2-ylmethoxy)-2,2,6,6-tetramethylpiperidine (12)

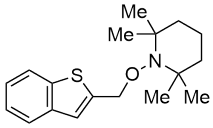

The title product was prepared according to **GP1** using 2-methylbenzo[*b*]thiophene (148.2 mg, 1.0 mmol), TEMPO (390.8 mg, 2.5 mmol, 2.5 equiv), KHMDS (1 M solution in THF, 2.0 mL, 2.0 mmol, 2.0 equiv), and anhydrous THF (0.5 mL). The product was purified *via* silica flash column chromatography with eluent conditions 5% EtOAc in hexanes to afford the product as a white solid (251.5 mg, 0.83 mmol, 83% yield). **MP:** 57-58 °C. **^{1}H NMR** (400 MHz, $CDCl_3$) δ 7.86 – 7.78 (m, 1H), 7.76 – 7.69 (m, 1H), 7.31 (pd, *J* = 7.2, 1.4 Hz, 2H), 7.19 (d, *J* = 1.0 Hz, 1H), 5.04 (d, *J* = 1.0 Hz, 2H), 1.71 – 1.31 (m, 6H), 1.29 (s, 6H), 1.17 (s, 6H). **^{13}C NMR** (101 MHz, $CDCl_3$) δ 141.5, 140.0, 139.5, 124.2, 124.1, 123.4, 122.4, 121.5, 74.5, 60.2, 39.8, 33.2, 20.2, 17.2 **IR** (neat, cm^{-1}) 3011, 2976, 2924, 2867, 1468, 1435, 1374, 1360, 1352, 1131, 1034, 955, 826, 742, 725. **HRMS** (ESI) [M+H]$^+$ calcd. for $[C_{18}H_{26}NOS]^+$ = 304.1730, 304.1789 found.

1-methyl-2-(((2,2,6,6-tetramethylpiperidin-1-yl)oxy)methyl)-1H-indole (13)

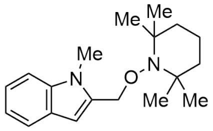

The title product was prepared according to **GP1** using 1,2-dimethyl-1H-indole (145.2 mg, 1.0 mmol), TEMPO (390.8 mg, 2.5 mmol, 2.5 equiv), KHMDS (1 M solution in THF, 2.0 mL, 2.0 mmol, 2.0 equiv), and anhydrous THF (0.5 mL) at 50°C. The product was purified *via* silica flash column chromatography with eluent conditions 5% EtOAc → 10% EtOAc in hexanes followed by filtering the resulting residue through silica with 2.5% EtOAc in hexanes to afford the product as a white solid (258.0 mg, 0.86 mmol, 86% yield). **MP:** 98-99 °C. **^{1}H NMR** (401 MHz, $CDCl_3$) δ 7.58 (dt, *J* = 7.9, 1.0 Hz, 1H), 7.35 – 7.28 (m, 1H), 7.21 (ddd, *J* = 8.3, 7.0, 1.2 Hz, 1H), 7.08 (ddd, *J* = 8.0, 7.0, 1.0 Hz, 1H), 6.46 (d, *J* = 0.8 Hz, 1H), 4.93 (s, 2H), 3.81 (s, 3H), 1.62 – 1.46 (m, 5H), 1.29 (s, 7H), 1.09 (s, 6H). **^{13}C NMR** (101 MHz, $CDCl_3$) δ 137.9, 136.8, 127.5, 121.6, 120.8, 119.4, 109.2, 101.9, 71.5, 60.1, 39.9, 33.4, 30.4, 20.2, 17.2. **IR** (neat, cm^{-1}) 2973, 2914, 2867, 2845, 1470, 1447, 1401, 1372, 1358, 1332, 1239, 1146, 1016, 987, 910, 799, 737, 685. **HRMS** (ESI) [M+H]$^+$ calcd. for $[C_{19}H_{29}N_2O]^+$ = 301.2275, 301.2286 found.

1-(dibenzo[*b,d*]thiophen-4-ylmethoxy)-2,2,6,6-tetramethylpiperidine (14)

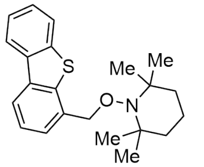

The title product was prepared according to **GP1** using 4-methyldibenzo[*b,d*]thiophene (198.3 mg, 1.0 mmol), TEMPO (390.8 mg, 2.5 mmol, 2.5 equiv), KHMDS (1 M solution in THF, 2.0 mL, 2.0 mmol, 2.0 equiv), and anhydrous THF (0.5 mL) at 50°C. The product was purified *via* silica flash column chromatography with eluent conditions 5% EtOAc in hexanes to afford the product as a white solid (333.3 mg, 0.94 mmol, 94% yield). **MP:** 99-101 °C. **^{1}H NMR** (401 MHz, $CDCl_3$) δ 8.20 – 8.11 (m, 1H), 8.09 (dd, *J* = 7.8, 1.3 Hz, 1H), 7.92 – 7.83 (m, 1H), 7.53 (d, *J* = 6.7 Hz, 1H), 7.50 – 7.40 (m, 3H), 5.10 (s, 2H), 1.72 – 1.44 (m, 5H), 1.33 (s, 7H), 1.20 (s, 6H). **^{13}C NMR** (101 MHz, $CDCl_3$) δ 139.6, 138.2, 135.9, 135.8, 132.9, 126.7, 125.6, 124.6, 124.4, 122.8, 121.7, 120.7, 77.9, 60.3, 39.90, 33.3, 20.5, 17.3. **IR** (neat, cm^{-1}) 2972, 2927, 2868, 1468, 1443, 1401, 1373, 1359, 1244, 1132, 1036, 746. **HRMS** (ESI) [M+H]$^+$ calcd. for $[C_{22}H_{28}NOS]^+$ = 354.1887, 354.1933 found.

8-(pyridin-2-yl)-2-(((2,2,6,6-tetramethylpiperidin-1-yl)oxy)methyl)benzofuro[2,3-*b*]pyridine (15)

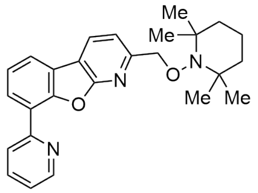

The title product was prepared according to **GP1** using 2-methyl-8-(pyridin-2-yl)benzofuro[2,3-*b*]pyridine (260.3 mg, 1.0 mmol), TEMPO (390.8 mg, 2.5 mmol, 2.5 equiv), LiHMDS (1 M solution in THF, 2.0 mL, 2.0 mmol, 2.0 equiv), and anhydrous THF (0.5 mL). The product was purified *via* silica flash column chromatography with eluent conditions 10% EtOAc in hexanes → 50% EtOAc in hexanes. Fractions which contained residual TEMPO were combined, concentrated *in vacuo* and filtered through silica with 10% EtOAc in hexanes until TEMPO (visibly orange band) eluted. The silica was then flushed with EtOAc, collected and concentrated *in vacuo* to afford the product as an off-white solid (362.8 mg, 0.87 mmol, 87% yield). **MP:** 168-170 °C. **^{1}H NMR** (401 MHz, $CDCl_3$) δ 8.78 (ddd, *J* = 4.8, 1.9, 0.9 Hz, 1H), 8.54 (dt, *J* = 8.0, 1.1 Hz, 1H), 8.40 (dd, *J* = 7.8, 1.3 Hz, 1H), 8.32 (d, *J* = 7.8 Hz, 1H), 7.99 (dd, *J* = 7.6, 1.3 Hz, 1H), 7.85 (td, *J* = 7.7, 1.9 Hz, 1H), 7.64 (d, *J* = 7.8 Hz, 1H), 7.53 (t, *J* = 7.7 Hz, 1H), 7.30 (ddd, *J* = 7.5, 4.8, 1.1 Hz, 1H), 5.12 (s, 2H), 1.73 – 1.32 (m, 6H), 1.27 (s, 6H), 1.22 (s, 6H). **^{13}C NMR** (101 MHz, $CDCl_3$) δ 162.2, 156.0, 152.5, 151.6, 149.3, 136.3, 129.7, 127.6, 124.2, 124.0, 123.4, 123.0, 122.3, 121.2, 116.6, 114.8, 79.4, 59.7, 39.3, 32.6, 20.0, 16.7. **IR** (neat, cm^{-1}) 3027, 3002 2971, 2927, 1501, 1468, 1358, 1262, 1132, 1025, 990, 955, 860, 799. **HRMS** (ESI) $[M+H]^+$ calcd. for $[C_{26}H_{30}N_3O_2]^+$ = 416.2333, 416.2348 found.

4'-(((2,2,6,6-tetramethylpiperidin-1-yl)oxy)methyl)-[1,1'-biphenyl]-2-carbonitrile (16)

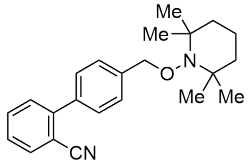

The title product was prepared according to **GP1** using 4'-methyl-[1,1'-biphenyl]-2-carbonitrile (193.2 mg, 1.0 mmol), TEMPO (390.8 mg, 2.5 mmol, 2.5 equiv), KHMDS (1 M solution in THF, 2.0 mL, 2.0 mmol, 2.0 equiv), and anhydrous THF (0.5 mL). The product was purified *via* silica flash column chromatography with eluent conditions 5% EtOAc in hexanes. The fractions were combined and concentrated *in vacuo*. TEMPO persisted and the impure residue was precipitated in MeOH to give a white solid. The solid was filtered over celite and rinsed with H_2O. The filtrand was flushed through the celite with DCM, collected, and dried over Na_2SO_4. The resulting mixture was filtered and concentrated *in vacuo* to afford the product as a white solid (174.0 mg, 0.5 mmol, 50% yield). **MP:** 92-93 °C. **^{1}H NMR** (400 MHz, $CDCl_3$) δ 7.77 (dd, *J* = 7.8, 1.4 Hz, 1H), 7.64 (td, *J* = 7.7, 1.4 Hz, 1H), 7.59 – 7.47 (m, 5H), 7.43 (td, *J* = 7.6, 1.3 Hz, 1H), 4.91 (s, 2H), 1.70 – 1.32 (m, 3H), 1.28 (s, 7H), 1.19 (s, 6H). **^{13}C NMR** (101 MHz, $CDCl_3$) δ 145.5, 139.1, 137.1, 133.9, 132.9, 130.1, 128.7, 127.7, 127.5, 118.9, 111.3, 78.3, 60.2, 39.8, 33.2, 20.5, 17.2. **IR** (neat, cm^{-1}) 3004, 2974, 2930, 2844, 2224, 1478, 2224, 1478, 1442, 1373, 1358, 1131, 1059, 819, 772. **HRMS** (ESI) $[M+H]^+$ calcd. for $[C_{23}H_{29}N_2O]^+$ = 349.2275, 349.2282 found.

2,2,6,6-tetramethyl-1-((4-((trifluoromethyl)thio)benzyl)oxy)piperidine (17)

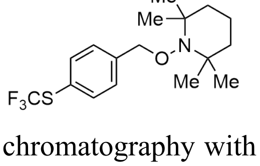

The title product was prepared according to **GP1** using *p*-tolyl(trifluoromethyl)sulfane (192.2 mg, 1.0 mmol), TEMPO (390.8 mg, 2.5 mmol, 2.5 equiv), KHMDS (1 M solution in THF, 2.0 mL, 2.0 mmol, 2.0 equiv), and anhydrous THF (0.5 mL). The product was purified *via* silica flash column chromatography with eluent conditions 5% EtOAc in hexanes to afford the product as an off-white solid (252.4 mg, 0.73 mmol, 73% yield). **MP:** 32-33 °C. **^{1}H NMR** (401 MHz, $CDCl_3$) δ 7.63 (d, *J* = 8.2 Hz, 2H), 7.42 (d, *J* = 8.0 Hz, 2H), 4.88 (s, 2H), 1.77 – 1.31 (m, 6H), 1.24 (s, 6H), 1.18 (s, 6H). **^{13}C NMR** (101 MHz, $CDCl_3$) δ 141.7, 136.4, 129.7 (q, *J* = 307.9 Hz), 128.1, 122.8 (q, *J* = 2.0 Hz), 77.9, 60.2, 39.8, 33.1,

20.4, 17.2. **^{19}F NMR** (377 MHz, $CDCl_3$) δ -42.91 (s, 3F). **IR** (neat, cm^{-1}) 3004, 2936, 2862, 1497, 1456, 1374, 1208, 1130, 809. **HRMS** (ESI) $[M+H]^+$ calcd. for $[C_{17}H_{25}F_3NOS]^+$ = 348.1604, 348.1636 found.

N,N-diethyl-2-(pyridin-2-yl)-2-((2,2,6,6-tetramethylpiperidin-1-yl)oxy)ethan-1-amine (18)

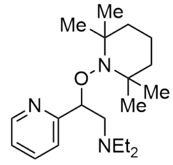

The title product was prepared according to **GP1** using *N,N*-diethyl-2-(pyridin-2-yl)ethan-1-amine (178.3 mg, 1.0 mmol), TEMPO (390.8 mg, 2.5 mmol, 2.5 equiv), KHMDS (1 M solution in THF, 2.0 mL, 2.0 mmol, 2.0 equiv), and anhydrous THF (0.5 mL). The crude residue was filtered through silica with 5% MeOH in DCM (150 mL, discarded) followed by a flush with 5% MeOH, 2% triethylamine in DCM to afford the product as a waxy semi-solid (161.1 mg, 0.48 mmol, 48% yield). **^{1}H NMR** (401 MHz, $CDCl_3$) δ 8.6 (d, *J* = 4.0 Hz, 1H), 7.6 (t, *J* = 7.1 Hz, 1H), 7.3 (d, *J* = 7.9 Hz, 1H), 7.1 (t, *J* = 6.2 Hz, 1H), 4.8 (dd, *J* = 9.2, 5.2 Hz, 1H), 3.0 (qd, *J* = 13.0, 7.1 Hz, 2H), 2.4 (dp, *J* = 22.7, 6.6 Hz, 4H), 1.7 – 1.1 (m, 12H), 1.0 (s, 3H), 0.8 (t, *J* = 7.1 Hz, 6H), 0.3 (s, 3H). **^{13}C NMR** (101 MHz, $CDCl_3$) δ 162.7, 148.6, 135.3, 124.3, 122.1, 87.1, 60.5, 59.0, 56.6, 47.7, 40.5, 33.8, 33.6, 20.3, 20.2, 17.2, 11.8. **IR** (neat, cm^{-1}) 3004, 2968, 2930, 2870, 2800, 1591, 1471, 1436, 1374, 1360, 1133, 989, 745. **HRMS** (ESI) $[M+H]^+$ calcd. for $[C_{20}H_{36}N_3O]^+$ = 334.2853, 334.2879 found.

3-(pyridin-4-yl)-3-((2,2,6,6-tetramethylpiperidin-1-yl)oxy)propan-1-amine (19)

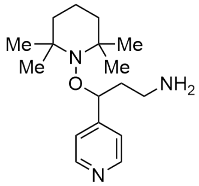

The title product was prepared according to **GP1** using 3-(pyridin-4-yl)propan-1-amine (136.2 mg, 1.0 mmol), TEMPO (390.8 mg, 2.5 mmol, 2.5 equiv), KHMDS (1 M solution in THF, 2.0 mL, 2.0 mmol, 2.0 equiv), and anhydrous THF (0.5 mL) at 50 °C. The product was purified by filtering the crude residue through silica with 10% MeOH in DCM until TEMPO eluted and flushing the silica with 20% triethylamine in MeOH to afford the product as a bronze oil (205.5 mg, 0.71 mmol, 71% yield). **^{1}H NMR** (401 MHz, $CDCl_3$) δ 8.52 (d, *J* = 5.2 Hz, 2H), 7.24 – 7.16 (m, 2H), 4.69 (dd, *J* = 8.9, 4.1 Hz, 1H), 3.86 – 3.17 (br s, 2H), 2.49 (dddd, *J* = 21.6, 18.5, 11.0, 5.8 Hz, 2H), 2.19 (dddd, *J* = 13.6, 10.1, 6.4, 4.1 Hz, 1H), 2.08 – 1.90 (m, 1H), 1.62 – 0.84 (m, 15H), 0.54 (s, 3H). **^{13}C NMR** (101 MHz, $CDCl_3$) δ 151.9, 149.7, 122.6, 84.4, 60.0, 59.8, 40.5, 38.3, 37.5, 34.5, 34.2, 20.4, 17.1. **IR** (neat, cm^{-1}) 2970, 2929, 2870, 1599, 1560, 1464, 1411, 1375, 1361, 1133, 957, 819. **HRMS** (ESI) $[M+H]^+$ calcd. for $[C_{17}H_{30}N_3O]^+$ = 292.2384, 292.2386 found.

3-((2,2,6,6-tetramethylpiperidin-1-yl)oxy)-3-(3-(trifluoromethyl)phenyl)propan-1-amine (20)

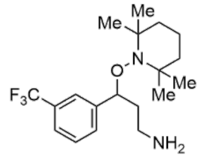

The title product was prepared according to **GP2** using 3-(3-(trifluoromethyl)phenyl)propan-1-amine (203.2 mg, 1.0 mmol), TEMPO (390.8 mg, 2.5 mmol, 2.5 equiv), 18-crown-6 (528.6 mg, 2.0 mmol, 2.0 equiv), KHMDS (1 M solution in THF, 2.0 mL, 2.0 mmol, 2.0 equiv), and anhydrous THF (0.5 mL). The product was purified *via* silica flash column chromatography with eluent conditions 10% MeOH in DCM until TEMPO (visibly orange band) eluted then 2% triethylamine, 10% MeOH in DCM to afford the product as an orange oil (187.2 mg, 0.52 mmol, 52% yield). **^{1}H NMR** (401 MHz, $CDCl_3$) δ 7.62 – 7.34 (m, 4H), 4.75 (dd, *J* = 9.4, 4.3 Hz, 1H), 2.49 (br s, 2H), 2.27 (dtt, *J* = 13.1, 8.8, 4.3 Hz, 1H), 2.03 – 1.90 (m, 1H), 1.67 – 0.75 (m, 17H), 0.45 (s, 3H). **^{13}C NMR** (101 MHz, $CDCl_3$) δ 144.5, 131.1, 130.4 (q, *J* = 32.0 Hz), 128.5, 124.6 (q, *J* = 3.8 Hz), 124.3 (q, *J* = 272.4 Hz), 124.1 (q, *J* = 3.9 Hz), 85.0, 60.3, 59.5, 40.5, 39.8, 38.5, 34.5, 34.2, 20.4, 17.2. **^{19}F NMR** (377 MHz, $CDCl_3$) δ -62.5. **IR** (neat, cm^{-1}) 3005, 2972, 2932, 2871,

1450, 1327, 1163, 1123, 1072, 803, 703, 657. **HRMS** (ESI) $[M+H]^+$ calcd. for $[C_{19}H_{30}F_3N_2O]^+$ = 359.2310, 359.2353.

2,2,6,6-tetramethyl-1-((4-(4,4,5,5-tetramethyl-1,3,2-dioxaborolan-2-yl)benzyl)oxy)piperidine (21)

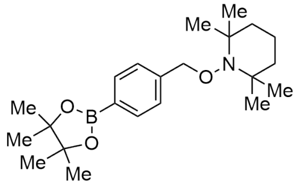

The title product was prepared according to **GP2** using 4,4,5,5-tetramethyl-2-(p-tolyl)-1,3,2-dioxaborolane (218.1 mg, 1.0 mmol), TEMPO (390.8 mg, 2.5 mmol, 2.5 equiv), 18-crown-6 (528.6 mg, 2.0 mmol, 2.0 equiv), KHMDS (1 M solution in THF, 2.0 mL, 2.0 mmol, 2.0 equiv), and anhydrous THF (0.5 mL). ^{1}H NMR spectroscopy of the crude reaction mixture was used to evaluate the crude yield of **21** (238 µmol, 24% yield). The crude residue was loaded onto a preparatory TLC plate and developed with 5% EtOAc in hexanes to afford a mixture of **21** and 4,4,5,5-tetramethyl-2-(p-tolyl)-1,3,2-dioxaborolane starting material. Using ^{1}H NMR spectroscopy, ^{1}H NMR (401 MHz, $CDCl_3$) δ 7.80 (d, *J* = 7.5 Hz, 2H), 7.37 (d, *J* = 7.6 Hz, 2H), 4.86 (s, 2H), 1.79 – 1.42 (m, 6H) 1.25 (s, 6H), 1.16 (s, 6H) signals (belonging only to **21** product) were identified which is consistent with a previous report.[5]

3-bromo-4-(((2,2,6,6-tetramethylpiperidin-1-yl)oxy)methyl)pyridine (22)

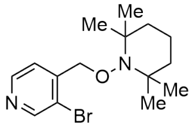

The title product was prepared according to **GP1** using 3-bromo-4-methylpyridine (172.0 mg, 1.0 mmol), TEMPO (390.8 mg, 2.5 mmol, 2.5 equiv), LiHMDS (1 M solution in THF, 2.0 mL, 2.0 mmol, 2.0 equiv), and anhydrous THF (0.5 mL). The crude residue was loaded onto a preparatory TLC plate and developed with 30% DCM in Hexanes to afford the product as a white solid (128.4 mg, 0.39 mmol, 39% yield). We note the lower yield compared to the crude yield reported in Table S1 is due to poor separation between TEMPO / TEMPOH and **22** on silica chromatography which required isolation *via* preparatory TLC in which full resolution was not observed. **MP:** 89-92 °C. **^{1}H NMR** (400 MHz, $CDCl_3$) δ 8.66 (s, 1H), 8.54 (d, *J* = 4.9 Hz, 1H), 7.56 (d, *J* = 4.8 Hz, 1H), 4.90 (s, 2H), 1.85 – 1.31 (m, 6H), 1.22 (s, 12H). **^{13}C NMR** (101 MHz, $CDCl_3$) δ 150.4, 147.5, 146.4, 121.8, 118.7, 75.9, 59.4, 38.9, 32.1, 19.7, 16.3. **IR** (neat, cm^{-1}) 2973, 2943, 2915, 2871, 1445, 1401, 1375, 1361, 1282, 1086, 1012, 832, 722. **HRMS** (ESI) $[M+H]^+$ calcd. for $[C_{15}H_{24}BrN_2O]^+$ = 327.1067, 327.1085 found.

(3*R*)-3-amino-1-(8-((2,2,6,6-tetramethylpiperidin-1-yl)oxy)-3-(trifluoromethyl)-5,6-dihydro-[1,2,4]triazolo[4,3-*a*]pyrazin-7(8*H*)-yl)-4-(2,4,5-trifluorophenyl)butan-1-one (23)

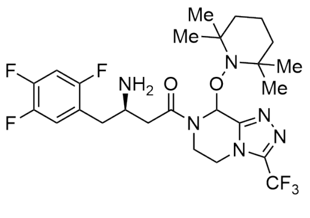

The title product was prepared according to **GP1** using (*R*)-3-amino-1-(3-(trifluoromethyl)-5,6-dihydro-[1,2,4]triazolo[4,3-*a*]pyrazin-7(8*H*)-yl)-4-(2,4,5-trifluorophenyl)butan-1-one (407.3 mg, 1.0 mmol), TEMPO (390.8 mg, 2.5 mmol, 2.5 equiv), LiHMDS (1 M solution in THF, 2.0 mL, 2.0 mmol, 2.0 equiv), and anhydrous THF (0.5 mL). The crude residue was filtered through silica with 1% MeOH in DCM (300 mL) (discarded) then flushed with 10 % MeOH in DCM (200 mL) (collected, concentrated *in vacuo*) to afford the product as a white solid (402.8 mg, 0.72 mmol, 72% yield, dr = 1.3:1). **Diastereomer 1:** ^{1}H NMR (401 MHz, $CDCl_3$) δ 7.1 (q, *J* = 8.7 Hz, 1H), 6.9 (td, *J* = 9.5, 6.5 Hz, 1H), 6.7 (s, 1H), 4.9 (dd, *J* = 14.1, 4.1 Hz, 1H), 4.2 (dd, *J* = 12.4, 3.7 Hz, 1H), 4.1 – 3.9 (m, 1H), 3.6 (s, 1H), 3.5 (t, *J* = 13.0 Hz, 1H), 3.1 (dd, *J* = 16.3, 8.6 Hz, 1H), 2.8 (s, 2H), 2.7 (d, *J* = 16.3 Hz, 1H), 2.1 (s, 2H), 1.8 – 1.1 (m, 12H), 1.1 (s, 3H), 1.0 (s, 3H). **Diastereomer 2:** **^{1}H NMR** (401

MHz, $CDCl_3$) δ 7.1 (td, *J* = 9.2, 6.7 Hz, 1H), 7.0 (td, *J* = 9.5, 6.5 Hz, 1H), 6.7 (s, 1H), 4.9 (dd, *J* = 13.8, 4.2 Hz, 1H), 4.2 (dd, *J* = 12.9, 3.9 Hz, 1H), 4.0 (td, *J* = 12.5, 4.4 Hz, 1H), 3.7 (br s, 1H), 3.5 – 3.4 (m, 1H), 3.2 (d, *J* = 16.2 Hz, 1H), 2.8 (d, *J* = 6.5 Hz, 2H), 2.5 (dd, *J* = 16.5, 9.3 Hz, 1H), 2.2 (br s, 2H), 1.6 – 1.1 (m, 12H), 1.0 (s, 3H), 0.9 (s, 3H). **^{13}C NMR** (mixture of diastereomers) (isolated dr: 3.3 : 1) (101 MHz, CD_3CN) δ 171.1, 170.8, 157.0 (d, *J* = 6.7 Hz), 154.6 (d, *J* = 8.4 Hz), 150.6, 149.2 (d, *J* = 12.4 Hz), 147.2 (d, *J* = 9.4 Hz), 146.8 (d, *J* = 12.4 Hz), 145.4 – 144.3 (m), 142.6 (q, *J* = 39.2 Hz), 122.9 – 121.7 (m), 120.0 – 118.5 (m), 106.6 – 104.99 (m), 81.1, 61.5, 61.4, 59.1, 58.9, 48.3, 48.0, 43.9, 43.7, 40.3, 39.8, 39.7, 39.6, 39.5, 39.4, 35.2, 33.4, 33.3, 32.1, 32.0, 31.7, 31.6, 19.5, 19.3, 19.2, 16.4, 16.3. **^{19}F NMR** (376 MHz, $CDCl_3$) δ -63.2, -63.2, -118.5 to -118.7 (m), -118.7 to -118.8 (m), -135.4 to -135.8 (m), -142.4 (dddd, *J* = 21.6, 16.2, 10.4, 6.5 Hz), -142.6 to -142.8 (m). **IR** (neat, cm^{-1}) 2978, 2939, 1665, 1519, 1422, 1334, 1271, 1186, 1148, 1017, 952, 910, 881, 756. **HRMS** (ESI) $[M+H]^+$ calcd. for $[C_{25}H_{33}F_6N_6O_2]^+$ = 563.2564, 563.2572 found.

N-((*R*)-1-(naphthalen-1-yl)ethyl)-3-((2,2,6,6-tetramethylpiperidin-1-yl)oxy)-3-(3-(trifluoromethyl)phenyl)propan-1-amine (24)

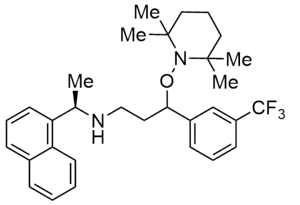

The title product was prepared according to **GP2** using (*R*)-*N*-(1-(naphthalen-1-yl)ethyl)-3-(3-(trifluoromethyl)phenyl)propan-1-amine (357.4 mg, 1.0 mmol), TEMPO (390.8 mg, 2.5 mmol, 2.5 equiv), 18-crown-6 (528.6 mg, 2.0 mmol, 2.0 equiv), KHMDS (1 M solution in THF, 2.0 mL, 2.0 mmol, 2.0 equiv), and anhydrous THF (0.5 mL) at room temperature for 2 h. The product was purified *via* silica flash column chromatography with eluent conditions 50% in hexanes → 100% EtOAc. Fractions containing impurities were combined, concentrated *in vacuo*, and filtered through silica with 25% EtOAc in hexanes (discarded) then flushed with EtOAc to afford the product as a brown oil (356.0 mg, 0.69 mmol, 69% yield, mixture of diastereomers, dr = 1.1 : 1 estimated from ^{19}F NMR spectroscopy). **^{1}H NMR** (mixture of diastereomers) (dr = 1.2 : 1, from isolated ^{19}F NMR spectrum) (401 MHz, $CDCl_3$) δ 8.11 (t, *J* = 7.4 Hz, 1H), 7.89 – 7.80 (m, 1H), 7.71 (d, *J* = 8.3 Hz, 1H), 7.58 – 7.38 (m, 6H), 7.39 – 7.29 (m, 1H), 4.76 (ddd, *J* = 13.6, 9.6, 3.7 Hz, 1H), 4.49 (p, *J* = 6.5 Hz, 1H), 2.57 – 2.22 (m, 3H), 2.10 – 1.83 (m, 1H), 1.75 – 1.03 (m, 17H), 0.96 (s, 3H), 0.46 (s, 3H). **^{13}C NMR** (mixture of diastereomers) (101 MHz, $CDCl_3$) δ 142.8, 142.7, 139.6, 139.4, 132.4, 132.3, 129.6, 129.6, 129.6, 129.4, 128.6 (q, *J* = 31.9 Hz), 127.3, 126.7, 126.7, 125.5, 125.5, 124.1, 124.1, 124.0, 123.7, 123.6, 123.0 (q, *J* = 3.5 Hz), 122.8 (q, *J* = 3.7 Hz), 122.3 (dq, *J* = 7.6, 3.6 Hz), 121.3, 121.1, 121.0, 83.6, 83.5, 58.4, 57.8, 52.2, 52.0, 42.5, 42.2, 38.8, 34.9, 34.8, 32.8, 32.5, 22.2, 21.7, 18.7, 15.5, 15.5. **^{19}F NMR** (mixture of diastereomers) (376 MHz, $CDCl_3$) δ -62.51, -62.53 (dr = 1 : 1.15). **IR** (neat, cm^{-1}) 2970, 2931, 1449, 1327, 1163, 1123, 1072, 973, 799, 777, 703. **HRMS** (ESI) $[M+H]^+$ calcd. for $[C_{31}H_{40}F_3N_2]^+$ = 513.3088, 513.3088 found.

(4-methoxyphenyl)(5-nitro-2-(1-((2,2,6,6-tetramethylpiperidin-1-yl)oxy)butyl)benzofuran-3-yl)methanone (25)

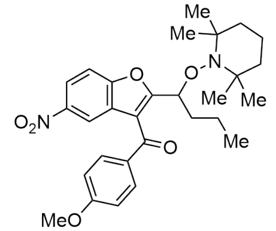

The title product was prepared according to **GP1** using (2-butyl-5-nitrobenzofuran-3-yl)(4-methoxyphenyl)methanone (353.4 mg, 1.0 mmol), TEMPO (390.8 mg, 2.5 mmol, 2.5 equiv), KHMDS (1 M solution in THF, 2.0 mL, 2.0 mmol, 2.0 equiv), and anhydrous THF (0.5 mL). The product was purified *via* silica flash column chromatography with eluent conditions 5% EtOAc / hexanes on neutralized silica (the silica gel was neutralized by preparing a silica slurry with 2% triethylamine /

hexanes solution). Fractions that coeluted with TEMPO were combined, concentrated *in vacuo* and filtered through silica with DCM (collected, concentrated *in vacuo*) to afford the product as a white solid (233.3 mg, 0.46 mmol, 46% yield). **MP:** 49-50 °C. **^{1}H NMR** (400 MHz, $CDCl_3$) δ 8.31 (d, *J* = 2.3 Hz, 1H), 8.26 (dd, *J* = 9.0, 2.4 Hz, 1H), 7.95 – 7.86 (m, 2H), 7.65 (d, *J* = 9.0 Hz, 1H), 7.04 – 6.96 (m, 2H), 5.19 (dd, *J* = 8.6, 6.2 Hz, 1H), 3.92 (s, 3H), 2.14 – 1.96 (m, 2H), 1.54 – 1.35 (m, 2H), 1.35 – 1.14 (m, 6H), 1.11 (s, 3H), 1.06 – 0.90 (m, 9H), 0.44 (s, 3H). **^{13}C NMR** (101 MHz, $CDCl_3$) δ 188.5, 164.3, 164.06, 156.2, 144.6, 132.1, 131.0, 127.6, 121.5, 120.8, 118.3, 114.0, 112.0, 76.7, 60.8, 59.2, 55.6, 40.2, 40.1, 34.4, 33.2, 31.9, 20.4, 20.3, 19.0, 17.0, 14.2. **IR** (neat, cm^{-1}) 3009, 2972, 2929, 2872, 1648, 1597, 1575, 145, 1343, 1252, 1167, 1133, 912, 834, 751. **HRMS** (ESI) $[M+H]^+$ calcd. for $[C_{29}H_{37}N_2O_6]^+$ = 509.2647, 509.2667 found.

1-methyl-3-(3-(((2,2,6,6-tetramethylpiperidin-1-yl)oxy)methyl)-4-(4-((trifluoromethyl)thio)phenoxy)phenyl)-1,3,5-triazinane-2,4,6-trione (26)

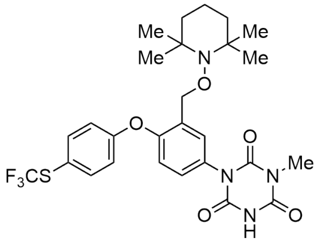

The title product was prepared according to **GP1** using 1-methyl-3-(3-methyl-4-(4-((trifluoromethyl)thio)phenoxy)phenyl)-1,3,5-triazinane-2,4,6-trione (425.4 mg, 1.0 mmol), TEMPO (390.8 mg, 2.5 mmol, 2.5 equiv), KHMDS (1 M solution in THF, 3.0 mL, 3.0 mmol, 3.0 equiv), and anhydrous THF (0.5 mL) at 50 °C. ^{1}H NMR spectroscopy of the crude reaction mixture was used to evaluate the initial yield of **26** (568 μmol, 57% yield). We note that if 2.0 eqiuiv KHMDS is used instead of 3.0 equiv then a 46% yield is observed in the crude reaction mixture (458 μmol, 46% yield). The product was purified *via* preparatory thin layer chromatography, as the product and residual TEMPO exhibit very poor resolution on silica. The crude residue was loaded onto a preparatory TLC plate and developed with 3% MeOH in DCM to afford the product as a white solid (226.3 mg, 0.39 mmol, 39% yield). **MP:** 85-87 °C. **^{1}H NMR** (400 MHz, $CDCl_3$) δ 7.60 (d, *J* = 8.7 Hz, 2H), 7.47 (d, *J* = 2.6 Hz, 1H), 7.18 (dd, *J* = 8.6, 2.6 Hz, 1H), 7.07 – 6.96 (m, 3H), 4.86 (s, 2H), 3.41 (s, 3H), 1.78 – 1.24 (m, 6H), 1.15 (s, 6H), 1.04 (s, 6H). **^{13}C NMR** (101 MHz, $CDCl_3$) δ 159.8, 153.4, 150.0, 149.0, 148.5, 138.5, 131.9, 129.7, 129.46 (q, J = 308.1 Hz), 129.4, 128.7, 120.3, 118.8, 117.9 (q, *J* = 2.4 Hz), 73.1, 60.1, 39.7, 32.9, 29.2, 20.3, 17.1. **^{19}F NMR** (377 MHz, $CDCl_3$) δ -43.52 (s, 3F). **IR** (neat, cm^{-1}) 3221, 3105, 2979, 2937, 1725, 1697, 1584, 1484, 1392, 1235, 1154, 1115, 1084, 907, 728. **HRMS** (ESI) $[M+H]^+$ calcd. for $[C_{27}H_{32}F_3N_4O_5S]^+$ = 581.2041, 581.2047.

(2*S*,3*R*,4*R*,5*S*,6*R*)-2-(4-chloro-3-((4-(((S)-tetrahydrofuran-3-yl)oxy)phenyl)((2,2,6,6-tetramethylpiperidin-1-yl)oxy)methyl)phenyl)-6-(hydroxymethyl)tetrahydro-2*H*-pyran-3,4,5-triol (27)

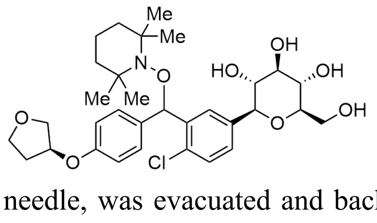

To a 8 mL, oven dried dram vial (vial A) with a stir bar, TEMPO (390.8 mg, 2.5 mmol, 2.5 equiv) and (2*S*,3*R*,4*R*,5*S*,6*R*)-2-(4-chloro-3-(4-(((S)-tetrahydrofuran-3-yl)oxy)benzyl)phenyl)-6-(hydroxymethyl)tetrahydro-2*H*-pyran-3,4,5-triol (450.9 mg, 1.0 mmol, 1.0 equiv) were added. The vial was sealed with a screwcap fitted with a PTFE septum and with a needle, was evacuated and backfilled three times with anhydrous nitrogen gas and left under a positive pressure of nitrogen gas on a manifold Schlenk line. To the vial, DMF (0.5 mL) was added *via* syringe and the solution was stirred. In a separate oven dried 8 mL dram vial (vial B), KHMDS (598.5 mg, 3.0 mmol, 3.0 equiv) was added. Vial B was sealed with a screwcap and PTFE septum and with a needle, was evacuated and backfilled three times with anhydrous nitrogen gas and left under a positive pressure of nitrogen gas on a manifold Schlenk line. DMF (1.75 mL) was added *via* syringe to vial B. The vial B

solution was then transferred to the vial A solution *via* syringe. The reaction mixture was then stirred for 2 h at room temperature. At this time, the vial was unsealed and MeOH (1.0 mL, 24.7 mmol, 24.7 equiv) was added *via* syringe to quench the reaction mixture. The crude reaction mixture was analyzed by ^{1}H NMR spectroscopy according to **GP1**. The crude reaction mixture and the NMR sample were then combined in a 125 mL separatory funnel with H_2O (50 mL) and extracted with DCM (3 x 50 mL). The combined organic layers were then transferred to a 250 mL separatory funnel and washed with LiCl (5% in H_2O, 50 mL). The organic layer was collected, dried over Na_2SO_4, filtered, transferred to a 500 mL round bottom flask, and concentrated *in vacuo*. *n*-Heptane (50 mL) was added to the flask and the solution was reconcentrated. The resulting crude residue was dissolved in DCM (5 mL) and added to hexanes dropwise (100 mL) to afford a white precipitate. This process was repeated twice more to afford the product as a white solid (365.4 mg, 0.60 mmol, 60% yield, dr = 2:1). **Note:** DMF was used for this reaction because (2*S*,3*R*,4*R*,5*S*,6*R*)-2-(4-chloro-3-(4-(((S)-tetrahydrofuran-3-yl)oxy)benzyl)phenyl)-6-(hydroxymethyl)tetrahydro-2*H*-pyran-3,4,5-triol is insoluble in THF. **^{1}H NMR** (mixture of diastereomers) (401 MHz, $CDCl_3$) δ 7.72 (d, *J* = 18.7 Hz, 1H), 7.36 (d, *J* = 8.2 Hz, 2H), 7.23 – 7.04 (m, 2H), 6.72 (d, *J* = 8.2 Hz, 2H), 5.99 (s, 1H), 4.79 (s, 1H), 4.43 – 3.98 (m, 2H), 3.98 – 3.23 (m, 11H), 3.15 – 2.84 (m, 1H), 2.65 (d, *J* = 62.9 Hz, 1H), 2.26 – 1.87 (m, 2H), 1.33 (d, *J* = 57.0 Hz, 6H), 1.09 (s, 6H), 0.90 – 0.52 (m, 6H). **^{13}C NMR** (mixture of diastereomers) (101 MHz, $CDCl_3$) δ 155.7, 142.2, 141.8, 137.4, 137.2, 135.2, 130.5, 128.8, 128.3, 128.1, 127.2, 126.8, 126.1, 125.5, 114.5, 85.7, 80.8, 79.1, 77.8, 76.7, 74.8, 74.3, 72.5, 70.0, 69.7, 69.1, 66.7, 66.7, 61.9, 61.6, 59.6, 59.1, 39.8, 33.8, 32.8, 32.5, 32.3, 20.1, 19.9, 16.7. **IR** (neat, cm^{-1}) 3383 (broad), 2932, 2932, 2871, 1609, 1507, 1472, 1361, 1240, 1175, 1084, 1043, 906, 859, 729, 647. **HRMS** (ESI) $[M+H]^+$ calcd. for $[C_{32}H_{45}ClNO_8]^+$ = 606.2829, 606.2879 found.

1-((2-bromo-5-methylphenyl)(5-(4-fluorophenyl)thiophen-2-yl)methoxy)-2,2,6,6-tetramethylpiperidine (28)

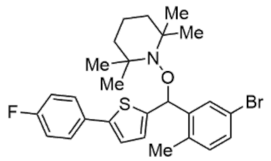

The title product was prepared according to **GP1** using 2-(2-bromo-5-methylbenzyl)-5-(4-fluorophenyl)thiophene (361.3 mg, 1.0 mmol), TEMPO (390.8 mg, 2.5 mmol, 2.5 equiv), KHMDS (1 M solution in THF, 2.0 mL, 2.0 mmol, 2.0 equiv), and anhydrous THF (0.5 mL). ^{1}H NMR spectroscopy of the crude reaction mixture was used to evaluate the yield of **28** (958 µmol, 96% yield, >20:1 selectivity). The product was purified *via* silica flash column chromatography with eluent conditions 2.5% Et_2O in hexanes. Fractions containing the product and impurities were combined and concentrated *in vacuo* and purified *via* flash column chromatography with eluent conditions 25% DCM in hexanes to afford the product as a yellow solid (233.4 mg, 0.45 mmol, 45% yield). **MP:** 54-56 °C. **Note:** this product appears to be unstable on silica, as color change and streaking occurred likely resulting in yield loss. **^{1}H NMR** (400 MHz, $CDCl_3$) δ 7.84 (s, 1H), 7.54 (dd, *J* = 8.7, 5.3 Hz, 2H), 7.32 (dd, *J* = 8.0, 2.2 Hz, 1H), 7.11 – 7.03 (m, 3H), 6.99 (d, *J* = 8.1 Hz, 1H), 6.90 (d, *J* = 3.6 Hz, 1H), 5.98 (s, 1H), 2.28 (s, 3H), 1.74 – 1.29 (m, 6H), 1.25 (s, 3H), 1.17 (s, 3H), 0.99 (s, 3H), 0.81 (s, 3H). **^{19}F NMR** (377 MHz, $CDCl_3$) δ -114.95 (tt, *J* = 8.4, 5.4 Hz). **IR** (neat, cm^{-1}) 2971, 2929, 2869, 1508, 1465, 1361, 1231, 1158, 1132, 832, 799. **HRMS** (ESI) $[M+H]^+$ calcd. for $[C_{27}H_{32}BrFNOS]^+$ = 516.1367, 516.1366 found.

4-((4-methoxyphenyl)((2,2,6,6-tetramethylpiperidin-1-yl)oxy)methyl)-5,6,7,8-tetrahydroquinoline (29)

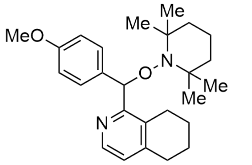

The title product was prepared according to **GP1** using 4-(4-methoxybenzyl)-5,6,7,8-tetrahydroquinoline (253.3 mg, 1.0 mmol), TEMPO (390.8 mg, 2.5 mmol, 2.5 equiv), NaHMDS (1 M solution in THF, 2.0 mL, 2.0 mmol, 2.0 equiv), and anhydrous THF (0.5 mL). Assessment of the crude reaction mixture by ^{1}H NMR spectroscopy indicated a >20:1 ratio for site selectivity. The product was purified *via* silica flash column chromatography with eluent conditions 10% → 15% EtOAc in hexanes. Fractions containing residual TEMPO were combined, concentrated *in vacuo*, and filtered through silica with DCM (200 mL) (discarded) followed by a flush with 2% MeOH in DCM (collected, concentrated *in vacuo*) to afford the product as a hard orange wax (378.7 mg, 0.93 mmol, 93% yield, >20:1 selectivity). **^{1}H NMR** (401 MHz, $CDCl_3$) δ 8.34 (d, *J* = 4.8 Hz, 1H), 7.46 (d, *J* = 8.1 Hz, 2H), 6.89 – 6.82 (m, 3H), 6.07 (s, 1H), 3.79 (s, 3H), 2.99 (d, *J* = 16.3 Hz, 1H), 2.94 – 2.13 (m, 3H), 1.91 – 1.39 (m, 9H), 1.39 – 1.23 (m, 4H), 1.17 (s, 3H), 1.03 (s, 3H), 0.56 (s, 3H). **^{13}C NMR** (101 MHz, $CDCl_3$) δ 157.6, 155.0, 143.4, 142.2, 131.1, 127.7, 124.8, 119.9, 110.1, 57.2, 56.2, 52.3, 37.3, 30.3, 30.1, 26.5, 21.9, 19.8, 19.0, 17.6, 17.5, 14.2. **IR** (neat, cm^{-1}) 3263 (broad), 2973, 2928, 2870, 1574, 1490, 1470, 1448, 1373, 1358, 1132, 1063, 1030, 956, 821, 695, 603. **HRMS** (ESI) $[M+H]^+$ calcd. for $[C_{26}H_{37}N_2O_2]^+$ = 409.2850, 409.2850 found.

5-ethyl-2-(((2,2,6,6-tetramethylpiperidin-1-yl)oxy)methyl)pyridine (30)

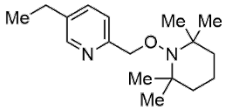

The title product was prepared according to **GP1** using 5-ethyl-2-methylpyridine (121.2 mg, 1.0 mmol), TEMPO (390.8 mg, 2.5 mmol, 2.5 equiv), KHMDS (1 M solution in THF, 2.0 mL, 2.0 mmol, 2.0 equiv), and anhydrous THF (0.5 mL). Assessment of the crude reaction mixture by ^{1}H NMR spectroscopy indicated a >20:1 ratio for site selectivity. The product was purified *via* silica flash column chromatography with eluent conditions 5% → 10% EtOAc in hexanes. Fractions containing residual TEMPO were combined, concentrated *in vacuo*, and filtered through silica with 5% EtOAc in hexanes (discarded) followed by a flush with EtOAc (collected, concentrated *in vacuo*) to afford the product as an orange oil (241.3 mg, 0.87 mmol, 87% yield). **^{1}H NMR** (400 MHz, $CDCl_3$) δ 8.44 (s, 1H), 7.57 (d, *J* = 7.8 Hz, 1H), 7.49 (d, *J* = 7.8 Hz, 1H), 5.01 (s, 2H), 2.69 (q, *J* = 7.6 Hz, 2H), 1.76 – 1.36 (m, 6H), 1.35 – 1.06 (m, 15H). **^{13}C NMR** (101 MHz, $CDCl_3$) δ 154.4, 147.2, 136.2, 134.4, 119.5, 78. 3, 58.6, 38.2, 31.6, 24.5, 18.9, 15.7, 14.1. **IR** (neat, cm^{-1}) 2968, 2929, 2871, 1570, 1490, 1450, 1394, 1358, 1262, 1132, 1067, 1027, 926, 834. **HRMS** (ESI) $[M+H]^+$ calcd. for $[C_{17}H_{29}N_2O]^+$ = 277.2275, 277.2277 found.

5-methyl-2-(((2,2,6,6-tetramethylpiperidin-1-yl)oxy)methyl)pyridine (31)

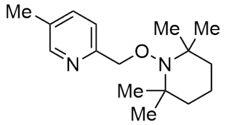

The title product was prepared according to **GP1** using 2,5-dimethylpyridine (107.2 mg, 1.0 mmol), TEMPO (390.8 mg, 2.5 mmol, 2.5 equiv), KHMDS (1 M solution in THF, 2.0 mL, 2.0 mmol, 2.0 equiv), and anhydrous THF (0.5 mL). Assessment of the crude reaction mixture by ^{1}H NMR spectroscopy indicated a >20:1 ratio for site selectivity. The product was purified *via* silica flash column chromatography with eluent conditions 5% → 20% EtOAc in hexanes. Fractions containing residual TEMPO were combined, concentrated *in vacuo*, and filtered through silica with DCM (discarded) followed by a flush with MeOH (collected, concentrated *in vacuo*) to afford the product as an orange oil (159.4 mg, 0.61 mmol, 61% yield). **^{1}H NMR** (401 MHz, $CDCl_3$) δ 8.37 (s, 1H), 7.49 (dd, *J* = 8.0, 2.2 Hz, 1H), 7.40 (d, *J* = 7.9 Hz, 1H), 4.94 (s, 2H), 2.32 (s, 3H),

1.78 – 1.29 (m, 6H), 1.23 (s, 6H), 1.17 (s, 6H). **^{13}C NMR** (101 MHz, $CDCl_3$) δ 149.5, 143.2, 131.1, 125.3, 114.8, 73.6, 53.9, 33.7, 27.0, 14.3, 12.4, 11.1. **IR** (neat, cm^{-1}) 3263 (borad) 2973, 2928, 2870, 1574, 1490, 1469, 1448, 1373, 1358, 1262, 1245, 1132, 1063, 820, 695, 603. **HRMS** (ESI) $[M+H]^+$ calcd. for $[C_{16}H_{27}N_2O]^+$ = 263.2118, 263.2154 found.

3-methyl-4-(1-((2,2,6,6-tetramethylpiperidin-1-yl)oxy)propyl)pyridine (32)

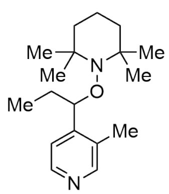

The title product was prepared according to **GP1** using 3-methyl-4-propylpyridine (135.1 mg, 1.0 mmol), TEMPO (390.8 mg, 2.5 mmol, 2.5 equiv), KHMDS (1 M solution in THF, 2.0 mL, 2.0 mmol, 2.0 equiv), and anhydrous THF (0.5 mL). Assessment of the crude reaction mixture by ^{1}H NMR spectroscopy indicated a >20:1 ratio for site selectivity. The crude residue was filtered through silica with 10% EtOAc / hexanes (200 mL) until TEMPO eluted (visibly orange band) (discarded) followed by a flush with 30% EtOAc (collected, concentrated *in vacuo*). The resulting residue was filtered once more through silica with 5% EtOAc / hexanes (discarded) followed by a flush with 100% EtOAc to the product as a white solid (242.9 mg, 0.84 mmol, 84% yield). **^{1}H NMR** (401 MHz, $CDCl_3$) δ 8.42 (d, J = 5.0 Hz, 1H), 8.35 (s, 1H), 7.27 (d, J = 5.2 Hz, 1H), 4.87 (dd, J = 8.2, 3.9 Hz, 1H), 2.30 (s, 3H), 2.04 – 1.84 (m, 2H), 1.63 – 0.88 (m, 15H), 0.69 (t, J = 7.5 Hz, 3H), 0.61 (s, 3H). **MP:** 61-63 °C. **^{13}C NMR** (101 MHz, $CDCl_3$) δ 150.4, 150.2, 146.8, 129.7, 121.7, 83.6, 59.3, 40.0, 33.9, 33.0, 26.7, 19.9, 19.8, 16.7, 16.0, 7.9. **IR** (neat, cm^{-1}) 2999, 2966, 2933, 2873, 1593, 1465, 1445, 1358, 1134, 1055, 1004, 984, 910, 832, 702. **HRMS** (ESI) $[M+H]^+$ calcd. for $[C_{18}H_{31}N_2O]^+$ = 291.2431, 291.2430 found.

1-((2,6-dibromo-3-methylbenzyl)oxy)-2,2,6,6-tetramethylpiperidine (33)

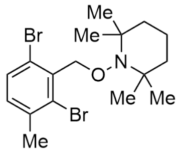

The title product was prepared according to **GP1** using 1,3-dibromo-2,4-dimethylbenzene (264.0 mg, 1.0 mmol), TEMPO (390.8 mg, 2.5 mmol, 2.5 equiv), NaHMDS (1 M solution in THF, 2.0 mL, 2.0 mmol, 2.0 equiv), and anhydrous THF (0.5 mL). Assessment of the crude reaction mixture by ^{1}H NMR spectroscopy indicated an 8:1 ratio for site selectivity. The product was purified *via* silica flash column chromatography with eluent conditions 25% → 50% DCM in hexanes to afford the product as a white solid (150.2 mg, 0.36 mmol, 36% yield). We note that **33** coelutes with its minor positional isomer 1-((2,4-dibromo-3-methylbenzyl)oxy)-2,2,6,6-tetramethylpiperidine. The isolated yield reported above represents the total mass of the isomeric mixture. The amount of 1-((2,4-dibromo-3-methylbenzyl)oxy)-2,2,6,6-tetramethylpiperidine in the mixture was determined by ^{1}H NMR spectroscopy to be 15% molar ratio (22.5 mg). **^{1}H NMR** (400 MHz, $CDCl_3$) δ 7.43 (d, J = 8.1 Hz, 1H), 7.03 (dd, J = 8.2, 0.7 Hz, 1H), 5.17 (s, 2H), 2.40 (s, 3H), 1.76 – 1.39 (m, 6H), 1.33 (s, 6H), 1.08 (s, 6H). **^{13}C NMR** (101 MHz, $CDCl_3$) δ 138.5, 137.4, 131.9, 131.1, 129.1, 123.0, 78.8, 60.1, 40.3, 33.9, 24.4, 20.3, 17.3. **IR** (neat, cm^{-1}) 2974, 2929, 2865, 1442, 1371, 1358, 1188, 1132, 1047, 1021, 990, 804. **HRMS** (ESI) $[M+H]^+$ calcd. for $[C_{17}H_{26}Br_2NO]^+$ = 420.0356, 420.0368 found

2-(3-phenyl-1-((2,2,6,6-tetramethylpiperidin-1-yl)oxy)propyl)pyridine (34)

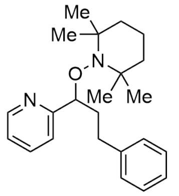

The title product was prepared according to **GP1** using 2-(3-phenylpropyl)pyridine (197.3 mg, 1.0 mmol), TEMPO (390.8 mg, 2.5 mmol, 2.5 equiv), KHMDS (1 M solution in THF, 2.0 mL, 2.0 mmol, 2.0 equiv), and anhydrous THF (0.5 mL). Assessment of the crude reaction mixture by ^{1}H NMR spectroscopy indicated a >20:1 ratio for site selectivity. The product was purified *via* silica flash column chromatography with eluent conditions 100% DCM until TEMPO eluted (1400 mL) then 1% MeOH → 3% MeOH in DCM to afford the product as an off-white solid (325.4 mL, 0.92 mmol, 92% yield). **MP:** 53-45 °C. **^{1}H NMR** (401 MHz, $CDCl_3$) δ 8.60 (d, *J* = 3.3 Hz, 1H), 7.64 (td, *J* = 7.6, 1.8 Hz, 1H), 7.40 (d, *J* = 7.8 Hz, 1H), 7.21 (t, *J* = 7.5 Hz, 2H), 7.17 – 7.04 (m, 4H), 4.82 (dd, *J* = 7.3, 3.3 Hz, 1H), 2.63 – 2.08 (m, 4H), 1.72 – 0.82 (m, 15H), 0.45 (s, 3H). **^{13}C NMR** (101 MHz, $CDCl_3$) δ 162.3, 148.6, 141.7, 135.5, 128.0, 127.9, 125.3, 122.9, 121.8, 87.3, 59.7, 59.1, 40.0, 35.8, 33.8, 33.2, 30.8, 20.0, 16.8. **IR** (neat, cm^{-1}) 3003, 2971, 2989, 2928, 2865, 1589, 1454, 1444, 1358, 1131, 986, 974, 759, 710. **HRMS** (ESI) $[M+H]^+$ calcd. for $[C_{23}H_{33}N_2O]^+$ = 353.2588, 353.2637.

1-((4-cyclohexylbenzyl)oxy)-2,2,6,6-tetramethylpiperidine (35)

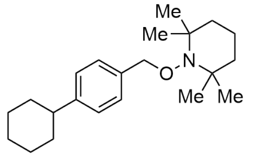

The title product was prepared according to **GP2** 1-cyclohexyl-4-methylbenzene (174.3 mg, 1.0 mmol), TEMPO (390.8 mg, 2.5 mmol, 2.5 equiv), 18-crown-6 (528.6 mg, 2.0 mmol, 2.0 equiv), KHMDS (1 M solution in THF, 2.0 mL, 2.0 mmol, 2.0 equiv), and anhydrous THF (0.5 mL) for 15 h. Assessment of the crude reaction mixture by ^{1}H NMR spectroscopy indicated a >20:1 ratio for site selectivity. The product was purified *via* silica flash column chromatography with eluent conditions 2.5% EtOAc in hexanes to afford the product as a yellow solid (128.4 mg, 0.39 mmol, 39% yield). **MP:** 72-75 °C. **^{1}H NMR** (401 MHz, $CDCl_3$) δ 7.34 (d, *J* = 7.9 Hz, 2H), 7.22 (d, *J* = 8.1 Hz, 2H), 4.84 (s, 2H), 2.53 (ddt, *J* = 11.3, 6.9, 3.5 Hz, 1H), 2.04 – 1.74 (m, 5H), 1.71 – 1.36 (m, 10H), 1.31 (s, 7H), 1.20 (s, 6H). **^{13}C NMR** (101 MHz, $CDCl_3$) δ 147.3, 135.8, 127.7, 126.8, 78.8, 60.1, 44.5, 39.9, 34.6, 33.2, 27.0, 26.3, 20.4, 17.3. **IR** (neat, cm^{-1}) 2979,, 2927, 2853, 1512, 1444, 1376, 1263, 1245, 1051, 812, 705. **HRMS** (ESI) $[M+H]^+$ calcd. for $[C_{22}H_{36}NO]^+$ = 330.2792, 330.2848 found.

2,2,6,6-tetramethyl-1-((3'-methyl-[1,1'-biphenyl]-4-yl)methoxy)piperidine (36)

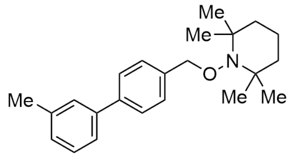

The title product was prepared according to **GP2** using 3,4'-dimethyl-1,1'-biphenyl (182.3 mg, 1.0 mmol), TEMPO (390.8 mg, 2.5 mmol, 2.5 equiv), 18-crown-6 (528.6 mg, 2.0 mmol, 2.0 equiv), KHMDS (1 M solution in THF, 3.0 mL, 3.0 mmol, 3.0 equiv), and anhydrous THF (0.5 mL) for 2 h. Assessment of crude reaction mixture by ^{1}H NMR spectroscopy indicated a 7:1 ratio for site selectivity. The product was purified *via* silica flash column chromatography with eluent conditions 2.5% → 5% EtOAc in hexanes. Fractions containing impurities (other isomer, starting material) were combined, concentrated *in vacuo*, and filtered through silica with hexanes (400 mL) (discarded) followed by a flush with EtOAc (collected, concentrated *in vacuo*) to afford the product as a white solid (208.2 mg, 0.62 mmol, 62% yield). **MP:** 33-35 °C. **^{1}H NMR** (401 MHz, $CDCl_3$) δ 7.61 – 7.52 (m, 2H), 7.45 – 7.36 (m, 4H), 7.32 (t, *J* = 7.7 Hz, 1H), 7.15 (d, *J* = 7.0 Hz, 1H), 4.86 (s, 2H), 2.42 (s, 3H), 1.72 – 1.32 (m, 6H), 1.28 (s, 6H), 1.17 (s, 6H). **^{13}C NMR** (101 MHz, $CDCl_3$) δ 141.2, 140.5, 138.4, 137.3, 128.7, 128.0, 128.0, 127.1, 124.3, 78.6, 60.1, 39.9, 33.2, 21.7 20.5, 17.3. **IR** (neat, cm^{-1}) 2973, 2927, 2868, 1607, 1484, 1470, 1449, 1400,

1358, 1262, 1244, 1132, 1047, 820, 779, 699, 618. **HRMS** (ESI) $[M+H]^+$ calcd. for $[C_{23}H_{32}NO]^+$ = 338.2479, 338.2525 found.

1-((2-bromo-3-ethylbenzyl)oxy)-2,2,6,6-tetramethylpiperidine (1° selective functionalization of 37)

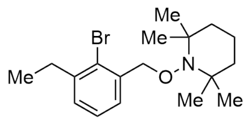

The title product was prepared according to **GP1** using 2-bromo-1-ethyl-3-methylbenzene (199.1 mg, 1.0 mmol), TEMPO (390.8 mg, 2.5 mmol, 2.5 equiv), KHMDS (1 M solution in THF, 2.0 mL, 2.0 mmol, 2.0 equiv), and anhydrous THF (0.5 mL) at 50 °C. ^{1}H NMR spectroscopy of the crude reaction mixture was used to evaluate the yield of 1-((2-bromo-3-ethylbenzyl)oxy)-2,2,6,6-tetramethylpiperidine (693 µmol, 69% yield; >20:1, 1° selective). The crude residue was filtered through silica with DCM (200 mL) and the filtrate was collected and concentrated *in vacuo*. The resulting concentrate which contained residual starting material was filtered through silica with 100% hexanes (200 mL, discarded) followed by a flush with EtOAC (100 mL, collected, concentrated *in vacuo*) to afford the product as a white solid (235.5 mg, 0.66 mmol, 66% yield). **MP:** 57-60 °C. **^{1}H NMR** (400 MHz, $CDCl_3$) δ 7.43 (d, *J* = 7.3 Hz, 1H), 7.26 (t, *J* = 7.6 Hz, 1H), 7.15 (dd, *J* = 7.6, 1.8 Hz, 1H), 4.90 (s, 2H), 2.79 (q, *J* = 7.5 Hz, 2H), 1.82 – 1.29 (m, 6H), 1.29 – 1.12 (m, 15H). **^{13}C NMR** (101 MHz, $CDCl_3$) δ 143.5, 138.7, 128.0, 127.2, 126.0, 123.8, 78.5, 60.2, 39.9, 33.1, 29.8, 20.6, 17.3, 14.4. **IR** (neat, cm^{-1}) 3010, 2971, 2930, 2884, 2842, 1462, 1422, 1353, 1262, 1135, 1071, 1024, 807, 780, 713. **HRMS** (ESI) $[M+H]^+$ calcd. for $[C_{18}H_{29}BrNO]^+$ = 354.1428, 354.1447 found.

1-(1-(2-bromo-3-methylphenyl)ethoxy)-2,2,6,6-tetramethylpiperidine (2° selective functionalization of 37)

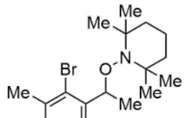

The title product was prepared according to **GP2** using 2-bromo-1-ethyl-3-methylbenzene (199.1 mg, 1.0 mmol), TEMPO (390.8 mg, 2.5 mmol, 2.5 equiv), 18-crown-6 (528.6 mg, 2.0 mmol, 2.0 equiv), KHMDS (1 M solution in THF, 2.0 mL, 2.0 mmol, 2.0 equiv), and anhydrous THF (0.5 mL) at room temperature for 2 h. ^{1}H NMR spectroscopy of the crude reaction mixture was used to evaluate the selectivity of 1-(1-(2-bromo-3-methylphenyl)ethoxy)-2,2,6,6-tetramethylpiperidine (>20:1, 2° selective). Due to partial overlap between the product methine signal and KHMDS impurity (see General Reagent Information section for details), the crude yield reported was calculated from a 0.1 mmol scale reaction which uses solid KHMDS and does not have the impurity. Using TMB as an internal standard, an 84% yield was calculated (84 µmol, 84%). The crude residue was filtered through silica with DCM (200 mL) and the filtrate was collected and concentrated *in vacuo*. The concentrated filtrate was then filtered once more through silica with 5% EtOAc / hexanes until the product eluted (200 mL) to afford the product as a yellow oil (204.0 mg, 0.58 mmol, 58% yield). **^{1}H NMR** (400 MHz, $CDCl_3$) δ 7.41 (dd, *J* = 7.8, 1.8 Hz, 1H), 7.23 (t, *J* = 7.5 Hz, 1H), 7.13 (ddd, *J* = 7.4, 1.8, 0.8 Hz, 1H), 5.26 (q, *J* = 6.5 Hz, 1H), 2.43 (s, 3H), 1.80 – 0.50 (m, 21H). **^{13}C NMR** (101 MHz, $CDCl_3$) δ 146.4, 137.8, 128.9, 127.1, 125.4, 124.1, 83.0, 60.0, 59.4, 40.5, 34.9, 33.5, 24.1, 23.1, 20.5, 17.4. **IR** (neat, cm^{-1}) 2972, 2928, 2870, 1464, 1450, 1374, 1360, 1242, 1132, 1045, 1023, 937, 781, 713. **HRMS** (ESI) $[M+H]^+$ calcd. for $[C_{18}H_{29}BrNO]^+$ = 354.1428, 354.1448.

2-ethyl-6-(((2,2,6,6-tetramethylpiperidin-1-yl)oxy)methyl)pyridine (1° selective functionalization of 38)

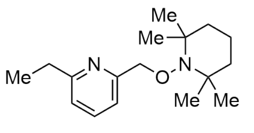

The title product was prepared according to **GP3** using 2-ethyl-6-methylpyridine (121.2 mg, 1.0 mmol), TEMPO (390.8 mg, 2.5 mmol, 2.5 equiv), KHMDS (219.5 mg, 1.1 mmol, 1.1 equiv), and benzene (2.5 mL). ^{1}H NMR spectroscopy of the crude reaction mixture was used to evaluate the yield of 2-ethyl-6-(((2,2,6,6-tetramethylpiperidin-1-yl)oxy)methyl)pyridine (997 µmol, 100% yield; 4 : 1, 1° selective; note: this yield was reported as >90% in Scheme 1). The product was purified *via* preparatory thin layer chromatography, as the product and residual TEMPO exhibit very poor resolution on silica. The crude residue was loaded onto a preparatory TLC plate and developed twice with 20% Et_2O in DCM to afford the product as a brown oil (102.2 mg, 0.37 mmol, 37% yield). **^{1}H NMR** (400 MHz, $CDCl_3$) δ 7.60 (t, *J* = 7.7 Hz, 1H), 7.35 (d, *J* = 7.7 Hz, 1H), 7.03 (d, *J* = 7.7 Hz, 1H), 4.97 (s, 2H), 2.79 (q, *J* = 7.6 Hz, 2H), 1.75 – 1.32 (m, 7H), 1.29 (t, *J* = 7.6 Hz, 3H), 1.20 (d, *J* = 9.4 Hz, 11H). **^{13}C NMR** (101 MHz, $CDCl_3$) δ 162.8, 158.3, 136.8, 120.1, 117.9, 79.9, 60.0, 39.8, 33.1, 31.5, 20.4, 17.2, 14.2. **IR** (neat, cm^{-1}) 3002, 2962, 2933, 2870, 1497, 1455, 1374, 1244, 1132, 973, 808. **HRMS** (ESI) $[M+H]^+$ calcd. for $[C_{17}H_{29}N_2O]^+$ = 277.2275, 277.2299 found.

2-methyl-6-(1-((2,2,6,6-tetramethylpiperidin-1-yl)oxy)ethyl)pyridine (2° selective functionalization of 38)

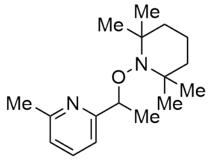

The title product was prepared according to **GP2** using 2-ethyl-6-methylpyridine (121.2 mg, 1.0 mmol), TEMPO (199.9 mg, 2.5 mmol, 2.5 equiv), 18-crown-6 (528.6 mg, 2.0 mmol, 2.0 equiv), KHMDS (1 M solution in THF, 2.0 mL, 2.0 mmol, 2.0 equiv), and anhydrous THF (0.5 mL) at rt for 2 h. ^{1}H NMR spectroscopy of the crude reaction mixture was used to evaluate the yield of 2-methyl-6-(1-((2,2,6,6-tetramethylpiperidin-1-yl)oxy)ethyl)pyridine (965 µmol, 97% yield, > 20 : 1, 2° selective; note: this yield was reported as >90% in Scheme 1). The product was purified *via* silica flash column chromatography with eluent conditions 2.5% → 10% EtOAc in hexanes. Fractions that coeluted with TEMPO were filtered through silica with DCM (discarded) followed by a flush with MeOH (collected, concentrated *in vacuo*) to afford the product as a white solid (207.4 mg, 0.75 mmol, 75% yield). **MP:** 81-83 °C. **^{1}H NMR** (401 MHz, $CDCl_3$) δ 7.51 (t, *J* = 7.7 Hz, 1H), 7.23 (d, *J* = 7.8 Hz, 1H), 6.95 (d, *J* = 7.6 Hz, 1H), 4.87 (q, *J* = 6.7 Hz, 1H), 2.51 (s, 3H), 1.85 – 0.83 (m, 18H), 0.64 (s, 3H). **^{13}C NMR** (101 MHz, $CDCl_3$) δ 164.4, 157.0, 136.4, 121.2, 117.9, 84.6, 59.6, 40.3, 34.3, 33.9, 24.5, 22.4, 20.4, 17.2. **IR** (neat, cm^{-1}) 2990, 2966, 2934, 2918, 2875, 1593, 1575, 1461, 1359, 973, 809, 760, 697. **HRMS** (ESI) $[M+H]^+$ calcd. for $[C_{17}H_{29}N_2O]^+$ = 277.2275, 277.2298.

2,2,6,6-tetramethyl-1-((4'-pentyl-[1,1'-biphenyl]-4-yl)methoxy)piperidine (1° selective functionalization of 39)

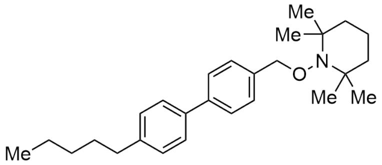

The title product was prepared according to **GP1** using 4-methyl-4'-pentyl-1,1'-biphenyl (238.4 mg, 1.0 mmol), TEMPO (390.8 mg, 2.5 mmol, 2.5 equiv), KHMDS (1 M solution in THF, 3.0 mL, 3.0 mmol, 3.0 equiv), and anhydrous THF (0.5 mL) at 50 °C. ^{1}H NMR spectroscopy of the crude reaction mixture was used to evaluate the yield of 2,2,6,6-tetramethyl-1-((4'-pentyl-[1,1'-biphenyl]-4-yl)methoxy)piperidine (565 µmol, 57% yield > 20: 1, 1° selective). The product was purified *via* silica flash column chromatography with eluent conditions

25% DCM in hexanes → 100% DCM to afford the product as a white solid (181.3 mg, 0.46 mmol, 46% yield). **MP:** 41-43 °C. **^{1}H NMR** (400 MHz, $CDCl_3$) δ 7.61 (d, J = 8.2 Hz, 2H), 7.56 (d, J = 8.2 Hz, 2H), 7.48 (d, J = 8.2 Hz, 2H), 7.30 (d, J = 8.1 Hz, 2H), 4.92 (s, 2H), 2.69 (dd, J = 8.8, 6.7 Hz, 2H), 1.84 – 1.49 (m, 7H), 1.48 – 1.37 (m, 5H), 1.34 (s, 6H), 1.23 (s, 6H), 1.06 – 0.92 (m, 3H). **^{13}C NMR** (101 MHz, $CDCl_3$) δ 142.1, 140.4, 138.5, 137.1, 128.9, 128.0, 127.1, 127.0, 78.7, 60.2, 39.9, 35.7, 33.3, 31.7, 31.3, 22.7, 20.5, 17.3, 14.2. **IR** (neat, cm^{-1}) 3002, 2971, 2927, 2869, 1501, 1468, 1372, 1358, 1132, 1055, 1025, 990, 860, 799. **HRMS** (ESI) $[M+H]^+$ calcd. for $[C_{27}H_{40}NO]^+$ = 394.3105, 394.3150.

2,2,6,6-tetramethyl-1-((1-(4'-methyl-[1,1'-biphenyl]-4-yl)pentyl)oxy)piperidine (2° selective functionalization of 39)

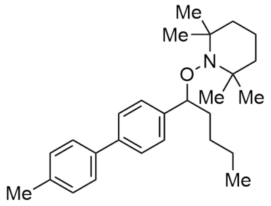

The title product was prepared according to **GP2** using 4-methyl-4'-pentyl-1,1'-biphenyl (238.4 mg, 1.0 mmol), TEMPO (199.9 mg, 2.5 mmol, 2.5 equiv), 18-crown-6 (528.6 mg, 2.0 mmol, 2.0 equiv), KHMDS (1 M solution in THF, 2.0 mL, 2.0 mmol, 2.0 equiv), and anhydrous THF (0.5 mL) at rt for 2 h. ^{1}H NMR spectroscopy of the crude reaction mixture was used to evaluate the yield of 2,2,6,6-tetramethyl-1-((1-(4'-methyl-[1,1'-biphenyl]-4-yl)pentyl)oxy)piperidine (709 μmol, 71% yield, >20 : 1, 2° selective). The product was purified *via* silica flash column chromatography with eluent conditions 50% DCM in hexanes → 100% DCM to afford the product as a white solid (148.3 mg, 0.38 mmol, 38% yield). **MP:** 84-86 °C. **^{1}H NMR** (400 MHz, $CDCl_3$) δ 7.52 (dd, J = 8.1, 1.4 Hz, 4H), 7.35 – 7.28 (m, 2H), 7.23 (d, J = 7.9 Hz, 2H), 4.60 (dd, J = 9.8, 3.9 Hz, 1H), 2.39 (s, 3H), 2.16 – 2.04 (m, 1H), 1.80 (dtd, J = 13.0, 10.0, 5.3 Hz, 1H), 1.63 – 0.92 (m, 19H), 0.82 (t, J = 7.3 Hz, 3H), 0.59 (s, 3H). **^{13}C NMR** (101 MHz, $CDCl_3$) δ 142.7, 139.5, 138.2, 136.7, 129.4, 128.2, 126.8, 126.2, 87.3, 60.0, 59.5, 40.5, 35.7, 34.4, 34.3, 27.6, 22.8, 21.1, 20.4, 17.3, 14.1. **IR** (neat, cm^{-1}) 3003, 2960, 2934, 2861, 1497, 1456, 1374, 1244, 1131, 985, 849. **HRMS** (ESI) $[M+H]^+$ calcd. for $[C_{27}H_{40}NO]^+$ = 394.3105, 394.3136**.**

1-((4-((4-ethylphenyl)ethynyl)benzyl)oxy)-2,2,6,6-tetramethylpiperidine (1° selective functionalization of 40)

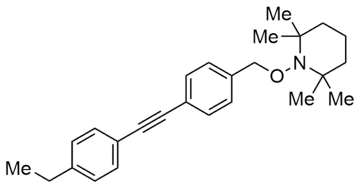

The title product was prepared according to **GP1** using 1-ethyl-4-(p-tolylethynyl)benzene (220.3 mg, 1.0 mmol), TEMPO (390.8 mg, 2.5 mmol, 2.5 equiv), KHMDS (1 M solution in THF, 2.0 mL, 2.0 mmol, 2.0 equiv), and anhydrous THF (0.5 mL). ^{1}H NMR spectroscopy of the crude reaction mixture was used to evaluate the yield of 1-((4-((4-ethylphenyl)ethynyl)benzyl)oxy)-2,2,6,6-tetramethylpiperidine. Full conversion was observed and the yield was normalized to 100% (original mass balance = 105%) and a >90% yield was reported (4 : 1, 1° selective). The product was purified *via* silica flash column chromatography with eluent conditions 50% DCM in hexanes → 100% DCM to afford the product as a white solid (199.1 mg, 0.53 mmol, 53% yield). **MP:** 109-110 °C. **^{1}H NMR** (401 MHz, $CDCl_3$) δ 7.49 (dd, J = 16.4, 8.2 Hz, 4H), 7.35 (d, J = 8.1 Hz, 2H), 7.19 (d, J = 8.1 Hz, 2H), 4.85 (s, 2H), 2.68 (q, J = 7.6 Hz, 2H), 1.73 – 1.32 (m, 6H), 1.30 – 1.22 (m, 9H), 1.18 (s, 6H). **^{13}C NMR** (101 MHz, $CDCl_3$) δ 144.7, 138.5, 131.7, 131.6, 128.0, 127.4, 122.5, 120.7, 89.5, 88.9, 78.6, 60.2, 39.9, 33.2, 29.0, 20.4, 17.3, 15.5. **IR** (neat, cm^{-1}) 3002, 2966, 2931, 2867, 1516, 1466, 1370, 1359, 1131, 1059, 829, 815. **HRMS** (ESI) $[M+H]^+$ calcd. for $[C_{26}H_{34}NO]^+$ = 376.2635, 376.2662 found.

2,2,6,6-tetramethyl-1-(1-(4-(p-tolylethynyl)phenyl)ethoxy)piperidine (2° selective functionalization of 40)

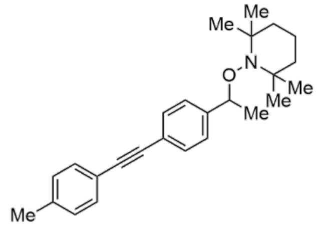

The title product was prepared according to **GP2** using 1-ethyl-4-(p-tolylethynyl)benzene (220.3 mg, 1.0 mmol), TEMPO (199.9 mg, 2.5 mmol, 2.5 equiv), 18-crown-6 (264.3 mg, 1.0 mmol, 1.0 equiv), KHMDS (1 M solution in THF, 3.0 mL, 3.0 mmol, 3.0 equiv), and anhydrous THF (0.5 mL) at rt for 2 h. ^{1}H NMR spectroscopy of the crude reaction mixture was used to evaluate the yield of 2,2,6,6-tetramethyl-1-(1-(4-(p-tolylethynyl)phenyl)ethoxy)piperidine (904 µmol, 90% yield, >20 : 1, 2° selective). The product was purified *via* silica flash column chromatography with eluent conditions 50% DCM in hexanes → 100% DCM to afford the product as a white solid (212.1 mg, 0.56 mmol, 56% yield). **MP:** 116-117 °C. **^{1}H NMR** (401 MHz, $CDCl_3$) δ 7.50 (d, *J* = 8.3 Hz, 2H), 7.44 (d, *J* = 8.2 Hz, 2H), 7.32 (d, *J* = 8.2 Hz, 2H), 7.17 (d, *J* = 7.9 Hz, 2H), 4.80 (q, *J* = 6.7 Hz, 1H), 2.38 (s, 3H), 1.76 – 0.89 (m, 18H), 0.68 (s, 3H). **^{13}C NMR** (101 MHz, $CDCl_3$) δ 146.1, 138.3, 131.6, 131.4, 129.2, 126.7, 121.9, 120.5, 89.3, 89.0, 83.1, 59.9, 59.8, 40.5, 34.6, 34.4, 23.5, 21.6, 20.4, 17.3. **IR** (neat, cm^{-1}) 3003, 2971, 2988, 2928, 2865, 1589, 1443, 1358, 1131, 985, 836, 759, 710, 694. **HRMS** (ESI) $[M+H]^+$ calcd. for $[C_{26}H_{34}NO]^+$ = 376.2635, 376.2675 found.

2-propyl-6-(((2,2,6,6-tetramethylpiperidin-1-yl)oxy)methyl)pyridine (1° selective functionalization of 41)

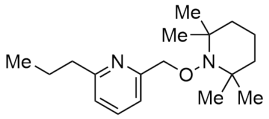

The title product was prepared according to **GP3** using 2-methyl-6-propylpyridine (135.2 mg, 1.0 mmol), TEMPO (390.8 mg, 2.5 mmol, 2.5 equiv), KHMDS (219.5 mg, 1.1 mmol, 1.1 equiv), and benzene (2.5 mL). ^{1}H NMR spectroscopy of the crude reaction mixture was used to evaluate the yield of 2-propyl-6-(((2,2,6,6-tetramethylpiperidin-1-yl)oxy)methyl)pyridine both isomers: (853 µmol, 85% yield, 10:1, 1° selective). The product was purified *via* silica flash column chromatography with eluent conditions 100% DCM until TEMPO eluted then 5% MeOH in DCM to afford the product as a bronze oil (173.4 mg, 0.6 mmol, 60% yield). **^{1}H NMR** (400 MHz, $CDCl_3$) δ 7.59 (t, *J* = 7.7 Hz, 1H), 7.36 (d, *J* = 7.6 Hz, 1H), 7.00 (d, *J* = 7.6 Hz, 1H), 4.99 (s, 2H), 2.79 – 2.69 (m, 2H), 1.73 (dt, *J* = 14.8, 7.4 Hz, 2H), 1.67 – 1.43 (m, 5H), 1.21 (d, *J* = 9.5 Hz, 13H), 0.98 (t, *J* = 7.3 Hz, 3H). **^{13}C NMR** (101 MHz, $CDCl_3$) δ 160.8, 157.7, 135.9, 120.2, 117.3, 79.3, 59.4, 39.9, 39.2, 32.5, 22.7, 19.7, 16.6, 13.4. **IR** (neat, cm^{-1}) 2964, 2929, 2870, 591, 1579, 1455, 1374, 1359, 1262, 1245, 1132, 1083, 1055, 992, 788. **HRMS** (ESI) $[M+H]^+$ calcd. for $[C_{18}H_{31}N_2O]^+$ = 291.2431, 291.2468 found.

2-methyl-6-(1-((2,2,6,6-tetramethylpiperidin-1-yl)oxy)propyl)pyridine (2° selective functionalization of 41)

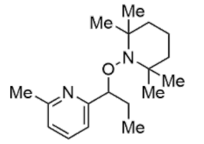

The title product was prepared according to **GP2** using 2-methyl-6-propylpyridine (135.2 mg, 1.0 mmol), TEMPO (199.9 mg, 2.5 mmol, 2.5 equiv), 18-crown-6 (528.6 mg, 2.0 mmol, 2.0 equiv), KHMDS (1 M solution in THF, 2.0 mL, 2.0 mmol, 2.0 equiv), and anhydrous THF (0.5 mL) at rt for 2 h. ^{1}H NMR spectroscopy of the crude reaction mixture was used to evaluate the yield of 2-methyl-6-(1-((2,2,6,6-tetramethylpiperidin-1-yl)oxy)propyl)pyridine (871 µmol, 87% yield, >20 : 1, 2° selective). The product was purified *via* silica flash column chromatography with eluent conditions 100% DCM until TEMPO eluted then 4% MeOH in DCM to afford

the product as an off-white solid (237.2 mg, 0.82 mmol, 82% yield). **MP:** 51-52 °C. **^{1}H NMR** (400 MHz, $CDCl_3$) δ 7.50 (t, *J* = 7.6 Hz, 1H), 7.17 (d, *J* = 7.7 Hz, 1H), 6.97 (d, *J* = 7.5 Hz, 1H), 4.70 (dd, *J* = 8.8, 3.8 Hz, 1H), 2.53 (s, 3H), 2.20 – 1.83 (m, 2H), 1.71 – 0.86 (m, 15H), 0.67 (t, *J* = 7.5 Hz, 3H), 0.51 (br s, 3H). **^{13}C NMR** (101 MHz, $CDCl_3$) δ 162.6, 157.2, 135.9, 121.3, 119.8, 89.5, 59.7, 40.5, 34.2, 33.8, 27.7, 24.7, 20.4, 17.3, 9.0. **IR** (neat, cm^{-1}) 2990, 2966, 2934, 2916, 2875, 1594, 1574, 1461, 1378, 1256, 1166, 1132, 1092, 973, 954, 918, 809, 760, 697. **HRMS** (ESI) $[M+H]^+$ calcd. for $[C_{18}H_{31}N_2O]^+$ = 291.2431, 291.2452 found.

V. Determination of Site Selectivity

Discussion: To determine selectivity for compounds with potential for multiple sites of functionalization, nuclear Overhauser effect NMR spectroscopy (NOESY) was used. For substrates not described below, the selectivity was assessed using obvious NMR correlations or signals, such as multiplicity (e.g., for 1° vs 2° selectivity).

a) Determination of site selectivity for 2-(3-phenyl-1-((2,2,6,6-tetramethylpiperidin-1-yl)oxy)propyl)pyridine (34)

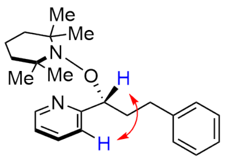

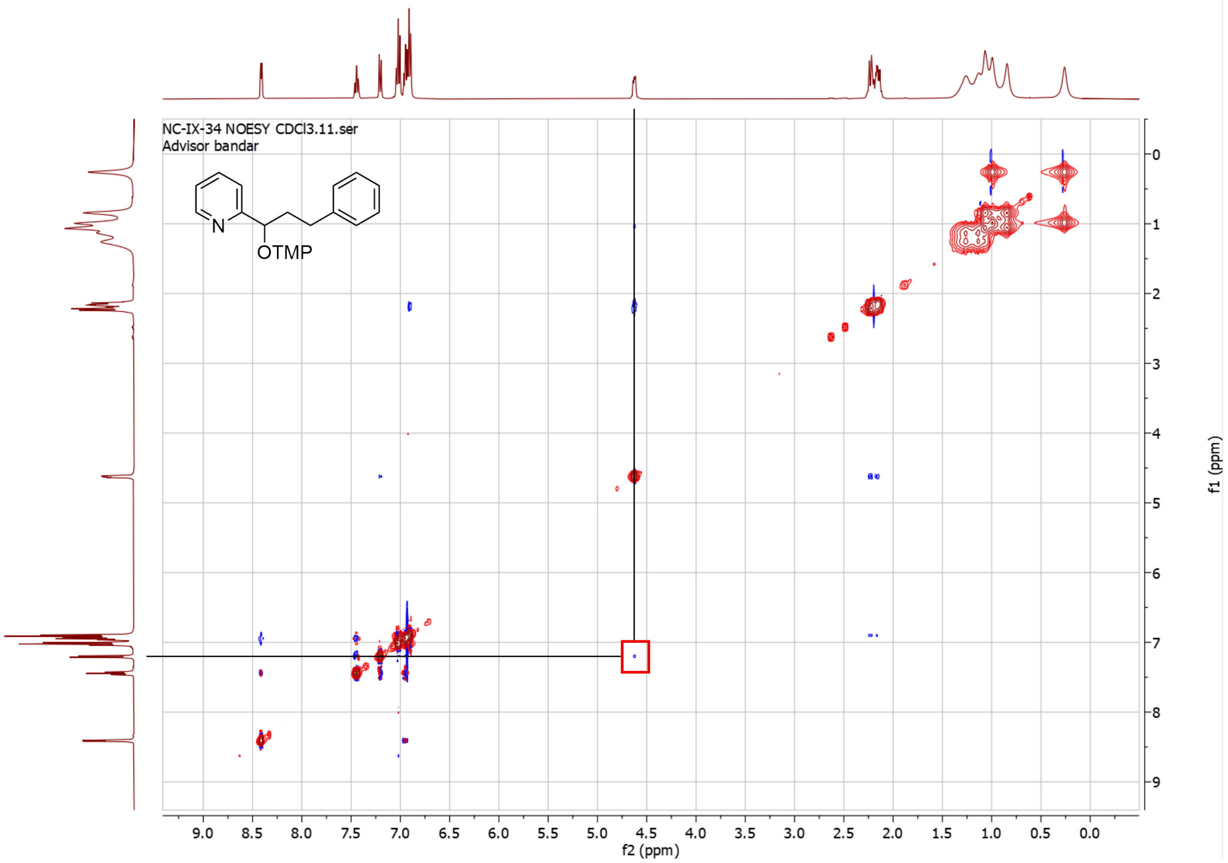

Figure S4: NOESY ^{1}H NMR spectrum of 2-(3-phenyl-1-((2,2,6,6-tetramethylpiperidin-1-yl)oxy)propyl)pyridine (**34**).

Analysis: The methine benzylic proton at 4.6 ppm (m, 1H) correlates to the 3-proton on the pyridine ring at 7.2 ppm (d, J = 7.8 Hz), suggesting functionalization near the pyridine ring. We therefore conclude that TEMPO incorporation occurred adjacent to the pyridine.

b) Determination of site selectivity for 2,2,6,6-tetramethyl-1-((3'-methyl-[1,1'-biphenyl]-4-yl)methoxy)piperidine (36)

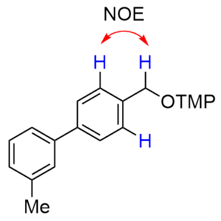

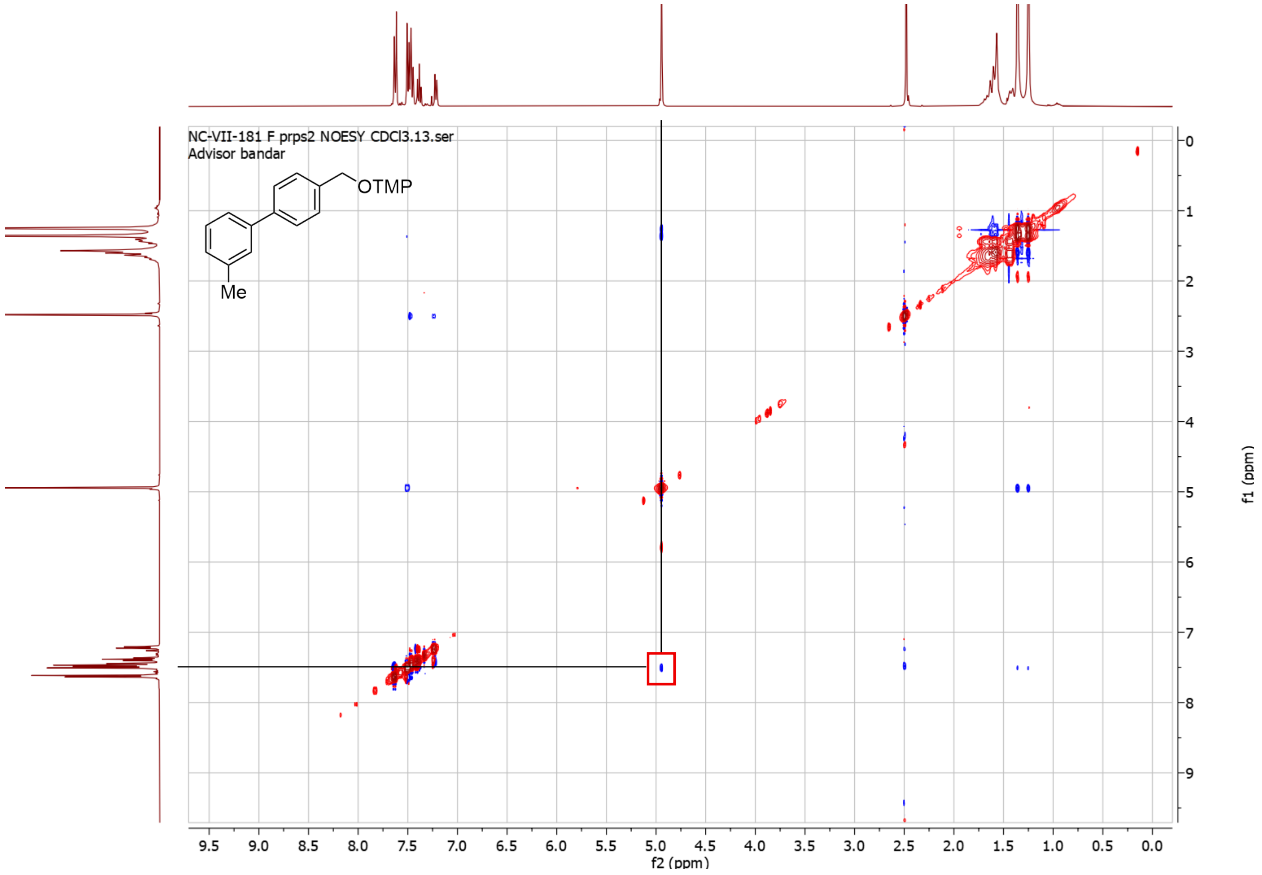

Figure S5: NOESY ^{1}H NMR spectrum of 2,2,6,6-tetramethyl-1-((3'-methyl-[1,1'-biphenyl]-4-yl)methoxy)piperidine (**36**)

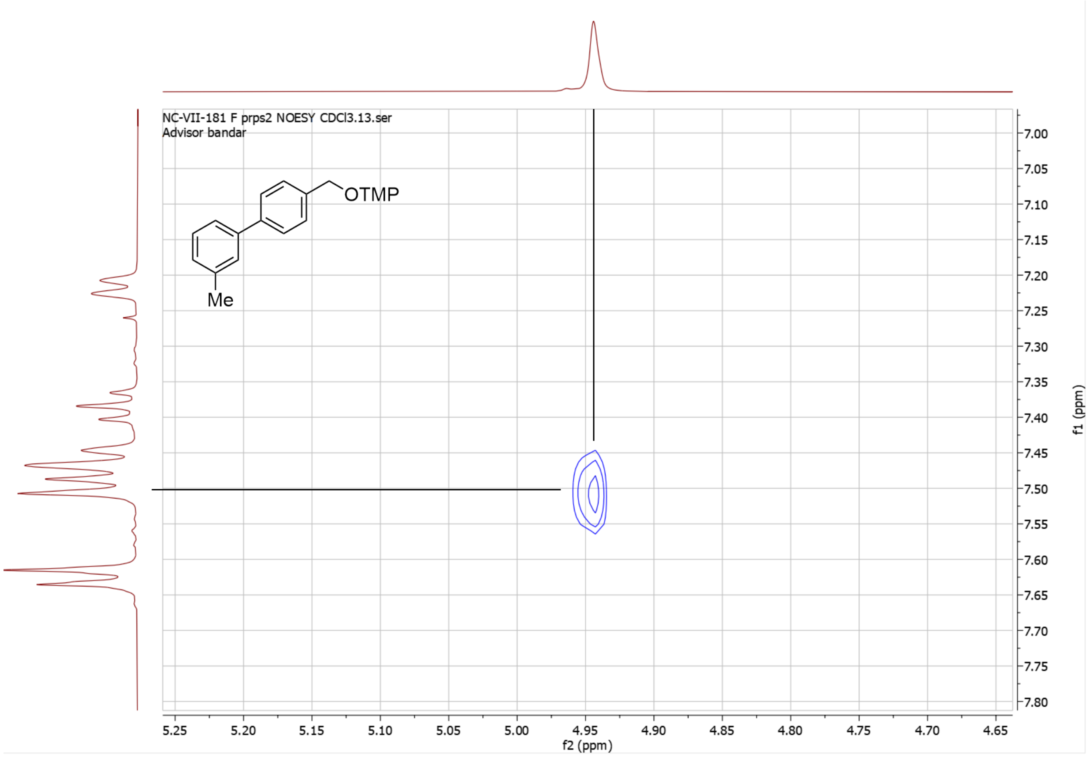

Figure S6: Spectral window of ^{1}H NOESY NMR spectrum of **36**.

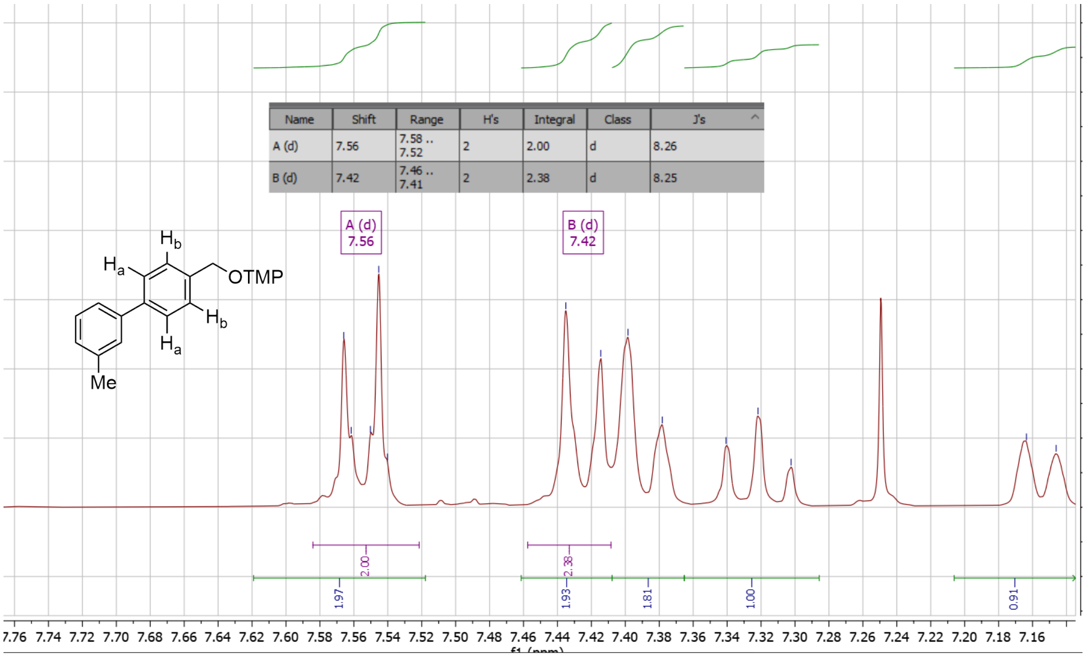

Figure S7: Spectral window of ^{1}H NMR spectrum of **36** displaying two pairs of aromatic doublets that each couple and integrate to 2H, indicating that they are on the symmetrical benzene ring.

Analysis: The methylene protons at 4.9 ppm (s, 2H) of **36** correlate to the doublet at 7.4 ppm (d, J = 8.25 Hz) which are on the symmetrical benzene ring, as they integrate to 2H and couple to the other protons on

the symmetrical benzene ring at 7.56 ppm (d, J = 8.26 Hz) which also integrate to 2H. Therefore, we conclude that TEMPO incorporation occurred at the 4'-methyl position of 3,4'-dimethyl-1,1'-biphenyl.

c) Determination of site selectivity for 1-((2,6-dibromo-3-methylbenzyl)oxy)-2,2,6,6-tetramethylpiperidine (33)

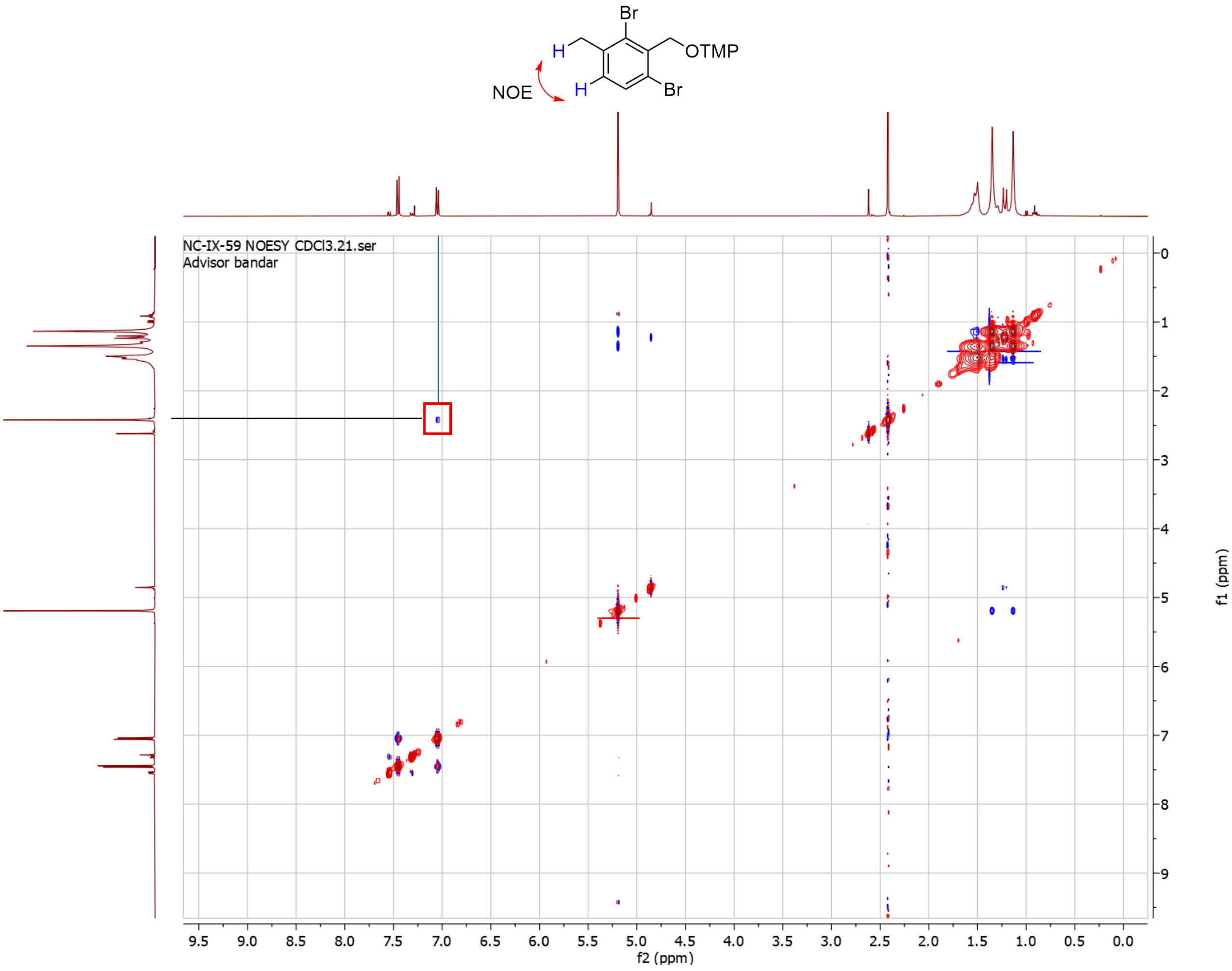

Figure S8: ^{1}H NOESY spectrum of **33**.

Analysis: The unfunctionalized methyl signal of **33** at 2.4 ppm (s, 3H) correlates with the aromatic signal at 7.0 ppm (dd, J = 8.2, 0.7 Hz, 1H) while the methylene signal at 5.2 ppm (s, 2H) does not correlate with an aromatic signal, suggesting that functionalization occurred at the 2-methyl of the arene. We therefore conclude that TEMPO incorporation occurred at the 2-position of 1,3-dibromo-2,4-dimethylbenzene.

VI. Mechanistic Studies and Control Reactions Experimental Details

a) Base-promoted deuterium exchange experiments of model substrates 1-3.

Discussion: The known acidity value of HMDS bases' conjugate acid (pK_a of H–N($SiMe_3$)$_2$ approximately 26 in DMSO)[6] indicates that the deprotonation of benzylic C–H bonds is generally an uphill process. However, these bases are known to activate benzylic positions towards addition reactions to electrophiles.[7] In this work, for the more acidic and moderately acidic alkylarenes (pK_a ~ 31 - 36), Li, Na, and KHMDS are used, while the less acidic alkylarenes (pK_a ≥ 40) require 18-crown-6 additive (increasing HMDS kinetic basicity) to achieve reactivity (see **Table S1-S3**). We sought to provide evidence for deprotonation of these benzylic positions when subjected to the optimized basic conditions. To do this, we conducted two types of deuterium exchange experiments on alkylarenes **1-3**:

First, to support the hypothesis that deprotonation can occur readily, we subjected a mixture of alkylarene and *tert*-butanol-d_{10} (1 equiv) to basic conditions and observed the resulting deuterium incorporation after stirring for 1 hour at room temperature (unless noted). Deuterium incorporation would be consistent with reversible deprotonation under these conditions, with the *in situ tert*-butanol-d_{10} serving as the deuterium source. At 1 hour, excess methanol (CH_3OH) was added to effectively stop the deprotonation reaction before analysis by ^{1}H NMR spectroscopy. These experiments are described in Section VIa.

Second, to assess the equilibrium position of deprotonation, we conducted an experiment in which base and alkylarene **2** were stirred together for 30 seconds (or 1 hour) and then excess methanol-d_4 was added rapidly. We rationalized that if any benzylic carbanionic intermediate were present at equilibrium, it would be deuterated upon addition of methanol-d_4. If the equilibrium has a very low concentration of benzyl carbanionic intermediate in solution, then no deuterated material would be detectable by ^{1}H NMR spectroscopy, consistent with the hypothesis of an uphill deprotonation step. These experiments are described in Section VIb.

Analysis: subjection of alkylarene and an exchangeable deuterium source to their respective basic conditions (described in Table S1-S3) results in benzylic deuterium incorporation for substrates **1-3**. These results are consistent with the basic conditions enabling reversible benzylic deprotonation.

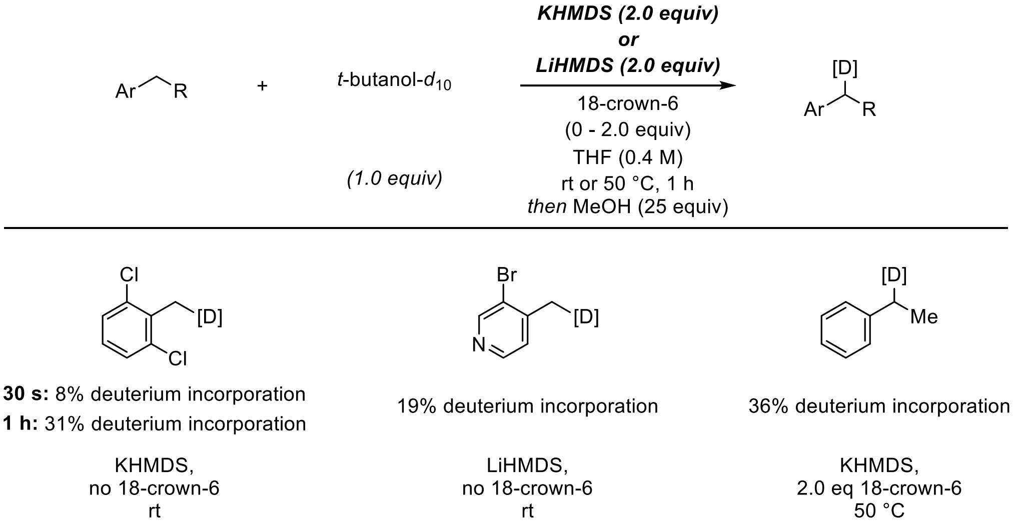

Procedure: Alkylarene (0.1 mmol, 1.0 equiv) was added *via* pipette to a 4 mL, oven-dried dram vial with a stir bar. The vial was sealed with a screwcap with a septum and was brought into a N_2 filled glovebox.

The vial was then unsealed and THF (0.25 mL, 0.4 M) was added *via* pipette. If necessary, 18-crown-6 (52.9 mg, 0.2 mmol, 2.0 equiv) was added at this time. *Tert*-butanol-d_{10} (9.4 µL, 0.1 mmol, 1.0 equiv) was added *via* microsyringe. Base (0.2 mmol, 2.0 equiv) was added. For the reaction stopped at 30 seconds, the vial was placed on a stir plate and the reaction mixture was stirred for 30 seconds and then quenched with methanol (0.1 mL, 2.47 mmol, 24.7 equiv) which was added *via* syringe in the glovebox. Otherwise, the vial was sealed with a screwcap and septum, removed from the glovebox and the reaction mixture was stirred at rt or 50 °C for 1 h. At this time, the vial was unsealed and methanol (0.1 mL, 2.47 mmol, 24.7 equiv) was added *via* syringe. TMB was then added to the crude reaction mixture. The mass of TMB added to the vial for each experiment was weighed and recorded separately. A small aliquot from the crude reaction mixture was removed, charged into an NMR tube, and constituted in $CDCl_3$ (0.5 mL). ^1H NMR spectroscopy (400 MHz, $CDCl_3$) was used to determine the degree of deuterium incorporation. A 10 second relaxation delay was used to acquire quantitative ^1H NMR data. TMB was used to assess mass balance. An aromatic signal (alkylarene **1** and **2**) or alkyl signal (for alkylarene **3**) of the alkylarene was set to a known integration based on the number of protons expected in that signal. Then the benzylic signal was integrated against the signal with set value to determine the percentage deuterium exchange. For example, the methyl signal of 1,3-dichloro-2-methylbenzene at 2.45 ppm (m) was integrated against the aromatic signal of 1,3-dichloromethyl benzene at 7.05 and the reduction of the signal integration from expected integral of 3.00 was used to determine the percentage deuterium exchange. An example analysis for substrate **1** is shown below.

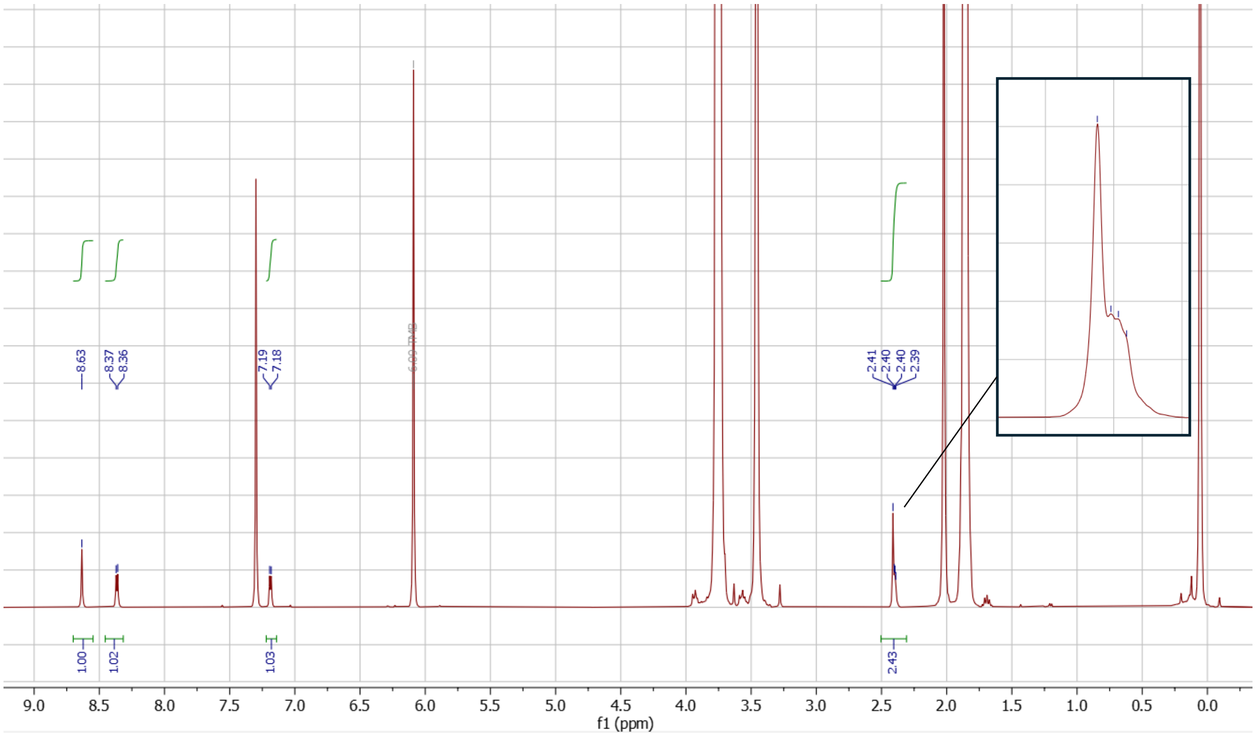

Figure S9: ^1H NMR spectrum of the crude reaction solution of 3-bromo-4-methylpyridine (**1**) reaction with *tert*-butanol-d_{10} after 1 hour of reaction. The percent deuterium exchange is based on the reduction of 3-bromo-4-methylpyridine's methyl signal (2.40 ppm, 3H) integration from expected 3.00 when integrated against an aromatic signal set to 1.00. **Note:** the change in signal appearance (see zoomed in window) is also consistent with deuterium exchange.

b) Assessment of deprotonative equilibrium position for 1,3-dichloro-2-methylbenzene (2).

Discussion: This section describes the experimental information for the second deuterium incorporation study described in Section VIa.

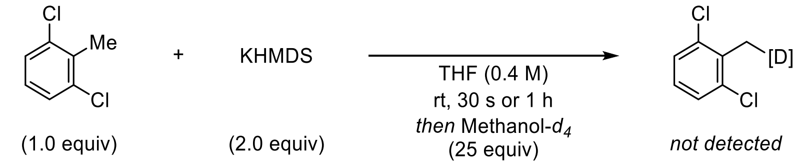

Procedure: 1,3-Dichloro-2-methylbenzene (16.1 mg, 0.1 mmol, 1.0 equiv) was added *via* pipette to a 4 mL, oven-dried dram vial with a stir bar. The vial was sealed with a screwcap lined with a PTFE septum and brought into a N_2 filled glovebox. The vial was unsealed, and THF (0.25 mL, 0.4 M) was added *via* micropipette. KHMDS (39.9 mg, 0.2 mmol, 2.0 equiv) was then added. For the reaction run for 30 s, the vial was sealed and the reaction mixture was stirred for 30 seconds at rt in the glovebox. At this time, the vial was unsealed and methanol-d_4 (0.1 ml, 2.46 mmol, 25 equiv) was added *via* syringe to quench the reaction mixture. For the reaction run for 1 h, the vial was sealed, removed from the glovebox and the reaction mixture was stirred at rt for 1 h. At this time, the vial was unsealed and methanol-d_4 (0.1 ml, 2.46 mmol, 25 equiv) was added *via* syringe. TMB was added to the vial and the mass of standard added was recorded. ^{1}H NMR spectroscopy (400 MHz, $CDCl_3$) was used to determine the degree of deuterium incorporation. A 10 second relaxation delay was used to acquire quantitative ^{1}H NMR data.

Analysis: TMB was used to assess mass balance using the ^{1}H NMR spectrum of the quenched crude reaction mixture. For the 30 second reaction time experiment, the aromatic signal of 1,3-dichloro-2-methylbenzene at 7.05 ppm (t, J = 8.0 Hz, 1H) was integrated against the aromatic signal of TMB at 6.09 (s, 3H) to assess mass balance (100 µmol, 100% recovery). The methyl signal of 1,3-dichloro-2-methylbenzene at 2.47 ppm (s, 3H) was integrated against the aromatic signal of TMB at 6.09 ppm to assess the percentage deuterium exchange (100 µmol, 100% recovery). The result of this deuterium incorporation experiment is consistent with endergonic deprotonation and subsequent low concentration of carbanionic intermediate as deuterium incorporation is not detectable by ^{1}H NMR spectroscopy, suggesting the equilibrium substantially favors neutral 1,3-dichloro-2-methylbenzene. Furthermore, no visible change in signal appearance is observable (see Figure S9 for contrast). An identical reaction was conducted for 1 h before methanol-d_4 addition in which deuterium exchange at the benzylic position was also not observed (no change in methyl signal appearance, aromatic signal of **2** at 7.05 ppm integrates 1 : 3 with respect to the methyl signal of **2** at 2.47 ppm) albeit with lower mass balance (81% recovery), likely due to slow base-promoted decomposition of the substrate.

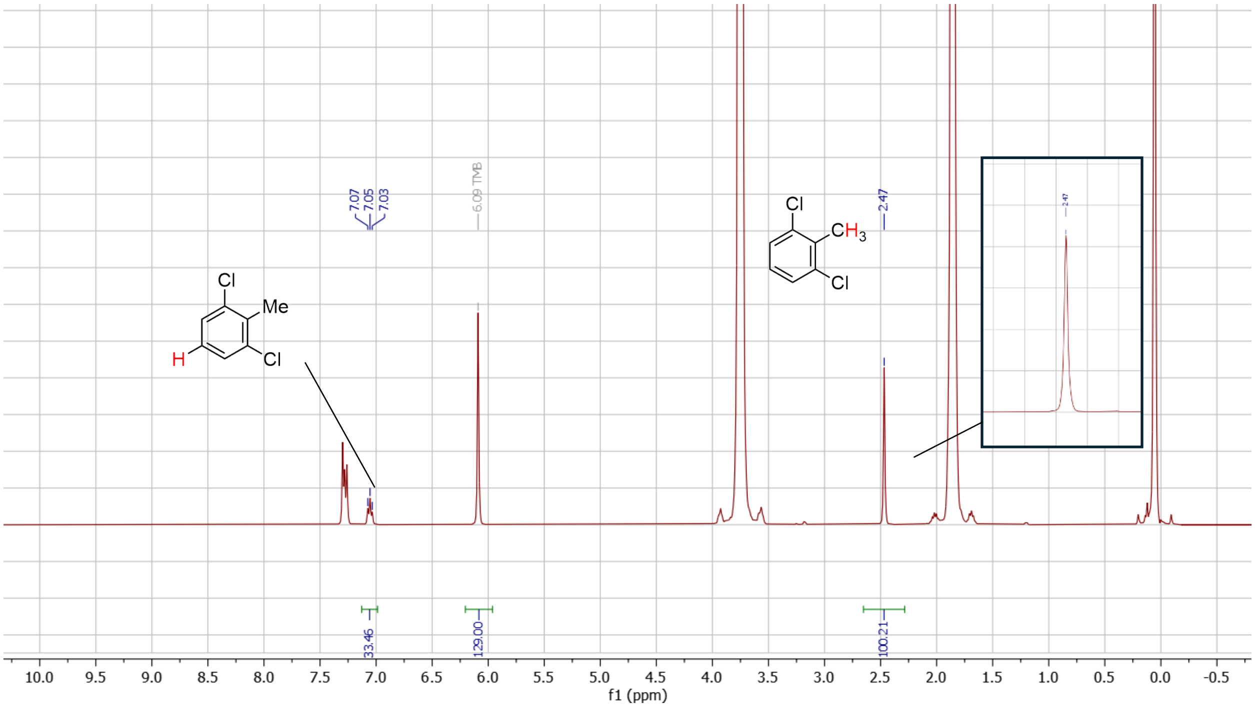

Figure S10: ^{1}H NMR spectrum for deuterium source quenching experiment with 1,3-dichloro-2-methylbenzene (**2**) (30 second reaction). TMB (21.7 mg, 129 μmol) was used as an internal standard. The results are consistent with no detectable deuterium exchange occurring at the benzylic position of 1,3-dichloro-2-methylbenzene, consistent with uphill deprotonation.

c) Evidence for benzylic deprotonation of complex substrates with acidic functionality.

Discussion: To provide evidence for the feasibility of benzylic deprotonation for alkylarenes bearing acidic functionality, we conducted deuterium exchange studies under the optimized reaction conditions on 1-methyl-3-(3-methyl-4-(4-((trifluoromethyl)thio)phenoxy)phenyl)-1,3,5-triazinane-2,4,6-trione (herein referred to as toltrazuril for conciseness, the starting material for compound **26**) and (2*S*,3*R*,4*R*,5*S*,6*R*)-2-(4-chloro-3-(4-(((*S*)-tetrahydrofuran-3-yl)oxy)benzyl)phenyl)-6-(hydroxymethyl)tetrahydro-2H-pyran-3,4,5-triol (referred to as empagliflozin herein for conciseness, the starting material for compound **27**). Toltrazuril bears an acidic isocyanuric N–H bond which is likely stoichiometrically (or nearly stoichiometrically) deprotonated after addition of base. Because excess base is used, it is possible for the remaining HMDS base to promote benzylic C–H deprotonation. Empagliflozin, on the other hand, contains four alcohols which are most likely in equilibrium with their alkoxide conjugate base after addition of KHMDS base ($H–N(SiMe_3)_2$ p*Ka* approximately 26 in DMSO[6] and alcohol pK_a approximately 30 in DMSO[8]), with the equilibrium likely favoring protonated alcohol. Because of this, we hypothesize that a sufficient amount of HMDS base remains in solution to enable productive diarylmethane benzylic deprotonation. It may also be possible that an alkoxide intermediate could act as a suitable base to promote the reaction. Regardless, the deuterium exchange studies shown below indicate that benzylic deprotonation can occur for both substrates.

Benzylic deuterium incorporation

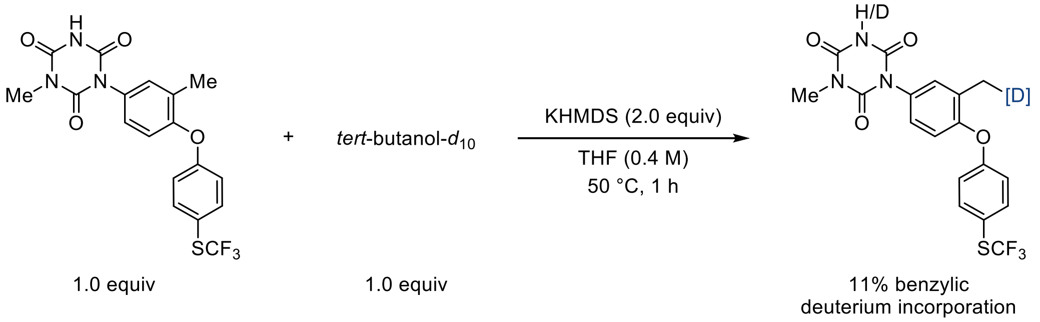

Deuterium exchange study for 1-methyl-3-(3-methyl-4-(4-((trifluoromethyl)thio)phenoxy)phenyl)-1,3,5-triazinane-2,4,6-trione: The procedure reported in Section VIa was followed using 1-methyl-3-(3-methyl-4-(4-((trifluoromethyl)thio)phenoxy)phenyl)-1,3,5-triazinane-2,4,6-trione (85.1 mg, 0.2 mmol, 1.0 equiv), *tert*-butanol-d_{10} (18.9 µL, 0.2 mmol, 1.0 equiv), KHMDS (79.8 mg, 0.4 mmol, 2.0 equiv), THF (0.5 mL, 0.4 M) at 50 °C, and the reaction solution was quenched with methanol (0.2 mL, 4.9 mmol, 25 equiv). TMB (42.3 mg, 251 µmol) was used as an internal standard to assess mass balance, with a 10 second relaxation delay used to acquire quantitative ^{1}H NMR data. An ^{1}H NMR spectrum of the crude reaction mixture with a comparison to the pure authentic starting material of toltrazuril is provided in Figures S11-12.

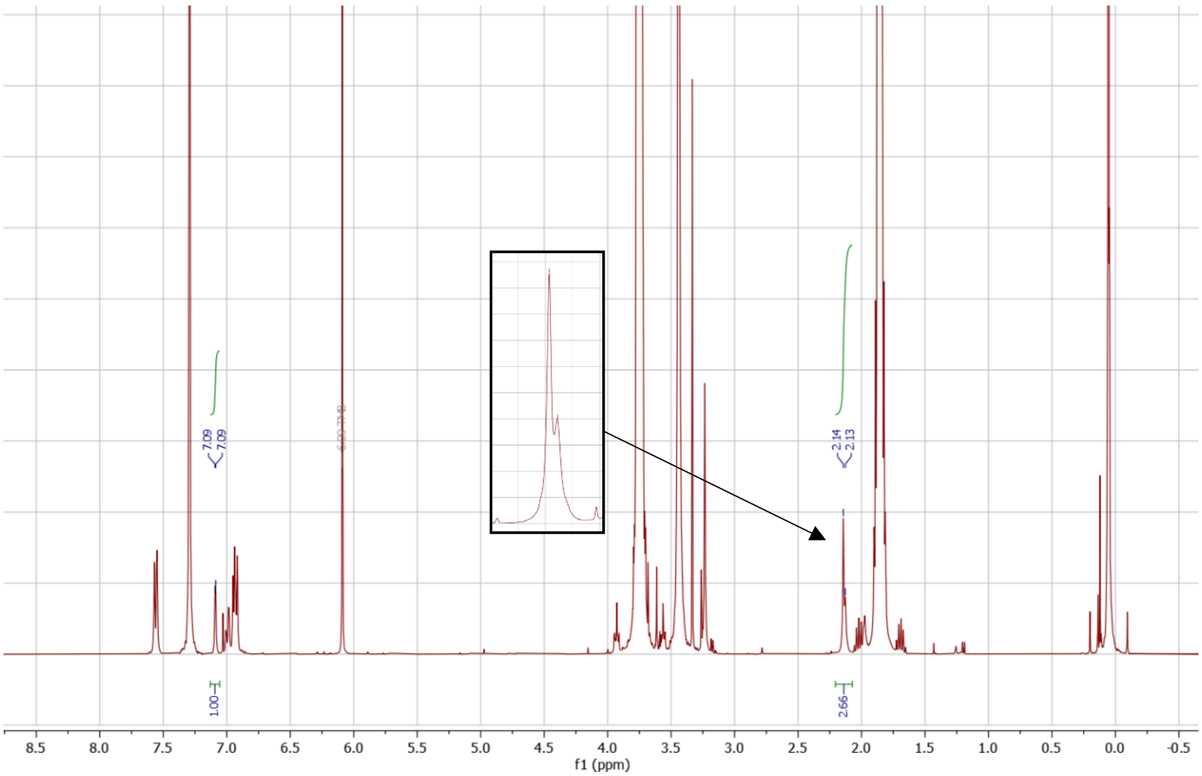

Figure S11: ^{1}H NMR spectrum for the deuterium exchange study for toltrazuril. TMB was used to assess mass balance. The aromatic signal of TMB at 6.09 ppm (s, 3H) (42.3 mg, 251 µmol) was integrated against an aromatic signal of 1-methyl-3-(3-methyl-4-(4-((trifluoromethyl)thio)phenoxy)phenyl)-1,3,5-triazinane-2,4,6-trione at 7.09 ppm (d, J = 2.4 Hz, 1H) (192 µmol, 96% recovery). The methyl signal of toltrazuril at

2.14 ppm (s, 3H) was integrated against the aromatic signal of tolrazuril at 7.09 ppm (d, $J = 2.4$ Hz, 1H) set to an integral of 1.00 to determine the percentage deuterium exchange (2.66 / 3.00 = benzylic 11% deuterium incorporation).

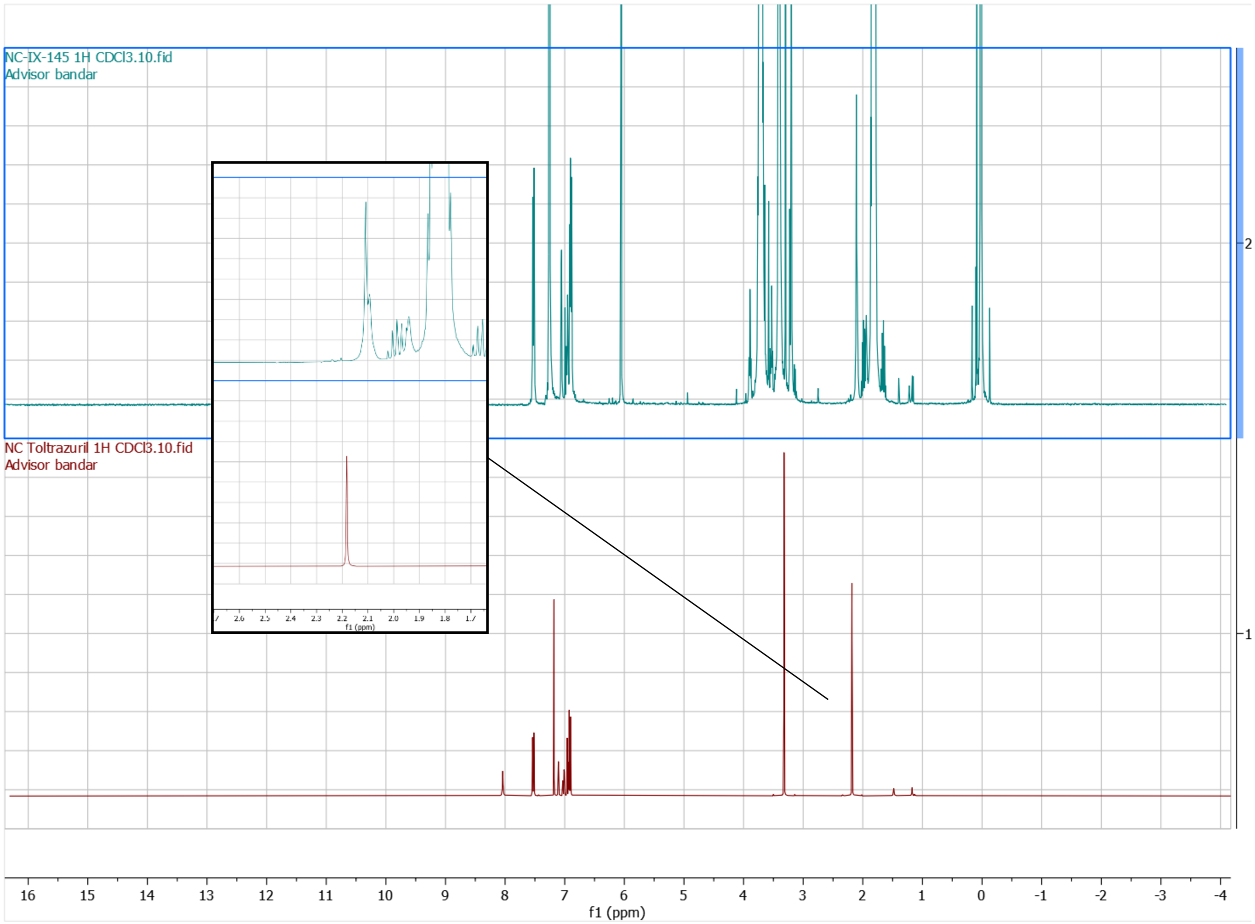

Figure S12: ^{1}H NMR spectrum of the crude reaction mixture of toltrazuril deuterium exchange experiment stacked atop an ^{1}H NMR spectrum of pure toltrazuril starting material.

Analysis: To determine the degree of deuterium incorporation the methyl signal of toltrazuril at 2.14 ppm (s, 3H) was integrated against the aromatic signal of TMB at 6.09 ppm (s, 3H) (42.3 mg, 251 µmol). The reduction of signal of toltrazuril's methyl signal from expected 0.2 mmol was used to calculate the percent deuterium exchange (169 µmol, 16% deuterium incorporation). A visible change in signal appearance for the benzylic methyl position compared to the starting material is also observed and consistent with deuterium exchange.

Benzylic deuterium incorporation

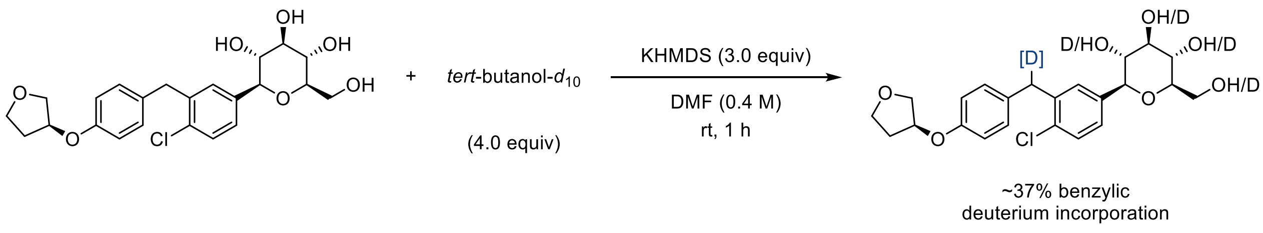

Deuterium exchange experiment with (2*S*,3*R*,4*R*,5*S*,6*R*)-2-(4-chloro-3-(4-(((*S*)-tetrahydrofuran-3-yl)oxy)benzyl)phenyl)-6-(hydroxymethyl)tetrahydro-2H-pyran-3,4,5-triol (empagliflozin): The procedure reported in Section VIa was followed using a 7.5 mL, oven dried dram vial with a stir bar, empagliflozin (225.0 mg, 0.5 mmol, 1.0 equiv), *tert*-butanol-d_{10} (188.6 µL, 2.0 mmol, 4.0 equiv), KHMDS (299.3 mg, 1.5 mmol, 3.0 equiv), and DMF (1.25 mL, 0.4 M), and the reaction solution was quenched with methanol (0.5 mL, 12.4 mmol, 25 equiv). The crude reaction mixture was transferred to a 125 mL separatory funnel with DCM (10 mL). H_2O (50 mL) was added to the separatory funnel and the mixture was extracted with DCM (3 x 25 mL) and the organic layers were combined. The resulting combined organic layers were transferred back to the funnel and the organic layers were washed with 5% aqueous LiCl solution (2 x 40 mL). The organic phase was collected, dried over Na_2SO_4, filtered, and concentrated *in vacuo*. To the concentrated mixture, *n*-heptane (50 mL) was added, and the solution was reconcentrated (this was done to further remove DMF). The resulting white solid was dissolved in DCM and transferred to a 20 mL scintillation vial and concentrated *in vacuo*. The white solid was then dissolved in DMSO-d_6 (~0.4 mL) and was transferred to an NMR tube. The solution was analyzed by NMR spectroscopy (various methods) to determine the degree of deuterium incorporation. **Note:** excess (4.0 equiv) *tert*-butanol-d_{10} was used due to the number of hydroxyl groups on empagliflozin which could also exchange with deuterium. **Note:** 18-crown-6 was omitted from this experiment for ^{1}H NMR spectral clarity as it overlaps with multiple signals. An identical experiment (not shown) with 18-crown-6 (3.0 equiv) was conducted on 0.2 mmol scale which also resulted in deuterium exchange. The change in benzylic ^{13}C NMR signal appearance as seen Figure S16 was also observed in this experiment, consistent with benzylic deuteration.

Discussion: To provide evidence for deuterium exchange at the benzylic position of empagliflozin, ^{1}H NMR spectroscopy of the crude mixture was analyzed following an aqueous workup. We found that the benzylic ^{1}H signal of empagliflozin is overlapped with another signal so the signal reduction was corroborated with ^{13}C NMR spectroscopy.

Analysis: ^{1}H NMR and HSQC spectra of pure empagliflozin are provided (Figure S13 and S14, respectively), showing that the benzylic diarylmethylene ^{1}H NMR signal is overlapped with another signal (m, 4.03 ppm). Next, a ^{1}H NMR spectrum of the crude deuteration reaction mixture is provided showing a reduction of signal integration where the benzylic methylene position is expected to appear, consistent with deuterium incorporation (Figure S15). Next, a ^{13}C NMR spectrum of the crude reaction mixture is provided showing that the diarylmethylene benzylic carbon ^{13}C signal exhibits a change in signal appearance, consistent with deuterium exchange (Figure S16). We have also compared this signal change to the ^{13}C NMR spectrum of a diphenylmethane deuterium incorporation control reaction (Figure S17). A DEPT-135 NMR spectrum of the crude reaction mixture is provided showing that the deuterated methylene carbon is not inverted while the non-deuterated empagliflozin methylene signal is inverted, consistent with deuterium exchange (Figure S18). We note that the lack of detectable deuterium exchange on the hydroxyl groups (seen in Figure S15) is likely due to proton exchange occurring from either the addition of methanol to quench the reaction mixture and / or from the aqueous workup. An identical experiment (not shown) was conducted on 0.1 mmol scale and was quenched with methanol-d_4 (0.1 mL, 2.4 mmol, 24 equiv); no aqueous workup was conducted. Deuterium exchange at the hydroxyl positions was evident *via* ^{1}H NMR spectroscopy of the crude reaction mixture.

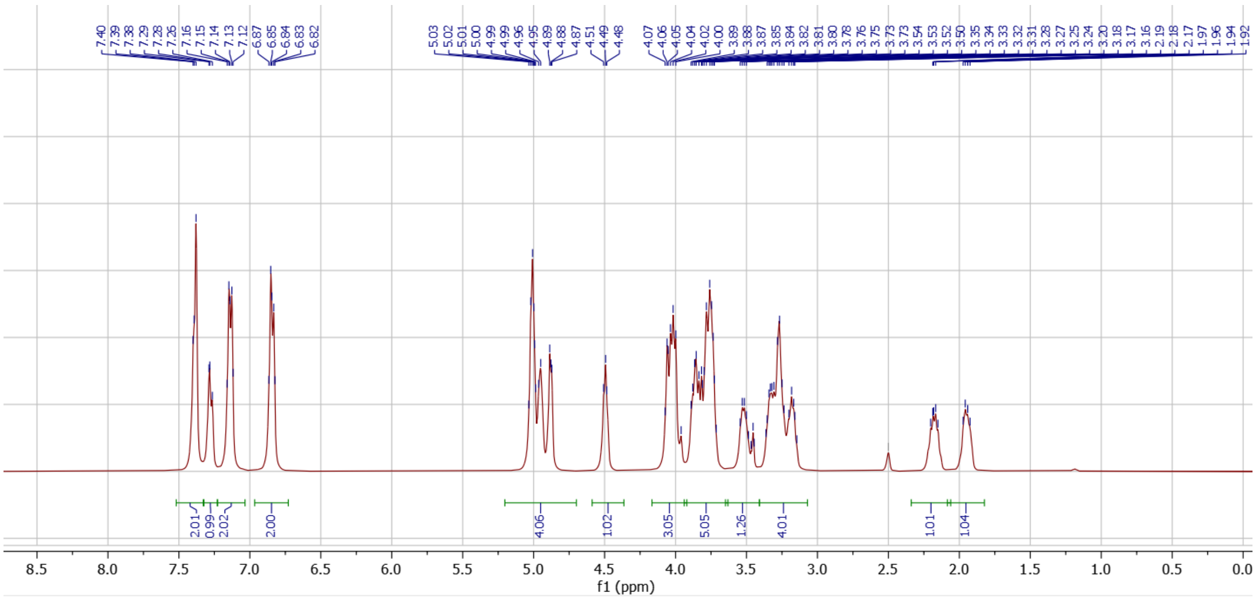

Figure S13: ^{1}H NMR spectrum of empagliflozin (CombiBlocks, #QE-8903), the methylene signal (likely s, 2H) is overlapped with the multiplet at 4.03 (m, 3H).

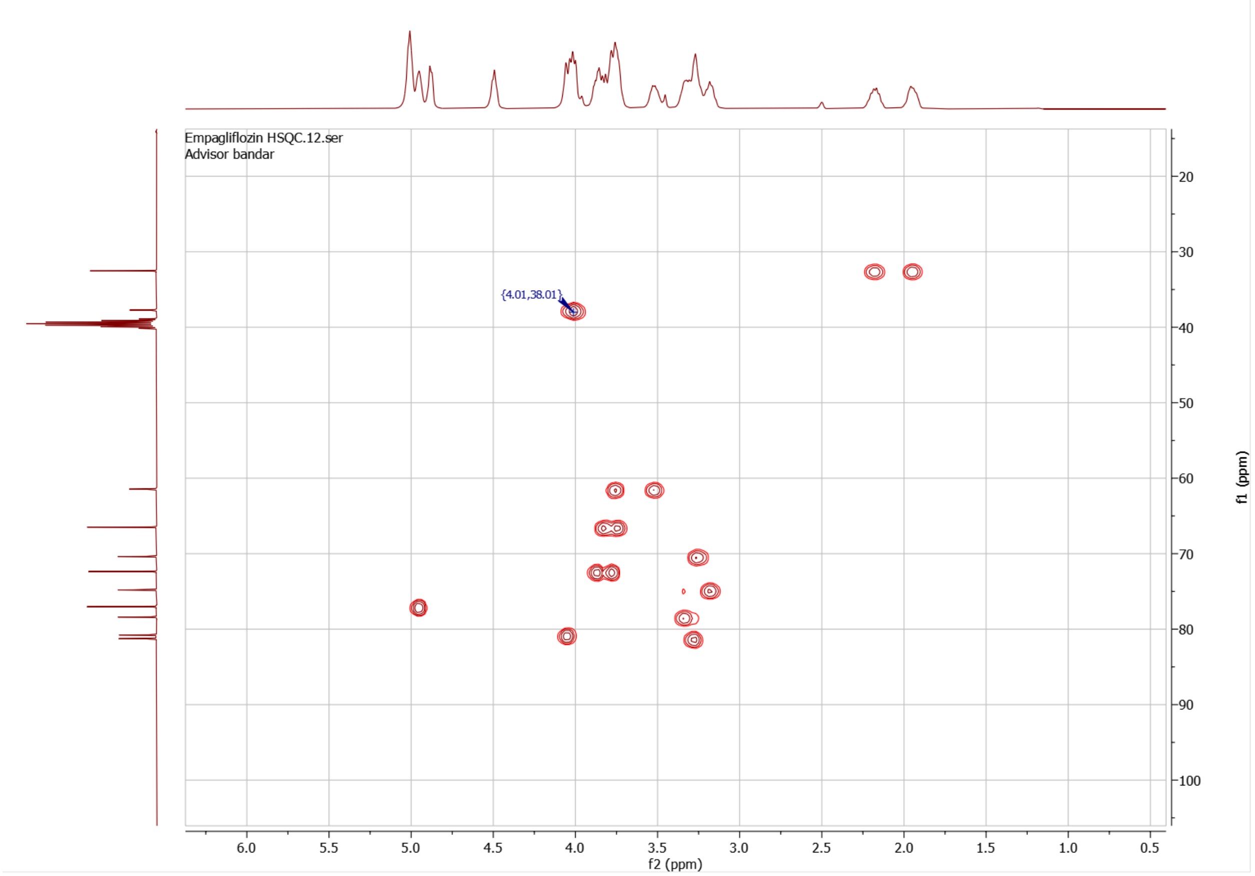

Figure S14: HSQC spectral window of pure empagliflozin authentic starting material with the benzylic methylene signal labeled. The labeled signal at (4.01 ppm, 38.01 ppm) represents the benzylic methylene

position. This spectrum shows that the benzylic methylene (likely s, 2H) is overlapped with another signal. The benzylic methylene ^{13}C NMR signal appears at 38.01 ppm.

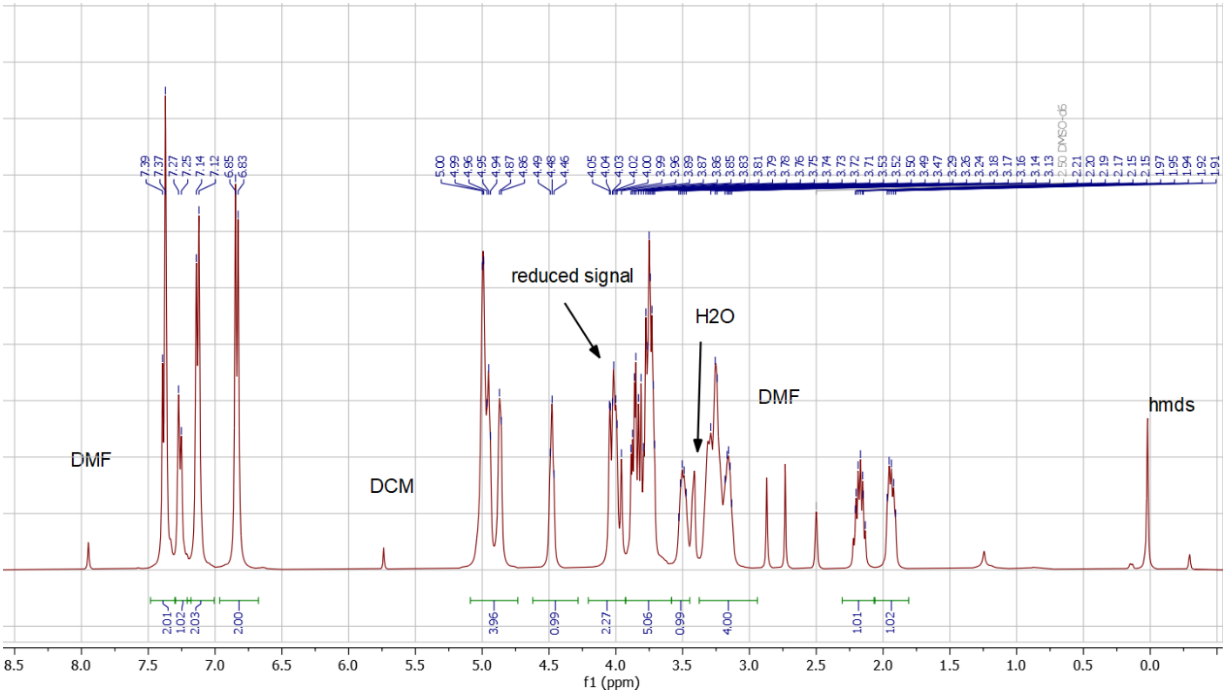

Figure S15: ^{1}H NMR spectrum of the crude reaction mixture of empagliflozin reaction with *tert*-butanol-d_{10} and KHMDS. The multiplet signal integration at 4.01 ppm is reduced from expected 3.00 (since the other signal accounts for 1 proton 1.27 / 2 = 0.635; 1 – 0.635 = 0.365 → ~37% deuterium incorporation estimated). Due to the overlapped signal, the deuterium exchange is reported as an approximate value.

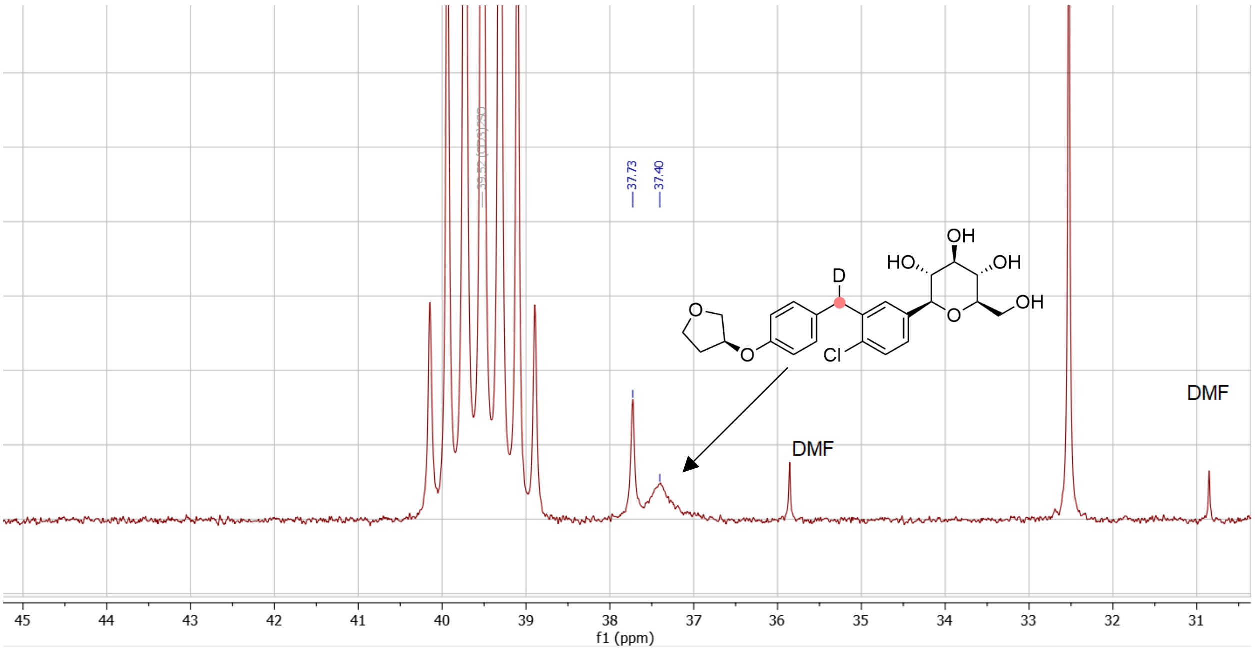

Figure S16: ^{13}C NMR spectral window of the crude reaction mixture of the reaction of empagliflozin with *tert*-butanol-d_{10} and KHMDS. The signal at 37.7 ppm represents the benzylic methylene carbon and the signal beside it (broad, 37.4 ppm) represents the deuterated methylene position.

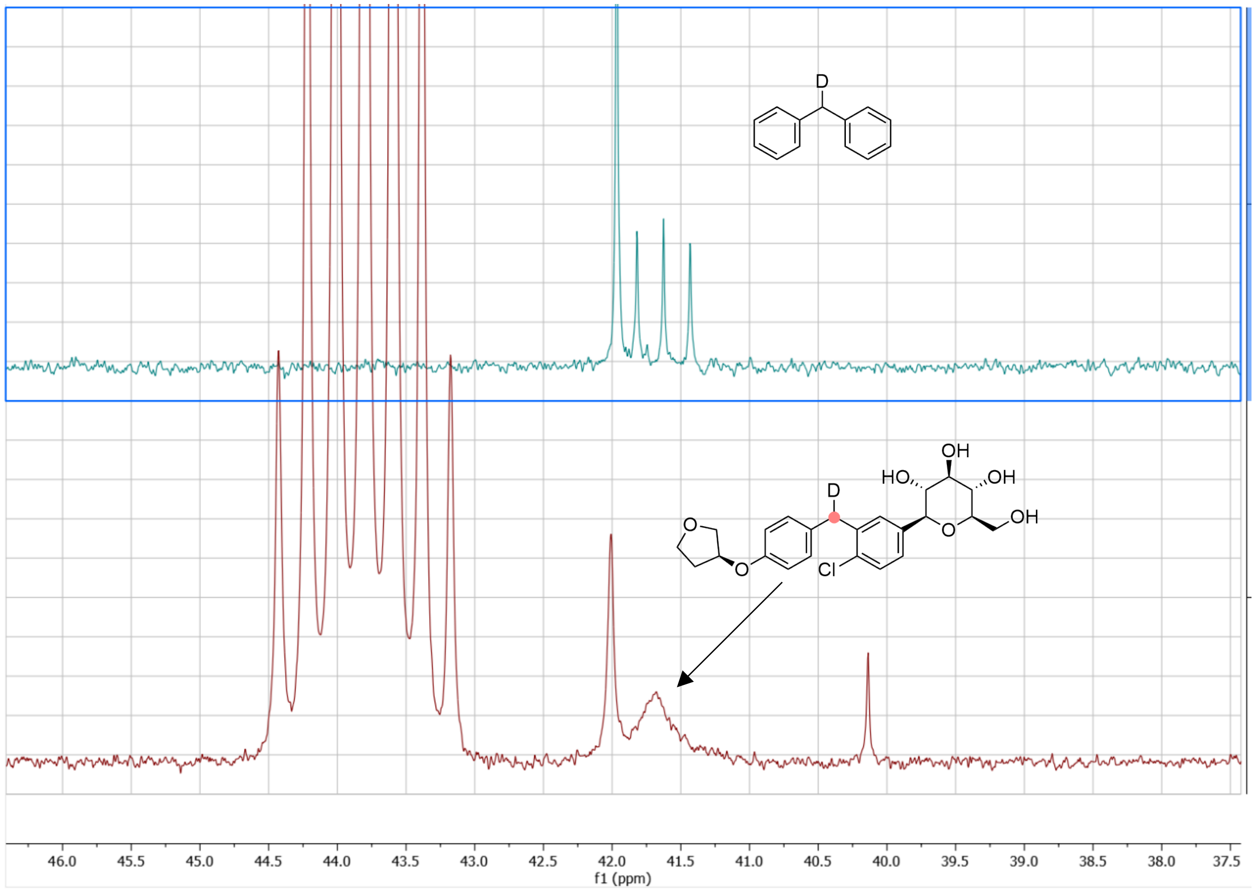

Figure S17: ^{13}C NMR spectra of crude reaction mixture of reaction of empagliflozin with *tert*-butanol-d_{10} and KHMDS (bottom, red spectrum, DMSO-d_6) and the crude reaction mixture for the deuteration of diphenylmethane (top, blue spectrum, $CDCl_3$), prepared using procedure described in Section VIa, using diphenylmethane (33.7 mg, 0.2 mmol, 1.0 equiv), KHMDS (80.0 mg, 0.4 mmol, 2.0 equiv), *tert*-butanol-d_{10} (18.8 µL, 0.2 mmol, 1.0 equiv) and THF (0.5 mL, 0.4 M)) to show resemblance. The spectra were aligned manually in order to aid visualization of how deuterium impacts ^{13}C NMR shifts, as the chemical shift for these ^{13}C signals are not the same.

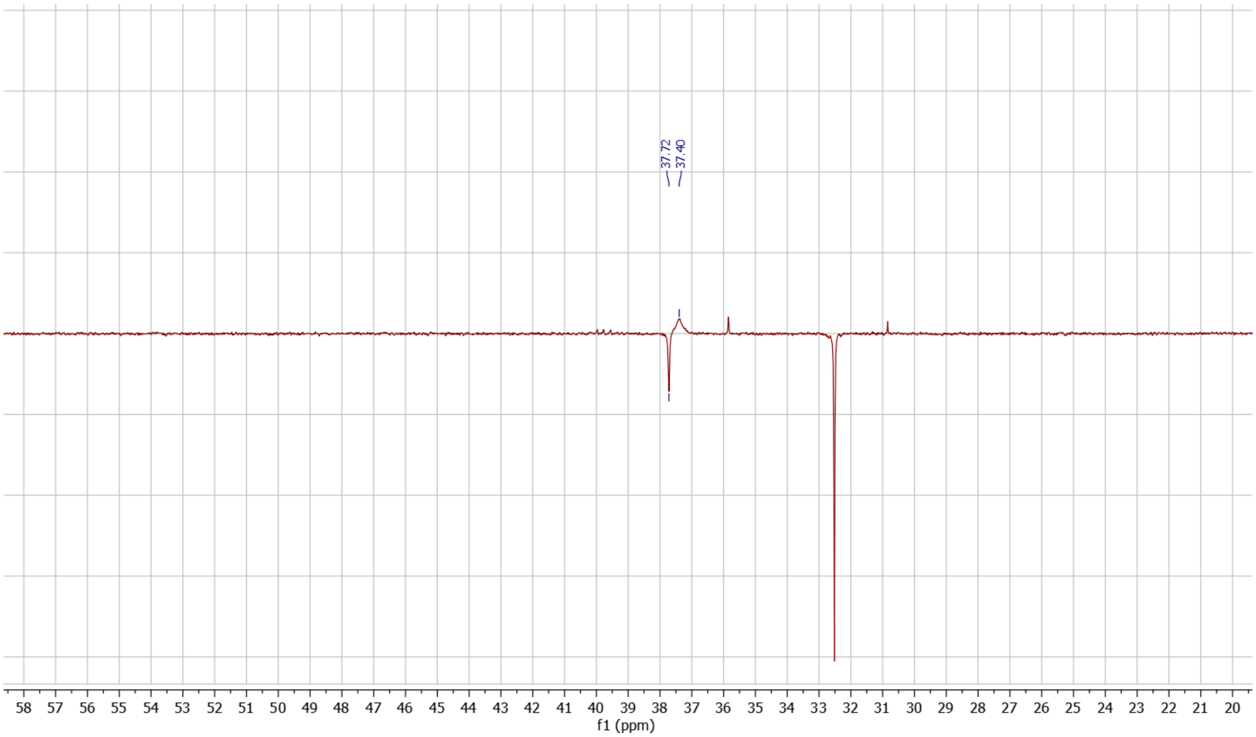

Figure S18: DEPT-135 NMR spectral window of the crude reaction mixture of the reaction of empagliflozin with *tert*-butanol-d_{10} and KHMDS displaying inverted benzylic methylene signal beside the monodeuterated methylene signal which is not inverted, consistent with deuterium exchange.

Summary: The signal intensity reduction of the benzylic position of empagliflozin in the crude ^{1}H NMR spectrum for the reaction of empagliflozin with *tert*-butanol-d_{10} and KHMDS and the change of appearance of the corresponding ^{13}C signal are consistent with deuterium incorporation. We therefore conclude that deuterium exchange takes place at the benzylic diarylmethylene position of empagliflozin when subjected to KHMDS and a deuterium source in DMF. Based on the signal reduction in the ^{1}H NMR spectrum, approximately 37% deuterium incorporation is observed. These results are consistent with benzylic deprotonation.

d) Protection from over-reaction: deuterium exchange between 1-((2,6-dichlorobenzyl)oxy)-2,2,6,6-tetramethylpiperidine (4) and a deuterium source in the presence of base.

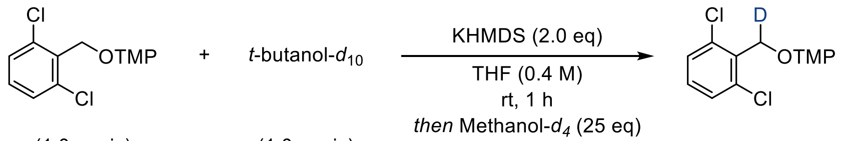

Procedure: 1-((2,6-dichlorobenzyl)oxy)-2,2,6,6-tetramethylpiperidine (**4**, 31.6 mg, 0.1 mmol, 1.0 equiv) was added *via* pipette to a 4 mL, oven-dried dram vial with a stir bar. The vial was sealed with a screwcap lined with a PTFE septum and brought into a N_2 filled glovebox. The vial was unsealed, and THF (0.25 mL, 0.4 M) was added *via* micropipette. *Tert*-butanol-d_{10} (9.4 µL, 0.1 mmol, 1.0 equiv) was then added *via* microsyringe followed by KHMDS (39.9 mg, 0.2 mmol, 2.0 equiv). The vial was removed from the glovebox and the reaction mixture was stirred at rt for 1 hour. At this time, the vial was unsealed and methanol-d_4 was added *via* syringe to quench the reaction mixture. TMB (29.5 mg, 175 µmol) was then added to the quenched crude reaction mixture. A small aliquot from the crude reaction mixture was removed, charged into an NMR tube, and constituted in $CDCl_3$ (0.5 mL). ^{1}H NMR spectroscopy (400 MHz, $CDCl_3$) was used to determine the degree of deuterium incorporation. A 10 second relaxation delay was used to acquire quantitative ^{1}H NMR data.

Analysis: The benzylic methylene signal at 5.06 ppm (s, 2H) (98.9 µmol, 99% recovery) was integrated against the aromatic signal of TMB at 6.09 ppm (s, 3H) (29.5 mg, 175 µmol) to the calculate the percentage of deuterium incorporation. A crude recovery of 99% indicates that no significant deuterium exchange occurs at the benzylic position, suggesting that deprotonation does not readily occur after functionalization.

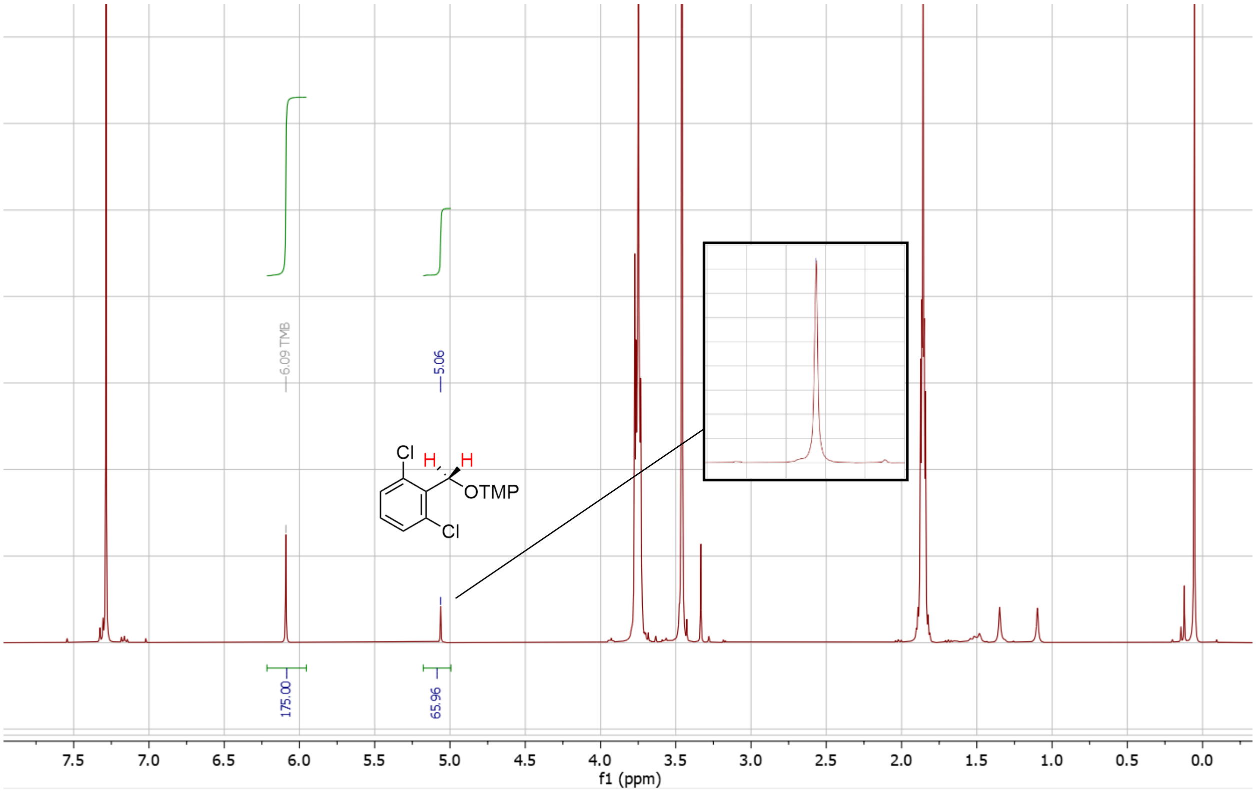

Figure S19: ^{1}H NMR spectrum for deuterium exchange experiment with 1-((2,6-dichlorobenzyl)oxy)-

2,2,6,6-tetramethylpiperidine (**4**). 1-((2,6-Dichlorobenzyl)oxy)-2,2,6,6-tetramethylpiperidine methylene signal at 5.06 ppm (98.9 µmol, 99% recovery) integrated against TMB.

e) Observations consistent with TEMPO serving as a single electron oxidant.

Discussion: during this work we made several observations consistent with TEMPO serving as a single electron oxidant. First, at no point is a greater than 50% yield of TEMPO functionalized alkylarene product observed when 1 equivalent of TEMPO is used (See Table S1-S3); this suggests the stoichiometry of TEMPO with respect to alkylarene is 2:1. Additionally, we observe the mass of 2,2,6,6-tetramethylpiperidin-1-ol (TEMPOH) *via* mass spectrometry in numerous crude reaction mixtures. Furthermore, a color change of the reaction mixture can be observed during the course of the reaction as TEMPO is consumed from orange (color of TEMPO) to colorless (color of TEMPOH)[9] (*vide infa*). These observations are consistent with TEMPO as the oxidant and prior literature of its use as a single electron oxidant for organometallic reagents (see refs 20-21 in the manuscript).

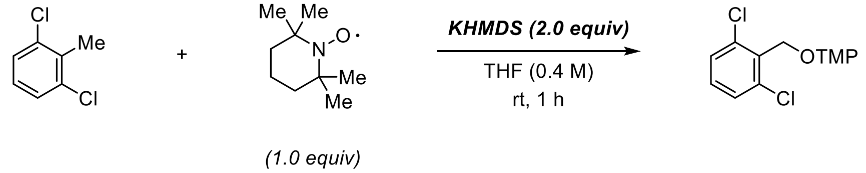

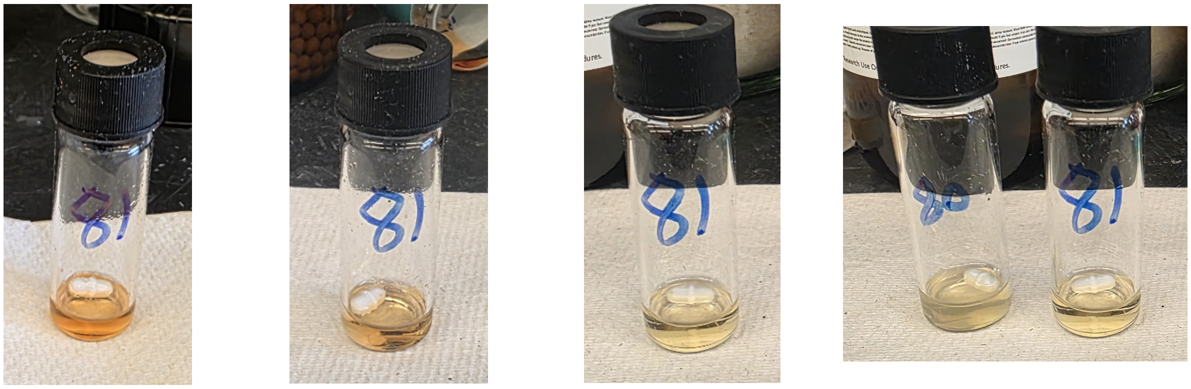

T = 1 min | T = 20 min | After quench with MeOH (1 h) | Beside identical reaction with no TEMPO

Figure S20: Qualitative color change assessment of a reaction that uses 1 equiv of TEMPO. The color change (from orange to colorless) is consistent with TEMPO consumption.

f) Evidence for TEMPO serving as the active oxidant: controls using TEMPO oxoammonium salt.

Discussion: Under acidic conditions, TEMPO disproportionates into 2,2,6,6-tetramethyl-1-oxopiperidin-1-ium ($TEMPO^+$) and 2,2,6,6-tetramethylpiperidin-1-ol (TEMPO-H).[10] To assess if 2,2,6,6-tetramethyl-1-oxopiperidin-1-ium ($TEMPO^+$) forms *in situ* under the basic reaction conditions and is serving as an active oxidant, we conducted experiments using 2,2,6,6-tetramethyl-1-oxopiperidin-1-ium tetrafluoroborate ($TEMPO^+BF_4^-$). $TEMPO^+BF_4^-$ was prepared according to a previous report.[11] Two experiments were conducted: 1) use of 10 mol% $TEMPO^+BF_4^-$ and 90 mol% TEMPO to mimic disproportionation, and 2) use of 2.5 equiv $TEMPO^+BF_4^-$ to demonstrate reactivity with exclusively 2,2,6,6-tetramethyl-1-oxopiperidin-1-ium in place of TEMPO.

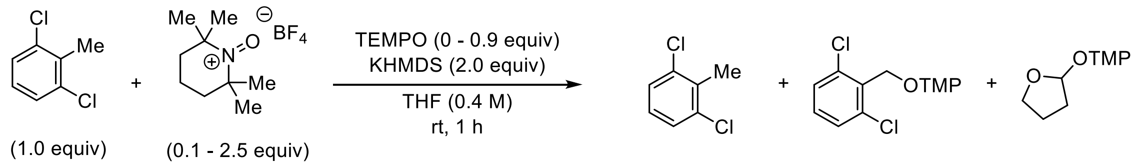

Procedure: 1,3-Dichloro-2-methylbenzene (16.1 mg, 0.1 mmol, 1.0 equiv) was added *via* pipette to a 4 mL, oven-dried dram vial with a stir bar. The vial was sealed with a screwcap lined with a PTFE septum and brought into a N_2 filled glovebox. The vial was unsealed, and THF (0.25 mL, 0.4 M) was added *via* micropipette. 2,2,6,6-Tetramethyl-1-oxopiperidin-1-ium tetrafluoroborate (if necessary, 2.4 mg or 60.8, 0.01 or 0.25 mmol, 0.1 or 2.5 equiv) was added followed by TEMPO (if necessary, 14.1 mg, 0.09 mmol, 0.9 equiv). KHMDS (39.9 mg, 0.2 mmol, 2.0 equiv) was added. The vial was sealed, removed from the glovebox and the reaction mixture was stirred at rt for 1 hour. At this time, the vial was unsealed and methanol (0.1 mL, 2.47 mmol, 24.7 equiv) was added *via* syringe. NMR sample preparation and analysis was conducted according to **General Procedure for Condition Variation**. **Note:** the yield of 2,2,6,6-tetramethyl-1-((tetrahydrofuran-2-yl)oxy)piperidine was calculated with respect to 0.1 mmol (i.e., a possible yield of 200% for 2,2,6,6-tetramethyl-1-((tetrahydrofuran-2-yl)oxy)piperidine is possible for Reaction 2 with KHMDS as the limiting reagent).

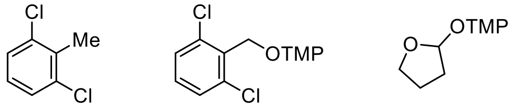

		**2**	**4**	
**(reaction 1)**	**0.1 equiv TEMPO⁺BF₄⁻**<br>**0.9 equiv TEMPO**	47%	46%	4%
**(reaction 2)**	**2.5 equiv TEMPO⁺BF₄⁻**<br>**no TEMPO**	91%	2%	72%

Analysis: A mixture of 10 mol% $TEMPO^+BF_4^-$ and 90 mol% TEMPO under otherwise standard conditions results in a mixture of **4** and 2,2,6,6-tetramethyl-1-((tetrahydrofuran-2-yl)oxy)piperidine (see reference 5 in the **References** section for details and characterization). The 46% of **4** yield observed in reaction 1 is consistent with TEMPO serving as a single electron oxidant as a maximum yield with 0.9 equiv of TEMPO is 45%. In contrast, the 4% of 2,2,6,6-tetramethyl-1-((tetrahydrofuran-2-yl)oxy)piperidine formed is likely promoted by the 10 mol% $TEMPO^+BF_4^-$. During this work, in no case was 2,2,6,6-tetramethyl-1-((tetrahydrofuran-2-yl)oxy)piperidine observed when using HMDS bases and TEMPO alone. Using 2.5 equiv $TEMPO^+BF_4^-$ with no TEMPO, a small amount (2%) of **4** is formed with the major product being 2,2,6,6-tetramethyl-1-((tetrahydrofuran-2-yl)oxy)piperidine. These results are inconsistent with $TEMPO^+$ serving as an active oxidant when using basic Conditions A-C reported in this work. A spectrum for Reaction **2** with 2.5 equivalence of $TEMPO^+BF_4^-$ is provided in Figure S21.

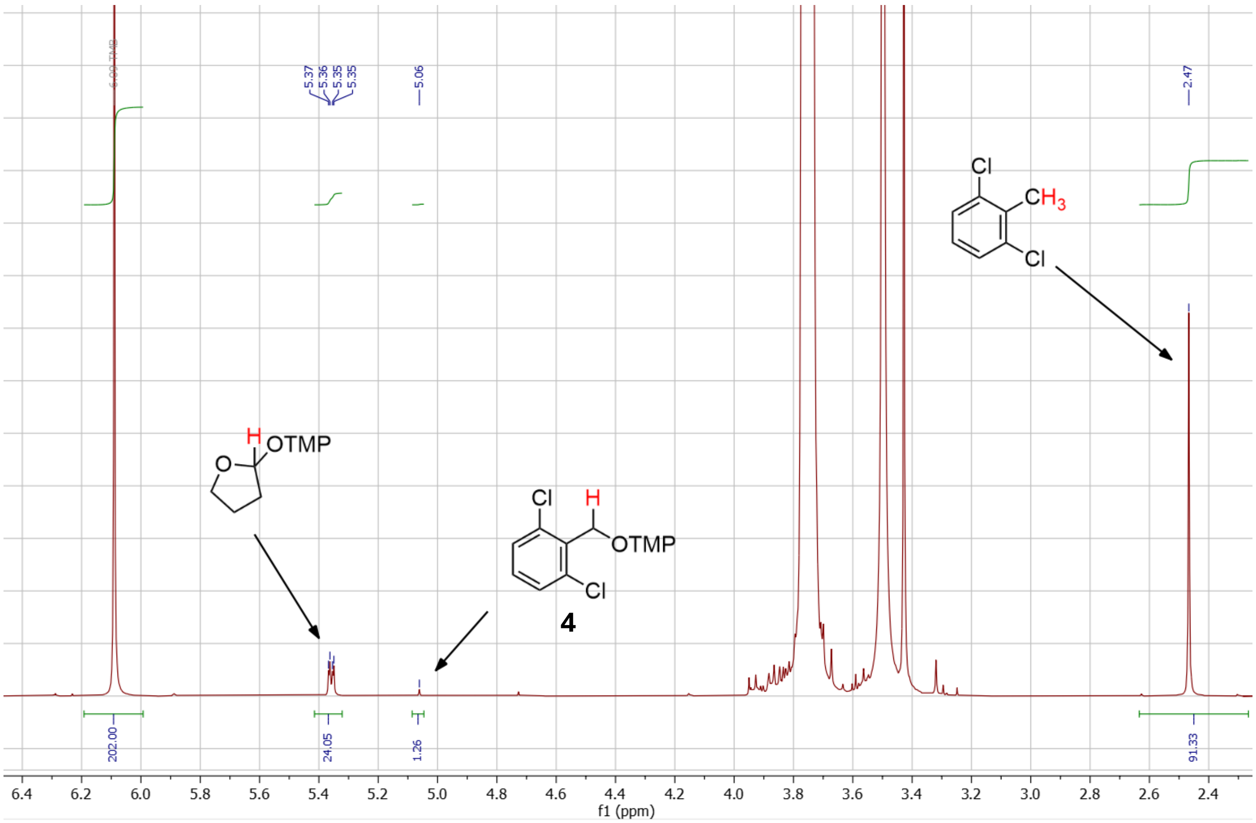

Figure S21: ^{1}H NMR spectral window of the reaction of 1,3-dichloro-2-methylbenzene (1.0 equiv) with $TEMPO^{+}BF_4^{-}$ (2.5 equiv) and KHMDS (2.0 equiv) in THF. The aromatic signal of TMB at 6.09 (s, 3H) (34.0 mg, 202 µmol) was integrated against the methine proton of 2,2,6,6-tetramethyl-1-((tetrahydrofuran-2-yl)oxy)piperidine at 5.36 ppm (dd, J = 5.4, 1.9 Hz) (72 µmol, 72% yield) and the methylene protons of **4** at 5.06 ppm (2 µmol, 2% yield).

g) Evidence for TEMPO serving as the active oxidant: site-selectivity for polyalkylarenes.

Discussion: To provide additional assessment on whether $TEMPO^{+}$ is an active oxidant in this system, we designed an experiment to contrast selectivity using $TEMPO^{+}BF_4^{-}$ or TEMPO as the oxidant. Two sets of reactions were carried out using 5-ethyl-2-methylpyridine and 1-ethyl-4-(*p*-tolylethynyl)benzene. 1-Ethyl-4-(*p*-tolylethynyl)benzene (**40**) is notable as the selectivity can be switched from methyl to ethyl by the addition of 18-crown-6 under otherwise standard conditions (Scheme 1 in manuscript). First, it is important to note that this comparison cannot be conducted in THF because the use of $TEMPO^{+}BF_4^{-}$ results in THF functionalization, which in itself is evidence that $TEMPO^{+}$ is not the active oxidant under the basic conditions with TEMPO. We therefore used aromatic solvents for this comparison. In both cases, the use of $TEMPO^{+}BF_4^{-}$ as the oxidant gives different (and poorer) selectivity than the use of TEMPO. For 1-ethyl-4-(p-tolylethynyl)benzene (**40**), the TEMPO reaction was conducted in THF to show switchable selectivity and the $TEMPO^{+}BF_4^{-}$ reaction was conducted in benzene. A control reaction (0.1 mmol scale, not shown) of **40** using standard conditions described in 1° selective functionalization of **40** (pg S26) using benzene as a solvent gives 18% yield of the 1° isomer (> 20 : 1 selectivity, by ^{1}H NMR spectroscopy) after 1 hour of reaction.

Selectivity comparison for 5-ethyl-2-methylpyridine

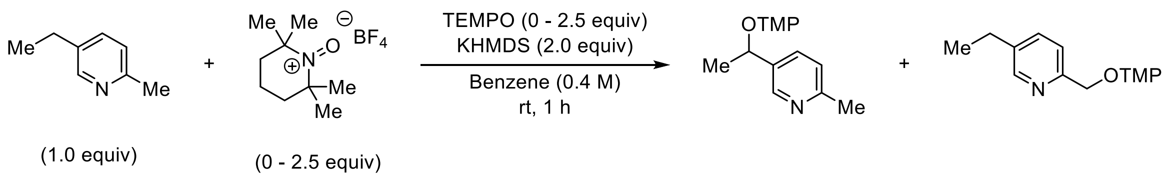

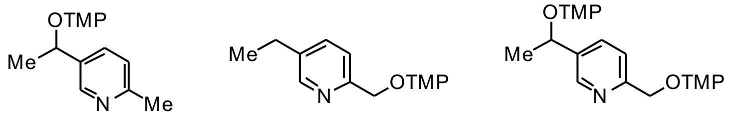

2.5 equiv TEMPO$^+$BF$_4^-$ no TEMPO	10%	14%	8%
no TEMPO$^+$BF$_4^-$ 2.5 equiv TEMPO	0%	82%	0%

Procedure for the selectivity comparison for 5-ethyl-2-methylpyridine: The procedure described in Section VIf was followed using 5-ethyl-2-methylpyridine (12.1 mg, 0.1 mmol, 1.0 equiv) instead of 1,3-dichloro-2-methylbenzene, TEMPO (if necessary, 39.1 mg, 0.25 mmol, 2.5 equiv), 2,6,6-tetramethyl-1-oxopiperidin-1-ium tetrafluoroborate (if necessary, 60.8, 0.25 mmol, 2.5 equiv) and benzene (0.25 mL, 0.4 M) instead of THF. The results are summarized above along with representative NMR spectra (Figure S22).

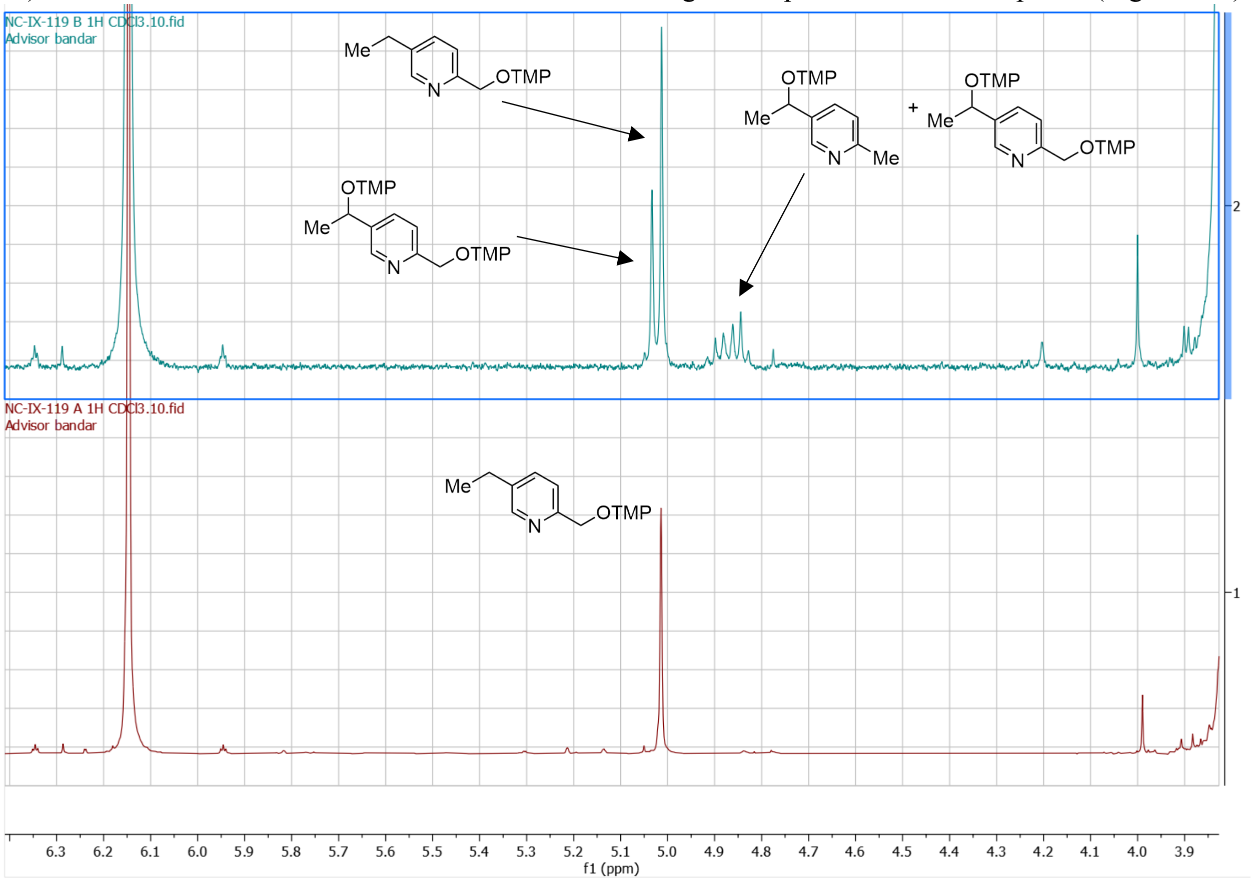

Figure S22: Stacked ^{1}H NMR spectra of the crude reaction mixtures for the reaction of 5-ethyl-2-methylpyridine with KHMDS and TEMPO$^+$BF$_4^-$ (top, blue spectrum) or TEMPO (bottom, red spectrum).

Analysis: Using $TEMPO^+BF_4^-$ as an oxidant under otherwise standard conditions in benzene, a mixture of isomers is observed, while using TEMPO as an oxidant affords a single isomer at the more acidic benzylic position. These results are inconsistent with $TEMPO^+BF_4^-$ serving as an active oxidant when using basic Conditions A-C and TEMPO in this work.

Selectivity assessment for 1-ethyl-4-(p-tolylethynyl)benzene

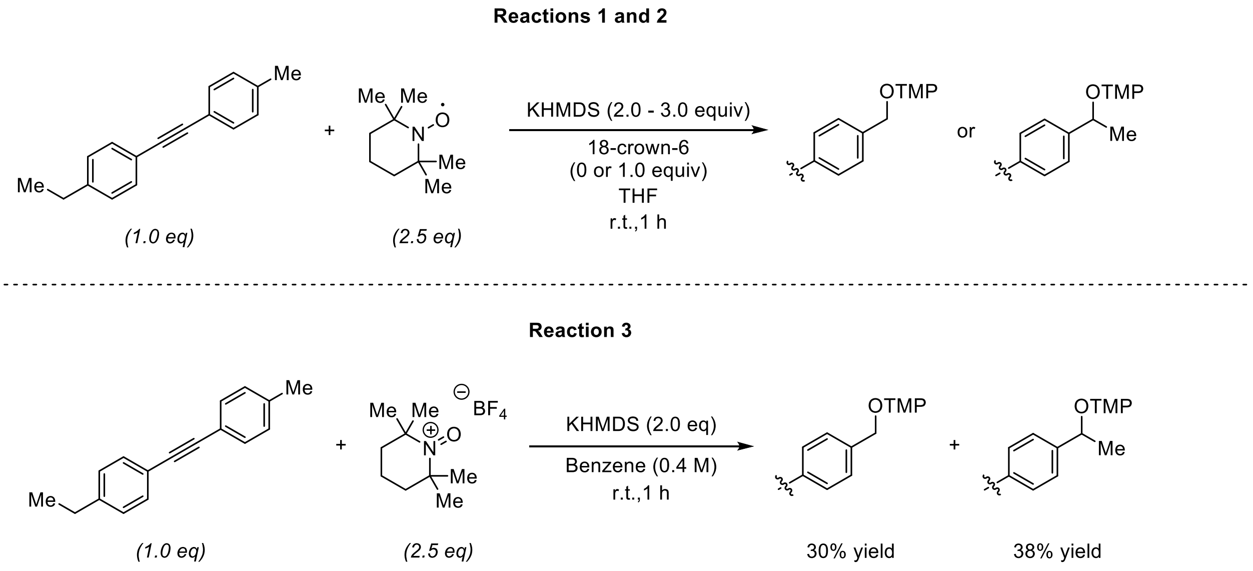

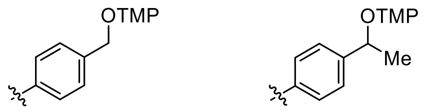

(reaction 1)	TEMPO<br>2.0 equiv KHMDS<br>No 18-crown-6	78%	22%
(reaction 2)	TEMPO<br>3.0 equiv KHMDS<br>1.0 equiv 18-crown-6	Not Detected	90%
(reaction 3)	$TEMPO^+BF_4^-$	30%	38%

Figure S23: Results for the reaction of 1-ethyl-4-(p-tolylethynyl)benzene and base with TEMPO or $TEMPO^+BF_4^-$. Percentage yields for reaction 1 and 2 were normalized to 100% mass balance.

Procedure: For Reactions 1 and 2 see pg S26 compound **40** for procedures. For Reaction 3 the procedure described in section VIf was followed using 1-ethyl-4-(p-tolylethynyl)benzene (22.0 mg, 0.1 mmol, 1.0 equiv), $TEMPO^+BF_4^-$ (60.8 mg, 0.25 mmol, 2.5 equiv), KHMDS (39.9 mg, 0.2 mmol, 2.0 equiv), and benzene (0.25 mL, 0.4 M).

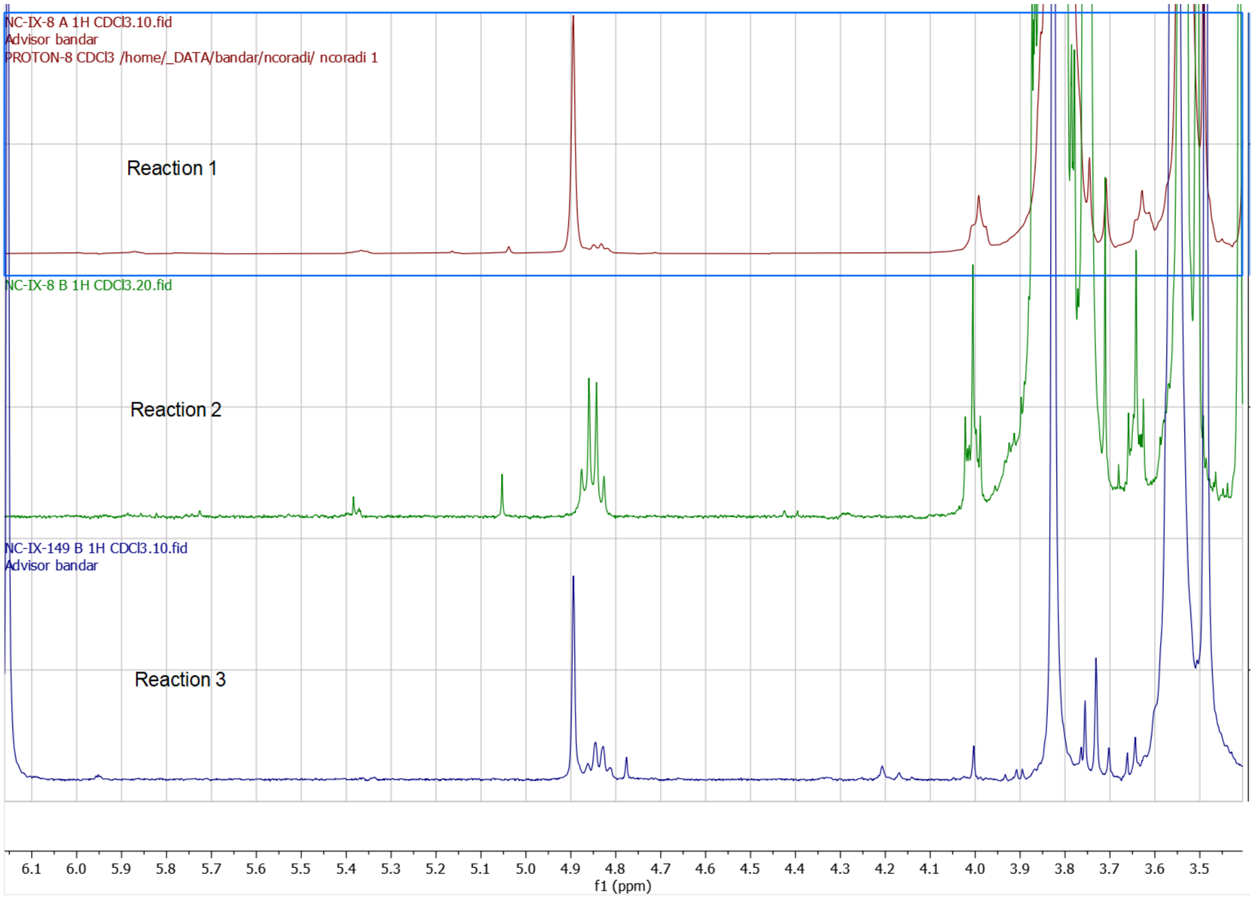

Figure S24: ^{1}H NMR spectra stacked for the selectivity of 1-ethyl-4-(*p*-tolylethynyl)benzene experiment using TEMPO or $TEMPO^+BF_4^-$ as an oxidant. The singlet signal (4.77 ppm, 2H) represents the 1° functionalized product and the quartet signal (4.72 ppm, J = 6.7 Hz) represents the 2° functionalized product.

Analysis: The reaction of 1-ethyl-4-(p-tolylethynyl)benzene under basic conditions B or C allows for switchable and good selectivity, while the use of $TEMPO^+BF_4^-$ as an oxidant under otherwise standard conditions gives poor selectivity, resulting in a mixture of isomers. These results are consistent with TEMPO serving as the active oxidant and not $TEMPO^+$ for this base-promoted method.

h) Evidence for TEMPO serving as a single electron oxidant: control reaction using a tertiary alcohol for potential β-scission.

Discussion: Lin and coworkers reported the oxidative functionalization of tertiary alcohols *via* a deprotonation-oxidation mechanism using 2,2,6,6-tetramethyl-1-oxo-piperidinium hexafluorophosphate ($TEMPO^+PF_6^-$) and KHMDS.[12] To provide additional evidence that $TEMPO^+$ is not an active oxidant in the basic conditions of this work, we conducted a control reaction by subjecting 1-phenylcyclohexan-1-ol to Conditions B (see Table S2). We reasoned that if an oxoammonium is present, scission products would be detected that are consistent with Lin's reported method.

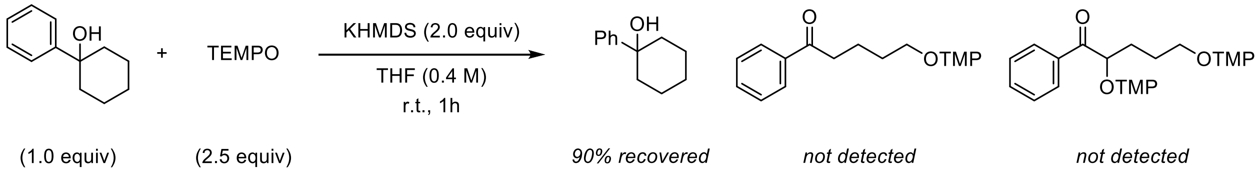

Procedure: General procedure for condition variation was followed using 1-phenylcyclohexan-1-ol (in place of alkylarene) (17.6 mg, 0.1 mmol, 1.0 equiv), TEMPO (39.1 mg, 0.25 mmol, 2.5 equiv), KHMDS (39.9 mg, 0.2 mmol, 2.0 equiv), and THF (0.25 mL, 0.4 M). The crude reaction mixture was concentrated *in vacuo*. To the concentrated mixture, $CDCl_3$ (0.5 mL) was added. Two (~0.2 mL) aliquots were removed and loaded into NMR tubes and constituted in $CDCl_3$. 1-Phenylcyclohexan-1-ol (~2 mg) was added to one of the tubes. The resulting crude reaction mixtures were analyzed by ^{1}H NMR spectroscopy and no ring opened products were detected. The aromatic signal of TMB at 6.09 ppm (s, 3H) was integrated against an aromatic signal of 1-phenylcyclohexan-1-ol (δ 7.52 (d, J = 7.3 Hz, 2H, 90 µmol 90% recovery) to assess mass balance. The ^{1}H NMR spectra of the crude reaction mixture and overlay of crude reaction mixture with 1-phenylcyclohexan-1-ol spiked in are provided in Figures S25-26.

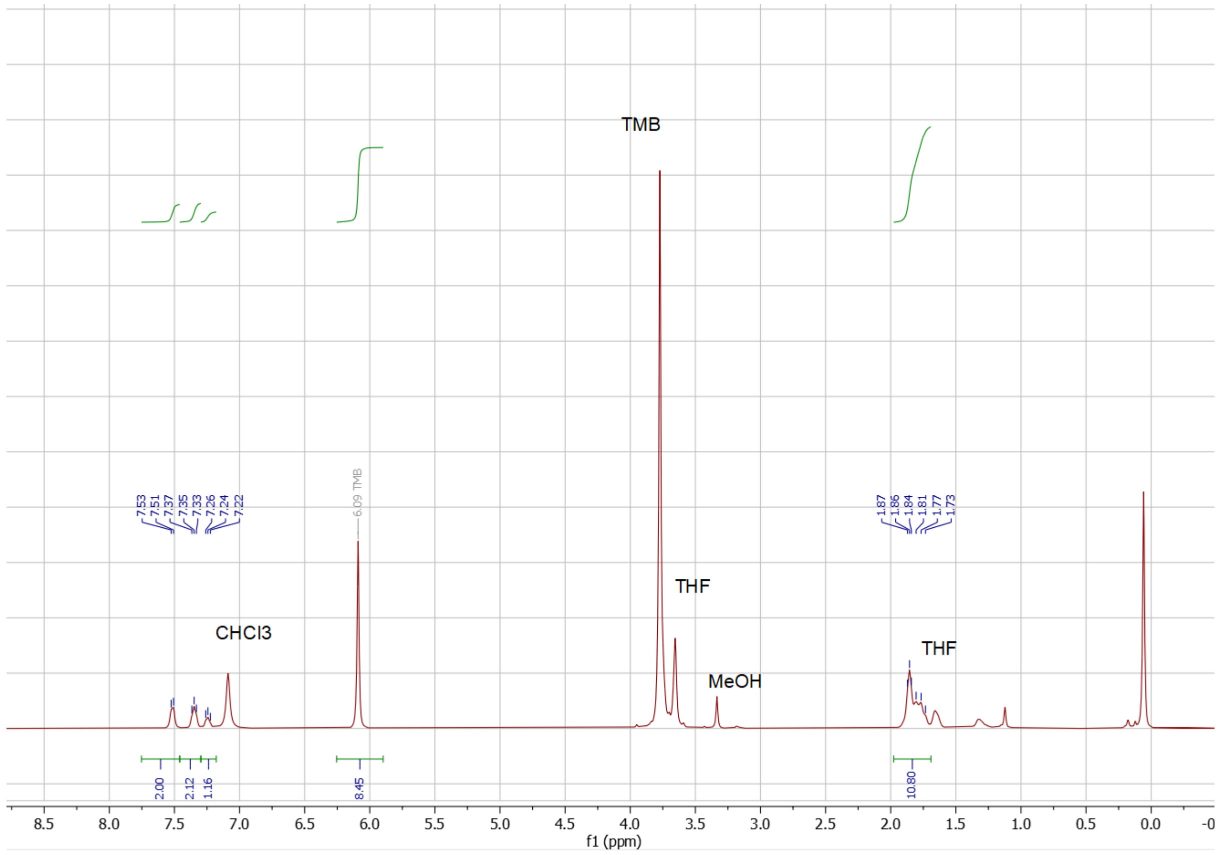

Figure S25: ^{1}H NMR spectrum of the concentrated crude reaction mixture of 1-phenylcyclohexan-1-ol subjected to basic Condition B.

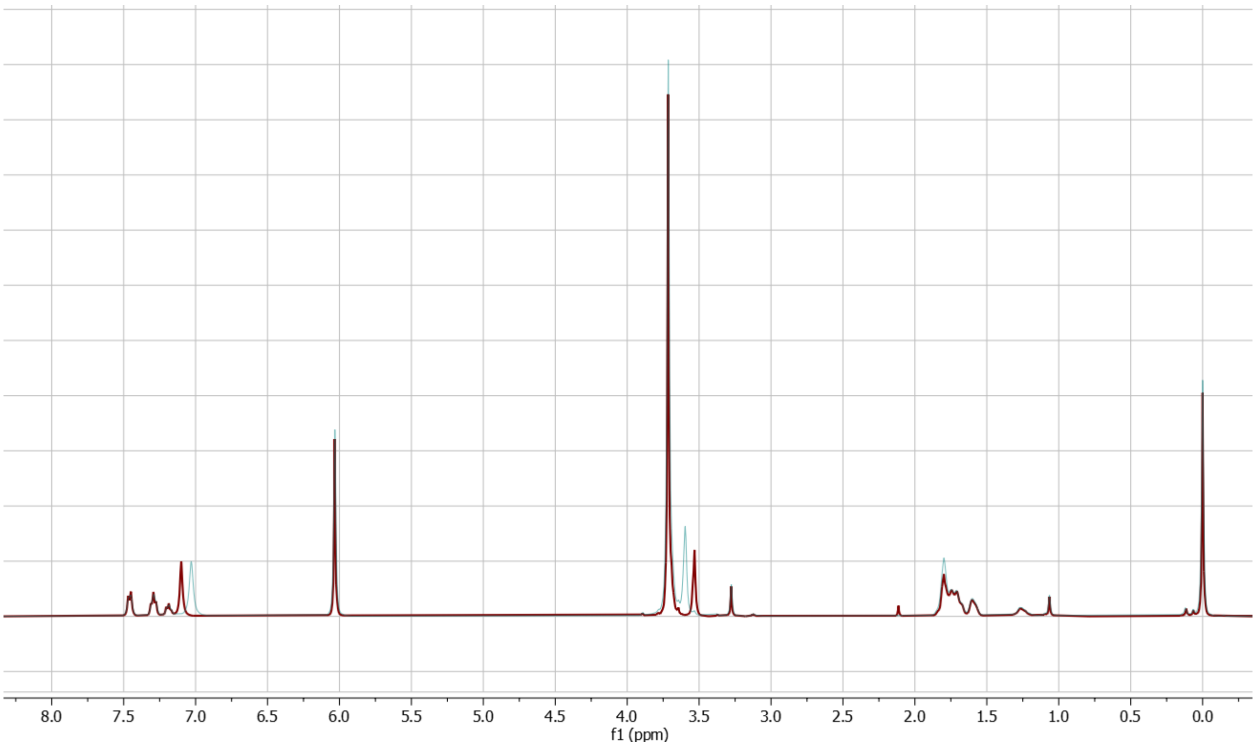

Figure S26: Overlayed ^1H NMR spectra of the crude the reaction mixture (Figure S26, blue spectrum) overlayed with crude reaction with 1-phenylcyclohexan-1-ol starting material spiked in (red spectrum).

Analysis: No discernible ring-opening products are observable by ^1H NMR spectroscopy when 1-phenylcyclohexan-1-ol is subjected to basic Condition B. Furthermore, a lack of TEMPOH signals is consistent with no redox occurring (TEMPO ^1H NMR signals are broad / difficult to detect, TEMPOH signals are easily detectable). We therefore conclude that it is unlikely that $TEMPO^+$ is forming *in situ* and serving as a single electron oxidant under these basic reaction conditions.

i) Reaction conducted with rigorous oxygen exclusion (sparging solvent with N_2).

Discussion: To provide evidence whether oxygen promotes or initiates this reaction, experiments where the reaction was conducted after oxygen was rigorously excluded were conducted. We reasoned that if oxygen played a role in the reaction, a discrepancy in reaction yield for model alkylarenes **1-3** would be apparent when oxygen was rigorously excluded.

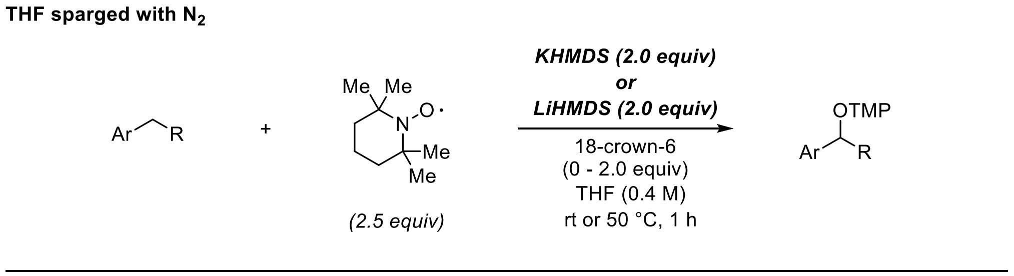

Procedure: A 250 mL, oven-dried round bottom flask was sealed with a rubber septum and was evacuated and backfilled with N_2 gas three times *via* needle connected to a Schlenk line and left under a positive pressure of N_2 gas. The flask was charged with THF (40 mL) which was deoxygenated and dried by passage

over packed columns of neutral alumina and copper (II) oxide under positive pressure of N_2. The flask was placed in a sonicator (ultrasonic cleaner) bath. Using an inlet needle and a vent needle, the THF was sparged under sonication for 15 minutes. At this time, the needles were removed and the headspace of the flask was evacuated using a vacuum line connected to a Schlenk line *via* a needle and the rubber septum was wrapped in parafilm. This was done so the flask could be safely brought into the glovebox which required placing the flask under vacuum. The flask was then brought into the glovebox. NMR sample preparation and analysis was conducted according to **General Procedure for Condition Variation**.

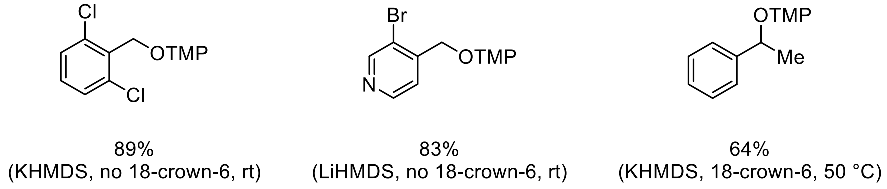

Figure S27: Results from rigorous oxygen exclusion experiment where the THF solvent was sparged under sonication for 15 minutes.

Analysis: There is little change in yield from results shown in Table S1-S3, suggesting that oxygen does not initiate or promote this reaction.

j) Reaction conducted with rigorous oxygen exclusion (freeze-pump-thaw solvent (FPT)):

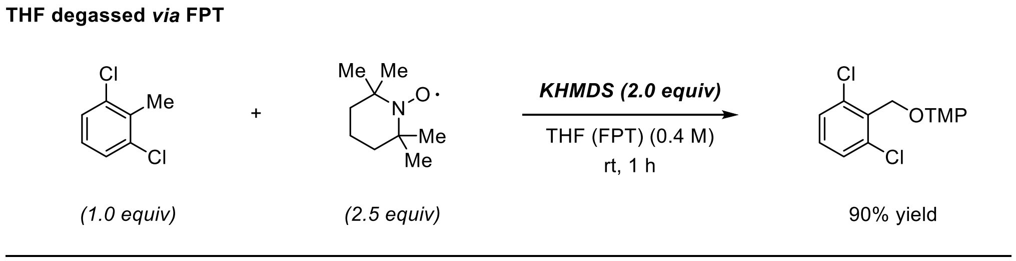

Procedure: A 250 mL Schlenk flask was flame dried and placed in an oven to cool to 120 °C. The flask was removed from the oven and was sealed with a glass stopper with vacuum grease. The flask was connected to a Schlenk line manifold *via* the sidearm, the stopcock was opened, the flask was evacuated and allowed cool under vacuum. Once cooled to rt, the flask was backfilled with N_2 gas and the glass stopper was removed. With a syringe, the flask was then charged with THF (40 mL) which was deoxygenated and dried by passage over packed columns of neutral alumina and copper (II) oxide under positive pressure of N_2. The flask was sealed with a rubber septum, and the flask was evacuated and backfilled three times with N_2 gas and left under a positive pressure of N_2 gas. The stopcock of the Schlenk flask was then closed and the flask was placed in a liquid N_2 bath. Once the THF was visibly frozen, the flask was evacuated and the THF solid was left under vacuum for 15 minutes. At this time, the stopcock was closed, the flask was removed from the liquid N_2 bath and the THF was allowed to thaw. Once the THF had melted the flask was backfilled with N_2 gas, the stopcock of the Schlenk flask was closed and the freeze-thaw process was repeated twice more. After the final thawing cycle, the THF (20 mL) was taken up into a syringe and charged into a 50 mL, flame-dried round bottom flask which had been fitted with a rubber septum and had been evacuated and backfilled three times with N_2 gas and left under a positive pressure of N_2 gas *via* a needle. The headspace of the round bottom flask was evacuated using a vacuum line connected to the Schlenk *via* a needle and the needle connected to the Schlenk line manifold was removed, leaving the THF under vacuum. The rubber septum was then wrapped in parafilm. This was done so the flask could

be safely brought into the glovebox. The flask was then brought into the glovebox. NMR sample preparation and analysis was conducted according to **General Procedure for Condition Variation**.

Analysis: There is little change in yield from the result shown in Table S2, entry 4, suggesting that oxygen does not initiate or promote reactivity.

k) Reaction conducted under ambient atmosphere.

Discussion: To provide additional assessment for whether oxygen plays a role in or interferes with this reaction, we conducted a series of reactions using non-degassed THF and under ambient atmosphere.

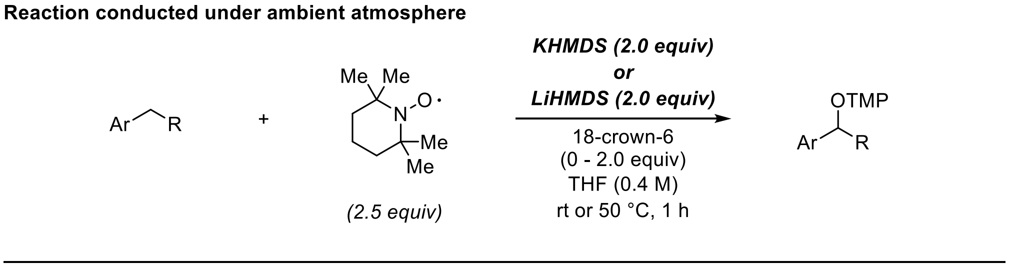

Procedure: A 4 mL, oven-dried dram vial with a stir bar was charged with alkylarene (0.1 mmol) *via* pipette. THF (0.25 mL, 0.4 M) was added to the vial *via* syringe. The THF used was from a commercial bottle stored in a flammable cabinet under ambient atmosphere, in contrast the THF used for all other experiments that is deoxygenated and dried by passage over packed columns of neutral alumina and copper (II) oxide under positive pressure of N_2 (see General Reagent Information, Section I). If required, 18-crown-6 (52.9 mg, 0.2 mmol, 2.0 equiv) was added at this time. TEMPO (39.1 mg, 0.25 mmol, 2.5 equiv) was added followed by solid base (0.2 mmol, 2.0 equiv). The vial was sealed with a screwcap and septum and stirred at the indicated temperature for 1 h. At this time, the vial was unsealed and methanol (0.1 mL, 2.47 mmol, 24.7 equiv) was added *via* syringe. NMR sample preparation and analysis was conducted according to **General Procedure for Condition Variation**.

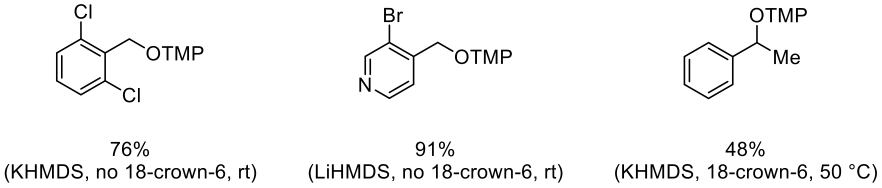

Figure S28: Results from ambient atmosphere experiment where each reaction was conducted under ambient atmosphere using non-deoxygenated THF.

Analysis: There is no or only slight reduction in yield from results shown in Table S1-S3 and no obvious benzylic side products are observed (e.g., peroxide or carbonyl formation). These results indicate that small amounts of oxygen (or moisture) do not substantially interfere with the desired reaction and are consistent with the reactivity being mainly promoted by TEMPO which is present in high concentration in the reaction solution.

m) Evidence for radical formation: stoichiometric metalation and TEMPO addition generates bibenzyl.

Discussion: To test if TEMPO can generate benzylic radicals from benzyl carbanionic intermediates (e.g., benzylpotassium), we conducted an experiment where TEMPO was added to a stoichiometrically

pregenerated benzylpotassium solution. We reasoned that if TEMPO promotes benzylpotassium oxidation to generate a benzyl radical, then bibenzyl may be observed. This would provide insight to the nature of the active intermediates in the HMDS/TEMPO-promoted oxidation method; however, we note that we do not observe benzylic dimer side products under Conditions A-C, consistent with our reaction design and proposal that a low concentration of benzyl carbanionic intermediate prevents radical dimerization and enables selective benzylic oxidation.

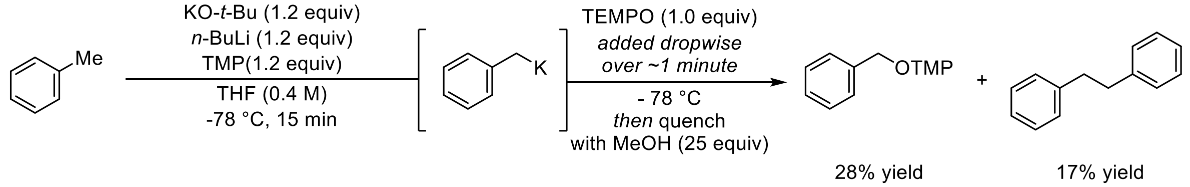

Procedure: Benzylpotassium was prepared according to previous a previous report[13]. All reagents used were used as received without further purification unless noted in the general reagent information section. To a 25 mL, oven-dried round bottom flask with a stir bar, KO-*t*-Bu (26.9 mg, 0.24 mmol, 1.2 equiv) was added. The flask was sealed with a rubber septum and was evacuated and backfilled three times with N_2 gas and left under a positive pressure of N_2 *via* a needle connected to a Schlenk line manifold. To the flask, THF (0.5 mL, 0.4 M) was added *via* syringe. Toluene (21.2 µL, 0.2 mmol, 1.0 equiv) was added *via* syringe and the flask was placed in a -78 °C acetone / dry ice bath and the solution was cooled to -78 °C while stirring (~5 min). To the flask, *n*-butyllithium (1 M in *n*-hexane, 0.24 mL, 0.24 mmol, 1.2 equiv), was added dropwise *via* syringe to afford an orange solution. To an oven-dried scintillation vial, TEMPO (31.2 mg, 0.2 mmol, 1.0 equiv) was added. The vial was sealed with a rubber septum and the septum was wrapped in parafilm. The vial was evacuated and backfilled three times with N_2 gas *via* a needle connected to a Schlenk line manifold. To the vial, THF (2.0 mL, 0.1 M) was added *via* a syringe. The solution was taken up into a syringe under N_2 and added dropwise to the benzylpotassium solution over the course of approximately 1 min. After the addition was complete, methanol (0.2 mL, 4.9 mmol, 25 equiv) was added *via* syringe. The flask was removed from the cooling bath and the quenched reaction mixture was allowed to warm to room temperature. Once warmed, the flask was unsealed and TMB (31.4 mg, 187 µmol) was added. An aliquot (~0.2 mL) of the crude, quenched reaction mixture was transferred to a scintillation vial and THF was removed *via* air blowdown evaporation. To the scintillation vial, $CDCl_3$ (1.0 mL) was added, the solution was taken up and transferred two NMR tubes (0.5 mL solution per tube). To one of the NMR tubes, bibenzyl authentic standard (Combi-Blocks, catalog #QA-0200, ~5 mg) was spiked in. ^1H NMR spectroscopy was used to evaluate the yield of 1-(benzyloxy)-2,2,6,6-tetramethylpiperidine and bibenzyl. NMR spectra of the crude reaction mixture are provided below in Figures S29-30.

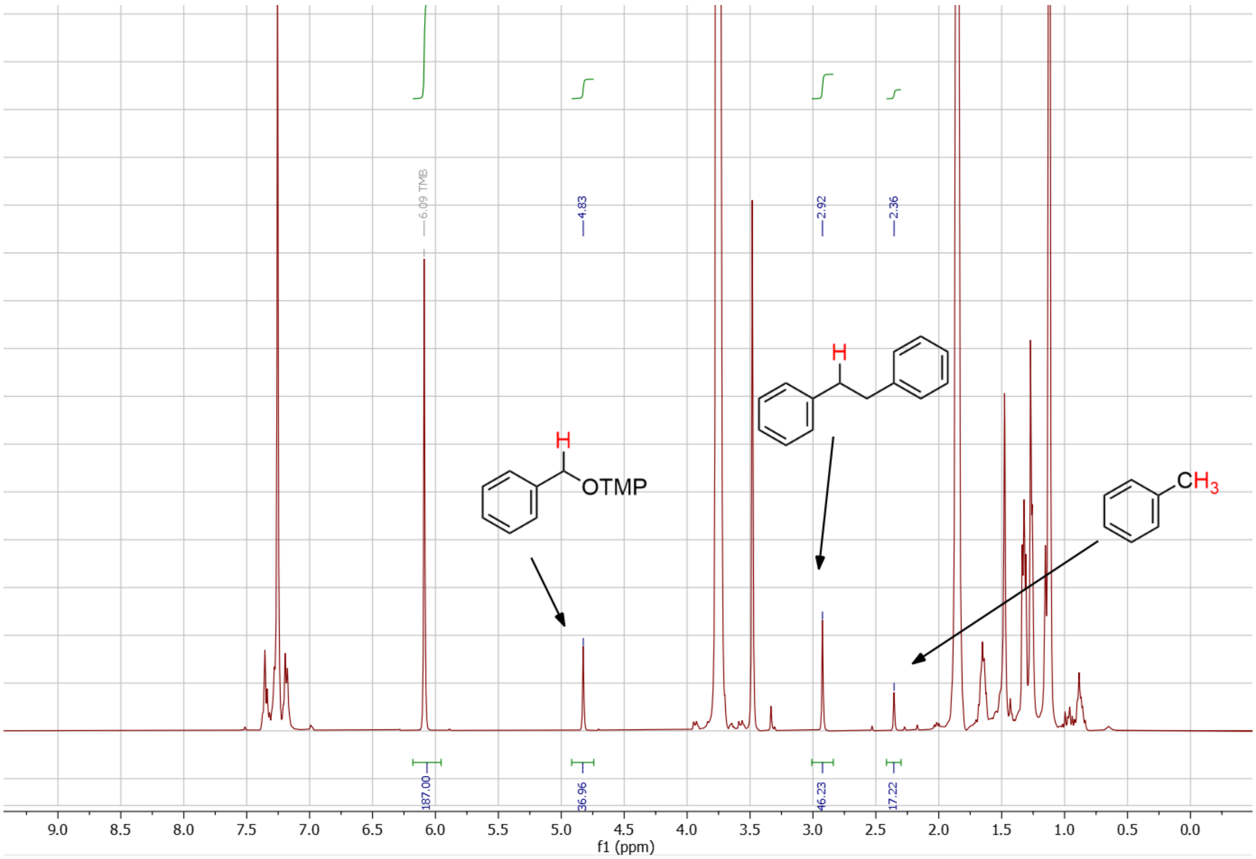

Figure S29: ^{1}H NMR spectrum of the crude reaction mixture of the reaction of benzylpotassium and TEMPO. The aromatic signal of TMB (31.4 mg, 187 µmol) at 6.09 ppm (s, 3H) was integrated against the methylene signal of 1-(benzyloxy)-2,2,6,6-tetramethylpiperidine at 4.83 ppm (s, 2H) (55 µmol, 28% yield) and the methylene signals of bibenzyl at 2.92 ppm (s, 4H) (35 µmol, 17% yield).

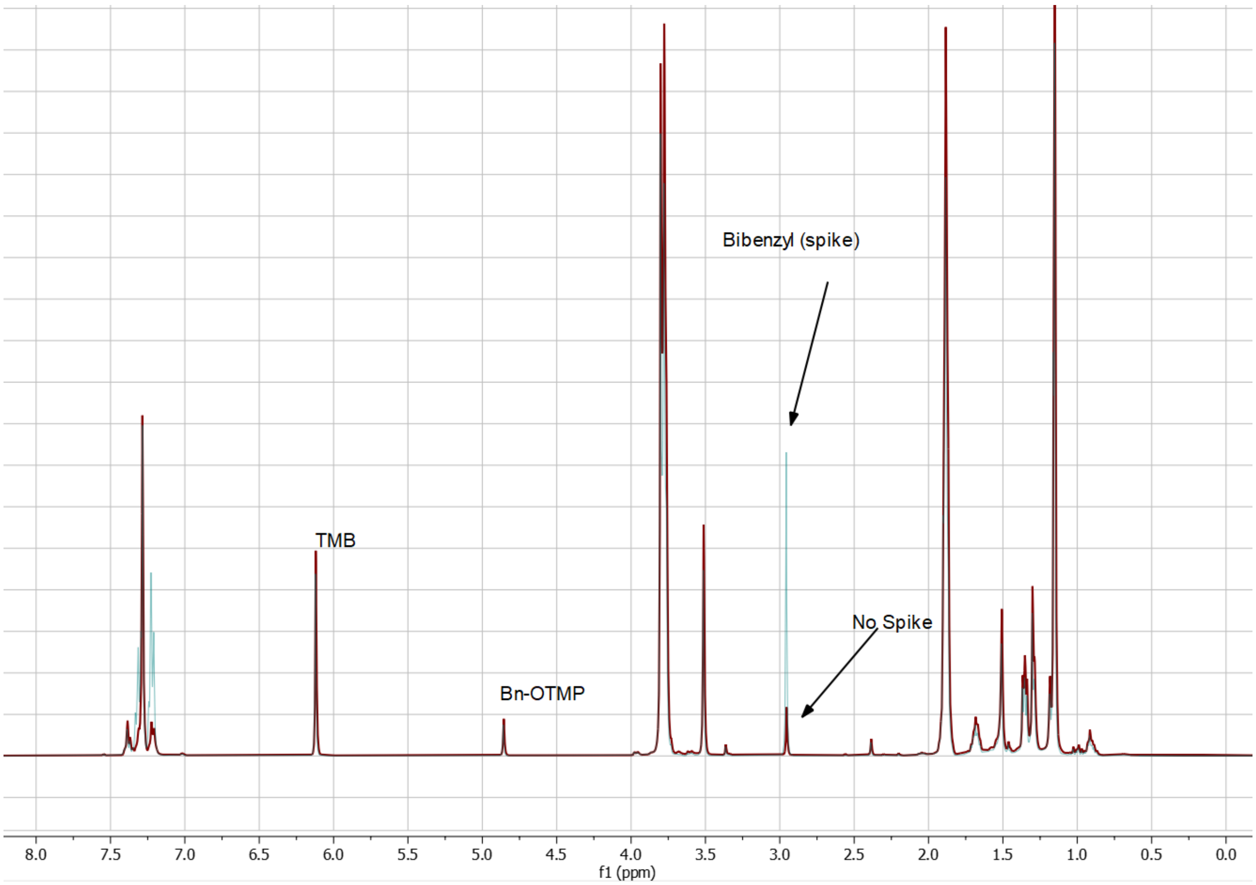

Figure S30: Overlayed ^{1}H NMR spectra of the crude reaction mixture of the reaction of benzylpotassium and TEMPO (red spectrum) and the crude reaction mixture with bibenzyl spiked in (blue spectrum).

Analysis: The addition of TEMPO (0.1 M in THF) solution to a solution of benzylpotassium (0.4 M in THF) results in a mixture of 1-(benzyloxy)-2,2,6,6-tetramethylpiperidine in 28% yield and bibenzyl in 17% yield. Regarding stoichiometry with respect to TEMPO: two moles of TEMPO are required for 1 mole of 1-(benzyloxy)-2,2,6,6-tetramethylpiperidine to form and two moles of TEMPO are required for one mole of bibenzyl to form. With the determined yields, for the 55 µmol of 1-(benzyloxy)-2,2,6,6-tetramethylpiperidine that formed, 110 µmol of TEMPO were required and for the 35 µmol of bibenzyl that formed, 70 µmol of TEMPO were required (total 180 µmol / 200 µmol consumed). The yields of these products are consistent with TEMPO serving as a single electron oxidant. Furthermore, upon the addition of methanol, the reaction solution turns colorless which is consistent with complete TEMPO consumption. The formation of bibenzyl is consistent with the formation of a discrete benzyl radical. To rule out the possibility of bibenzyl forming *via* an S_N2 reaction between 1-(benzyloxy)-2,2,6,6-tetramethylpiperidine and benzylpotassium, another control experiment (not shown, 0.2 mmol scale) was conducted where benzylpotassium was prepared in the same manner and 1-(benzyloxy)-2,2,6,6-tetramethylpiperidine (0.1 M in THF, 2 mL, 49.5 mg, 0.2 mmol, 1.0 equiv) was added dropwise over the course of 1 minute followed by a quench with methanol (0.2 mL, 4.9 mmol, 25 equiv). We observed <2% bibenzyl formation which could either form *via* trace oxygen in the solvent or reaction vessel headspace which oxidizes the benzylpotassium or *via* S_N2 from benzylpotassium with 1-(benzyloxy)-2,2,6,6-tetramethylpiperidine. Regardless, due to the low yield of bibenzyl that forms in this second control reaction, we conclude that S_N2 is not the primary pathway for bibenzyl formation. These collective observations are consistent with TEMPO oxidizing benzylpotassium to generate a benzyl radical intermediate.

VII. References

(1) Fulmer, G. R.; Miller, A. J. M.; Sherden, N. H.; Gottlieb, H. E.; Nudelman, A.; Stoltz, B. M.; Bercaw, J. E.; Goldberg, K. I. NMR Chemical Shifts of Trace Impurities: Common Laboratory Solvents, Organics, and Gases in Deuterated Solvents Relevant to the Organometallic Chemist. *Organometallics* **2010**, 29, 2176–2179.

(2) Planas, O.; Wang, F.; Leutzsch, M.; Cornella, J. Fluorination of Arylboronic Esters Enabled by Bismuth Redox Catalysis. *Science* **2020**, 367, 313–317.

(3) Ortalli, S.; Ford, J.; Trabanco, A. A.; Tredwell, M.; Gouverneur, V. Photoredox Nucleophilic (Radio)Fluorination of Alkoxyamines. *J. Am. Chem. Soc.* **2024**, *146*, 11599–11604.

(4) Li, L.; Yu, Z.; Shen, Z. Copper-Catalyzed Aminoxylation of Different Types of Hydrocarbons with TEMPO: A Concise Route to N-Alkoxyamine Derivatives. *Advan. Synth. Catal.* **2015**, *357*, 3495–3500.

(5) Lu, Z.; Ju, M.; Wang, Y.; Meinhardt, J. M.; Martinez Alvarado, J. I.; Villemure, E.; Terrett, J. A.; Lin, S. Regioselective Aliphatic C–H Functionalization Using Frustrated Radical Pairs. *Nature* **2023**, *619*, 514–520.

(6) Tabassum, S.; Sereda, O.; Reddy, P. V. G.; Wilhelm, R. Hindered Brønsted Bases as Lewis Base Catalysts. *Org. Biomol. Chem.* **2009**, *7*, 4009.

(7) Zhu(s), H.; Wu, Y.; Mao, J.; Xu, J.; Walsh, P. J.; Shi, H. C–H Functionalization through Benzylic Deprotonation with π-Coordination or Cation–π-Interactions. *Chem. Soc. Rev.* **2025**, *54*, 2520–2542

(8) Olmstead, W. N.; Margolin, Z.; Bordwell, F. G. Acidities of Water and Simple Alcohols in Dimethyl Sulfoxide Solution. *J. Org. Chem.* **1980**, *45*, 3295–3299.

(9) *2,2,6,6-Tetramethylpiperidin-1-ol, 97%* (CAS RN 7031-93-8). A746499; Ambeed: Buffalo Grove, IL, February 23, 2026. https://file.ambeed.com/static/upload/prosds/am/747/SDS-A746499.pdf

(10) Ma, Y.; Loyns, C.; Price, P.; Chechik, V. Thermal Decay of TEMPO in Acidic Media *via* an *N*-Oxoammonium Salt Intermediate. *Org. Biomol. Chem.* **2011**, 9, 5573–5578.

(11) Wang, J.; Yang, S. A Silver Triflate-Catalyzed Cascade of in Situ-Oxidation and Allylation of Arylbenzylamines. *Tetrahedron Lett.* **2016**, *57*, 3444–3448.

(12) Ju, M.; Lee, S.; Marvich, H. M.; Lin, S. Accessing Alkoxy Radicals via Frustrated Radical Pairs: Diverse Oxidative Functionalizations of Tertiary Alcohols. *J. Am. Chem. Soc.* **2024**, *146*, 19696–19703.

(13) Manvar, A.; Fleming, P.; O'Shea, D. F. General Ambient Temperature Benzylic Metalations Using Mixed-Metal Li/K-TMP Amide. *J. Org. Chem.* **2015**, *80*, 8727–8738.

VIII. NMR Spectra

1-((2,6-dichlorobenzyl)oxy)-2,2,6,6-tetramethylpiperidine (4)

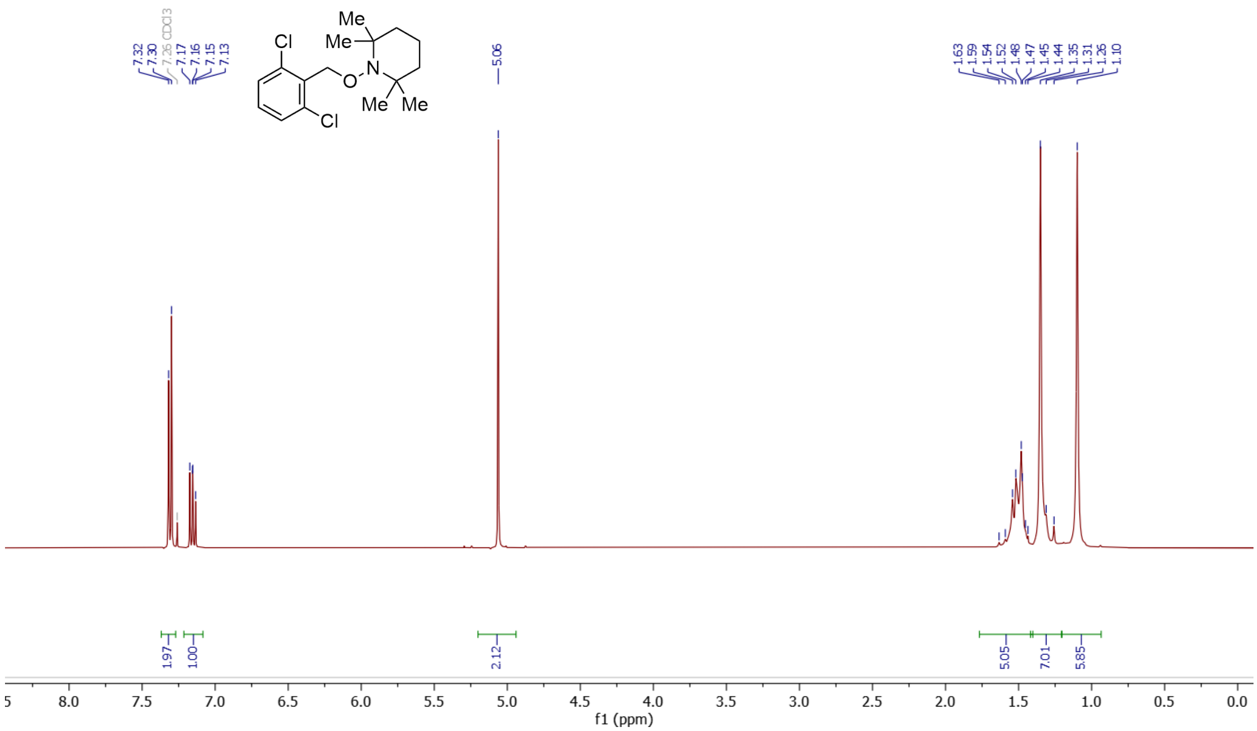

^{1}**H NMR** spectrum of **4** (400 MHz, $CDCl_3$)

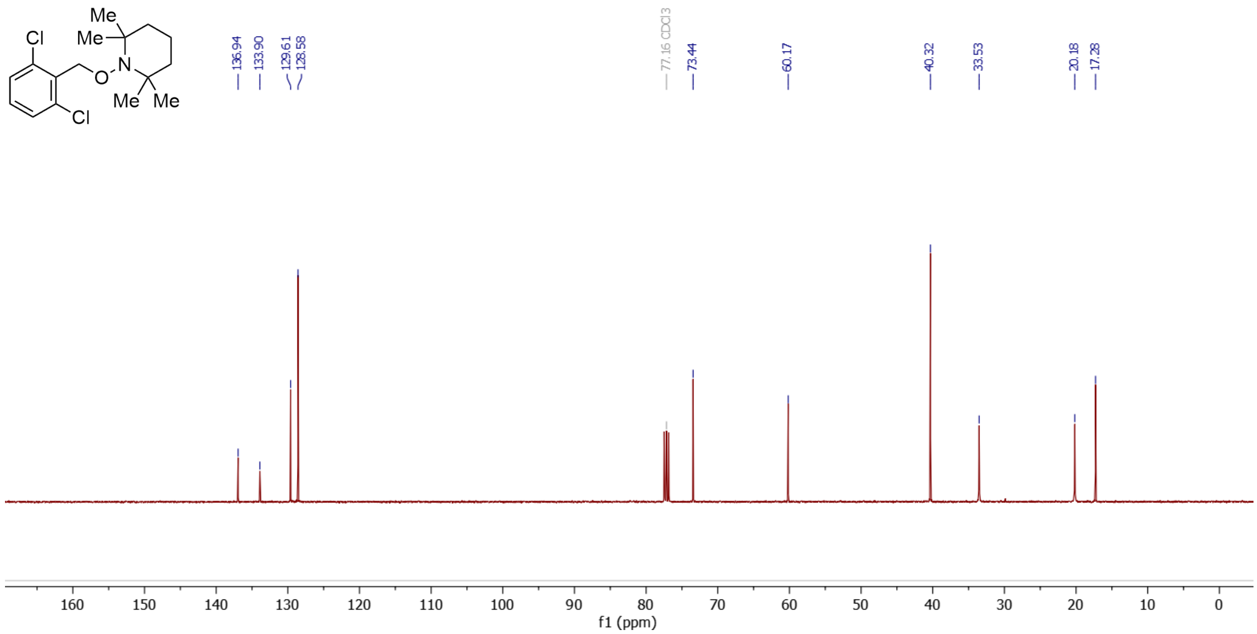

^{13}**C NMR** spectrum of **4** (101 MHz, $CDCl_3$)

2-(1-((2,2,6,6-tetramethylpiperidin-1-yl)oxy)ethyl)pyrazine (5)

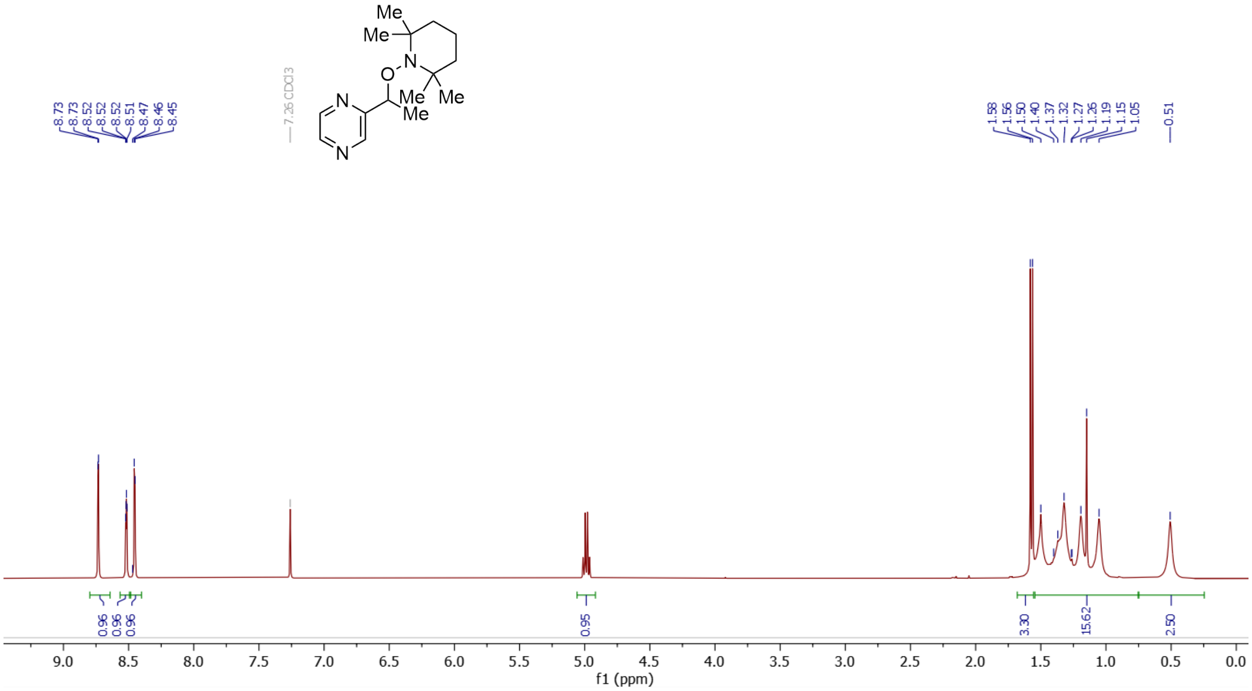

^{1}H NMR spectrum of **5** (400 MHz, $CDCl_3$)

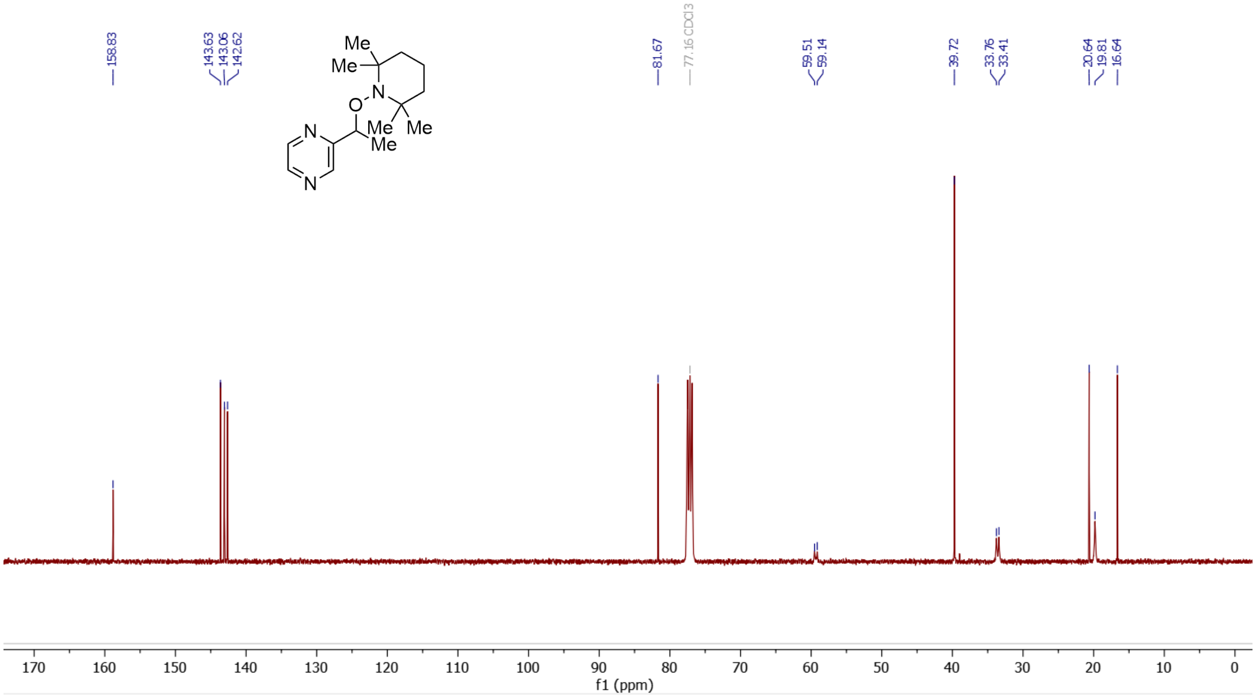

^{13}C NMR spectrum of **5** (101 MHz, $CDCl_3$)

2-(2-((2,2,6,6-tetramethylpiperidin-1-yl)oxy)propan-2-yl)pyridine (6)

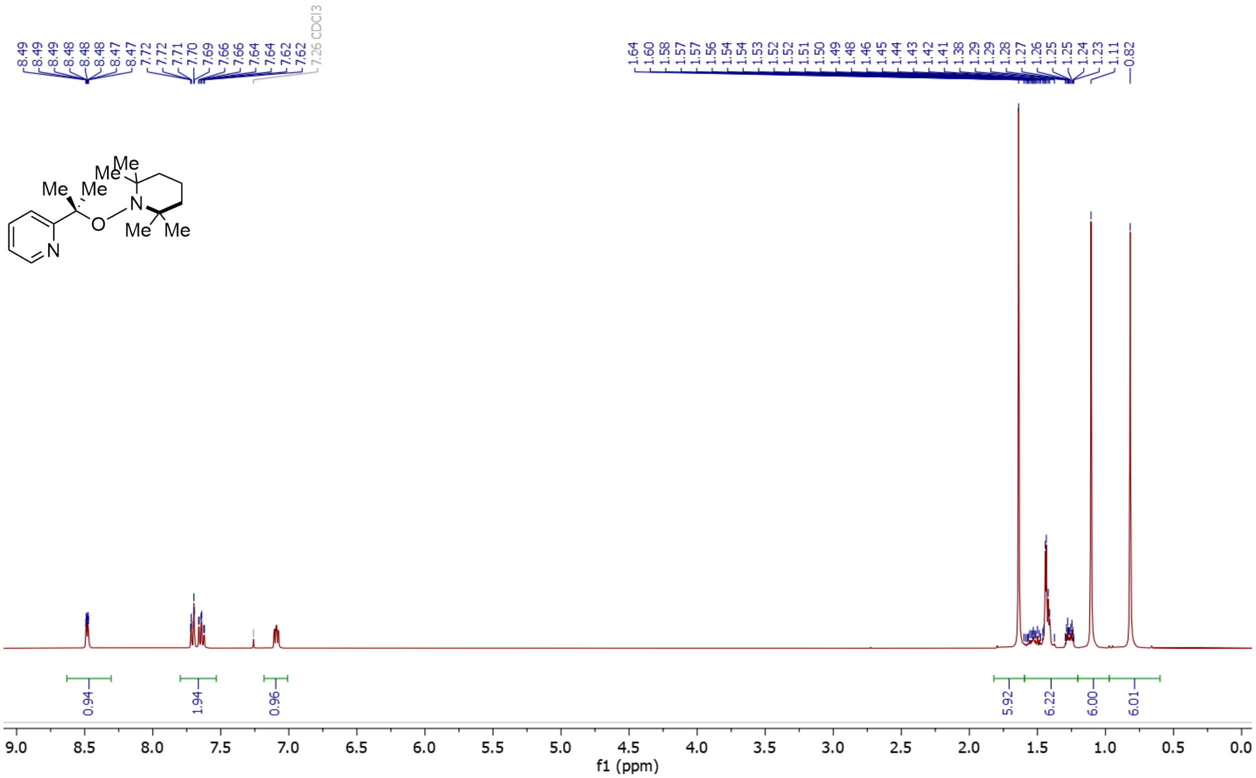

^{1}H NMR spectrum of **6** (400 MHz, $CDCl_3$)

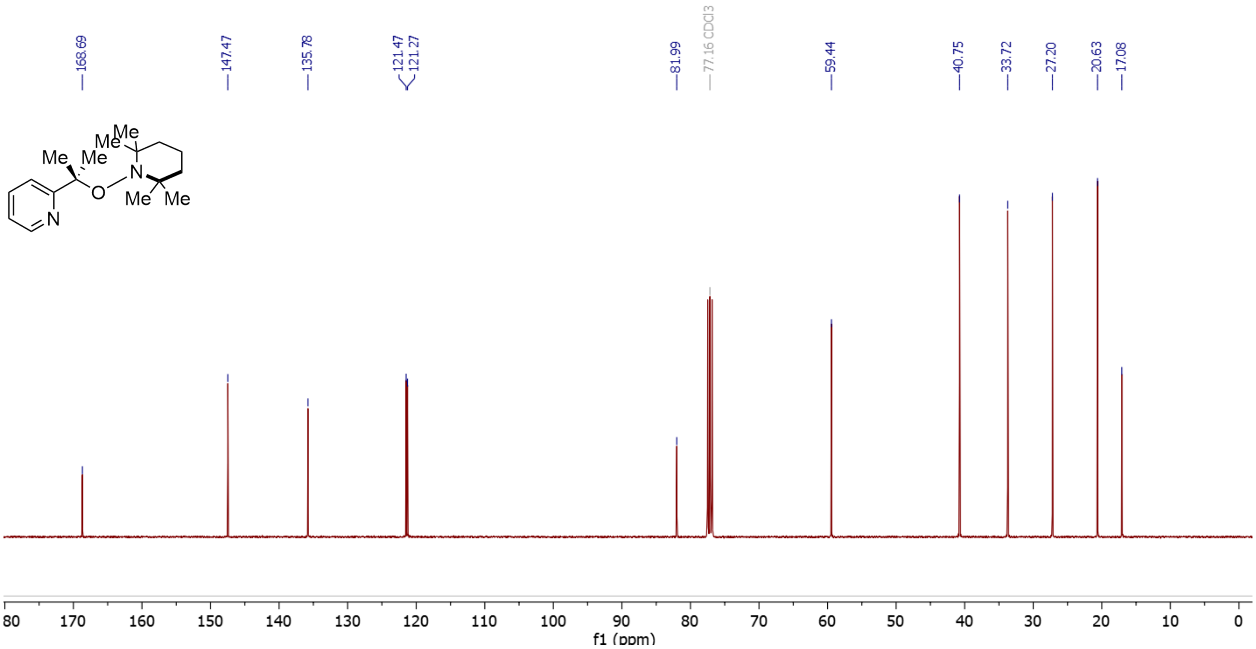

^{13}C NMR spectrum of **6** (101 MHz, $CDCl_3$)

2-(2-methyl-1-((2,2,6,6-tetramethylpiperidin-1-yl)oxy)propyl)thiazole (7)

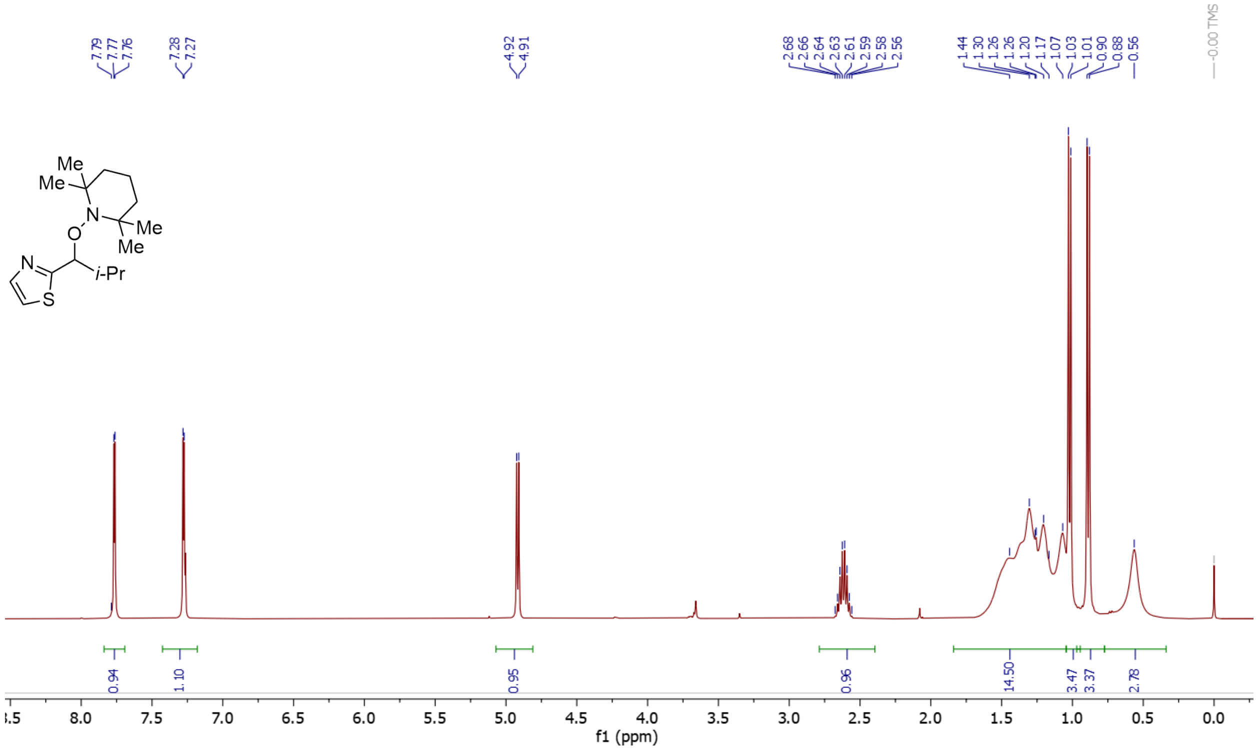

^{1}**H NMR** spectrum of **7** (400 MHz, $CDCl_3$)

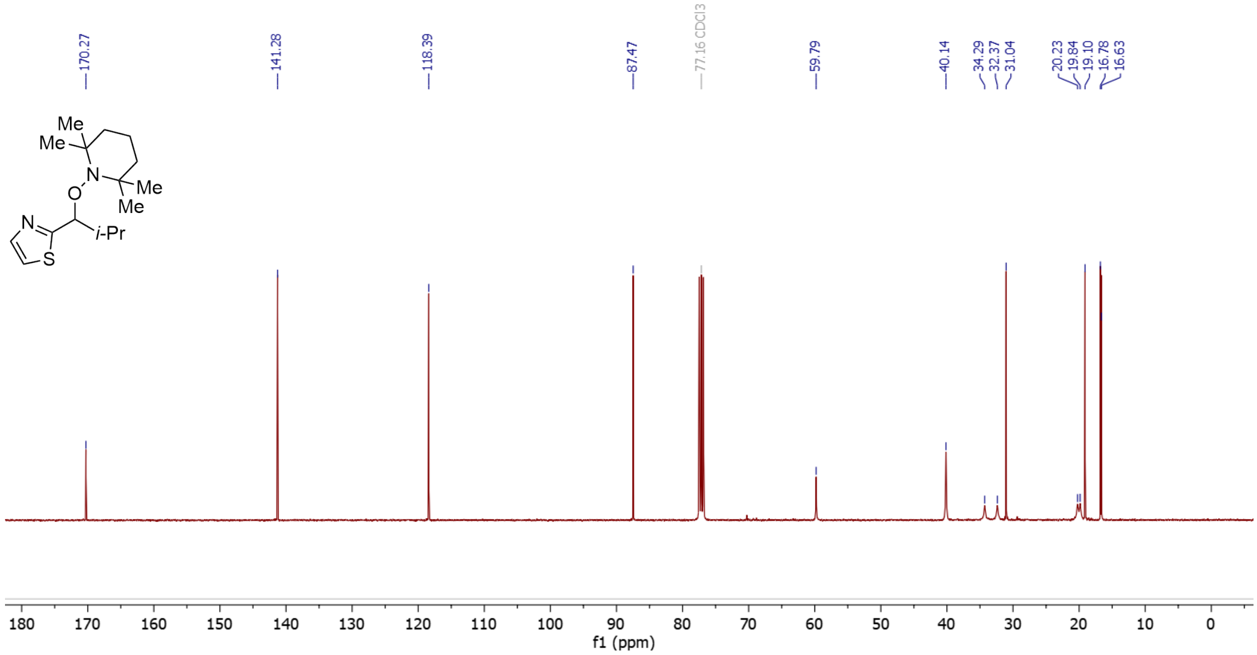

^{13}**C NMR** spectrum of **7** (101 MHz, $CDCl_3$)

3-(1-((2,2,6,6-tetramethylpiperidin-1-yl)oxy)ethyl)pyridine (8)

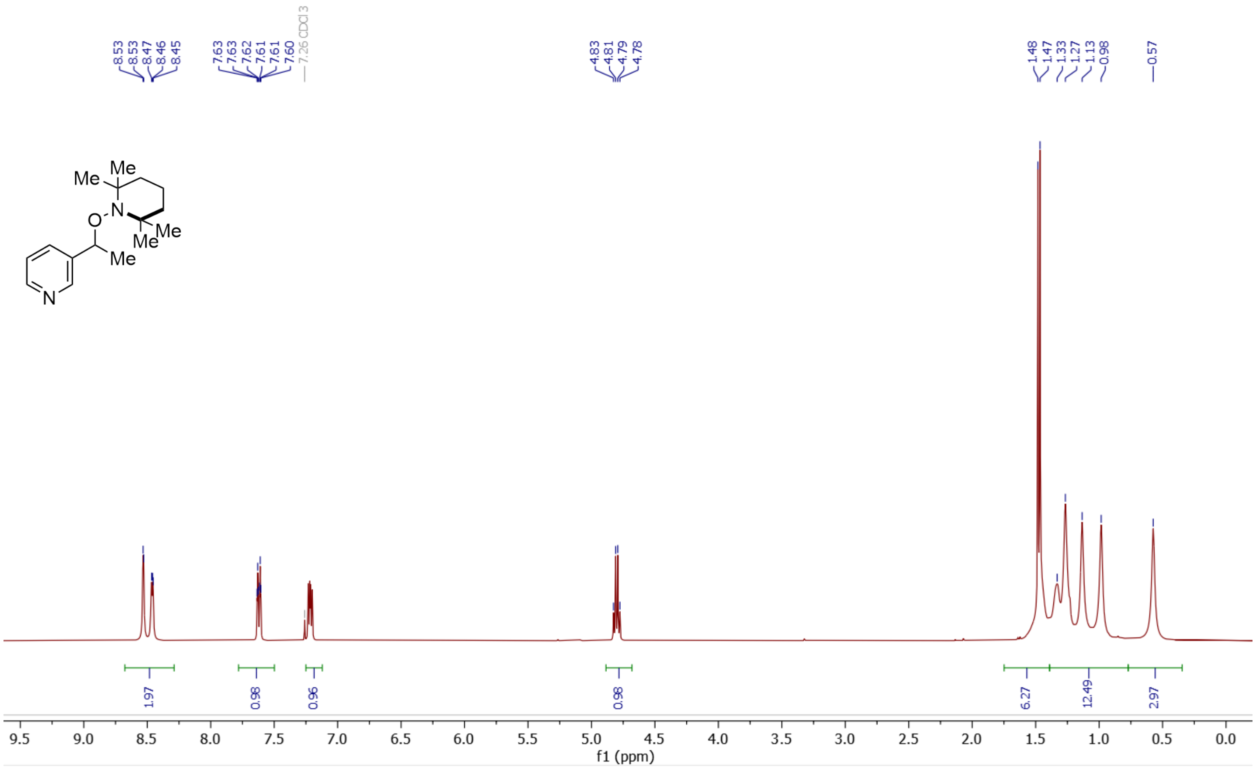

^{1}H NMR spectrum of **8** (400 MHz, $CDCl_3$)

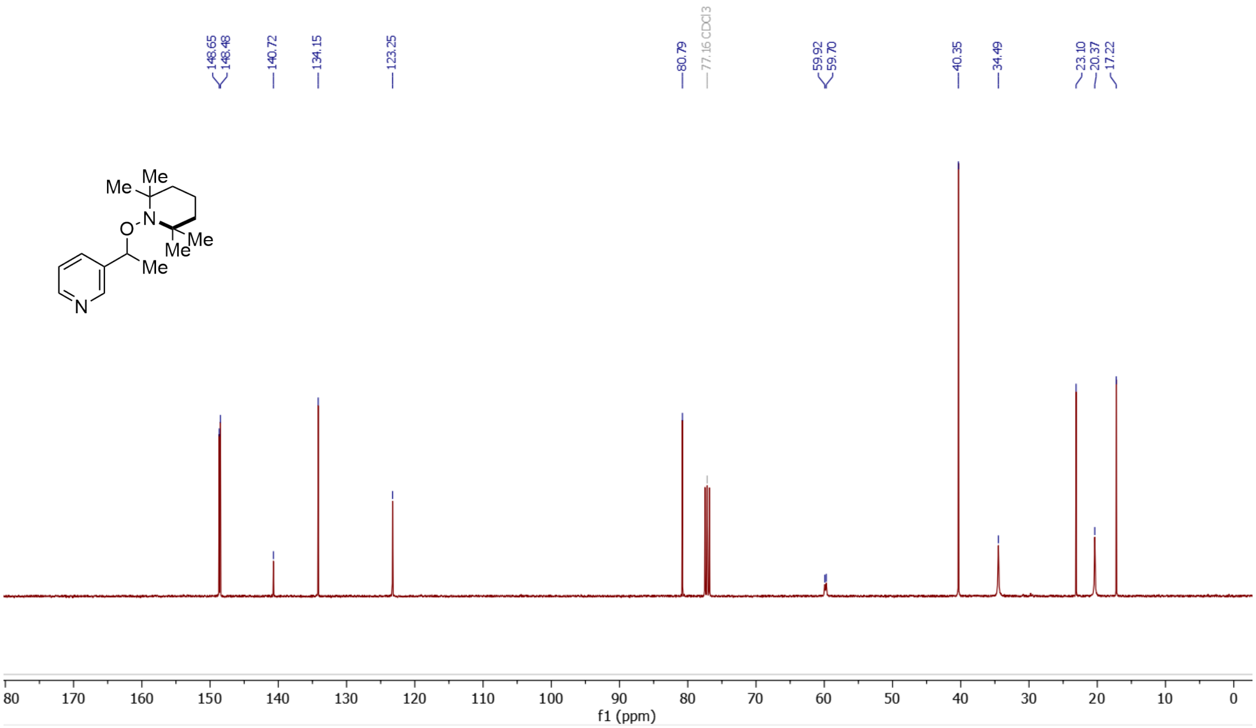

^{13}C NMR spectrum of **8** (101 MHz, $CDCl_3$)

2,2,6,6-tetramethyl-1-(1-phenylethoxy)piperidine (9)

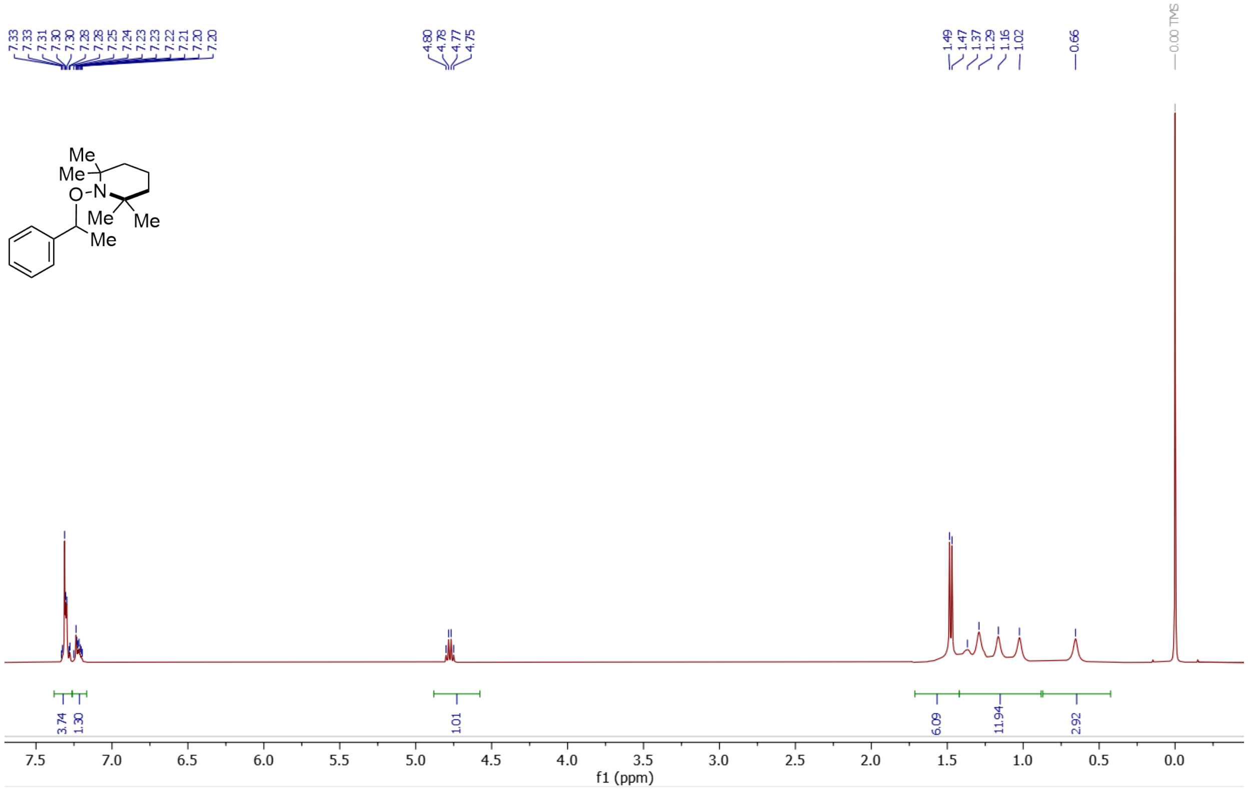

^{1}H NMR spectrum of **9** (400 MHz, $CDCl_3$)

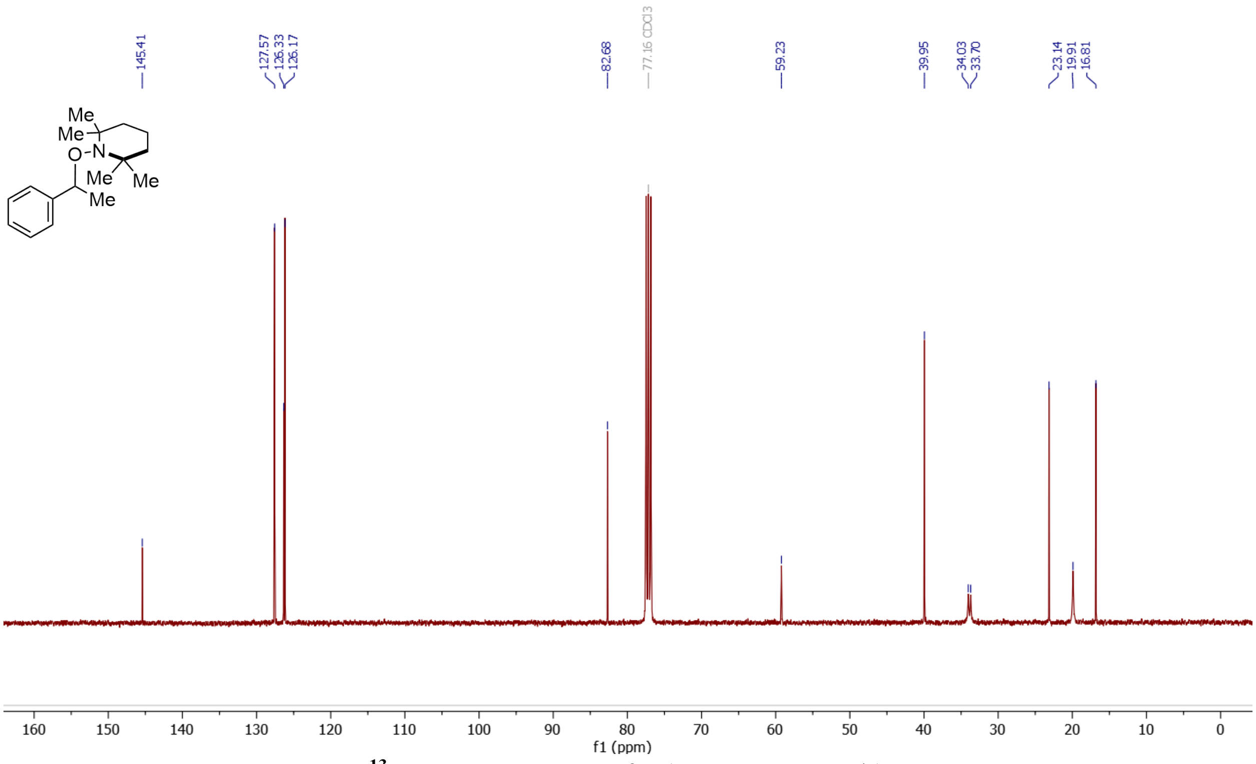

^{13}C NMR spectrum of **9** (101 MHz, $CDCl_3$)

4-(((2,2,6,6-tetramethylpiperidin-1-yl)oxy)methyl)benzo[c][1,2,5]thiadiazole (10)

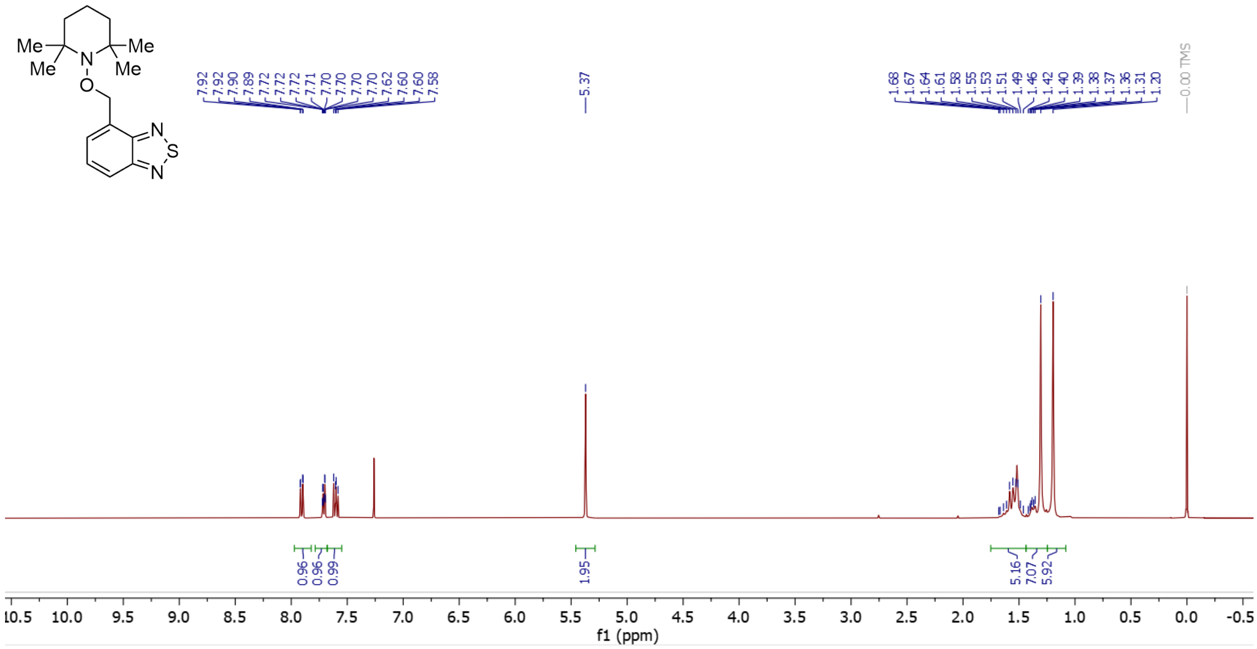

^{1}H NMR spectrum of **10** (400 MHz, $CDCl_3$)

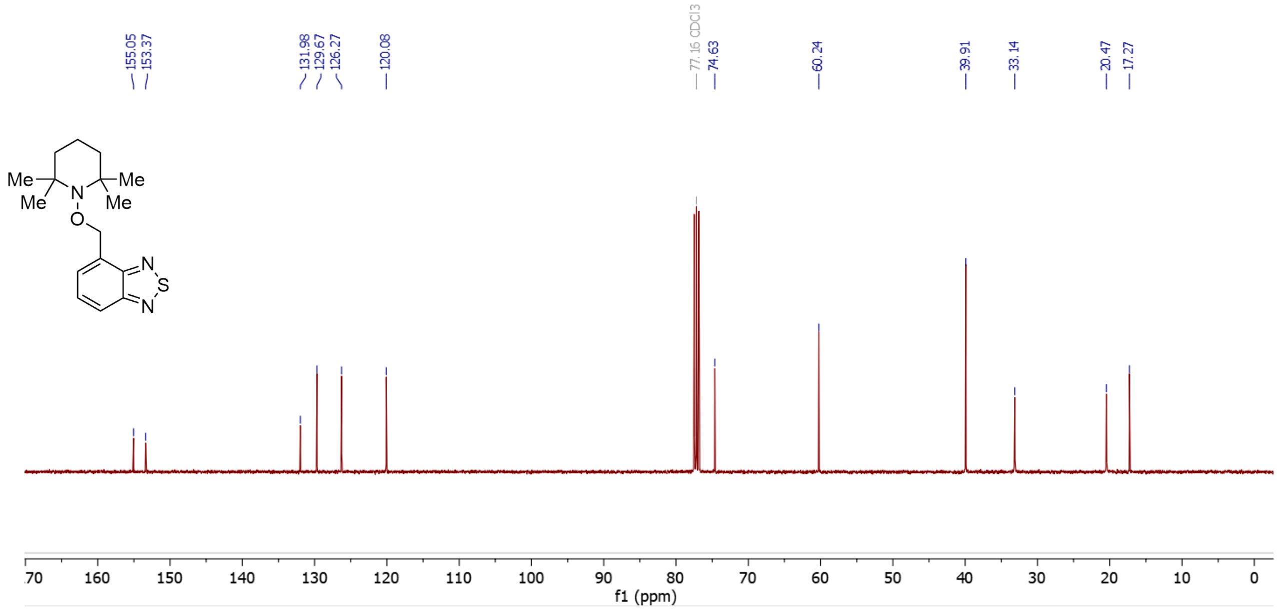

^{13}C NMR spectrum of **10** (101 MHz, $CDCl_3$)

5-(((2,2,6,6-tetramethylpiperidin-1-yl)oxy)methyl)benzo[c][1,2,5]oxadiazole (11)

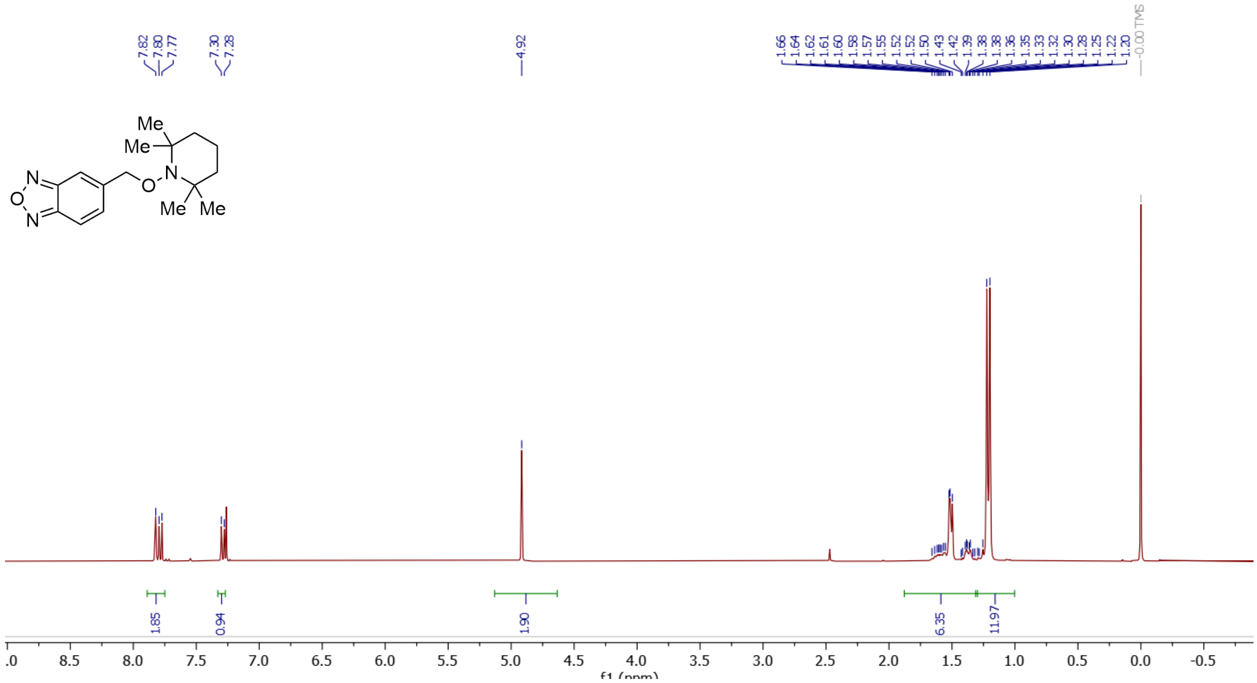

^{1}**H NMR** spectrum of **11** (400 MHz, $CDCl_3$)

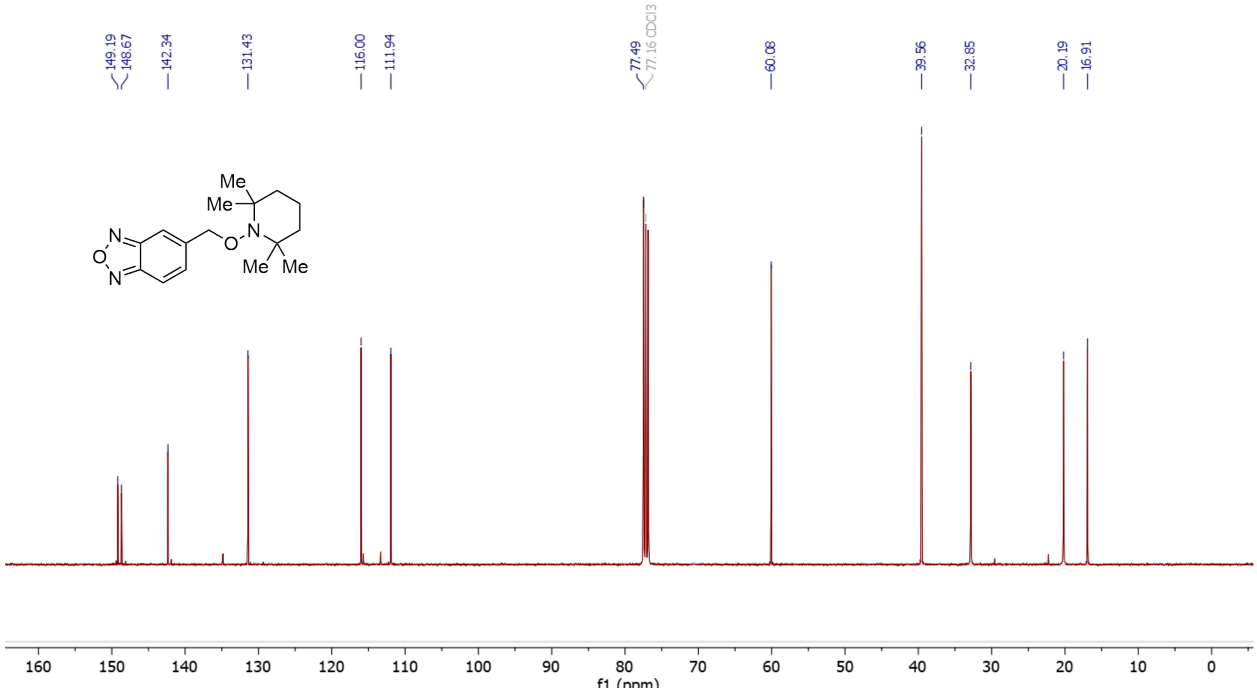

^{13}**C NMR** spectrum of **11** (101 MHz, $CDCl_3$)

1-(benzo[b]thiophen-2-ylmethoxy)-2,2,6,6-tetramethylpiperidine (12)

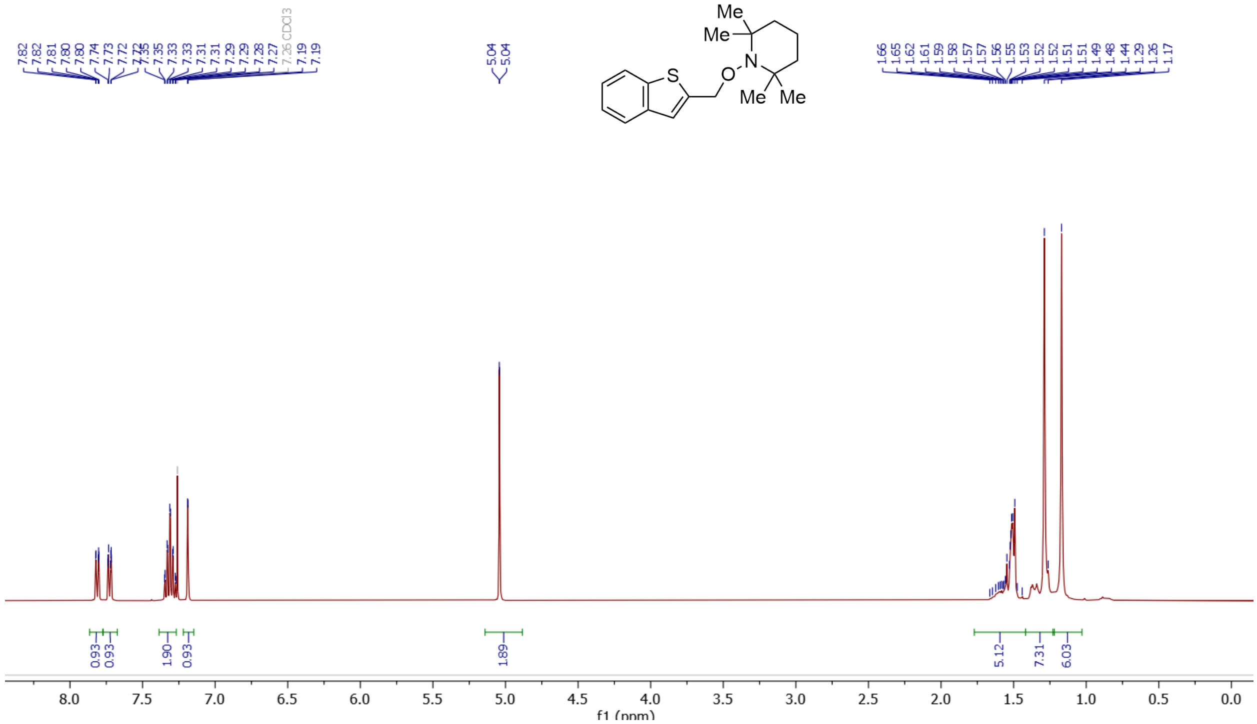

^{1}**H NMR** spectrum of **12** (400 MHz, $CDCl_3$)

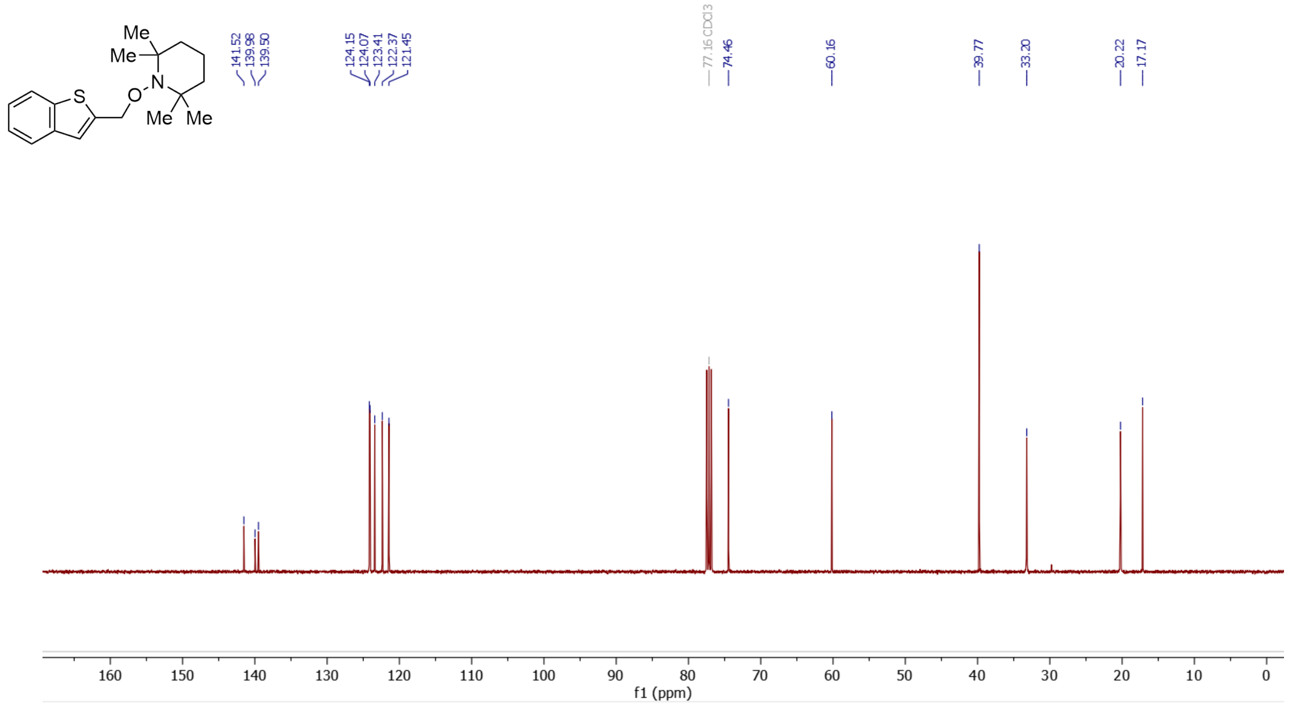

^{13}**C NMR** spectrum of **12** (101 MHz, $CDCl_3$)

1-methyl-2-(((2,2,6,6-tetramethylpiperidin-1-yl)oxy)methyl)-1H-indole (13)

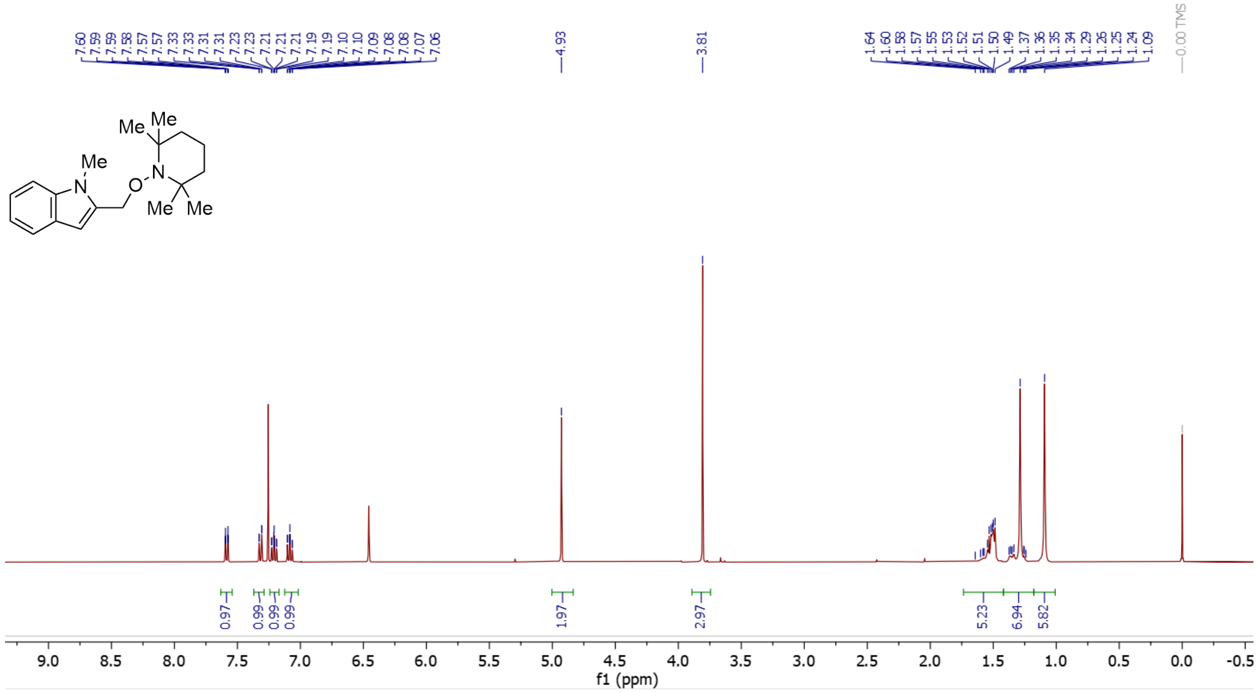

[1]**H NMR** spectrum of **13** (400 MHz, $CDCl_3$)

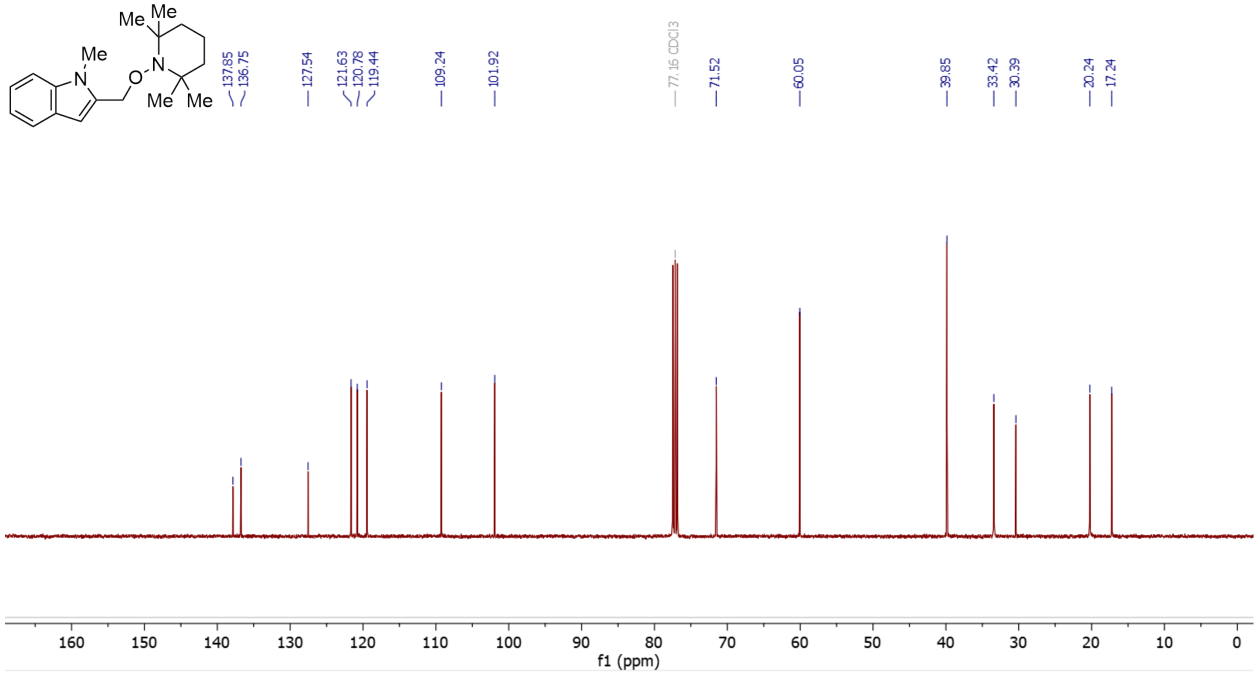

[13]**C NMR** spectrum of **13** (101 MHz, $CDCl_3$)

1-(dibenzo[*b,d*]thiophen-4-ylmethoxy)-2,2,6,6-tetramethylpiperidine (14)

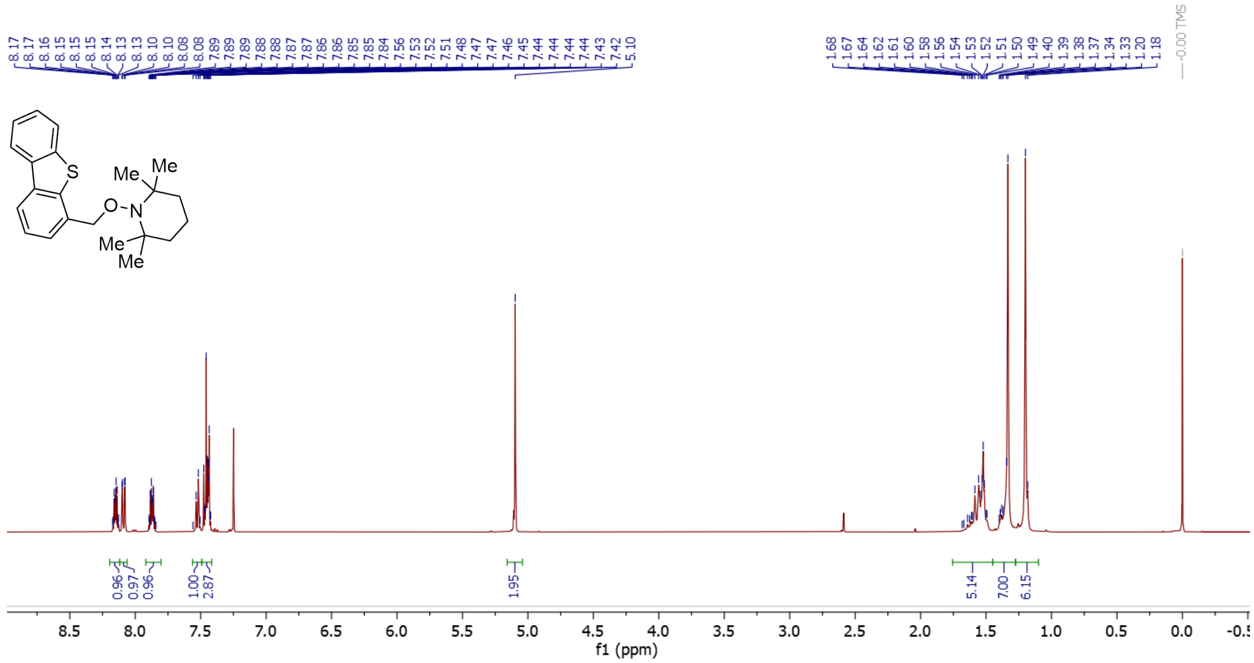

^{1}**H NMR** spectrum of **14** (400 MHz, $CDCl_3$)

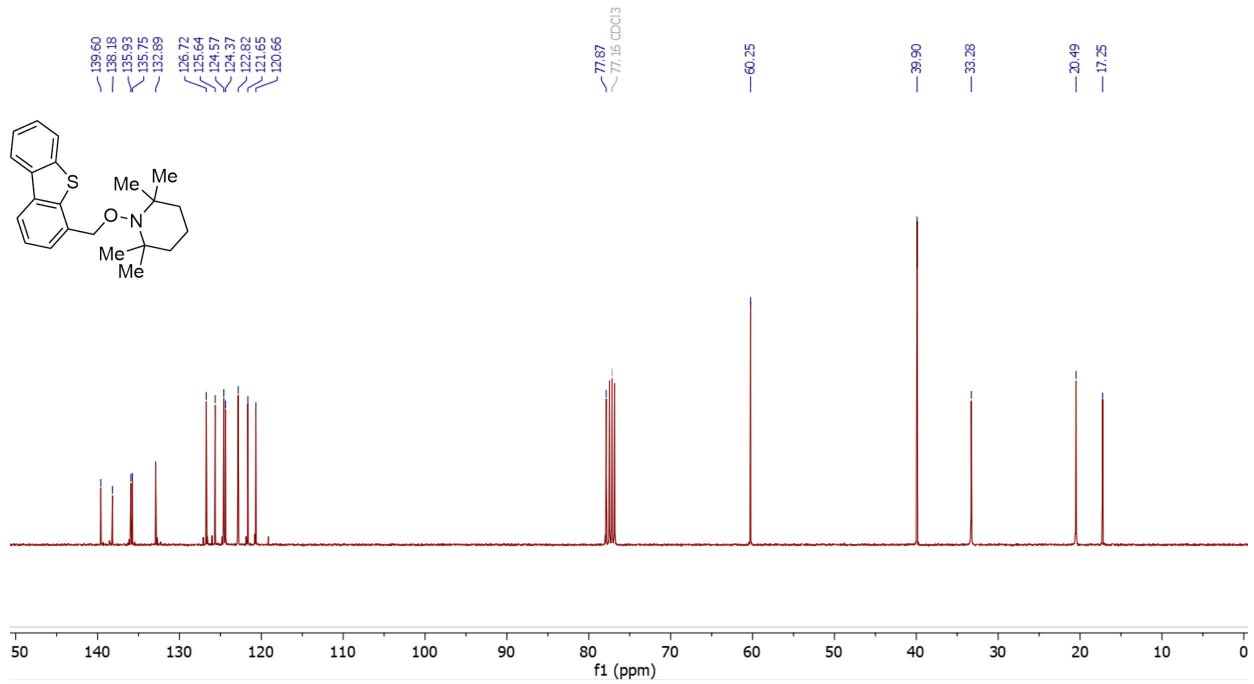

^{13}**C NMR** spectrum of **14** (101 MHz, $CDCl_3$)

8-(pyridin-2-yl)-2-(((2,2,6,6-tetramethylpiperidin-1-yl)oxy)methyl)benzofuro[2,3-b]pyridine (15)

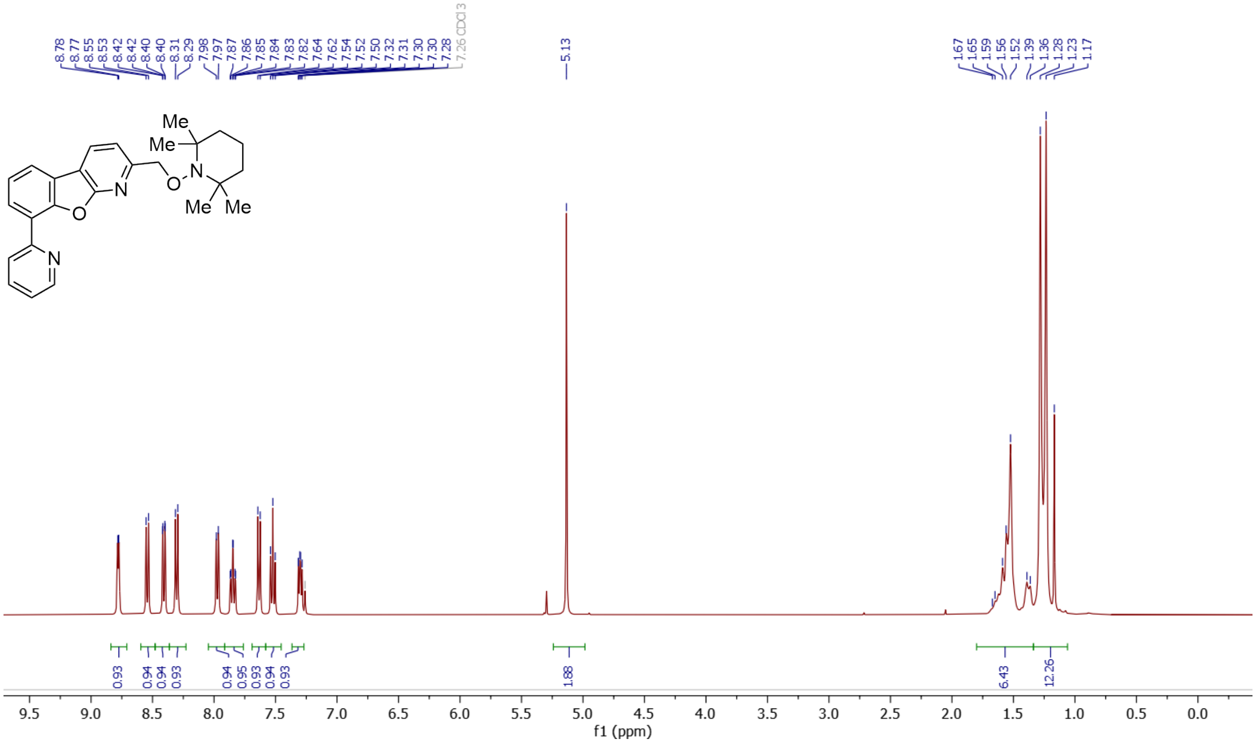

^{1}H NMR spectrum of **15** (400 MHz, $CDCl_3$)

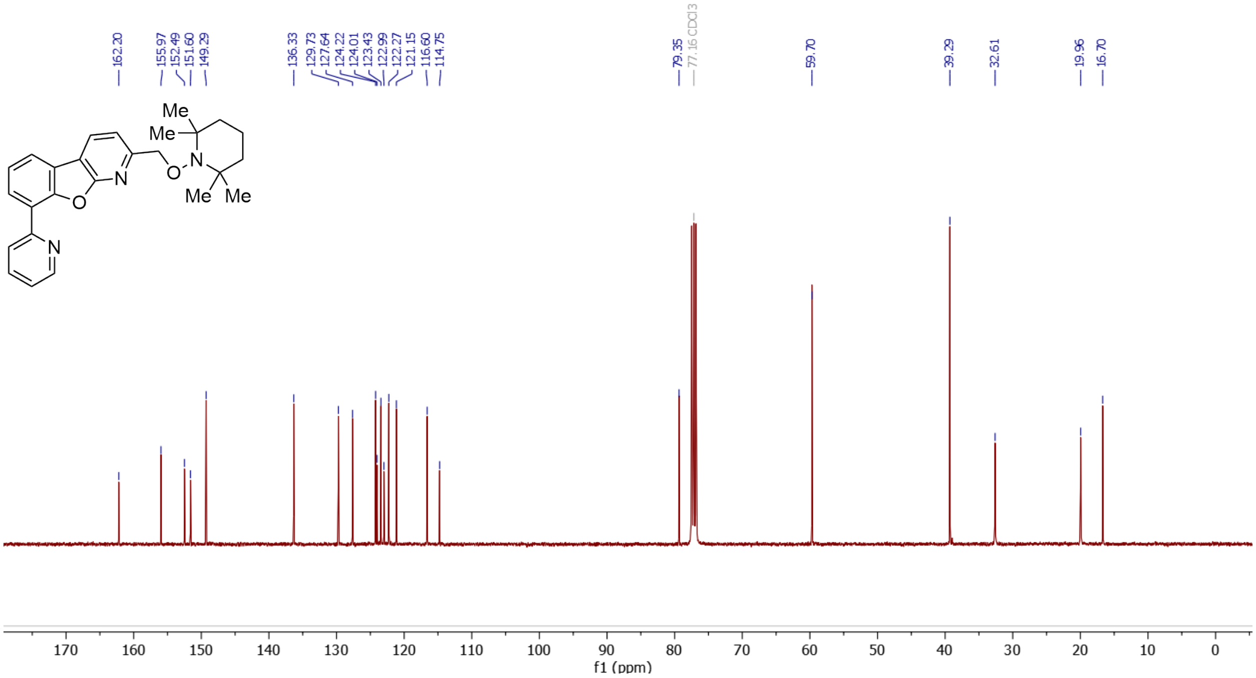

^{13}C NMR spectrum of **15** (101 MHz, $CDCl_3$)

4'-(((2,2,6,6-tetramethylpiperidin-1-yl)oxy)methyl)-[1,1'-biphenyl]-2-carbonitrile (16)

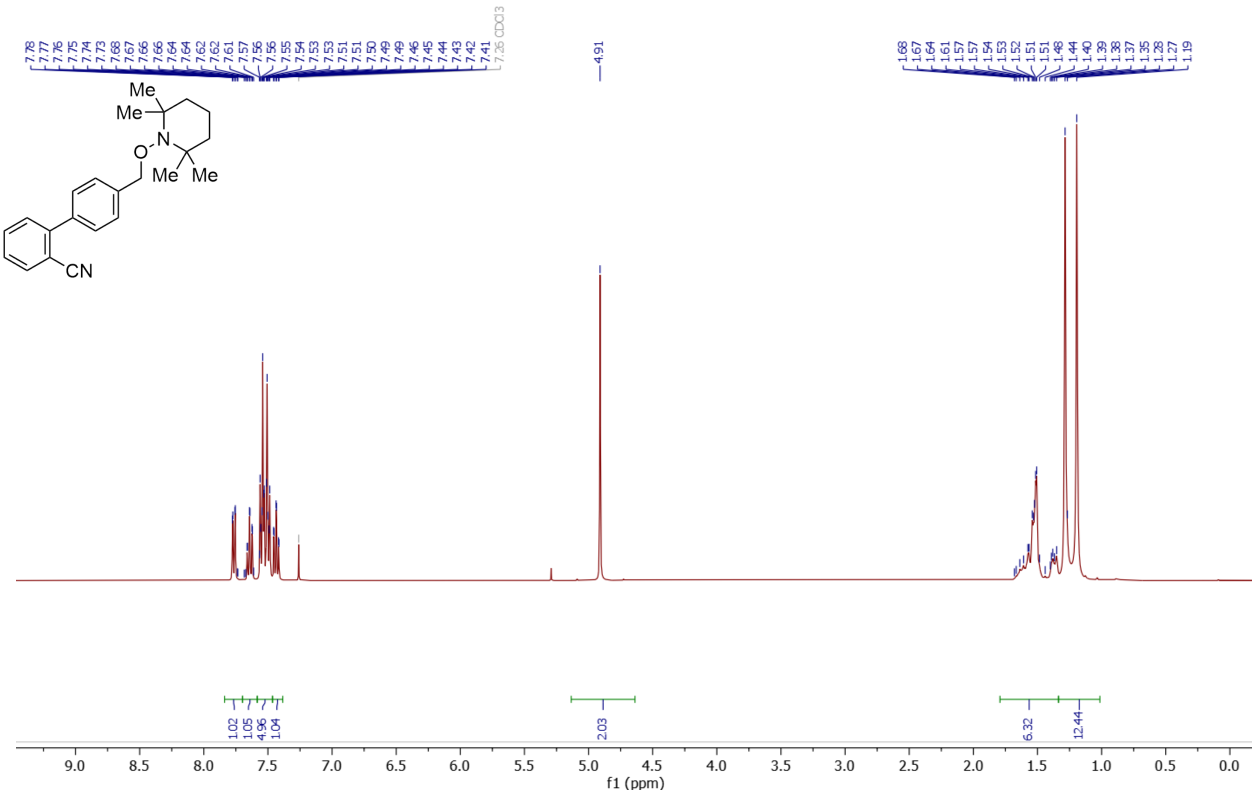

^{1}H NMR spectrum of **16** (400 MHz, $CDCl_3$)

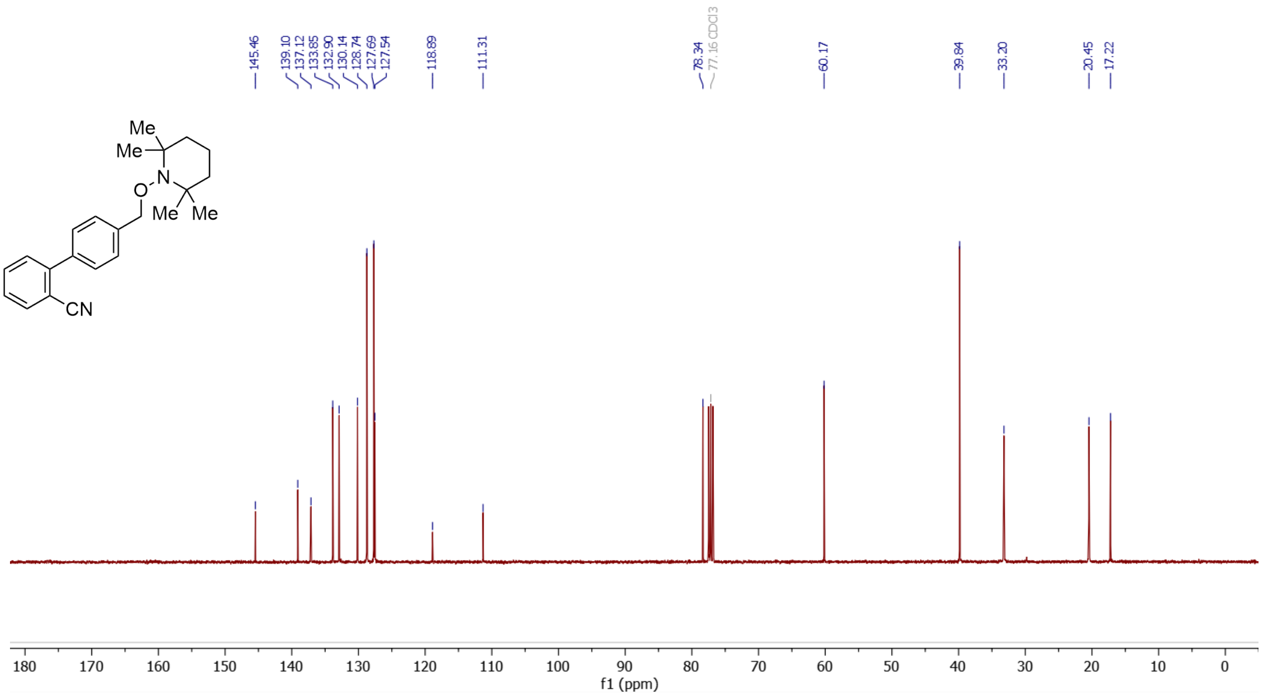

^{13}C NMR spectrum of **16** (101 MHz, $CDCl_3$)

2,2,6,6-tetramethyl-1-((4-((trifluoromethyl)thio)benzyl)oxy)piperidine (17)

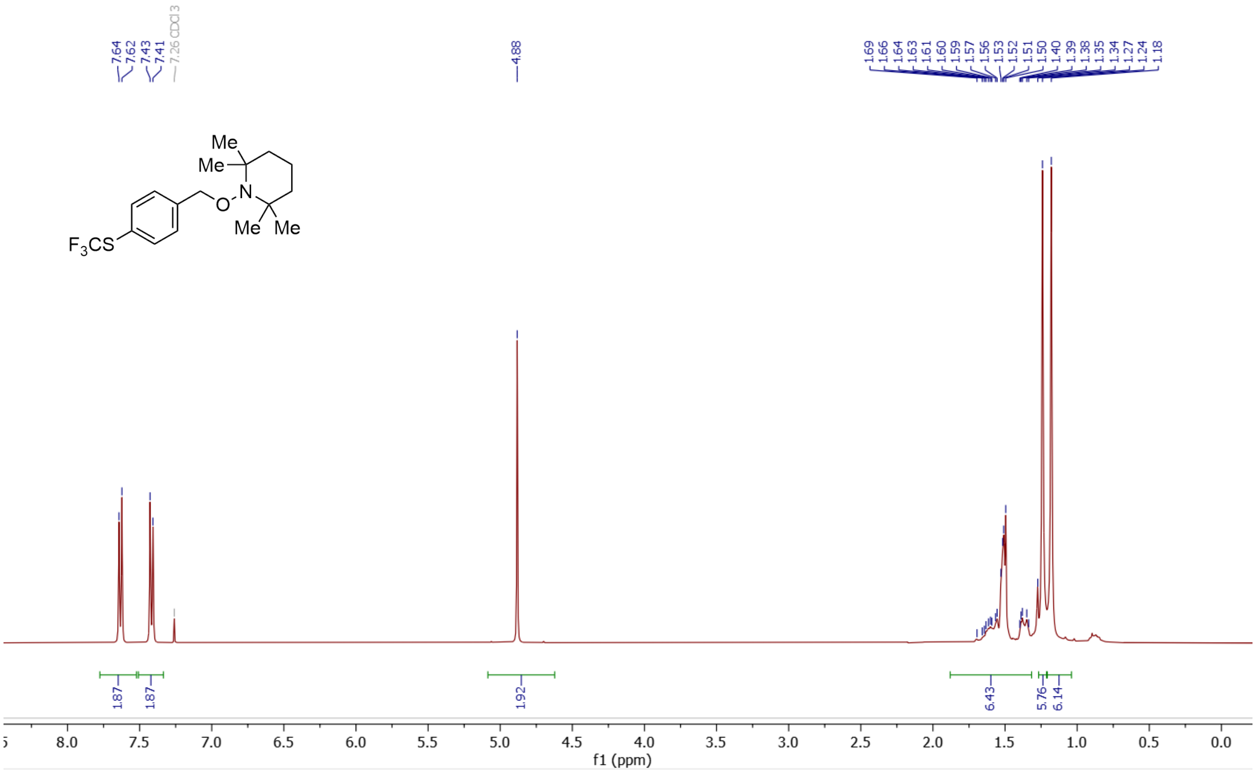

^{1}H NMR spectrum of **17** (400 MHz, $CDCl_3$)

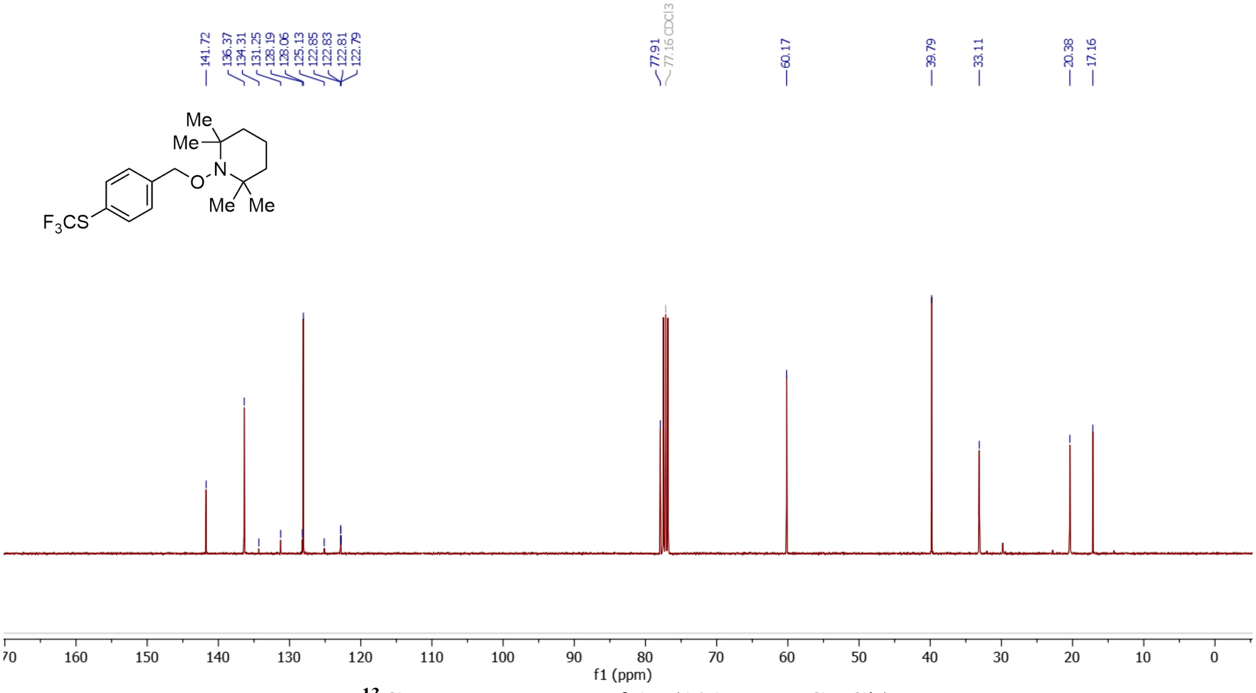

^{13}C NMR spectrum of **17** (101 MHz, $CDCl_3$)

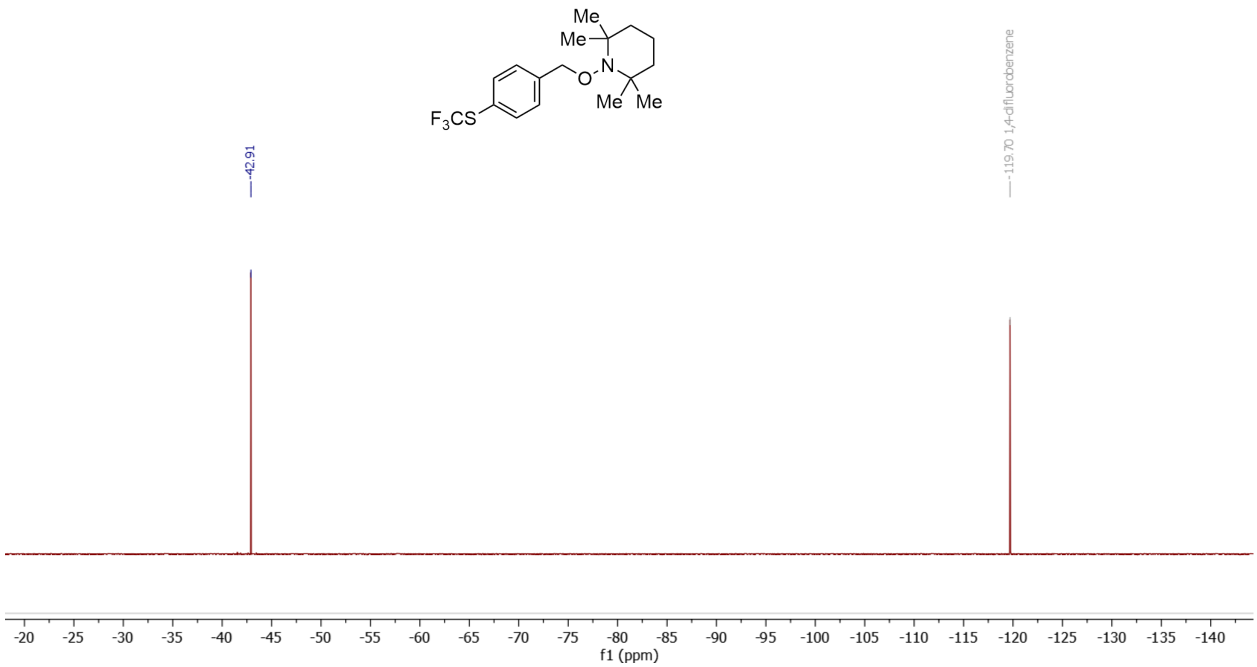

^{19}F NMR spectrum of **17** (376 MHz, $CDCl_3$), 1,4-difluorobenzene used as internal standard

N,N-diethyl-2-(pyridin-2-yl)-2-((2,2,6,6-tetramethylpiperidin-1-yl)oxy)ethan-1-amine (18)

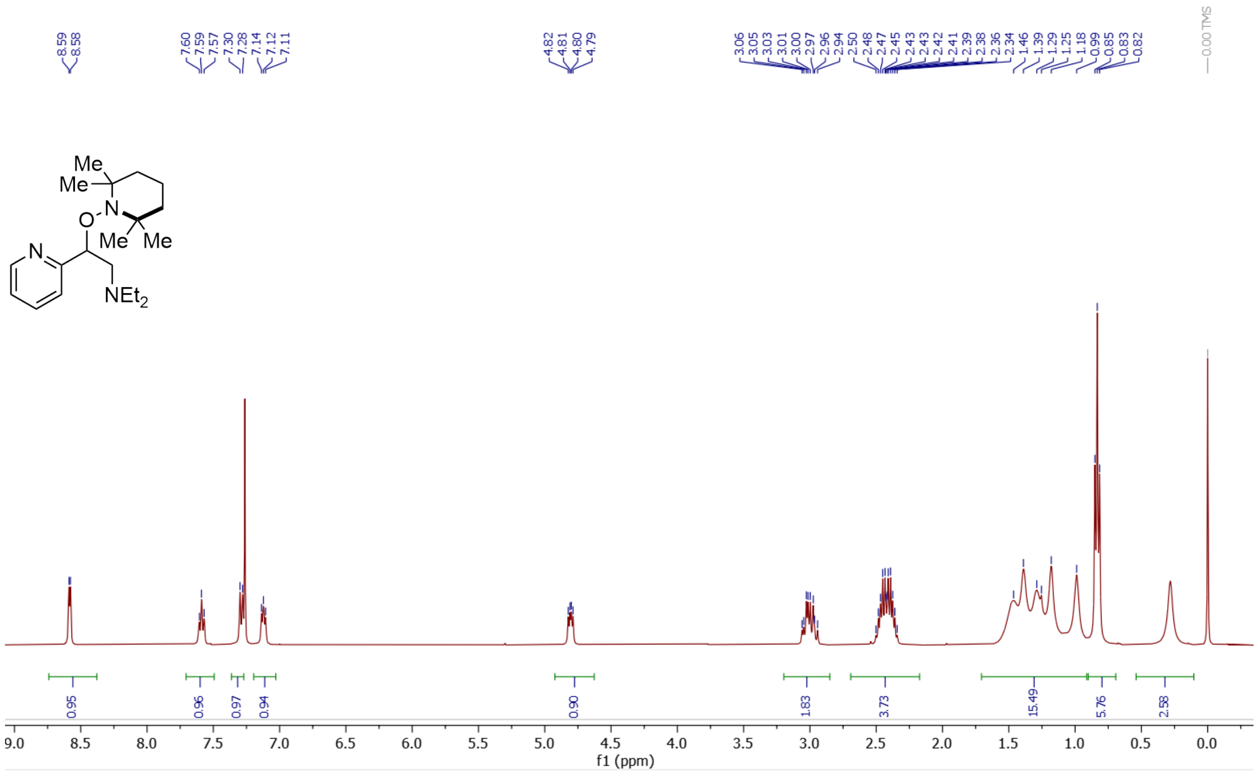

^{1}H NMR spectrum of **18** (400 MHz, $CDCl_3$)

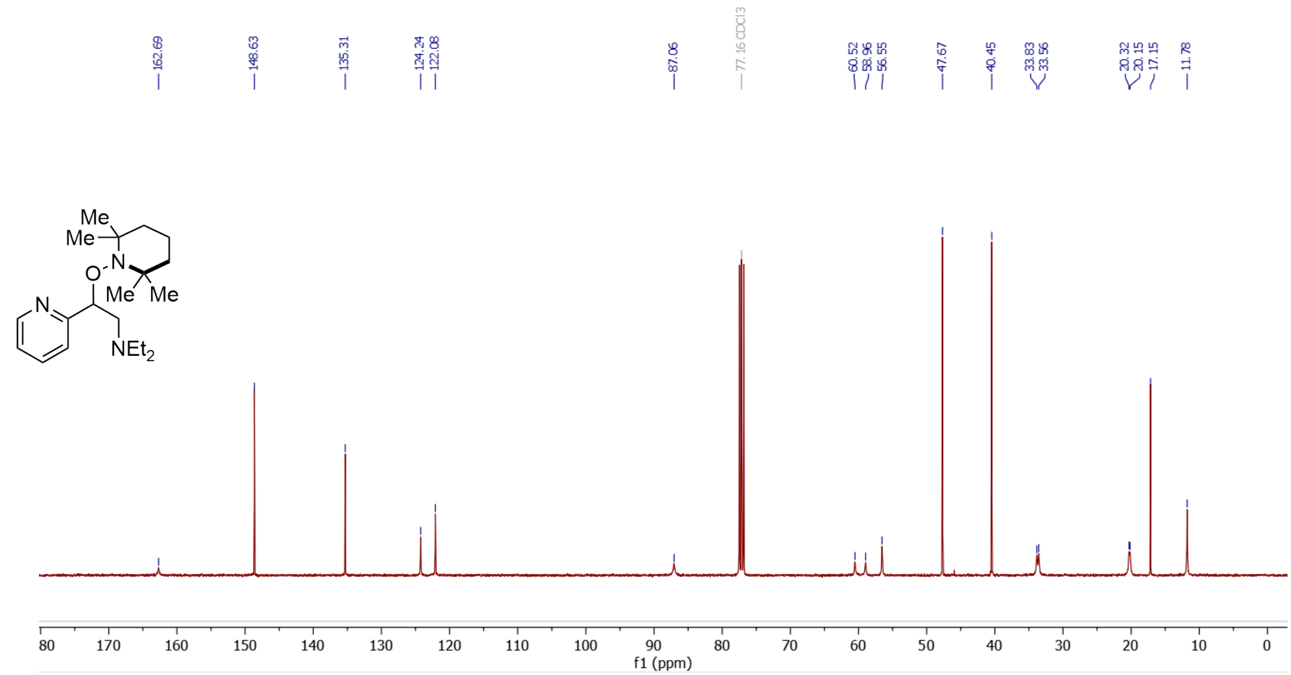

^{13}**C NMR** spectrum of **18** (101 MHz, $CDCl_3$)

3-(pyridin-4-yl)-3-((2,2,6,6-tetramethylpiperidin-1-yl)oxy)propan-1-amine (19)

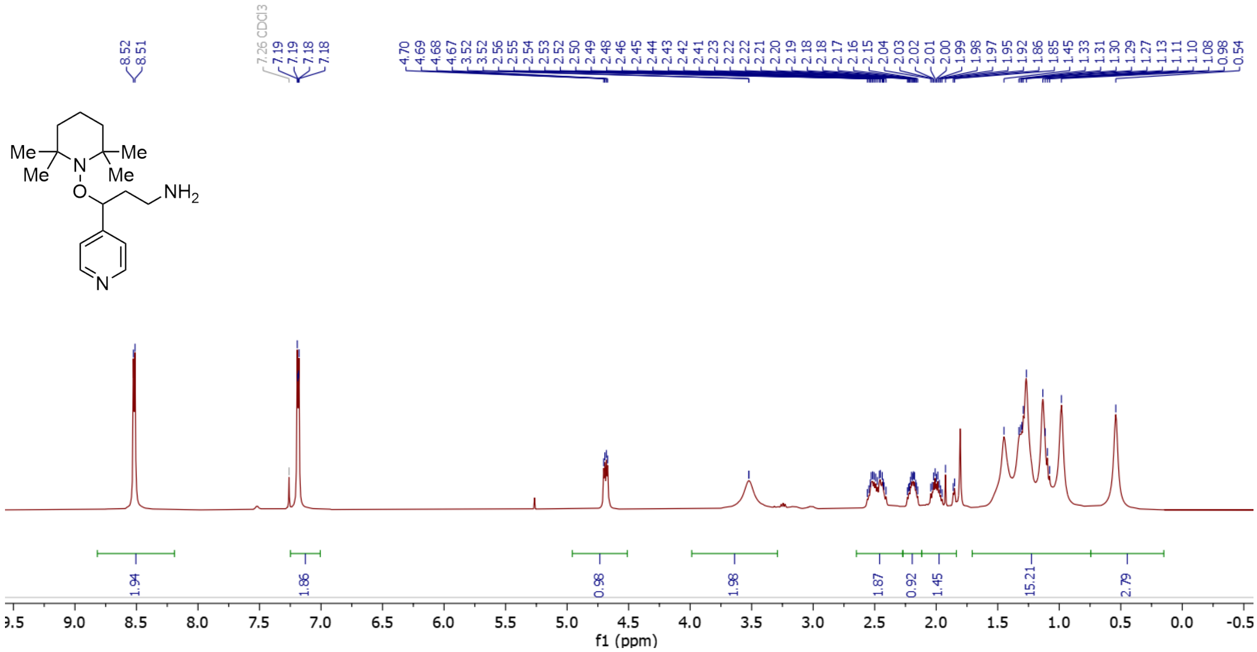

^{1}**H NMR** spectrum of **19** (400 MHz, $CDCl_3$)

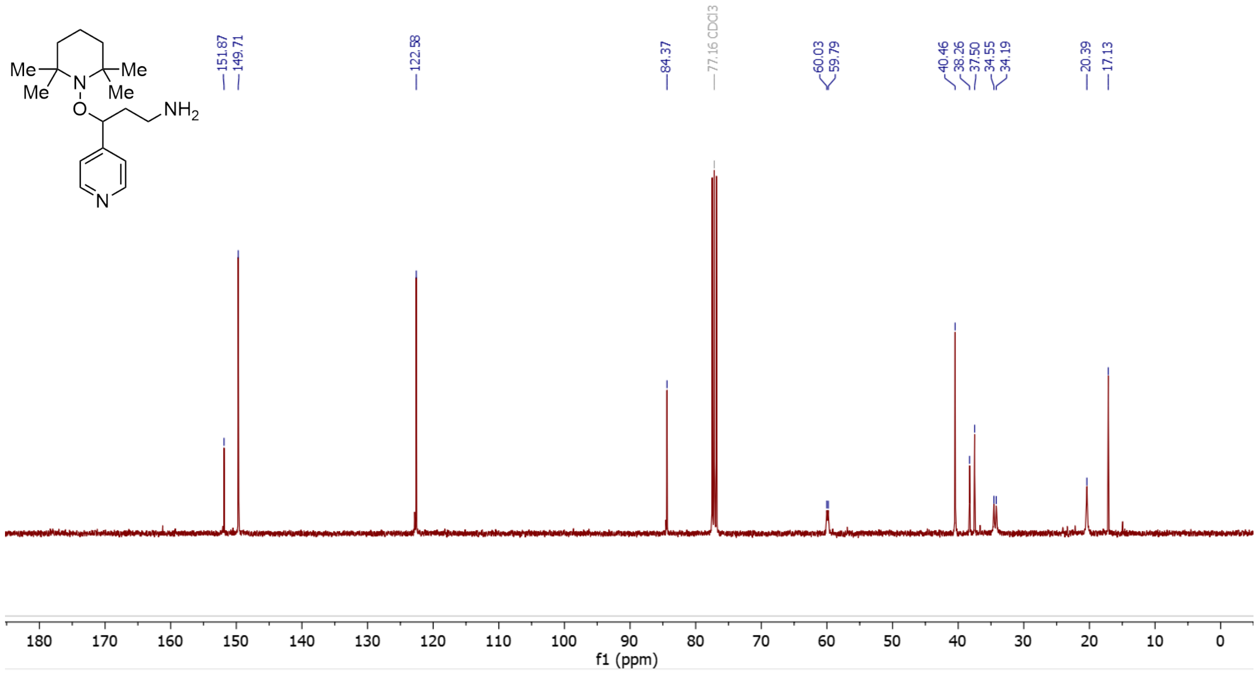

^{13}**C NMR** spectrum of **19** (101 MHz, $CDCl_3$)

3-((2,2,6,6-tetramethylpiperidin-1-yl)oxy)-3-(3-(trifluoromethyl)phenyl)propan-1-amine (20)

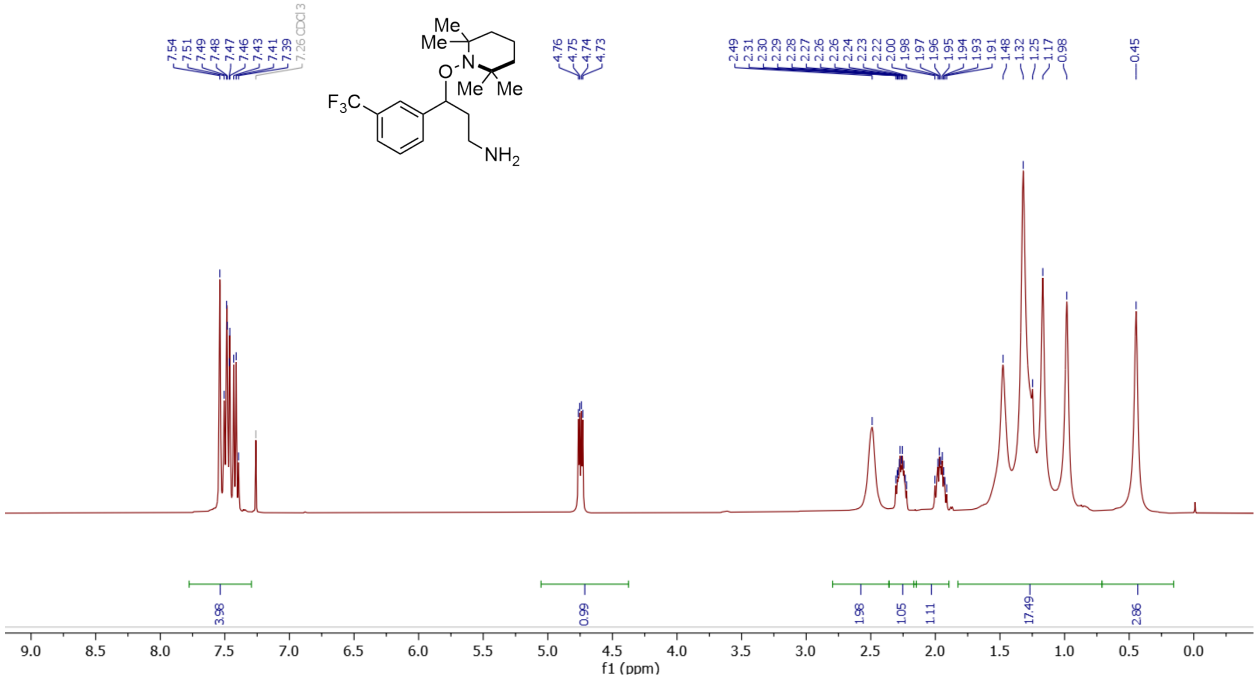

^{1}**H NMR** spectrum of **20** (400 MHz, $CDCl_3$)

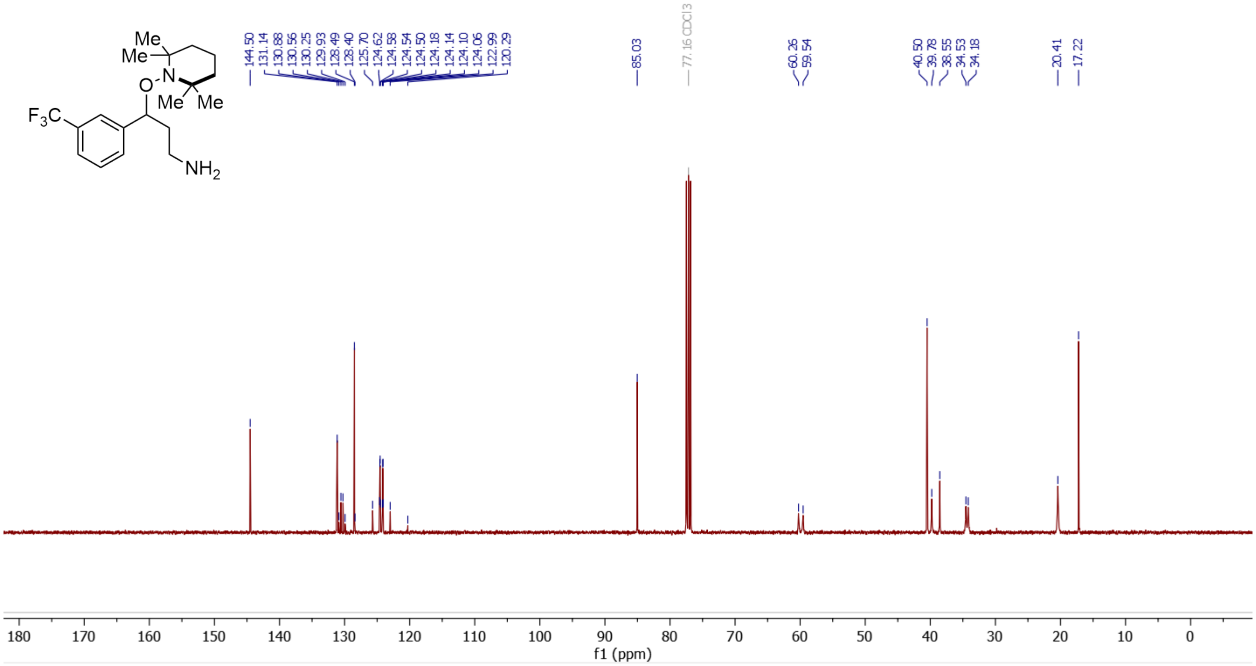

^{13}C NMR spectrum of **20** (101 MHz, $CDCl_3$)

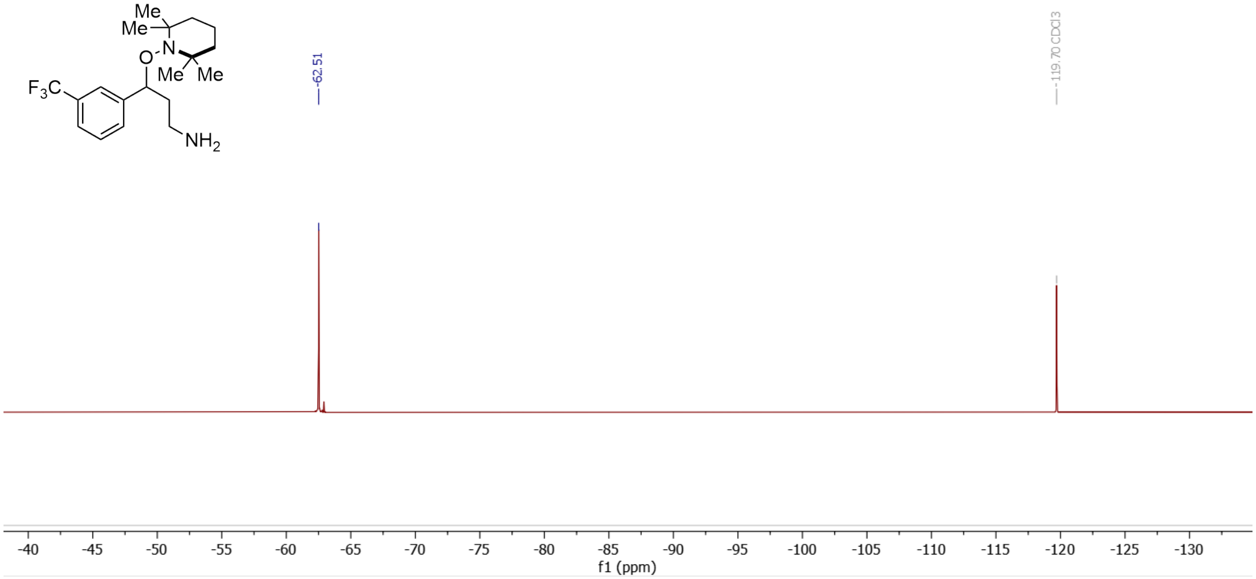

^{19}F NMR spectrum of **20** (376 MHz, $CDCl_3$), 1,4-difluorobenzene used as internal standard

2,2,6,6-tetramethyl-1-((4-(4,4,5,5-tetramethyl-1,3,2-dioxaborolan-2-yl)benzyl)oxy)piperidine (21)

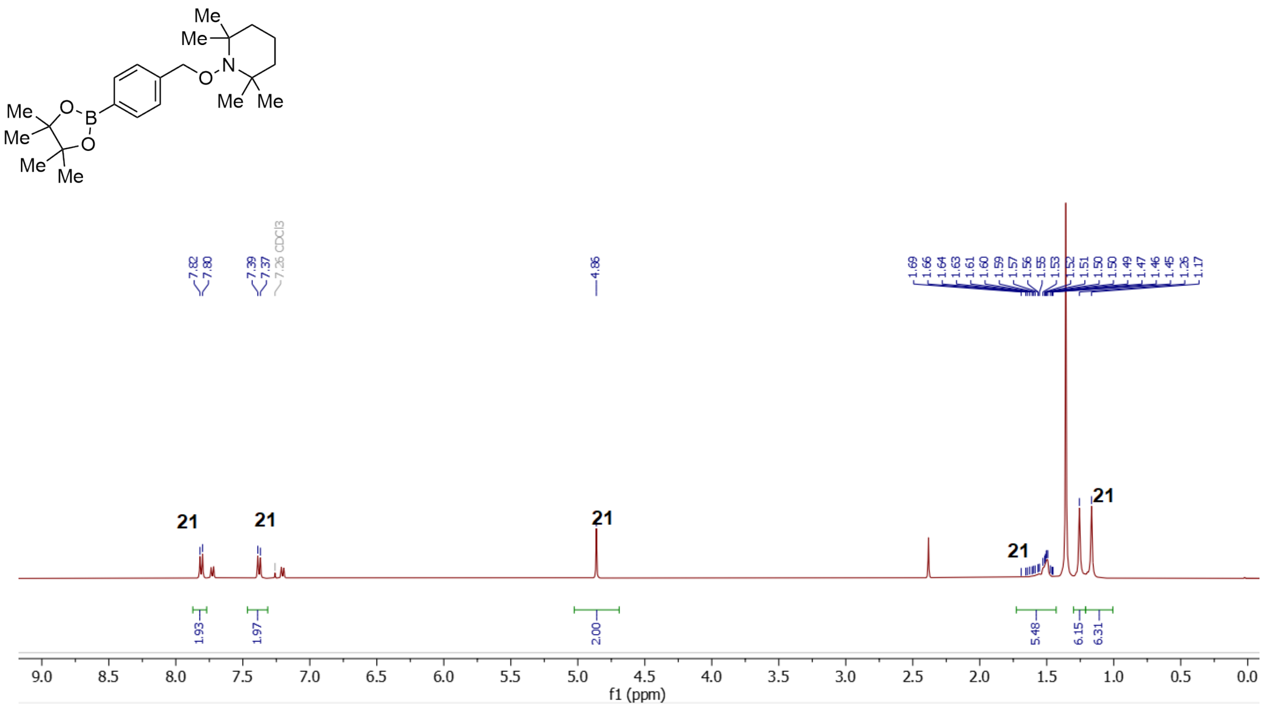

^{1}H NMR spectrum of **21** mixture with 4,4,5,5-tetramethyl-2-(p-tolyl)-1,3,2-dioxaborolane (400 MHz, $CDCl_3$)

3-bromo-4-(((2,2,6,6-tetramethylpiperidin-1-yl)oxy)methyl)pyridine (22)

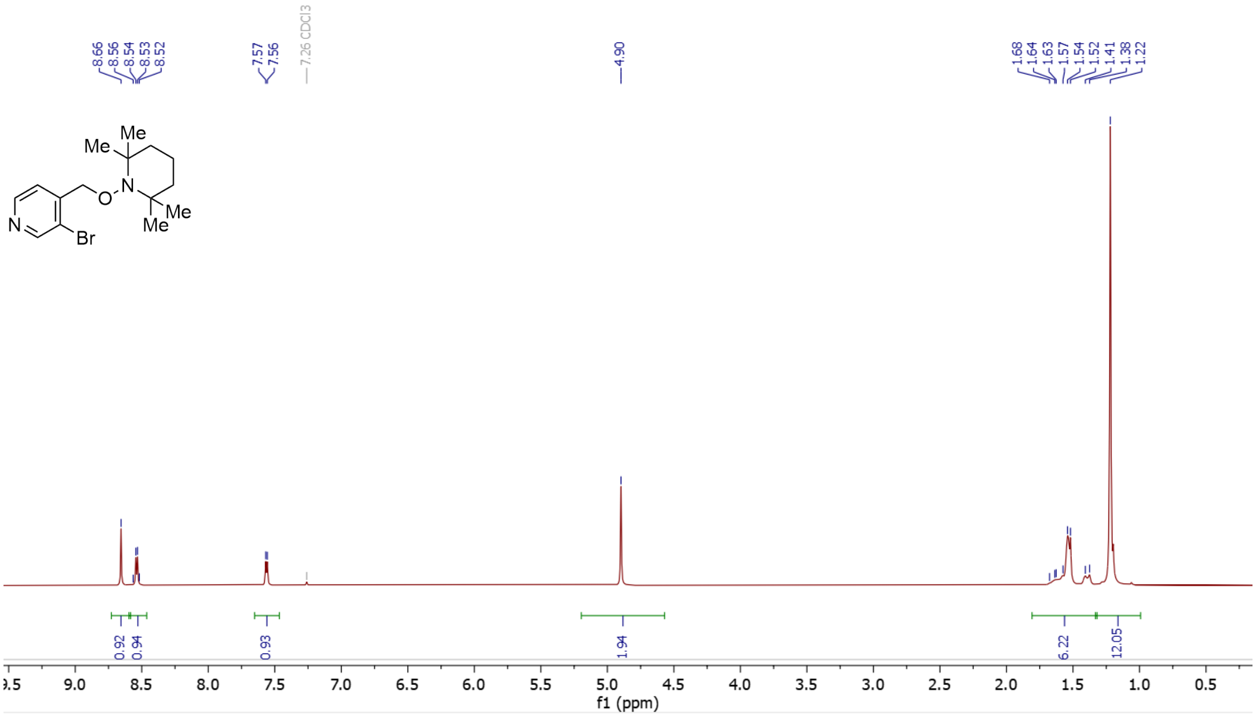

^{1}H NMR spectrum of **22** (400 MHz, $CDCl_3$)

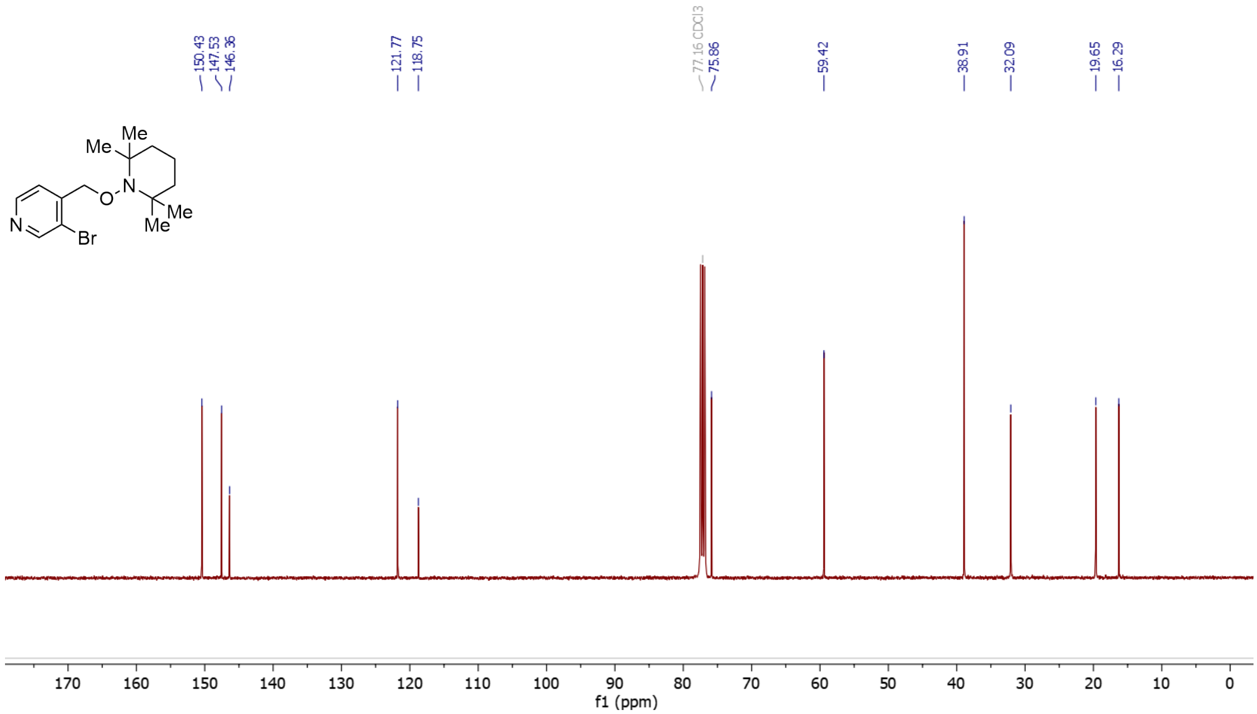

^{13}C **NMR** spectrum of **22** (101 MHz, $CDCl_3$)

(3*R*)-3-amino-1-(8-((2,2,6,6-tetramethylpiperidin-1-yl)oxy)-3-(trifluoromethyl)-5,6-dihydro-[1,2,4]triazolo[4,3-*a*]pyrazin-7(8*H*)-yl)-4-(2,4,5-trifluorophenyl)butan-1-one (23)

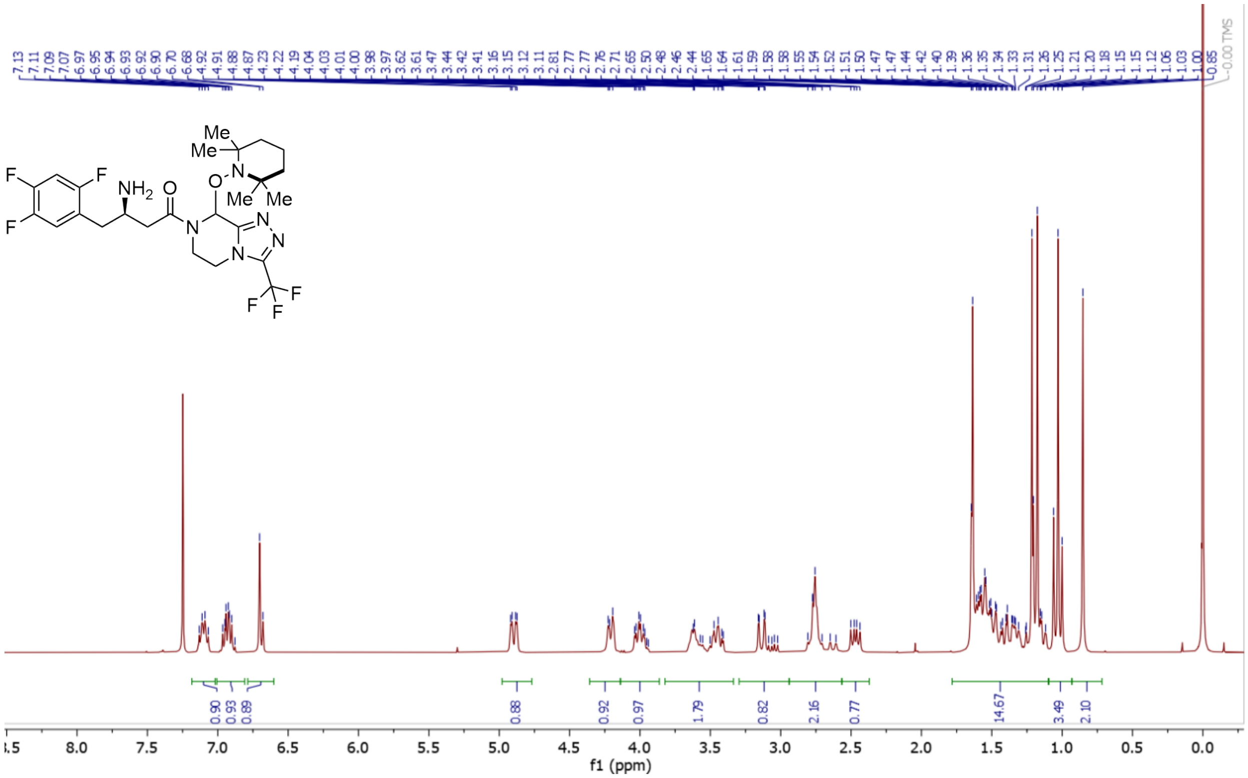

^{1}H **NMR** spectrum of **23** (mixture of diastereomers) (400 MHz, $CDCl_3$)

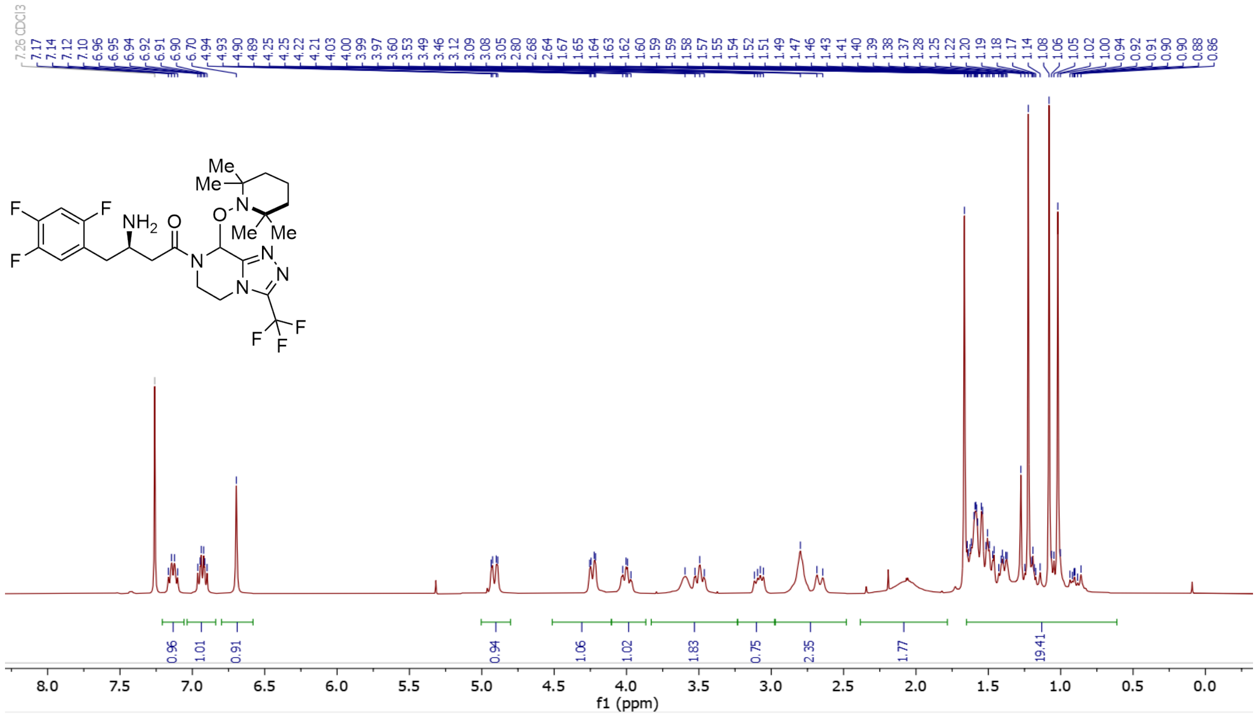

¹H NMR spectrum of **23** (diastereomer 1) (400 MHz, $CDCl_3$)

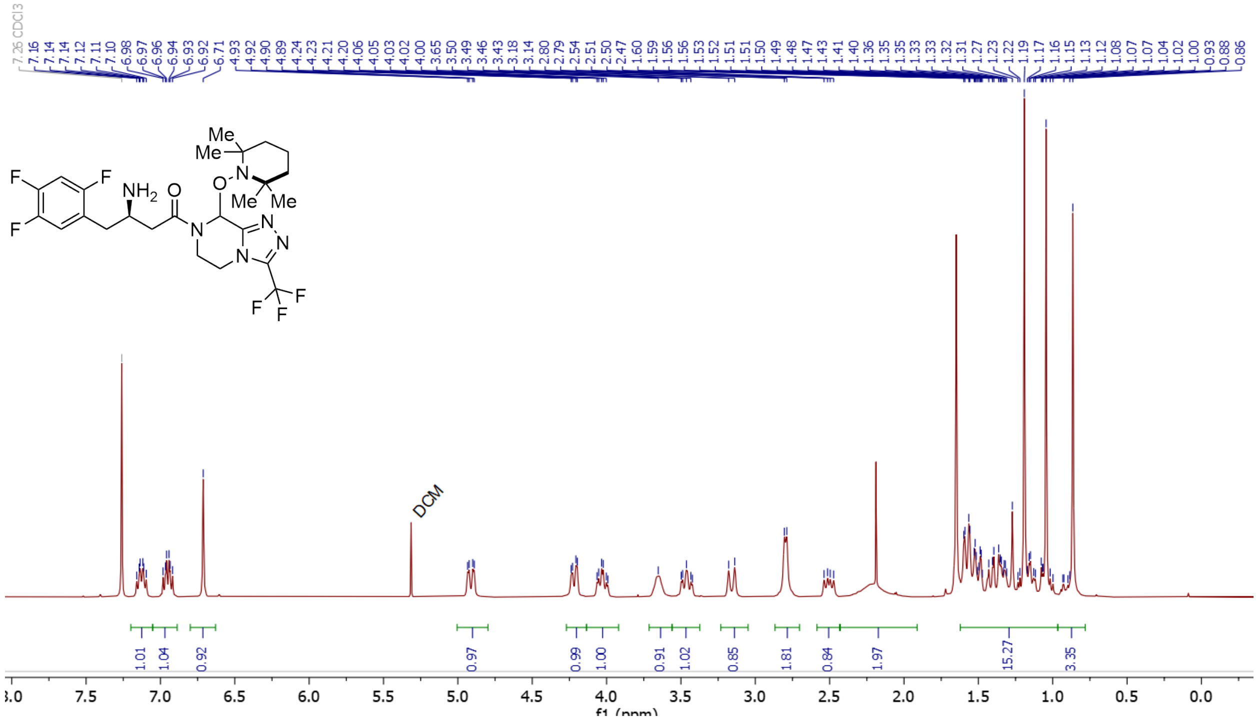

¹H NMR spectrum of **23** (diastereomer 2) (400 MHz, $CDCl_3$)

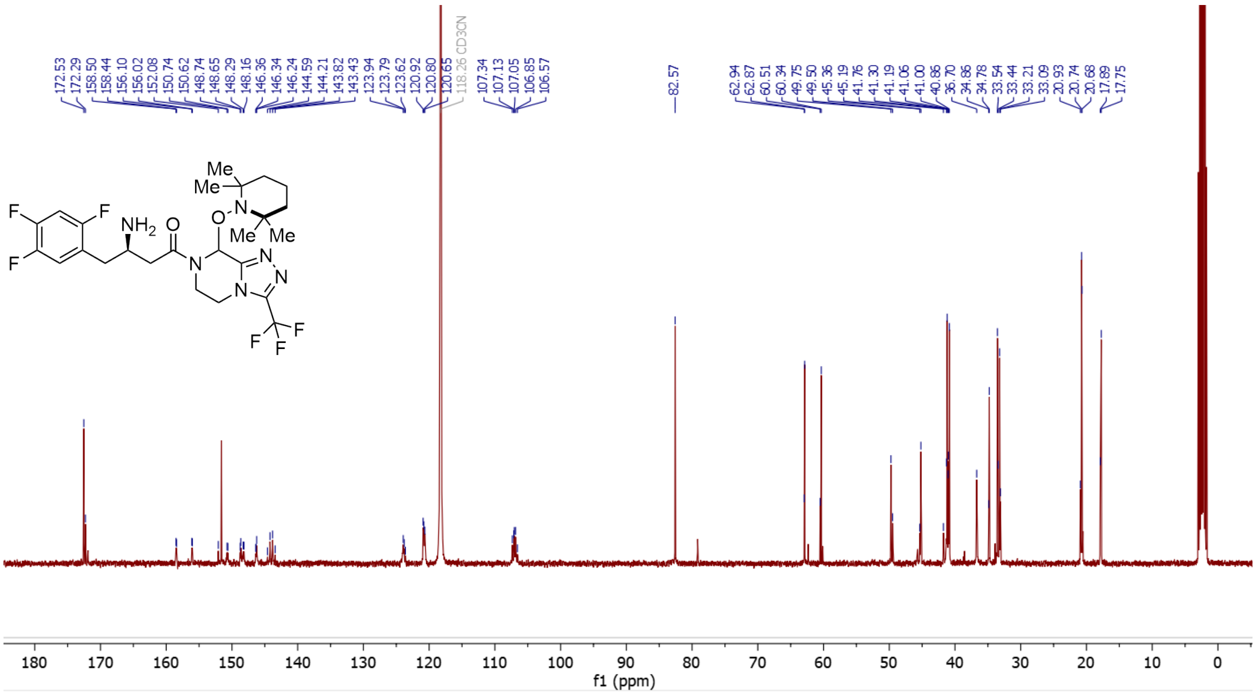

^{13}C NMR spectrum of **23** (mixture of diastereomers) (101 MHz, CD_3CN)

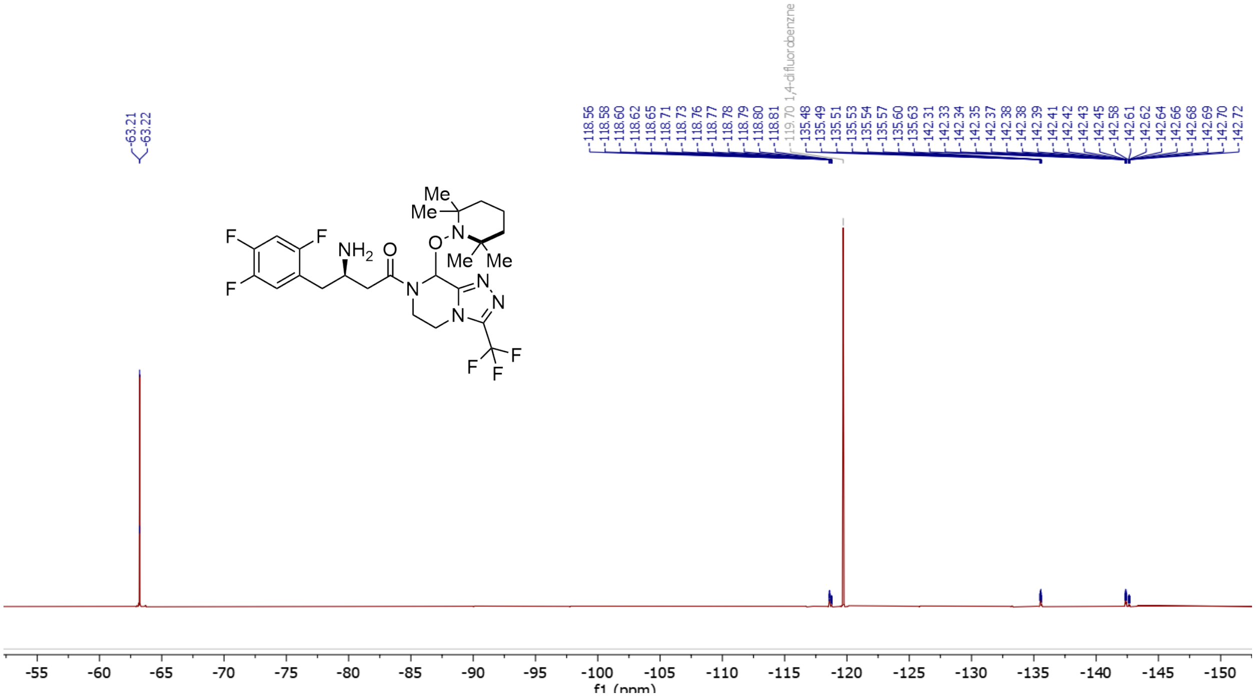

^{19}F NMR spectrum of **23** (mixture of diastereomers) (376 MHz, $CDCl_3$), 1,4-difluorobenzene used as internal standard

N-((R)-1-(naphthalen-1-yl)ethyl)-3-((2,2,6,6-tetramethylpiperidin-1-yl)oxy)-3-(3-(trifluoromethyl)phenyl)propan-1-amine (24)

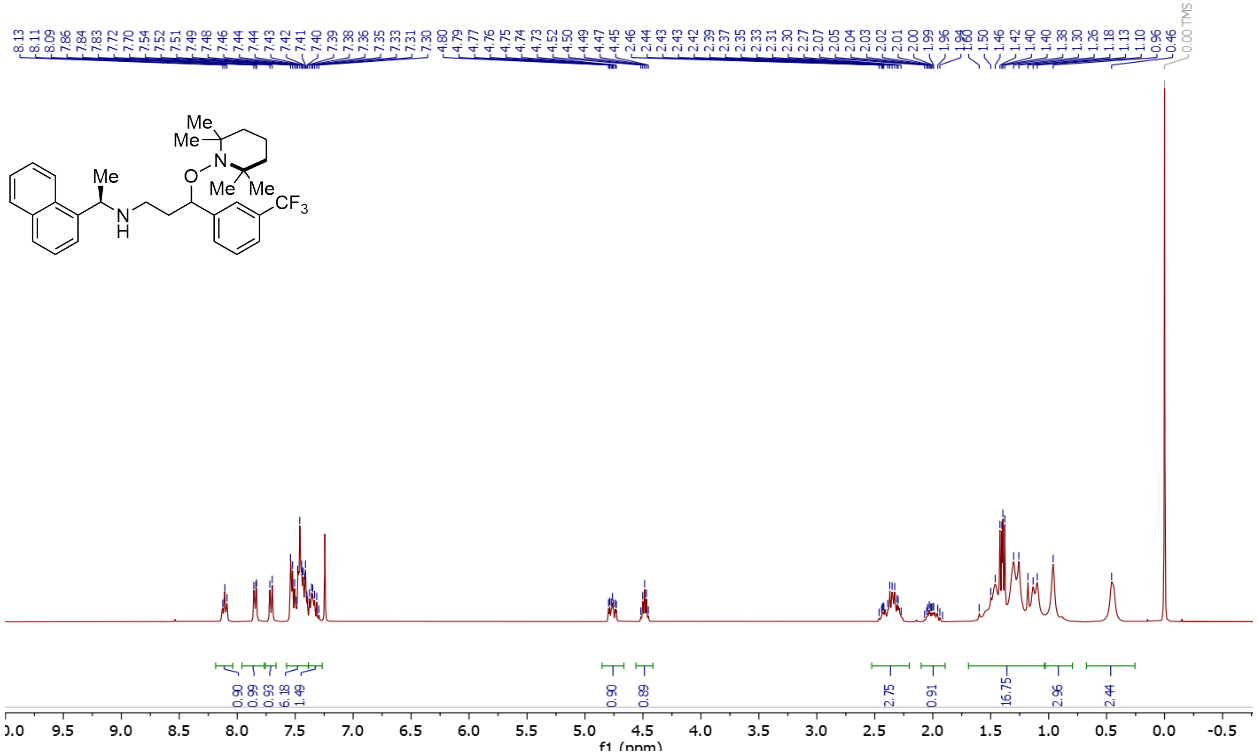

^{1}H NMR spectrum of **24** (400 MHz, $CDCl_3$)

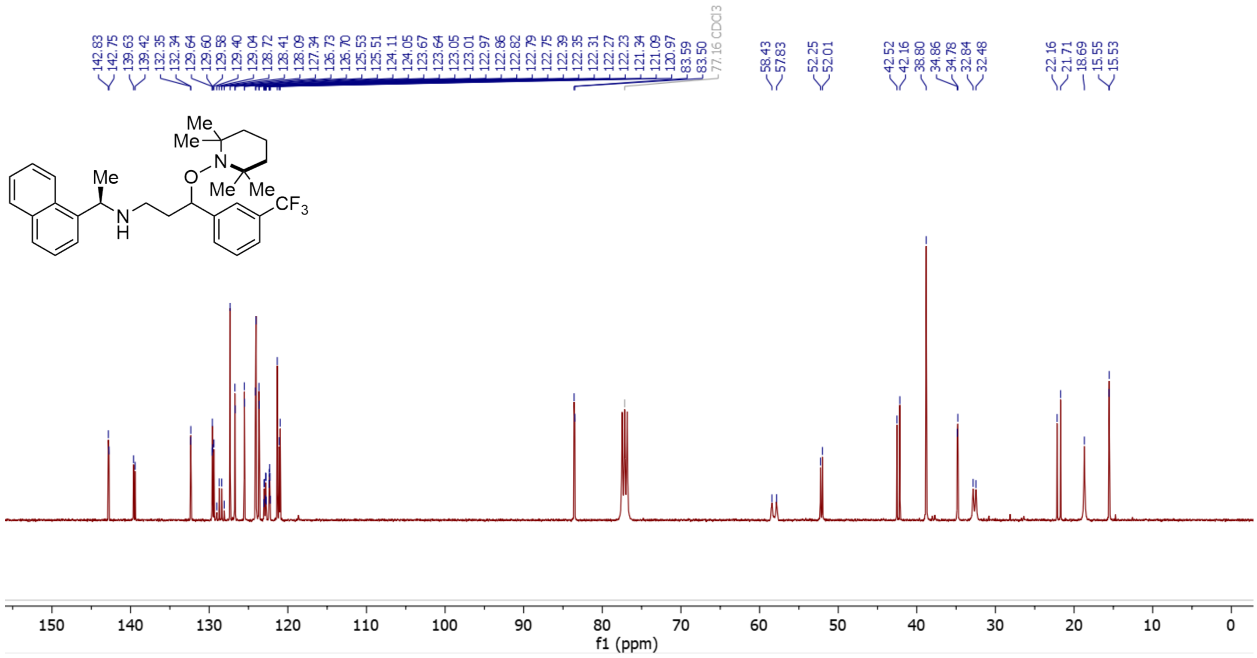

^{13}C NMR spectrum of **24** (101 MHz, $CDCl_3$)

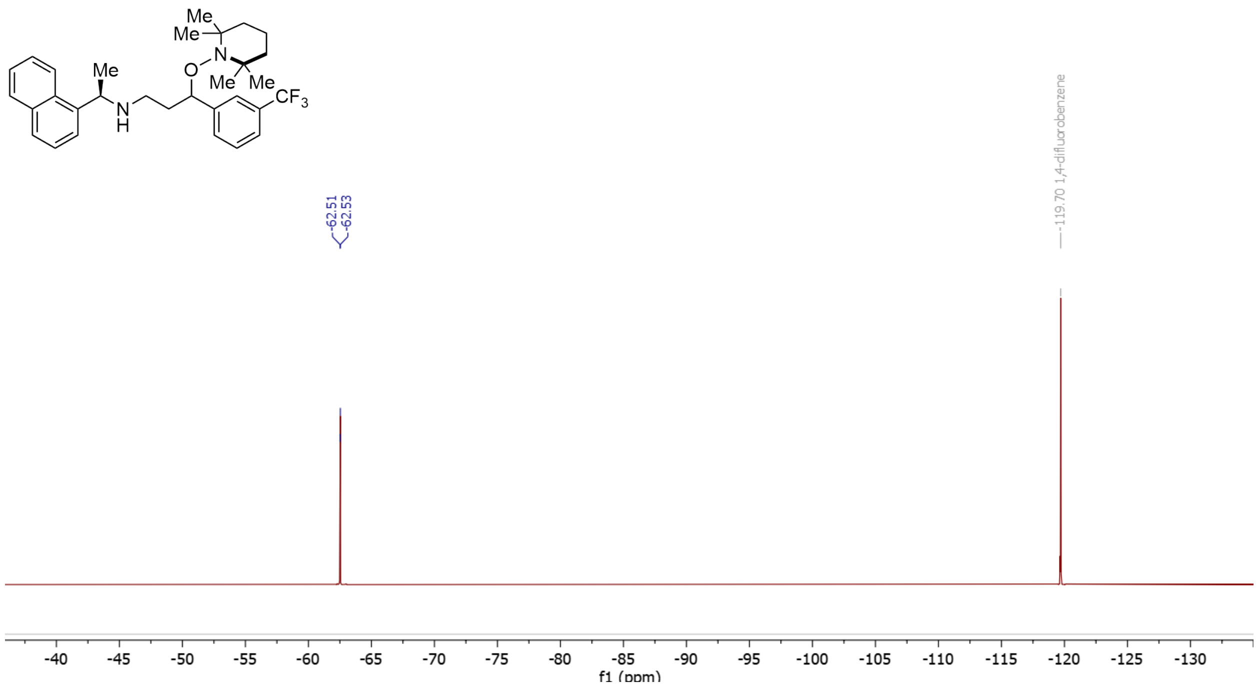

^{19}F NMR spectrum of **24** (376 MHz, $CDCl_3$), 1,4-difluorobenzene used as internal standard

(4-methoxyphenyl)(5-nitro-2-(1-((2,2,6,6-tetramethylpiperidin-1-yl)oxy)butyl)benzofuran-3-yl)methanone (25)

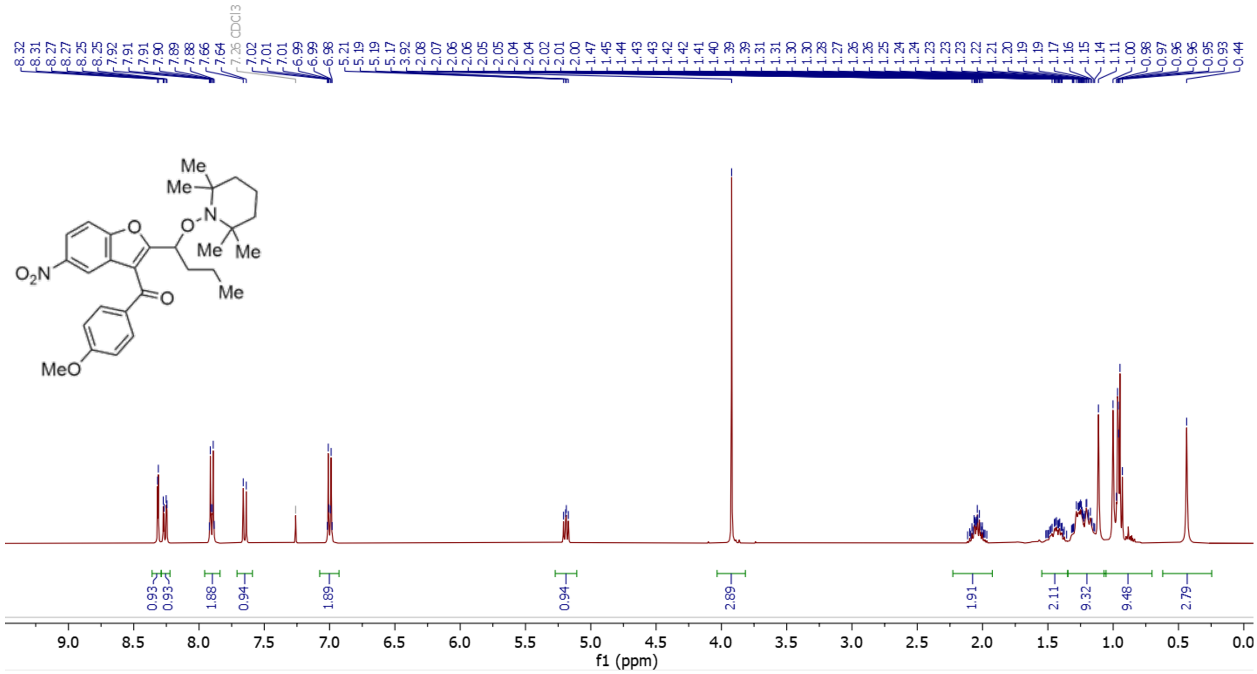

^{1}H NMR spectrum of **25** (400 MHz, $CDCl_3$)

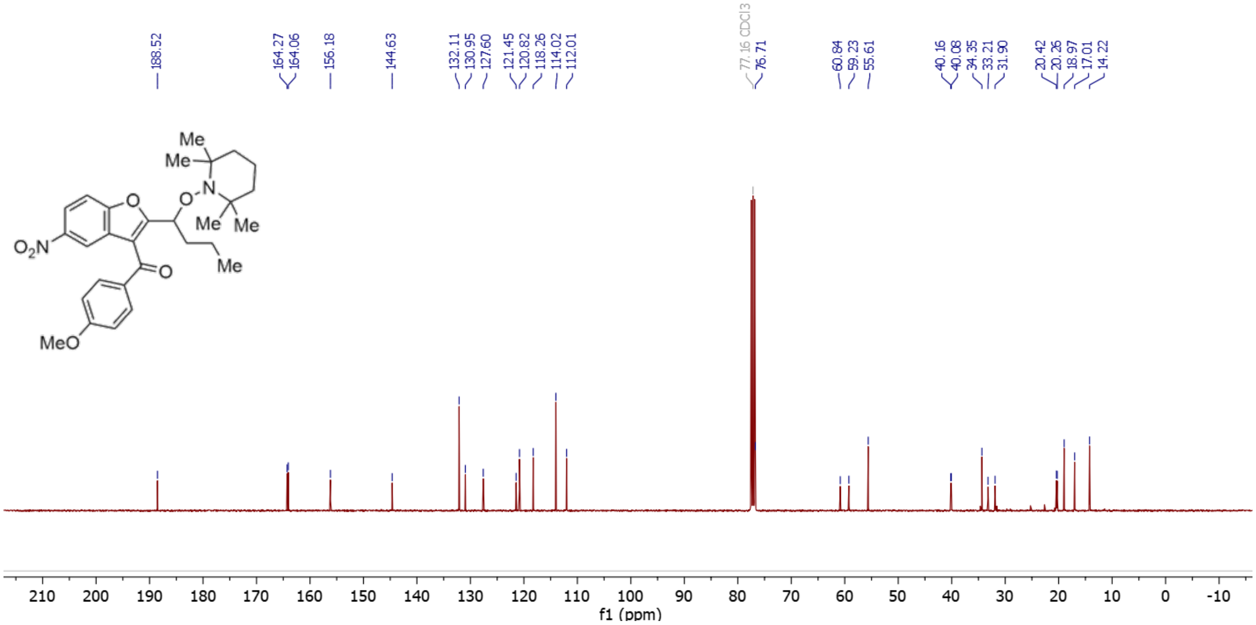

^{13}**C NMR** spectrum of **25** (101 MHz, $CDCl_3$)

1-methyl-3-(3-(((2,2,6,6-tetramethylpiperidin-1-yl)oxy)methyl)-4-(4-((trifluoromethyl)thio)phenoxy)phenyl)-1,3,5-triazinane-2,4,6-trione (26)

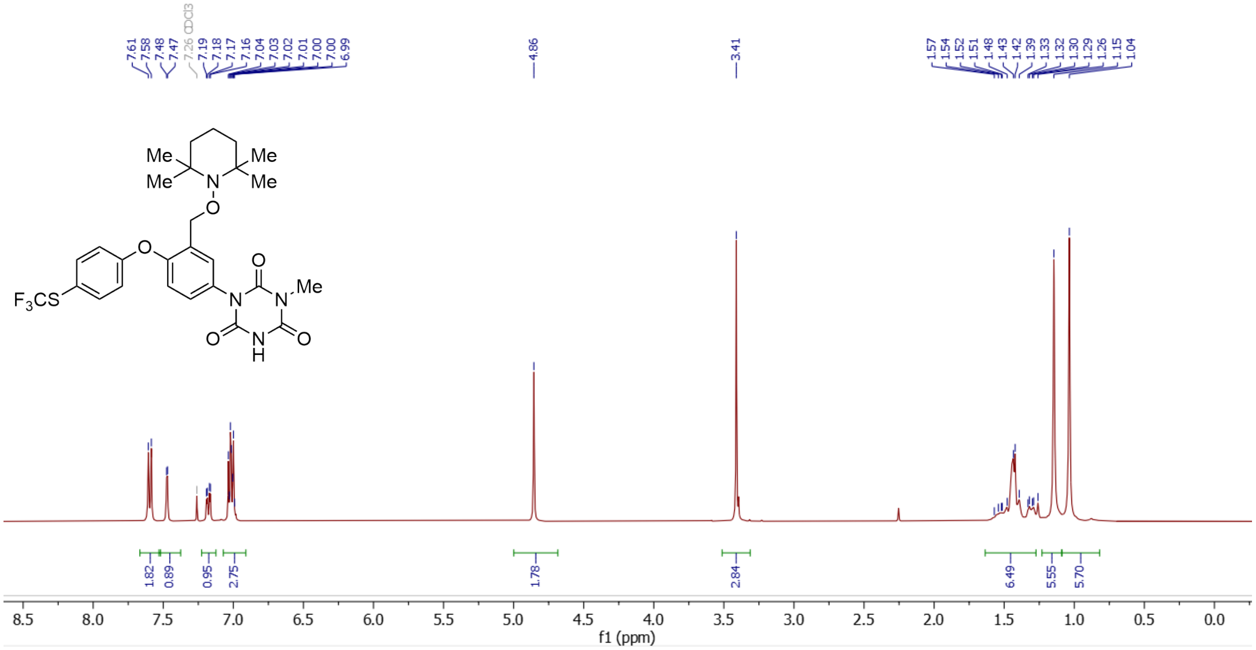

^{1}**H NMR** spectrum of **26** (400 MHz, $CDCl_3$)

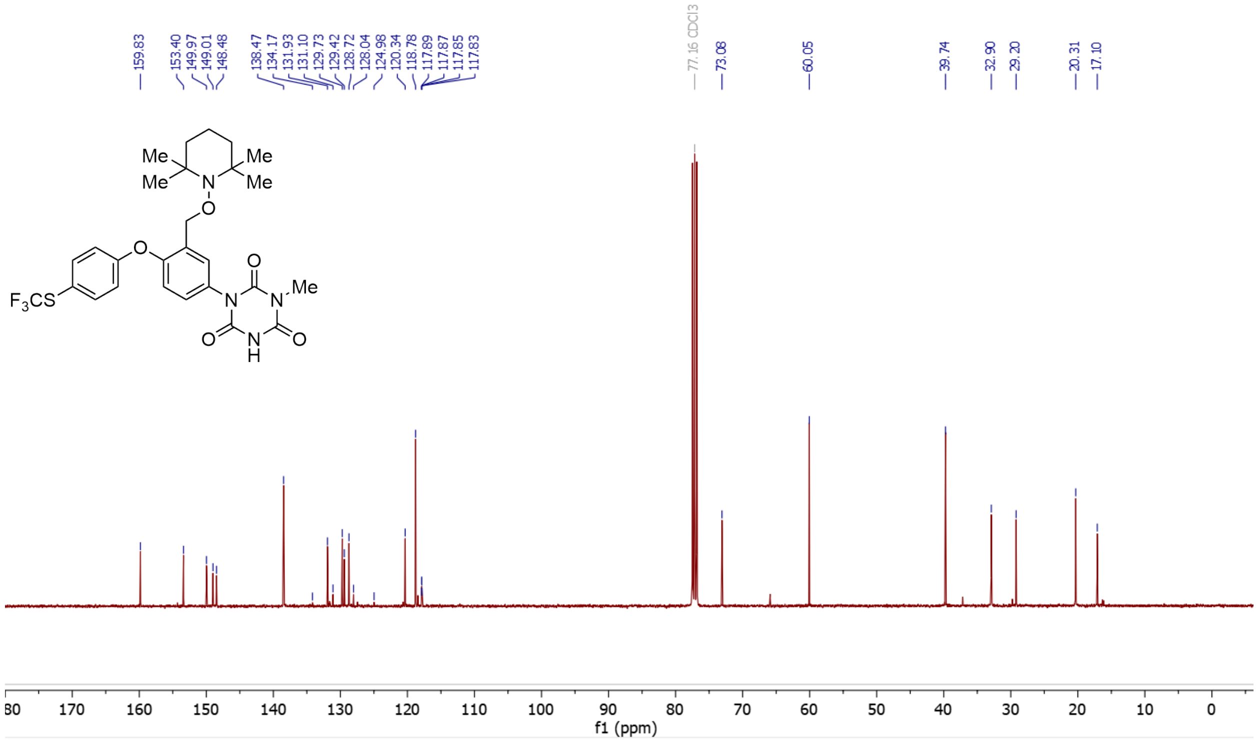

^{13}**C NMR** spectrum of **26** (101 MHz, $CDCl_3$)

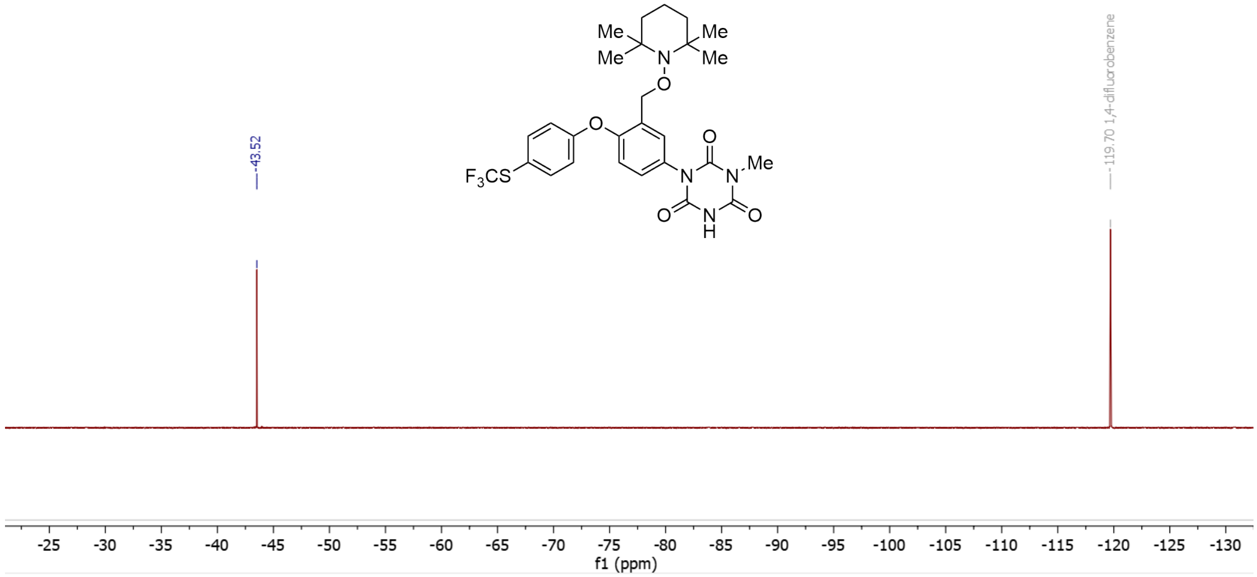

^{19}**F NMR** spectrum of **26** (376 MHz, $CDCl_3$), 1,4-difluorobenzene used as internal standard

(2*S*,3*R*,4*R*,5*S*,6*R*)-2-(4-chloro-3-((4-(((S)-tetrahydrofuran-3-yl)oxy)phenyl)((2,2,6,6-tetramethylpiperidin-1-yl)oxy)methyl)phenyl)-6-(hydroxymethyl)tetrahydro-2*H*-pyran-3,4,5-triol (27)

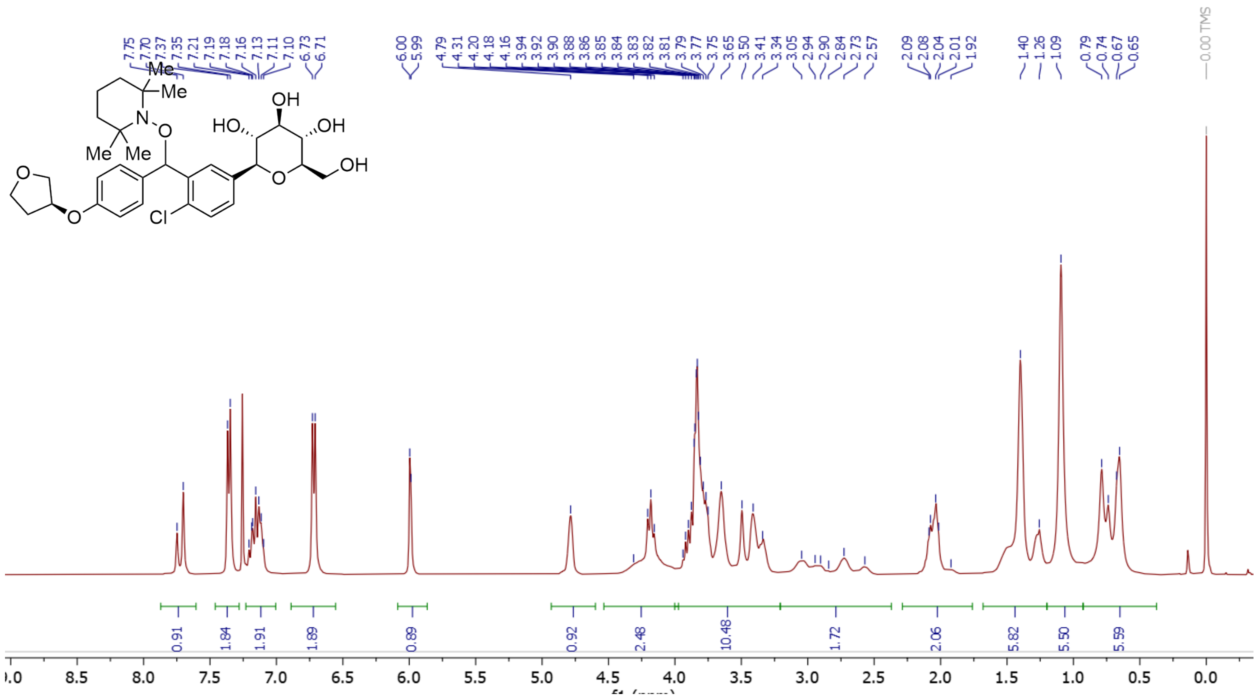

¹H NMR spectrum of **27** (mixture of diastereomers) (400 MHz, $CDCl_3$)

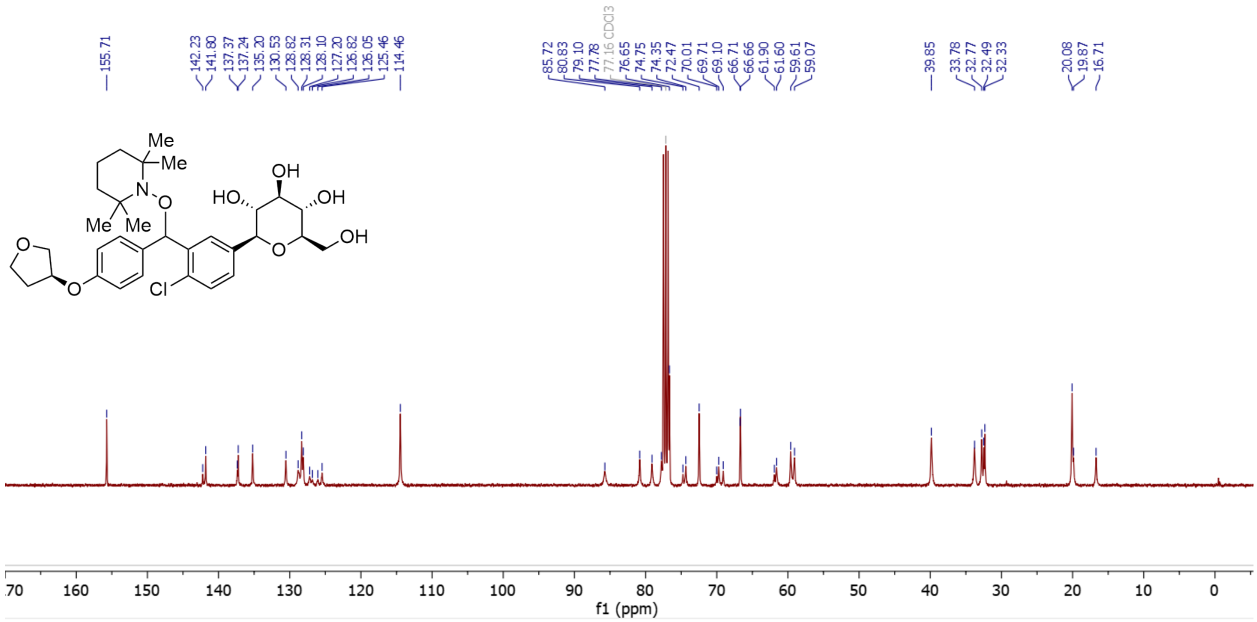

¹³C NMR spectrum of **27** (mixture of diastereomers) (101 MHz, $CDCl_3$)

1-((2-bromo-5-methylphenyl)(5-(4-fluorophenyl)thiophen-2-yl)methoxy)-2,2,6,6-tetramethylpiperidine (28)

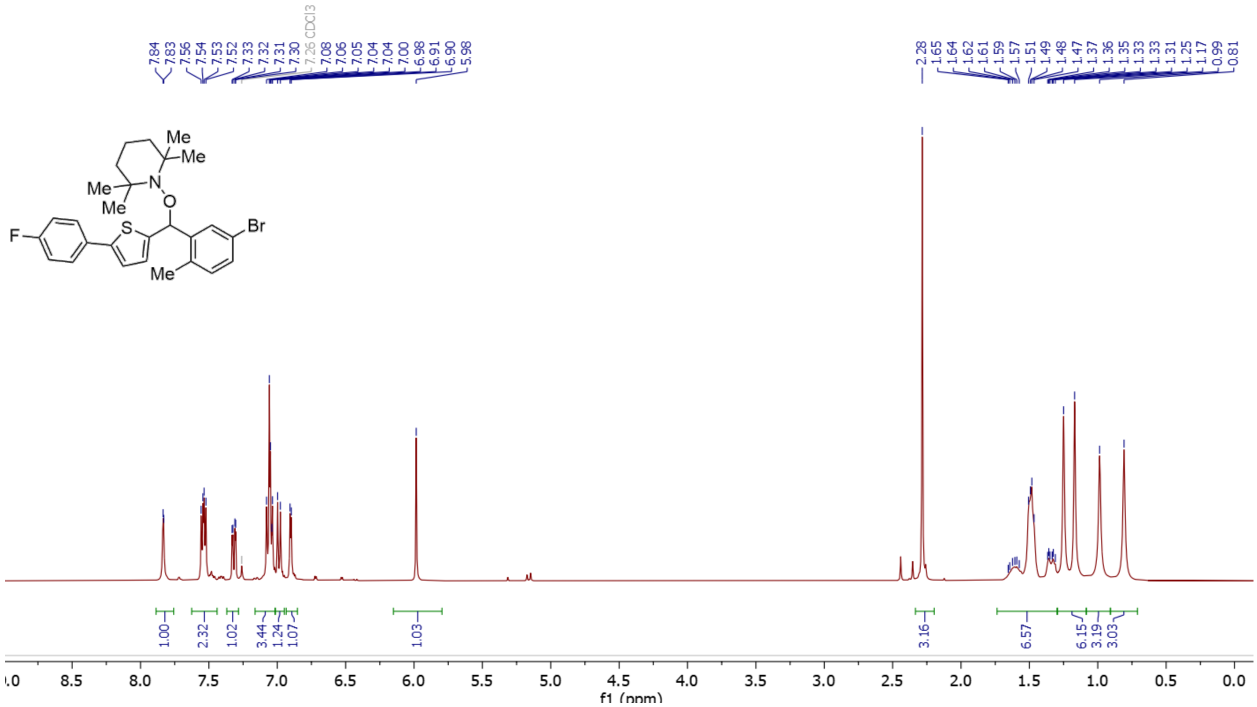

^{1}**H NMR** spectrum of **28** (400 MHz, $CDCl_3$)

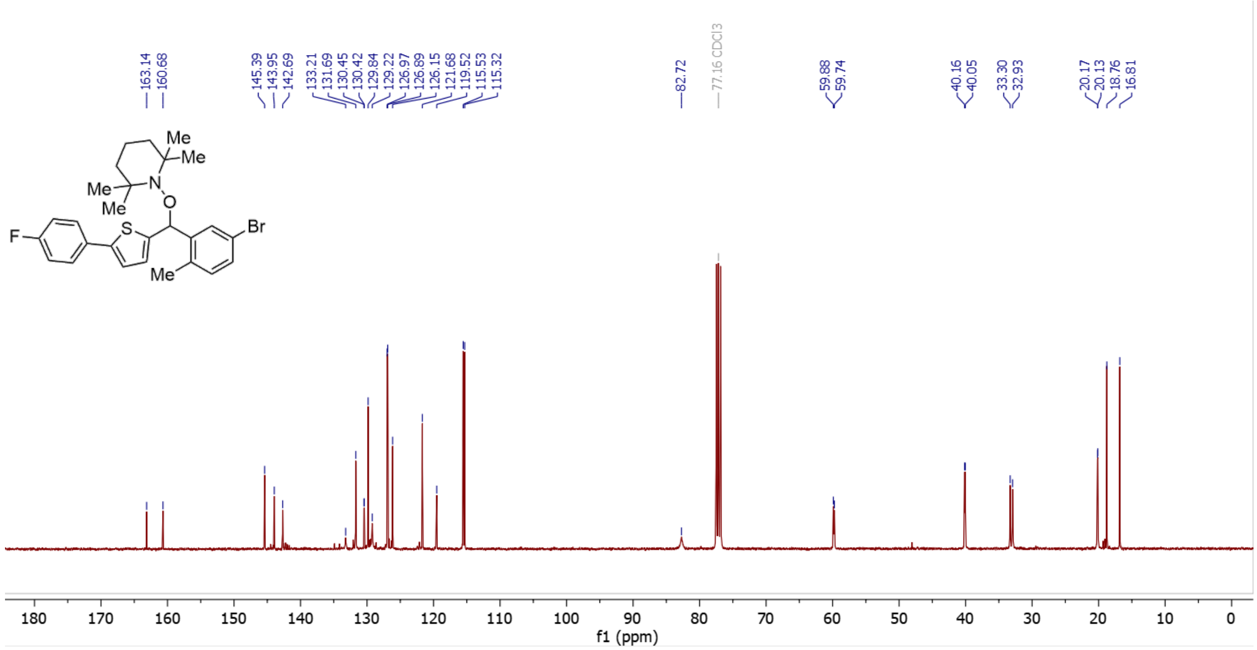

^{13}**C NMR** spectrum of **28** (101 MHz, $CDCl_3$)

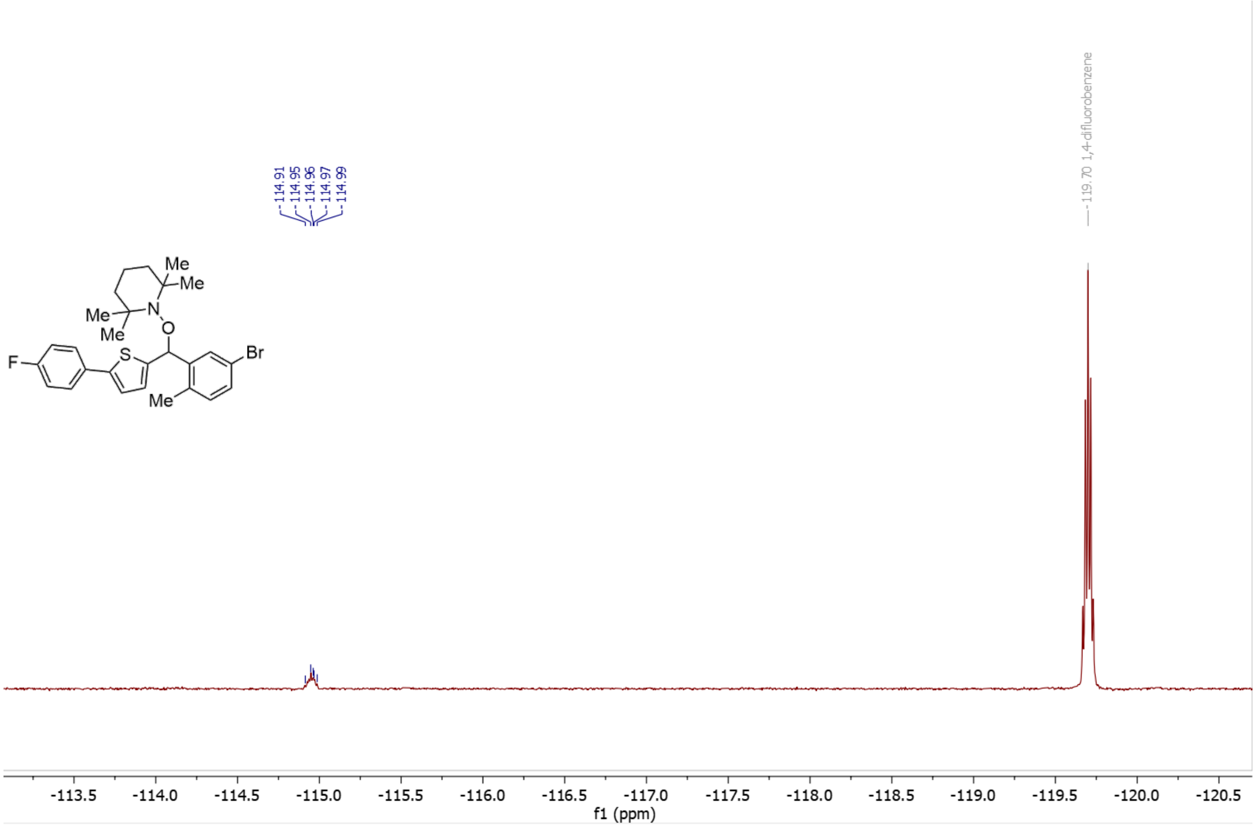

^{19}**F NMR** spectrum of **28** (376 MHz, $CDCl_3$), 1,4-difluorobenzene used as internal standard

4-((4-methoxyphenyl)((2,2,6,6-tetramethylpiperidin-1-yl)oxy)methyl)-5,6,7,8-tetrahydroquinoline (29)

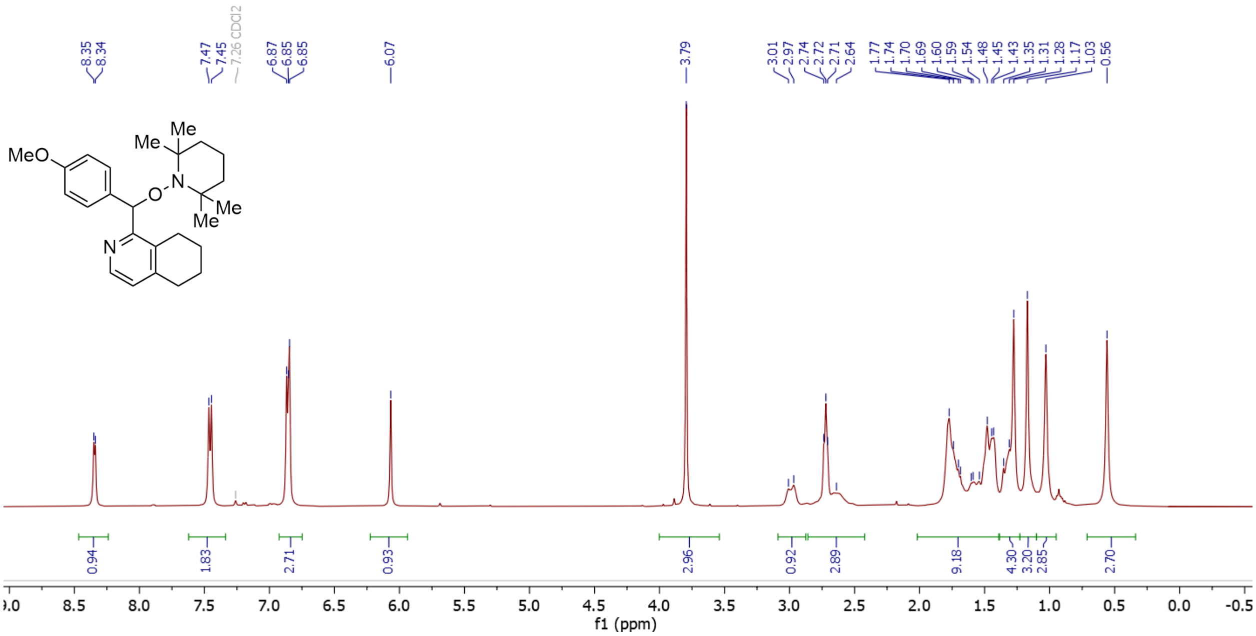

^{1}**H NMR** spectrum of **29** (400 MHz, $CDCl_3$)

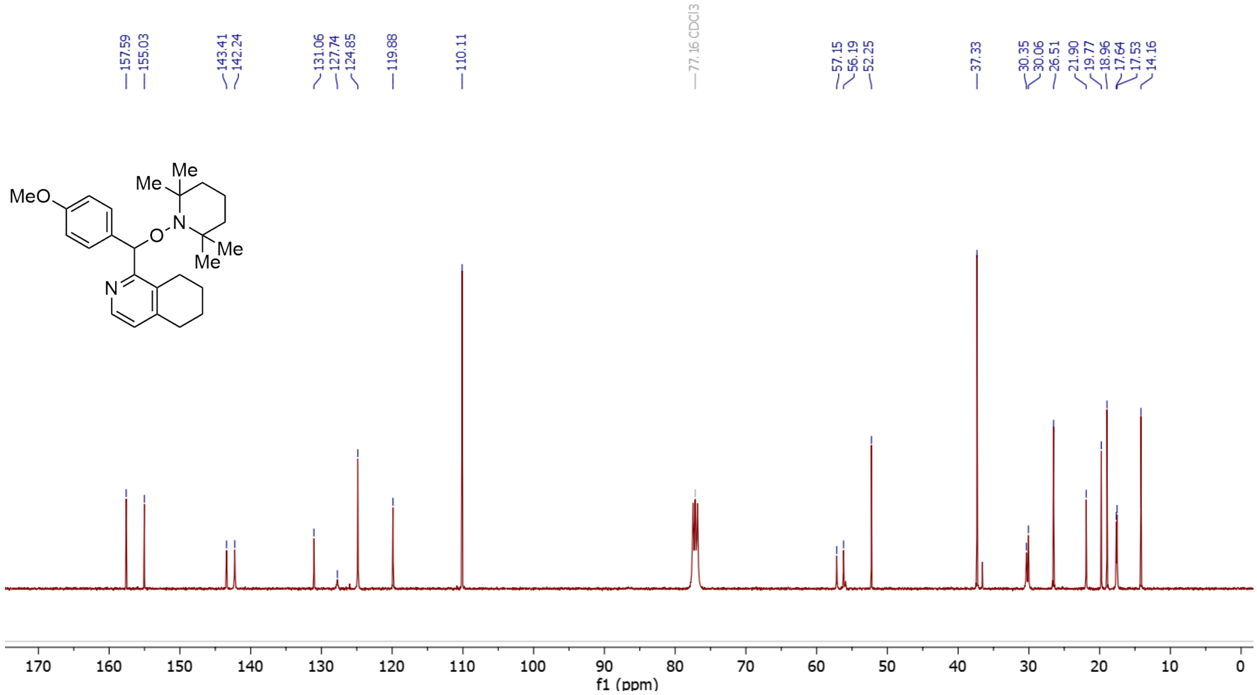

^{13}C NMR spectrum of **29** (101 MHz, $CDCl_3$)

5-ethyl-2-(((2,2,6,6-tetramethylpiperidin-1-yl)oxy)methyl)pyridine (30)

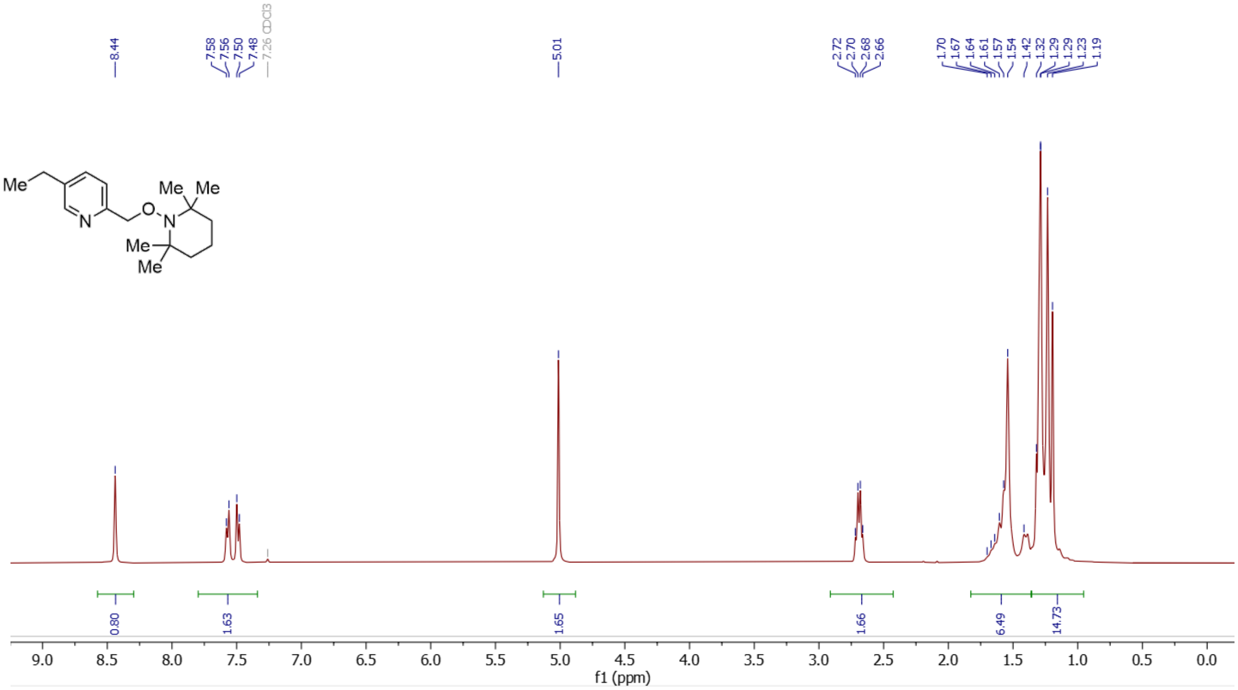

^{1}H NMR spectrum of **30** (400 MHz, $CDCl_3$)

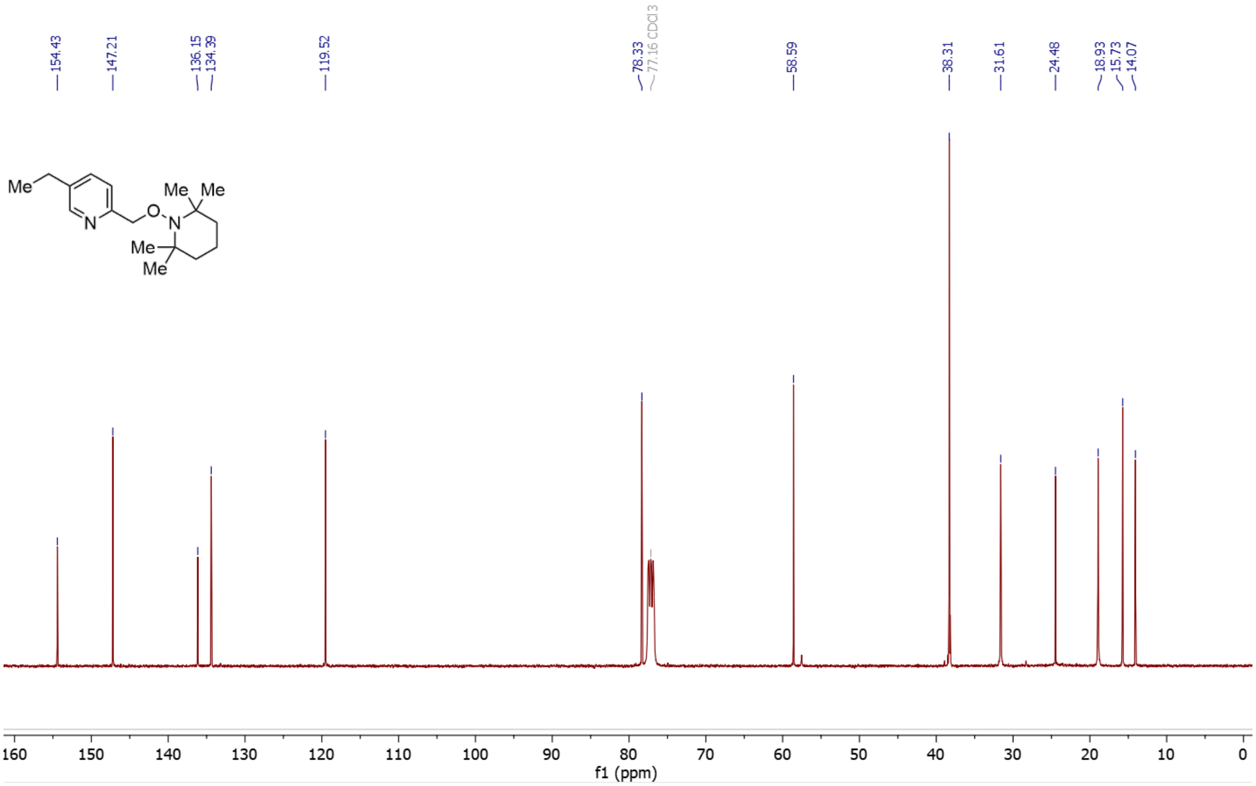

^{13}C NMR spectrum of **30** (101 MHz, $CDCl_3$)

5-methyl-2-(((2,2,6,6-tetramethylpiperidin-1-yl)oxy)methyl)pyridine (31)

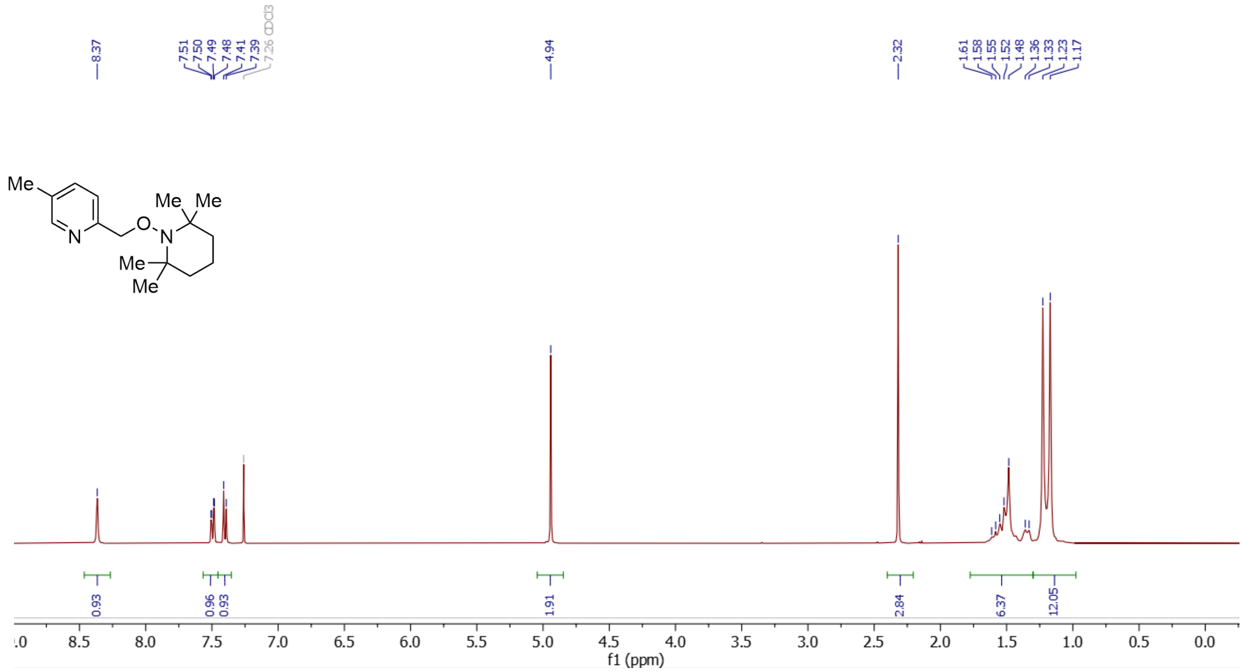

^{1}H NMR spectrum of **31** (400 MHz, $CDCl_3$)

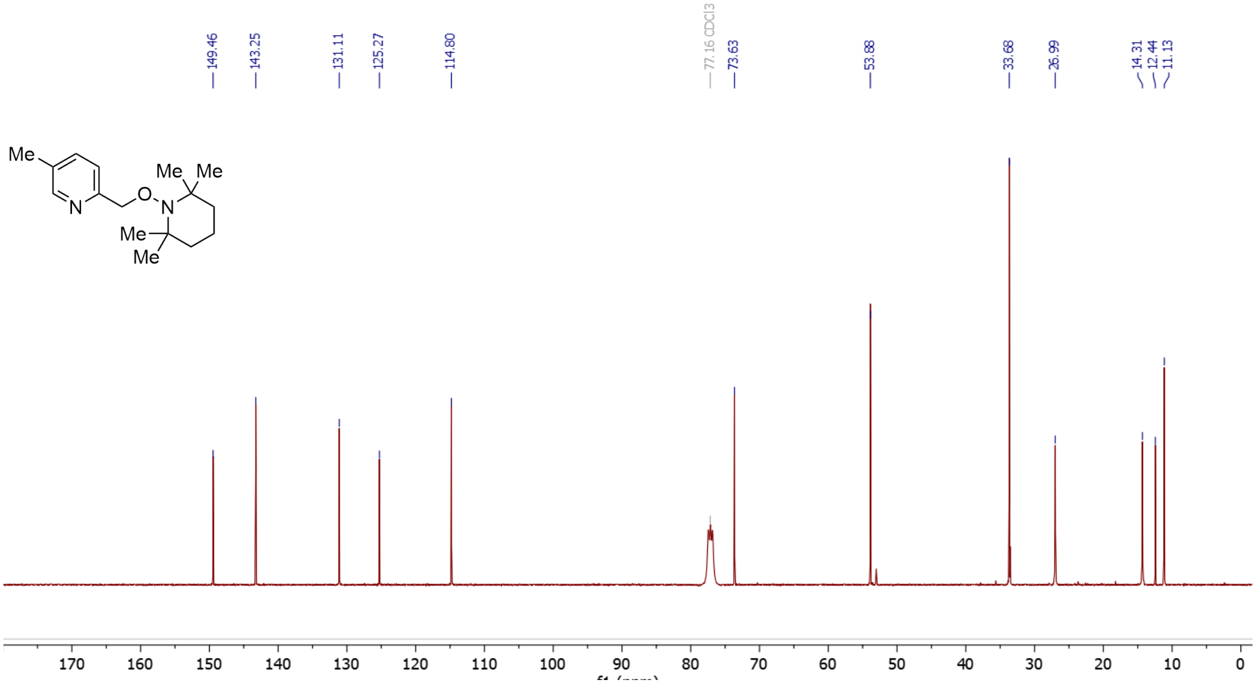

^{13}C **NMR** spectrum of **31** (101 MHz, $CDCl_3$)

3-methyl-4-(1-((2,2,6,6-tetramethylpiperidin-1-yl)oxy)propyl)pyridine (32)

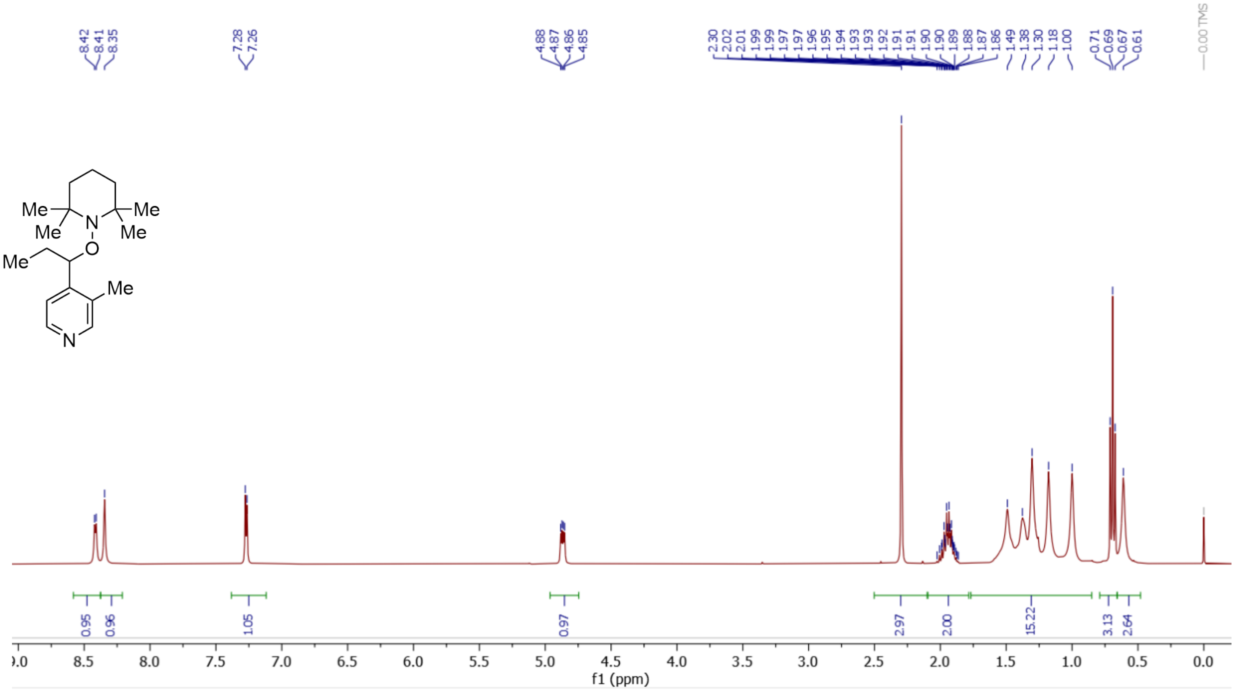

^{1}**H NMR** spectrum of **32** (400 MHz, $CDCl_3$)

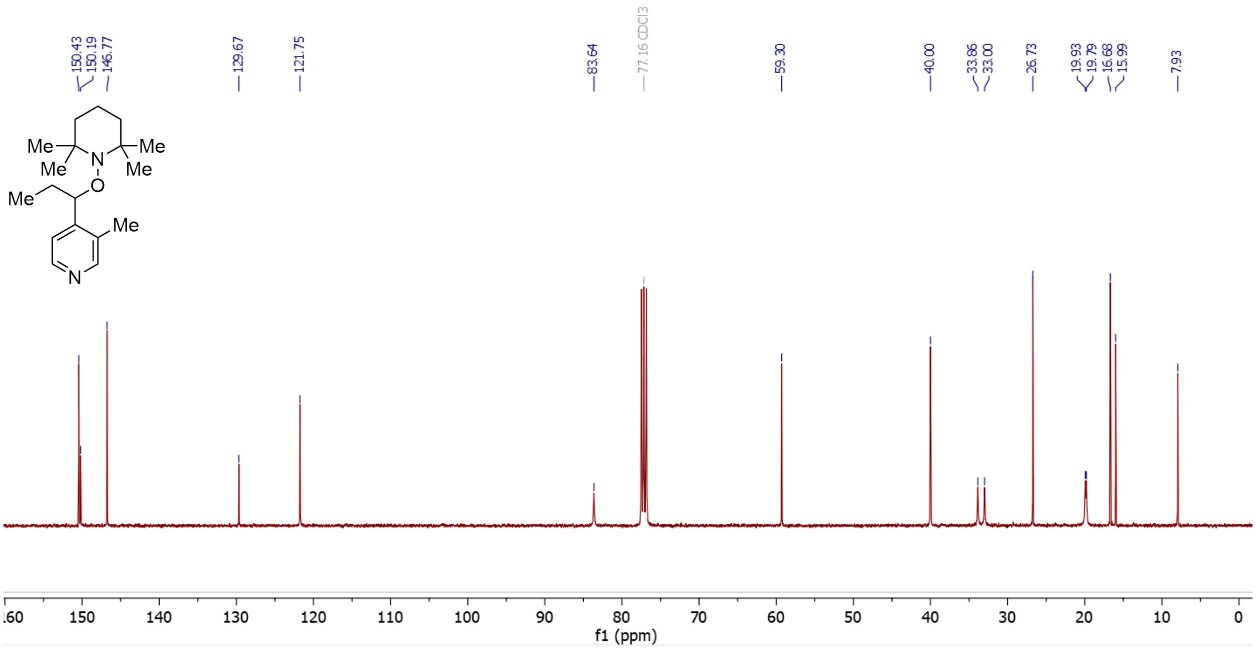

^{13}C **NMR** spectrum of **32** (101 MHz, $CDCl_3$)

1-((2,6-dibromo-3-methylbenzyl)oxy)-2,2,6,6-tetramethylpiperidine (33)

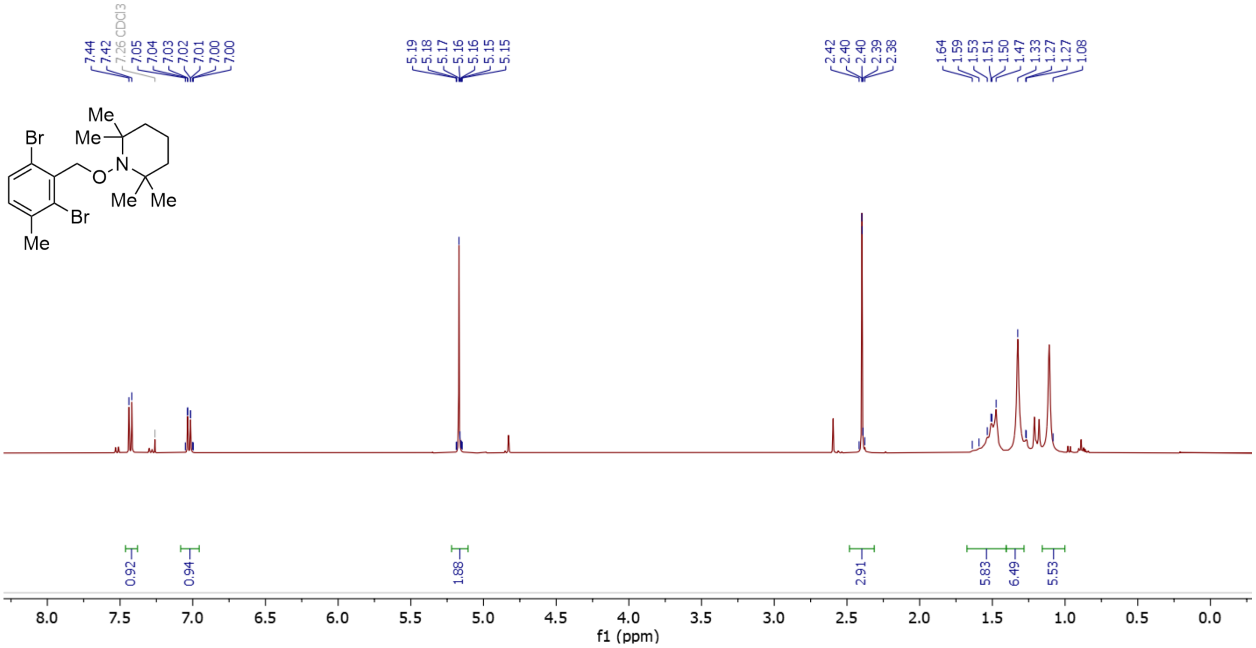

^{1}**H NMR** spectrum of **33** with minor amount of positional isomer (400 MHz, $CDCl_3$)

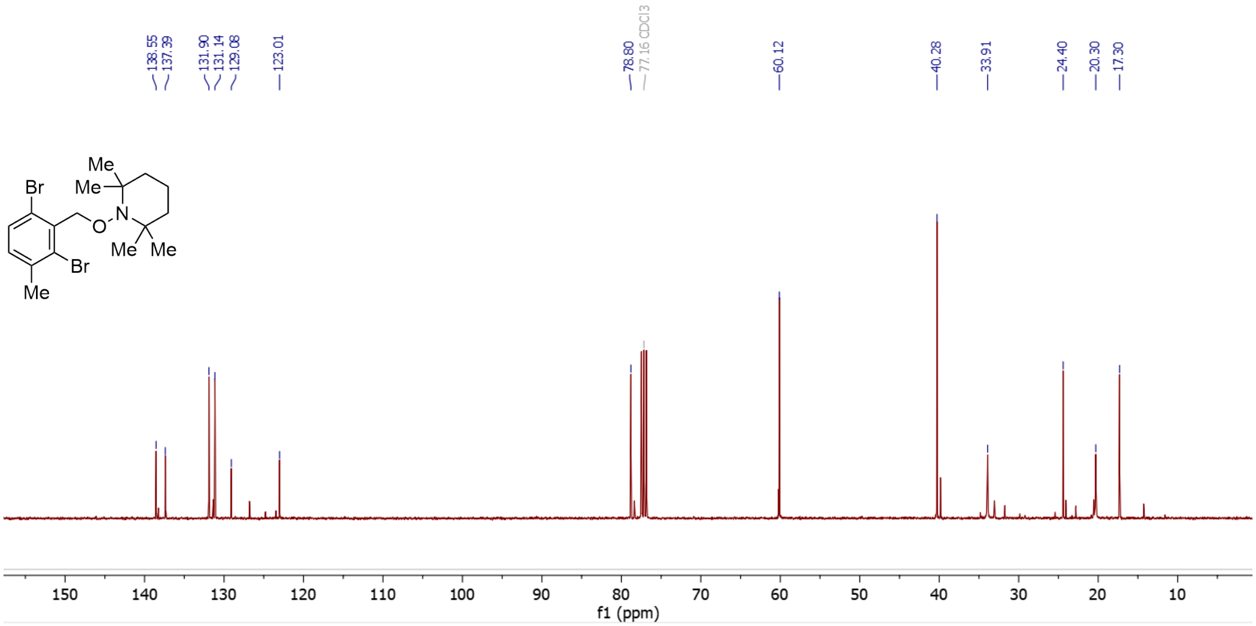

^{13}C NMR spectrum of **33** (101 MHz, $CDCl_3$)

2-(3-phenyl-1-((2,2,6,6-tetramethylpiperidin-1-yl)oxy)propyl)pyridine (34)

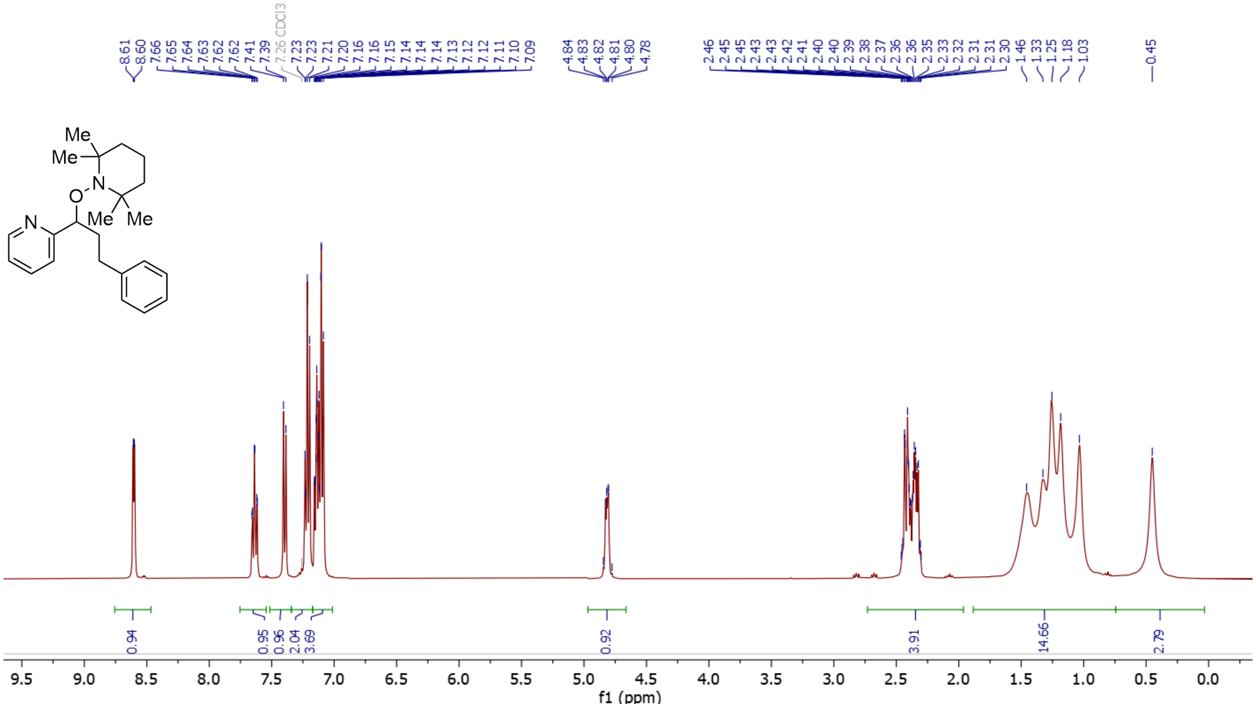

^{1}H NMR spectrum of **34** (400 MHz, $CDCl_3$)

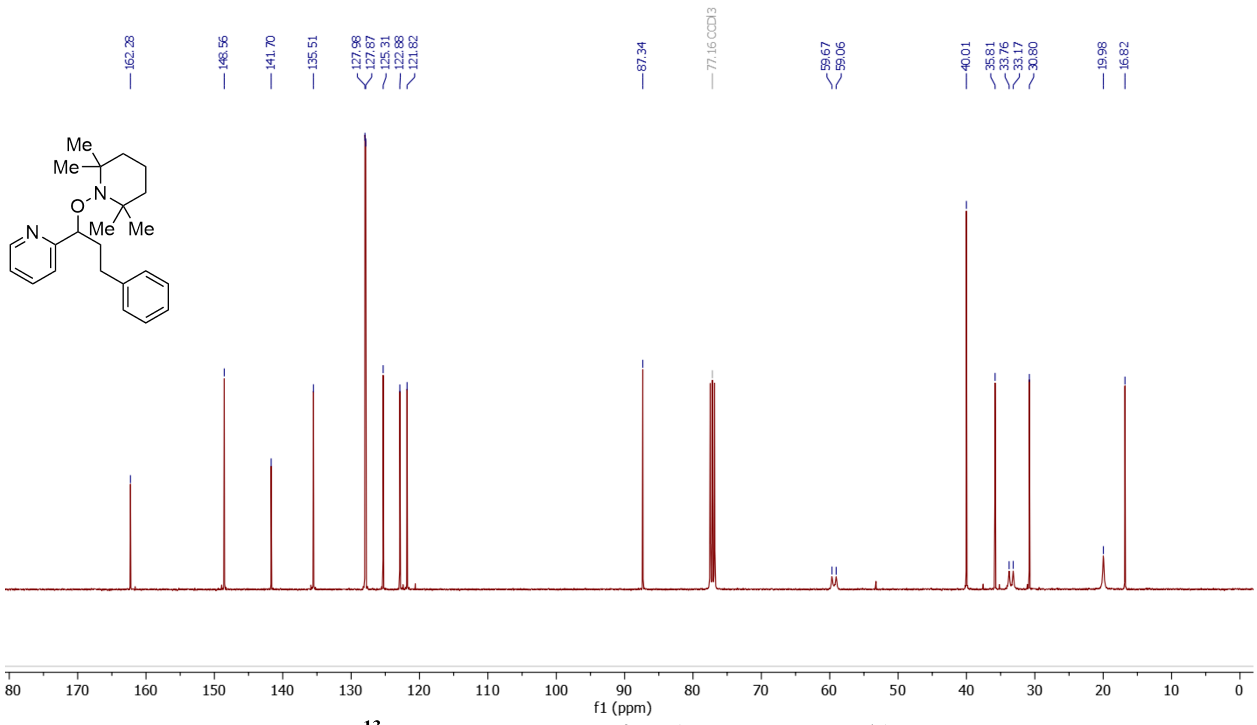

^{13}**C NMR** spectrum of **34** (101 MHz, $CDCl_3$)

1-((4-cyclohexylbenzyl)oxy)-2,2,6,6-tetramethylpiperidine (35)

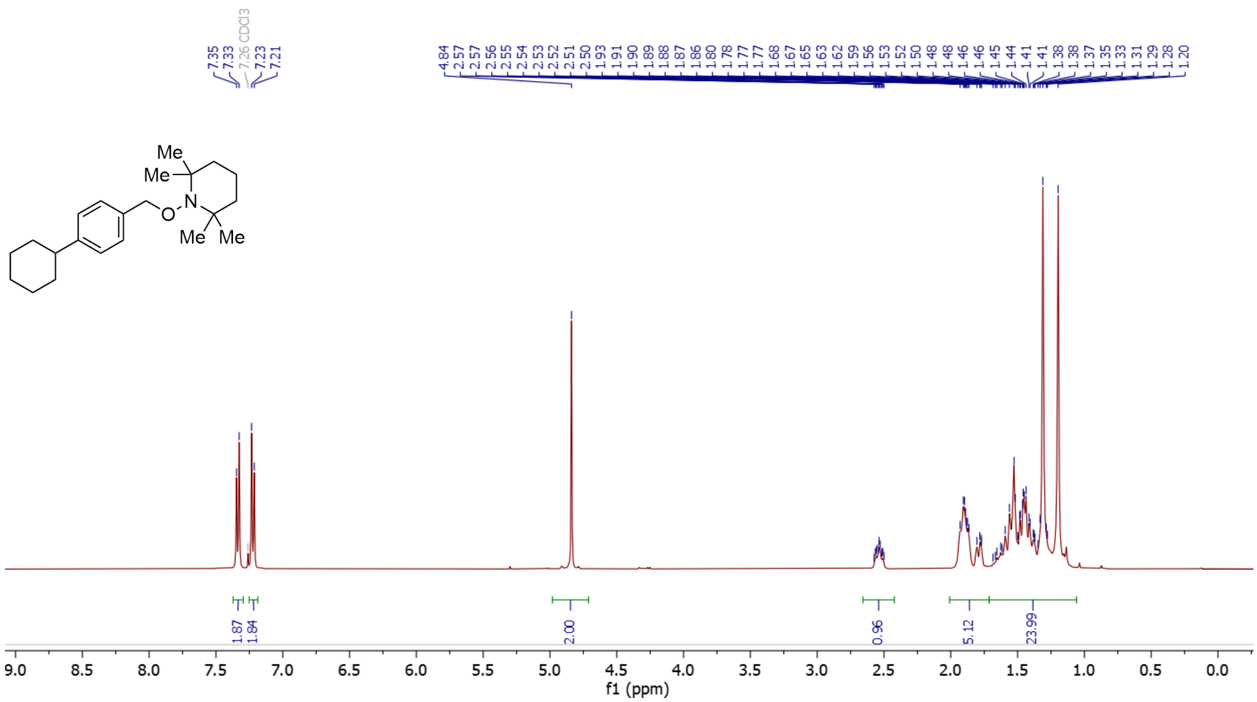

^{1}**H NMR** spectrum of **35** (400 MHz, $CDCl_3$)

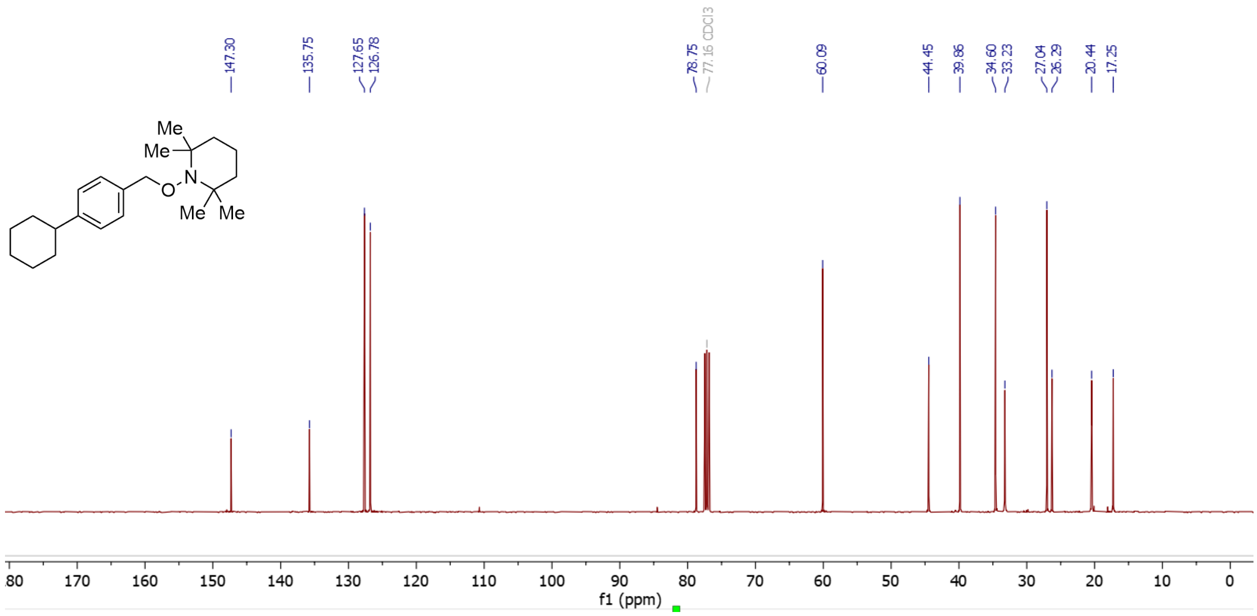

^{13}**C NMR** spectrum of **35** (101 MHz, $CDCl_3$)

2,2,6,6-tetramethyl-1-((3'-methyl-[1,1'-biphenyl]-4-yl)methoxy)piperidine (36)

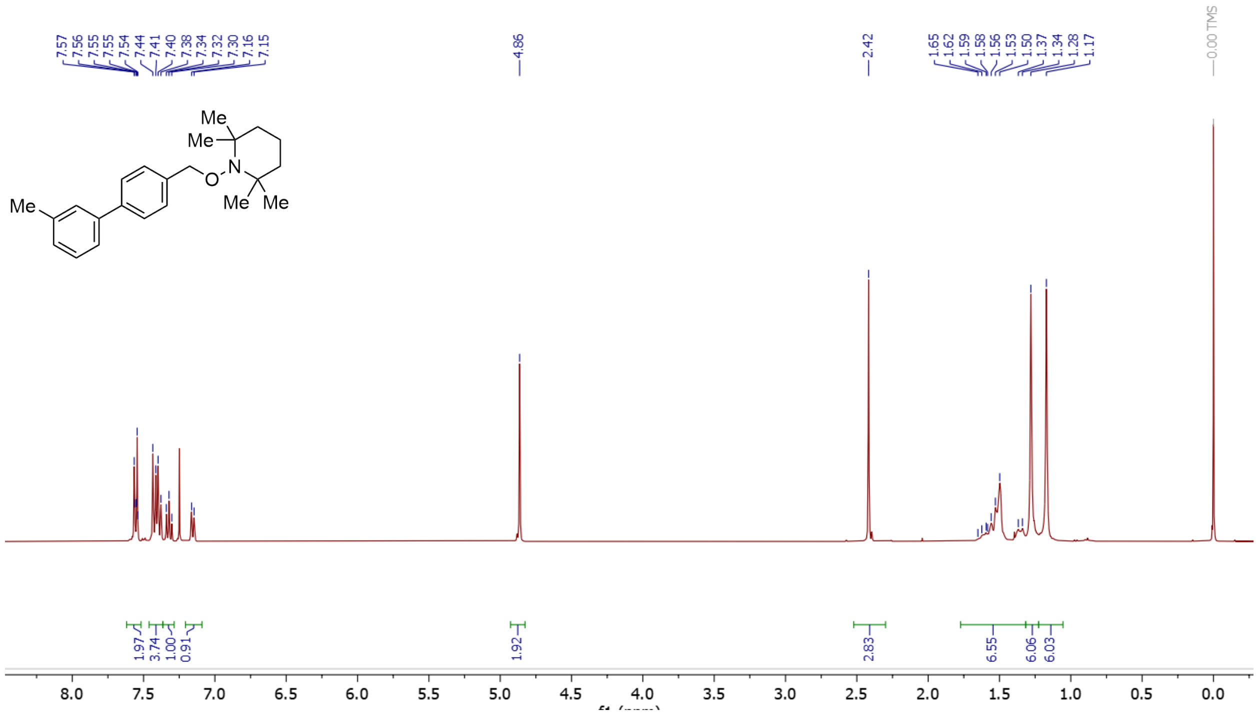

^{1}**H NMR** spectrum of **36** (400 MHz, $CDCl_3$)

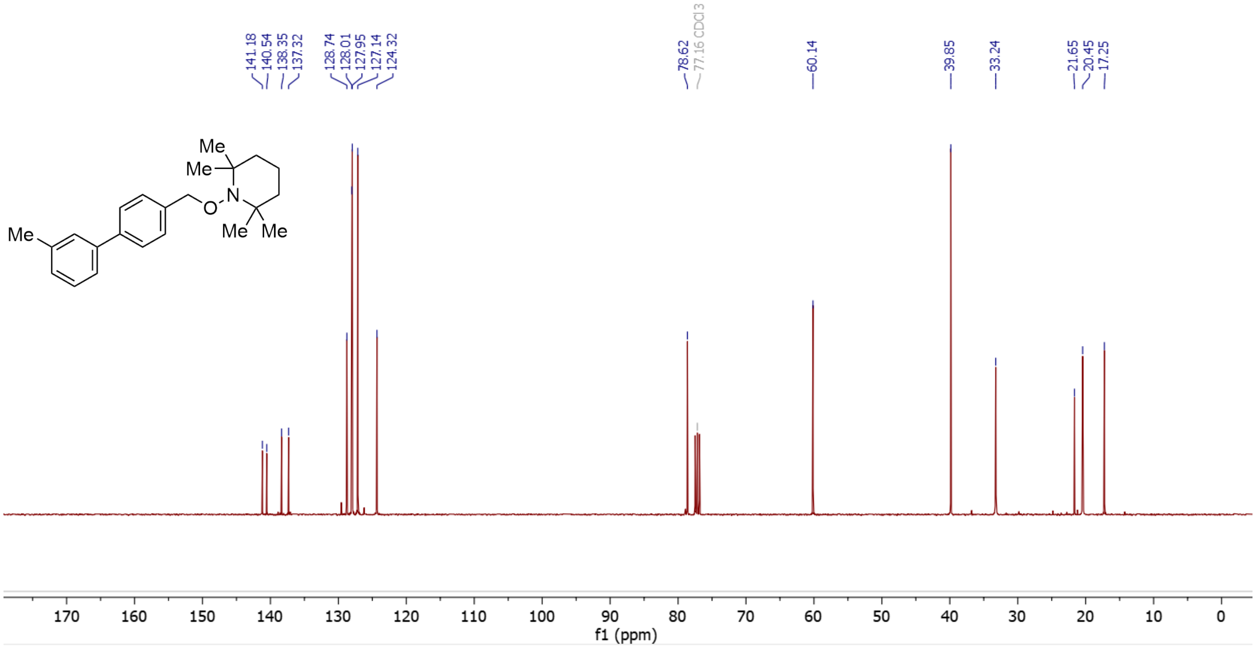

^{13}**C NMR** spectrum of **36** (101 MHz, $CDCl_3$)

1-((2-bromo-3-ethylbenzyl)oxy)-2,2,6,6-tetramethylpiperidine (37, 1° selective)

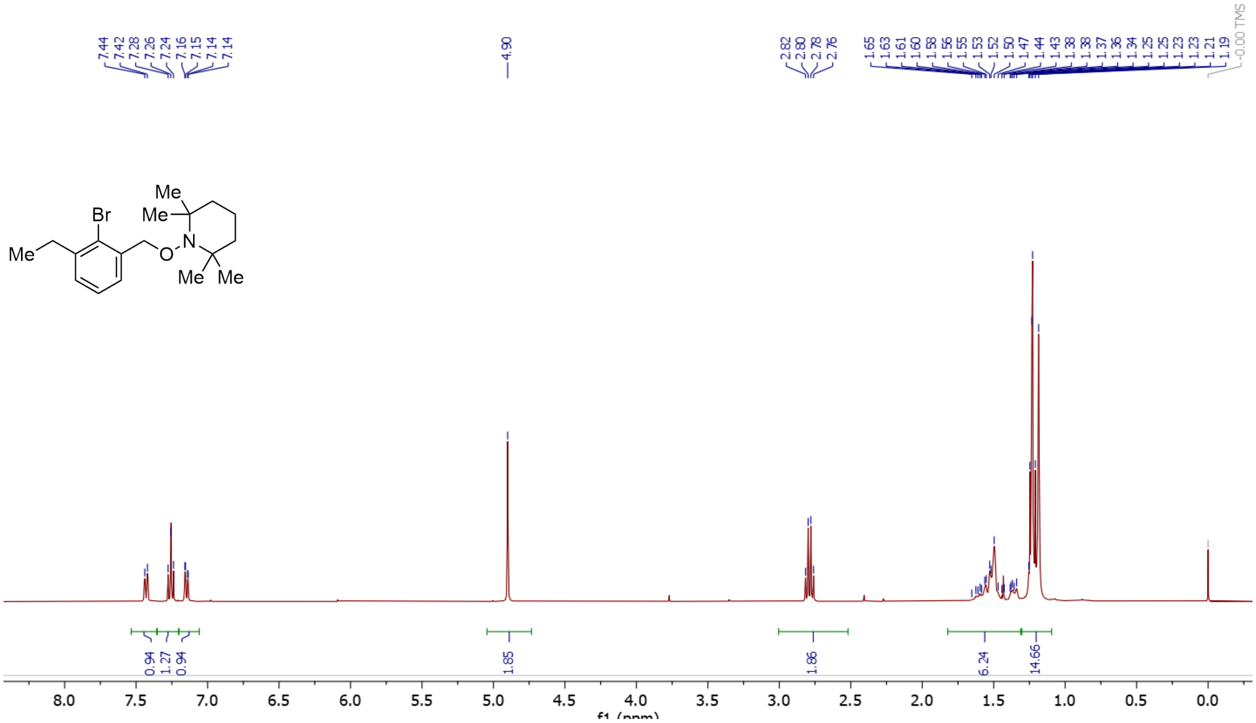

^{1}**H NMR** spectrum of **37, 1° selective** (400 MHz, $CDCl_3$)

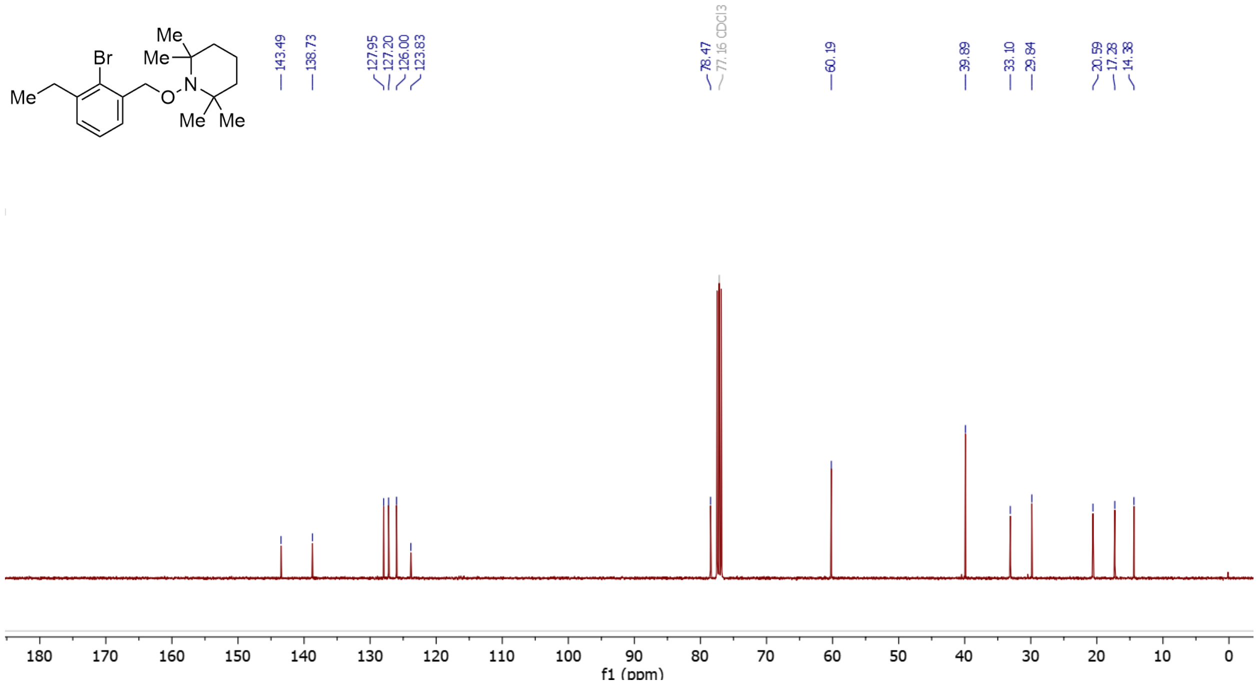

^{13}C **NMR** spectrum of **37, 1° selective** (101 MHz, $CDCl_3$)

1-(1-(2-bromo-3-methylphenyl)ethoxy)-2,2,6,6-tetramethylpiperidine (37, 2° selective)

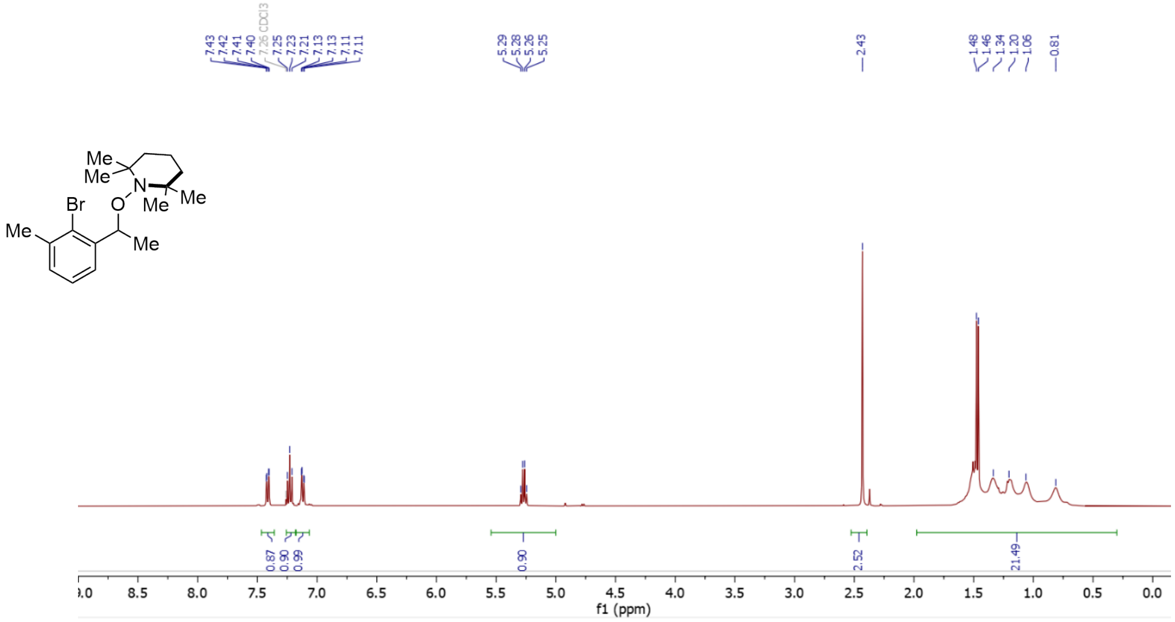

^{1}H **NMR** spectrum of **37, 2° selective** (400 MHz, $CDCl_3$)

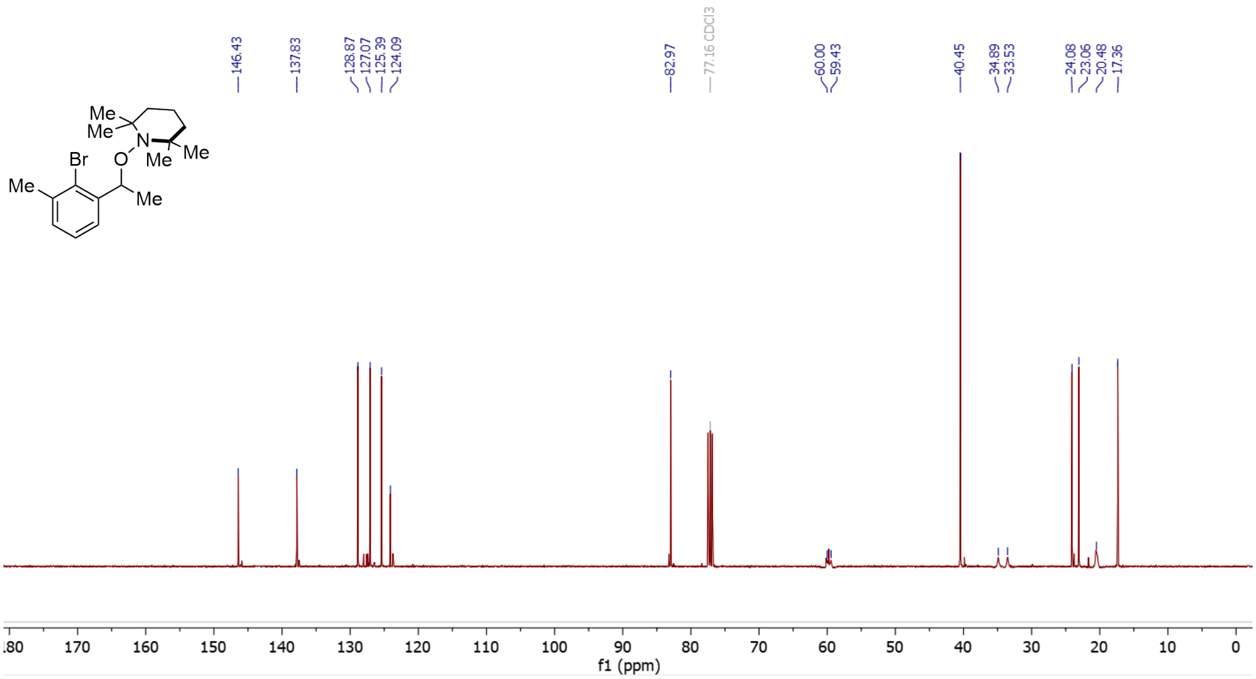

^{13}**C NMR** spectrum of **37, 2° selective** (101 MHz, $CDCl_3$)

2-ethyl-6-(((2,2,6,6-tetramethylpiperidin-1-yl)oxy)methyl)pyridine (38, 1° selective)

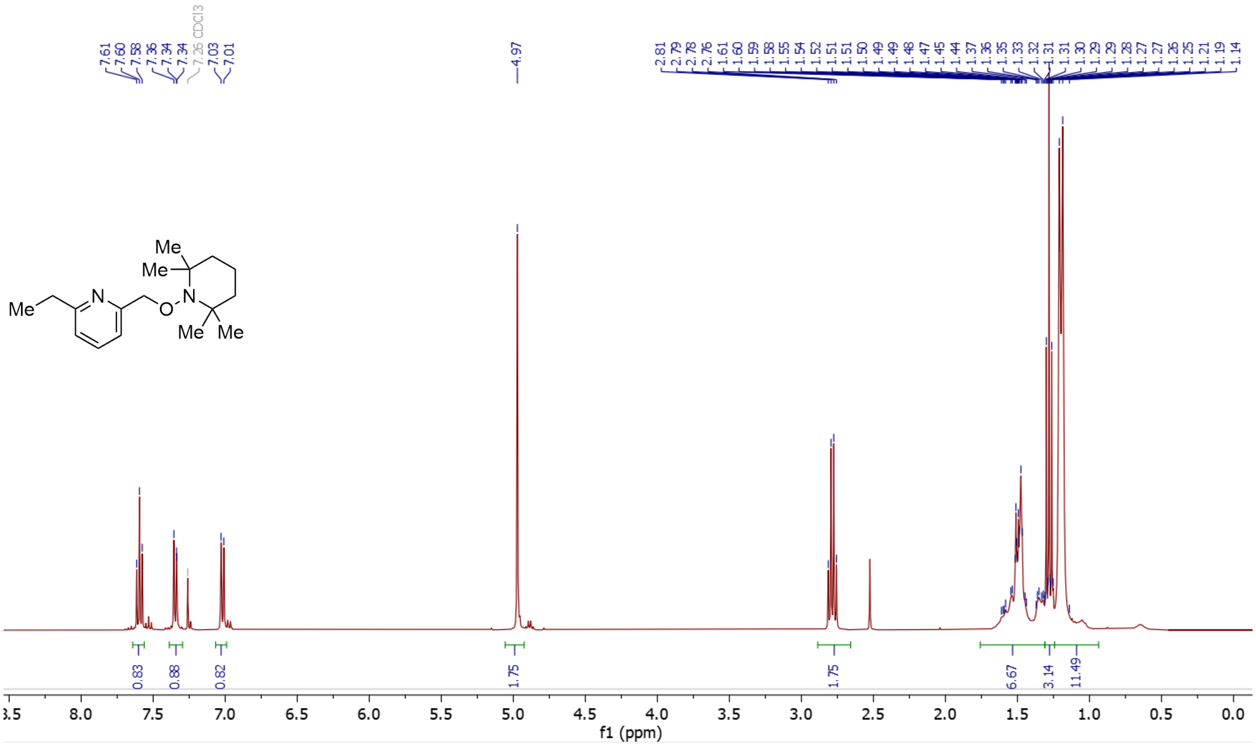

^{1}**H NMR** spectrum of **38, 1° selective** (400 MHz, $CDCl_3$)

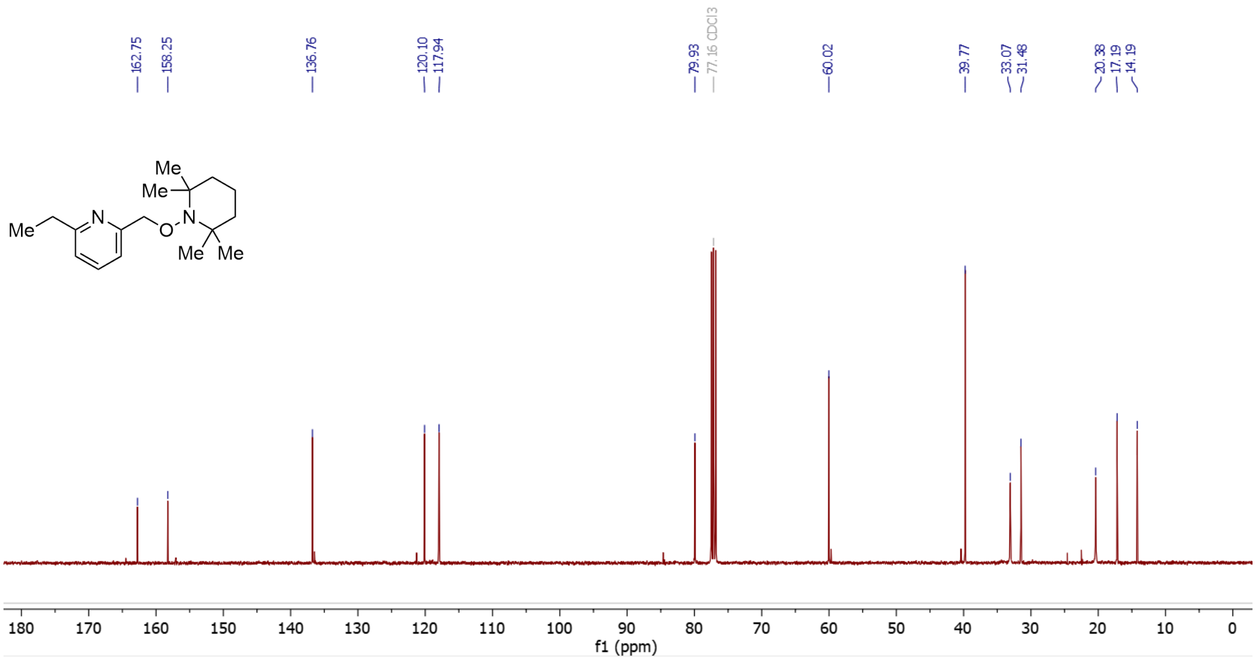

^{13}**C NMR** spectrum of **38, 1° selective** (101 MHz, $CDCl_3$)

2-methyl-6-(1-((2,2,6,6-tetramethylpiperidin-1-yl)oxy)ethyl)pyridine (38, 2° selective)

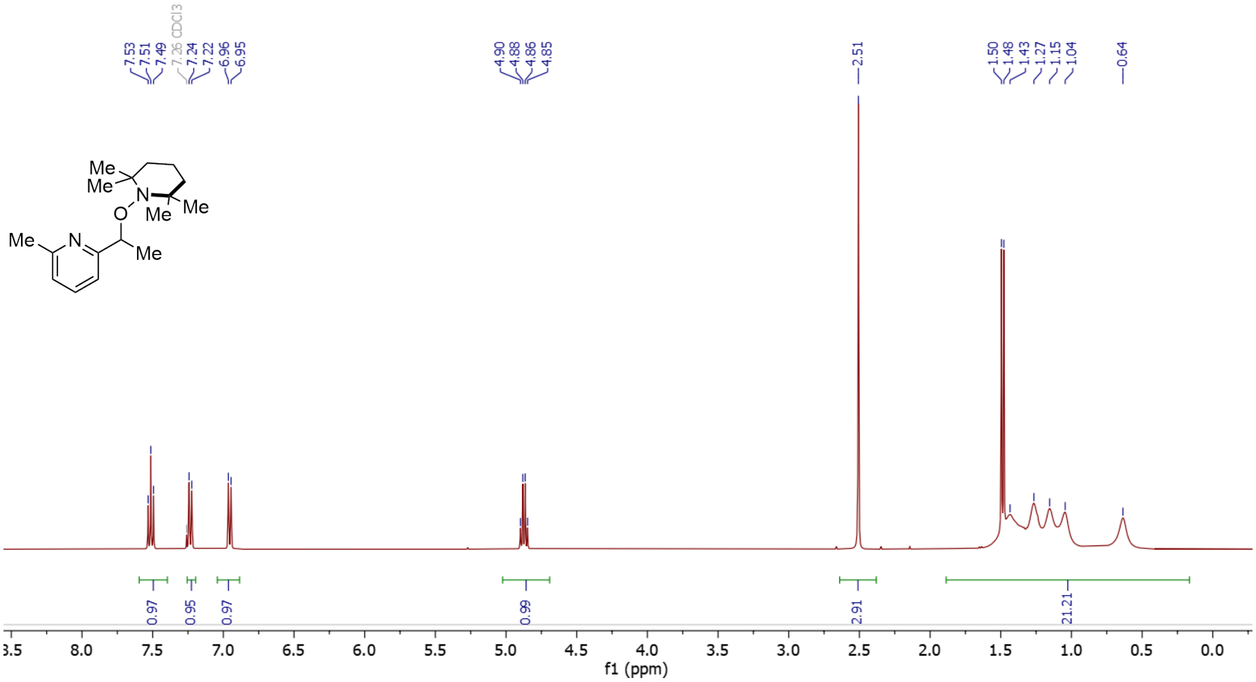

^{1}**H NMR** spectrum of **38, 2° selective** (400 MHz, $CDCl_3$)

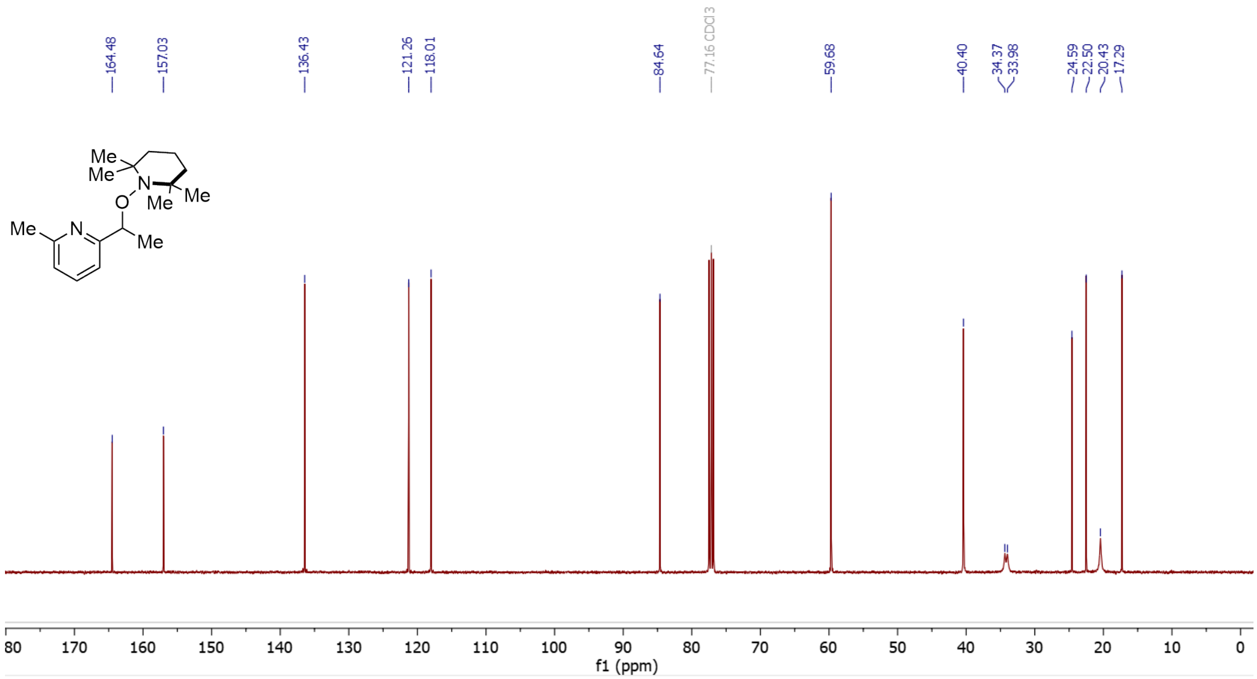

^{13}C **NMR** spectrum of **38, 2° selective** (101 MHz, $CDCl_3$)

2,2,6,6-tetramethyl-1-((4'-pentyl-[1,1'-biphenyl]-4-yl)methoxy)piperidine (39, 1° selective)

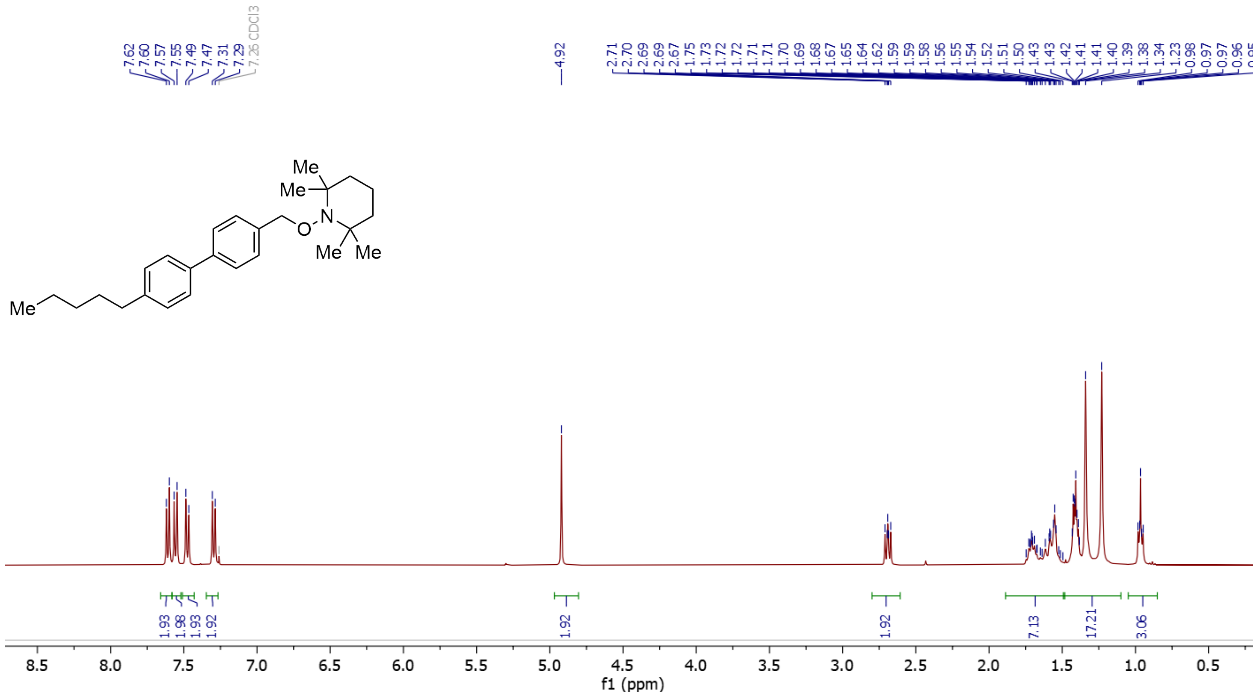

^{1}H **NMR** spectrum of **39, 1° selective** (400 MHz, $CDCl_3$)

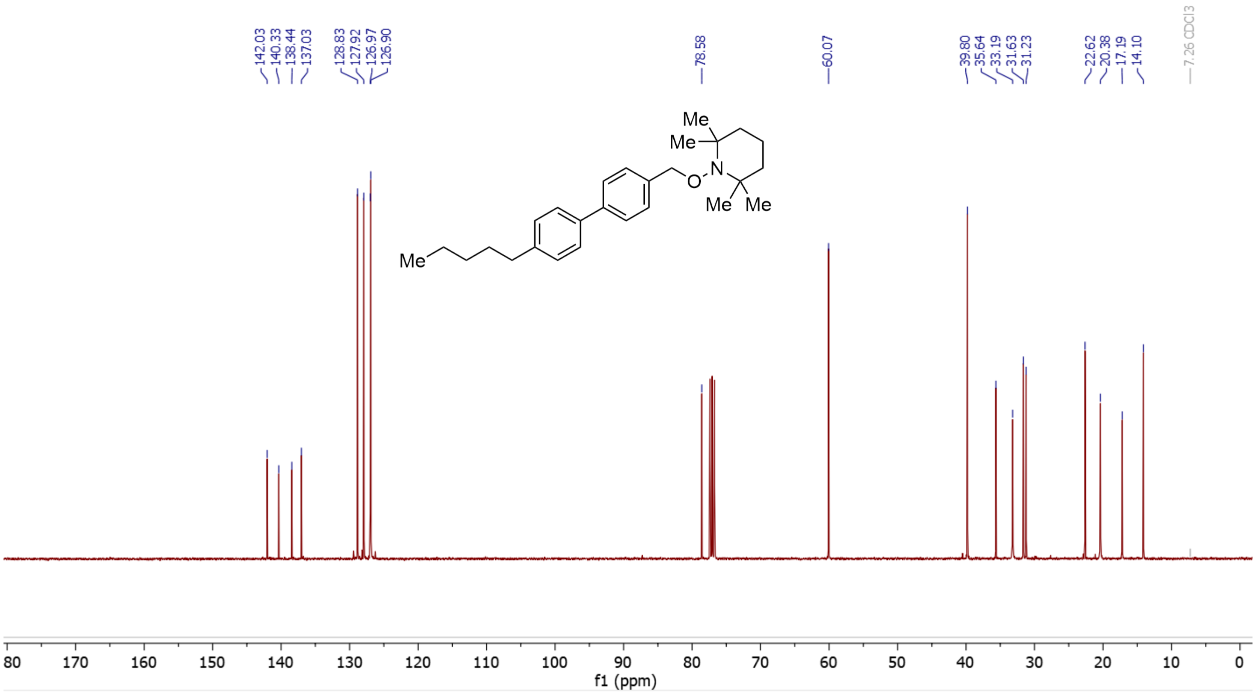

^{13}C **NMR** spectrum of **39, 1° selective** (101 MHz, $CDCl_3$)

2,2,6,6-tetramethyl-1-((1-(4'-methyl-[1,1'-biphenyl]-4-yl)pentyl)oxy)piperidine (39, 2° selective)

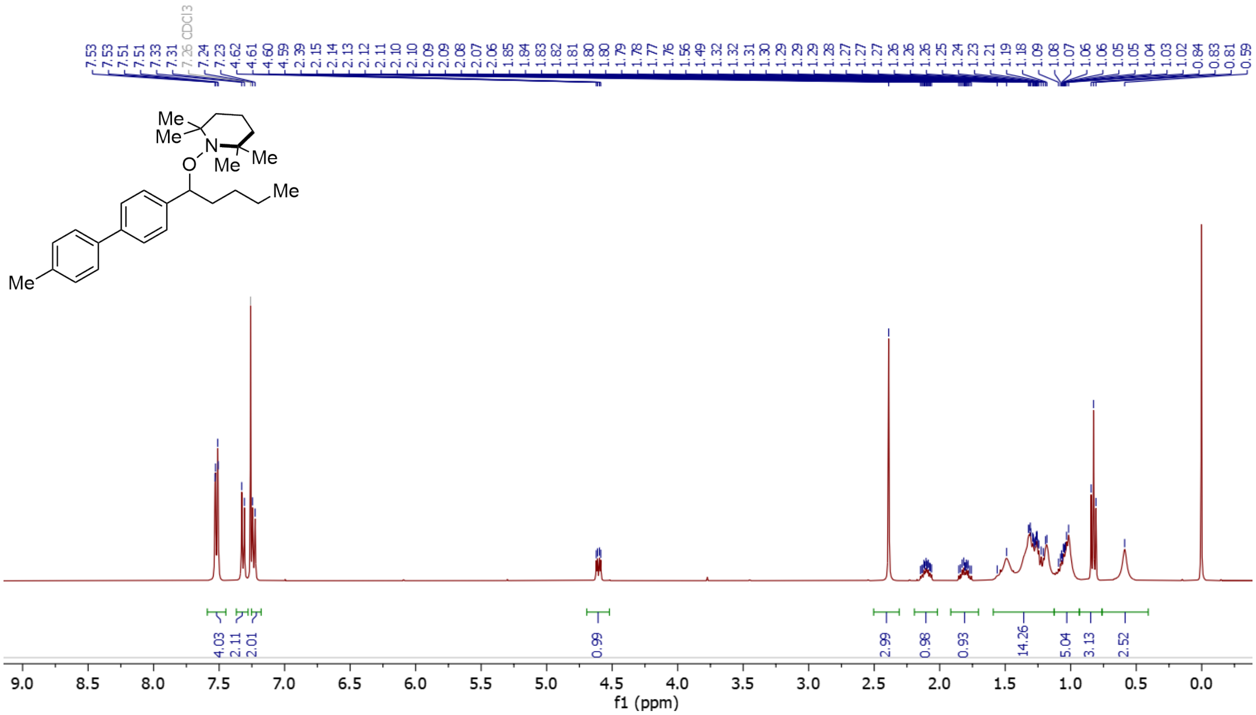

^{1}H **NMR** spectrum of **39, 2° selective** (400 MHz, $CDCl_3$)

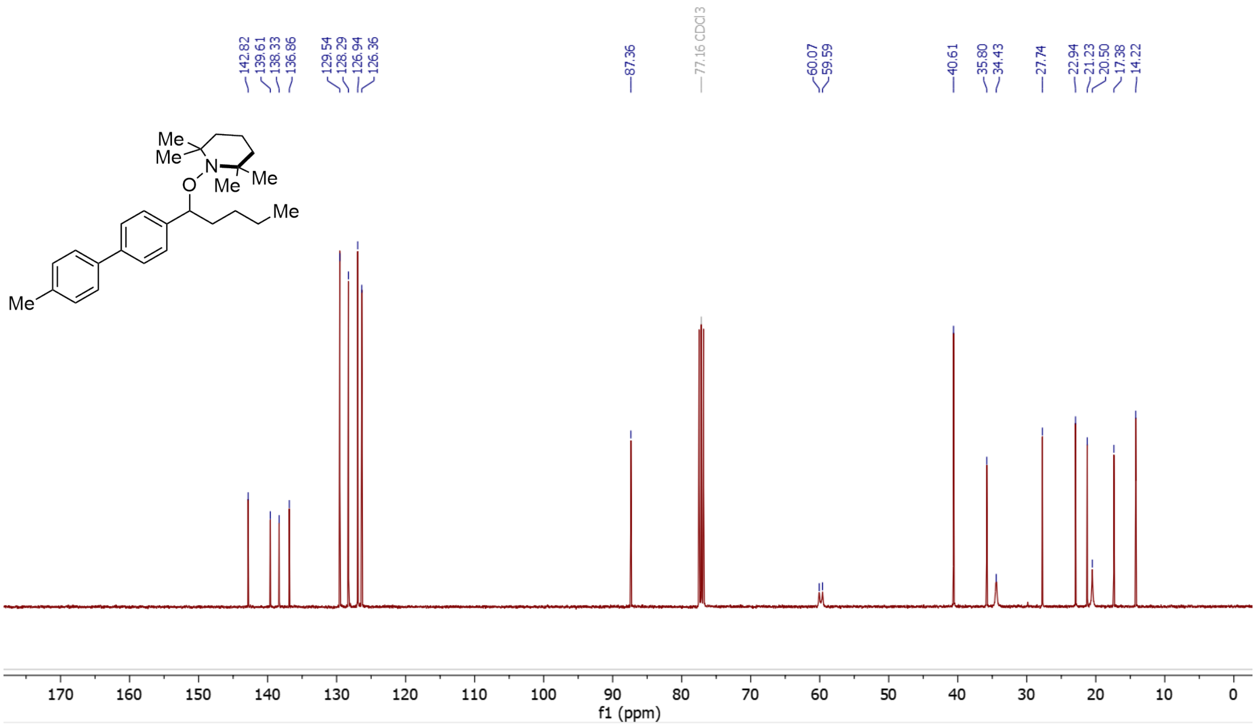

^{13}C NMR spectrum of **39, 2° selective** (101 MHz, $CDCl_3$)

1-((4-((4-ethylphenyl)ethynyl)benzyl)oxy)-2,2,6,6-tetramethylpiperidine (40, 1° selective)

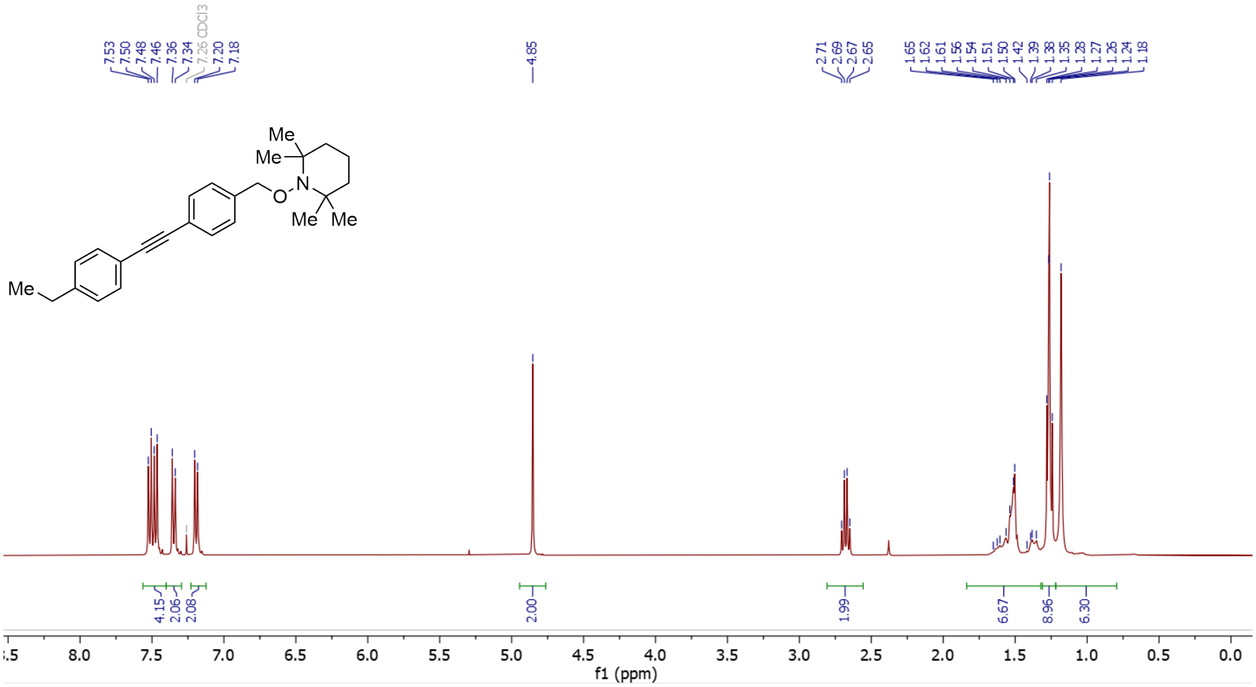

^{1}H NMR spectrum of **40, 1° selective** (400 MHz, $CDCl_3$)

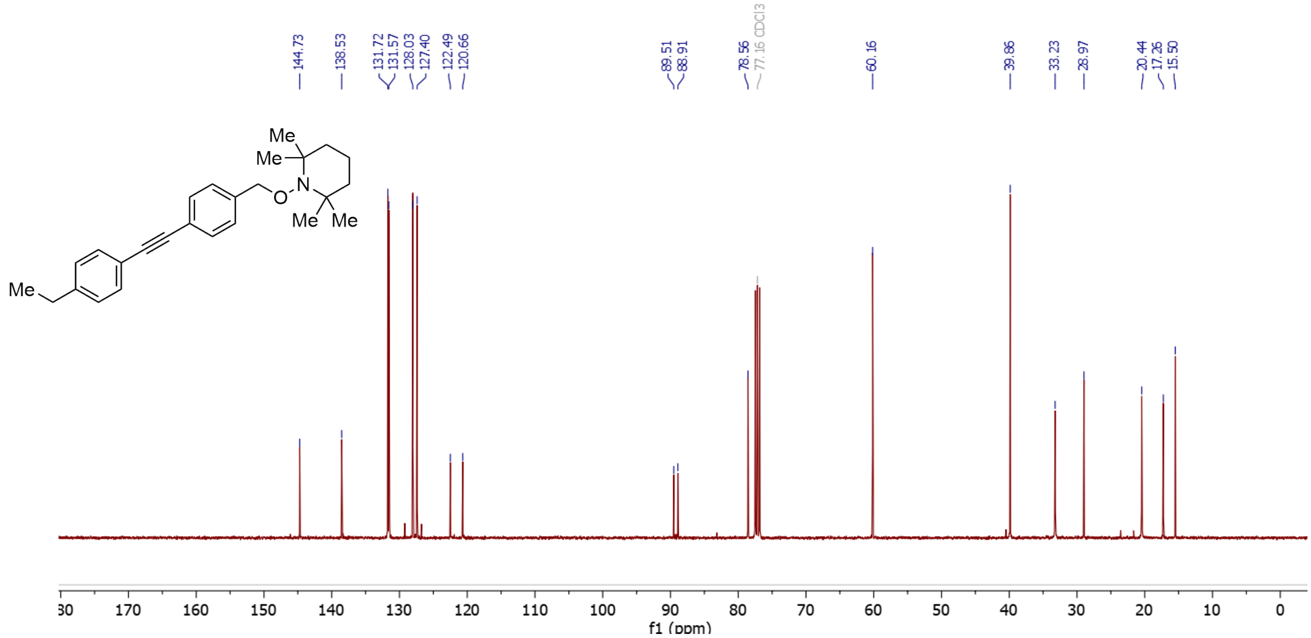

^{13}C NMR spectrum of **40, 1° selective** (101 MHz, $CDCl_3$)

2,2,6,6-tetramethyl-1-(1-(4-(p-tolylethynyl)phenyl)ethoxy)piperidine (40, 2° selective)

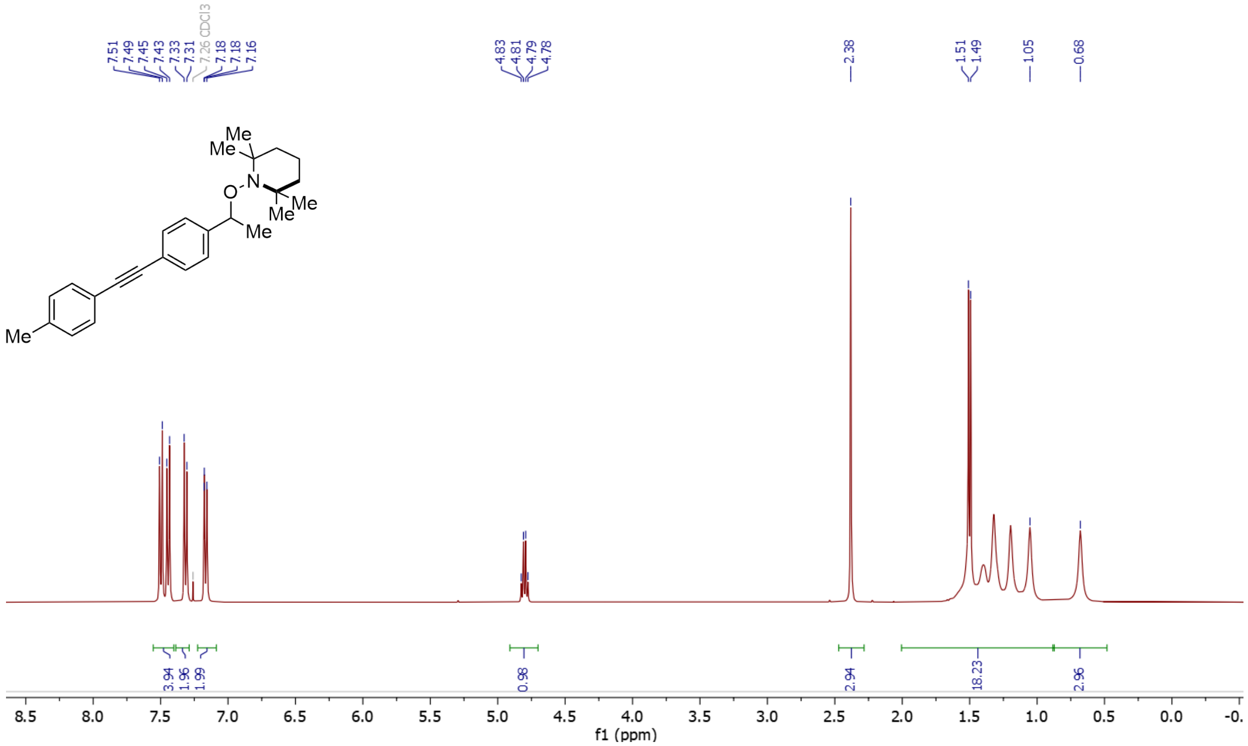

^{1}H NMR spectrum of **40, 2° selective** (400 MHz, $CDCl_3$)

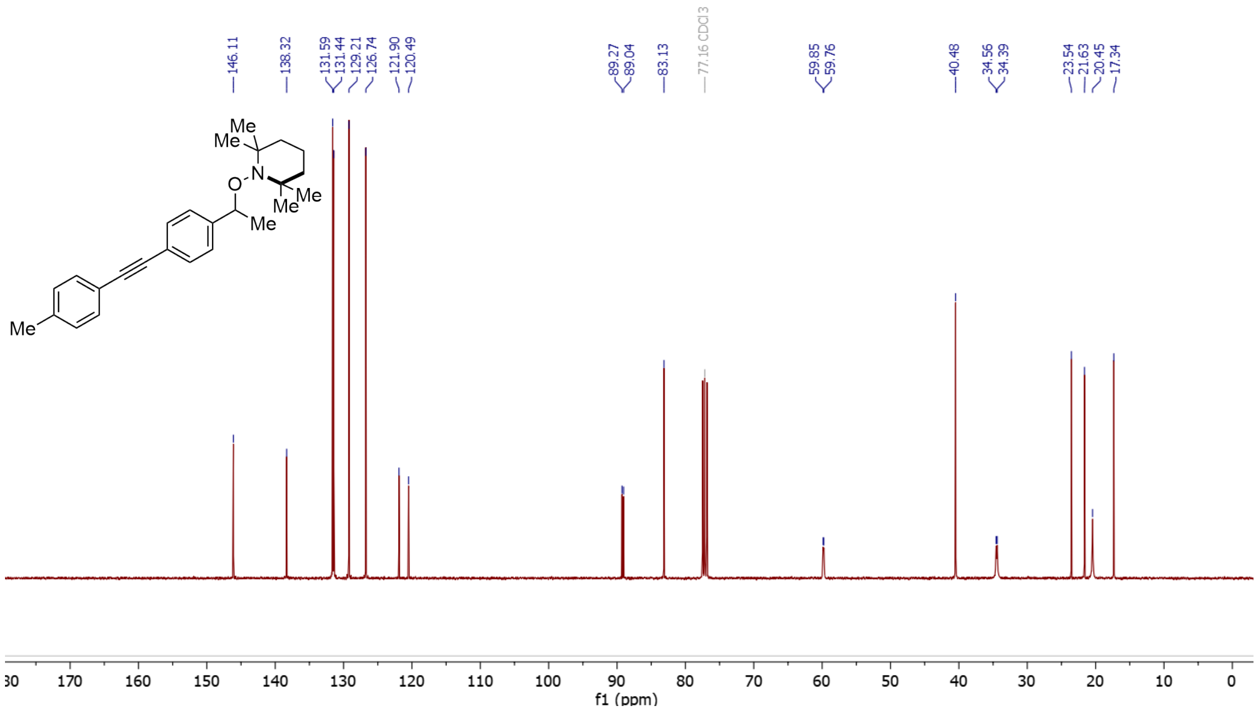

^{13}C **NMR** spectrum of **40, 2° selective** (101 MHz, $CDCl_3$)

2-propyl-6-(((2,2,6,6-tetramethylpiperidin-1-yl)oxy)methyl)pyridine (41, 1° selective)

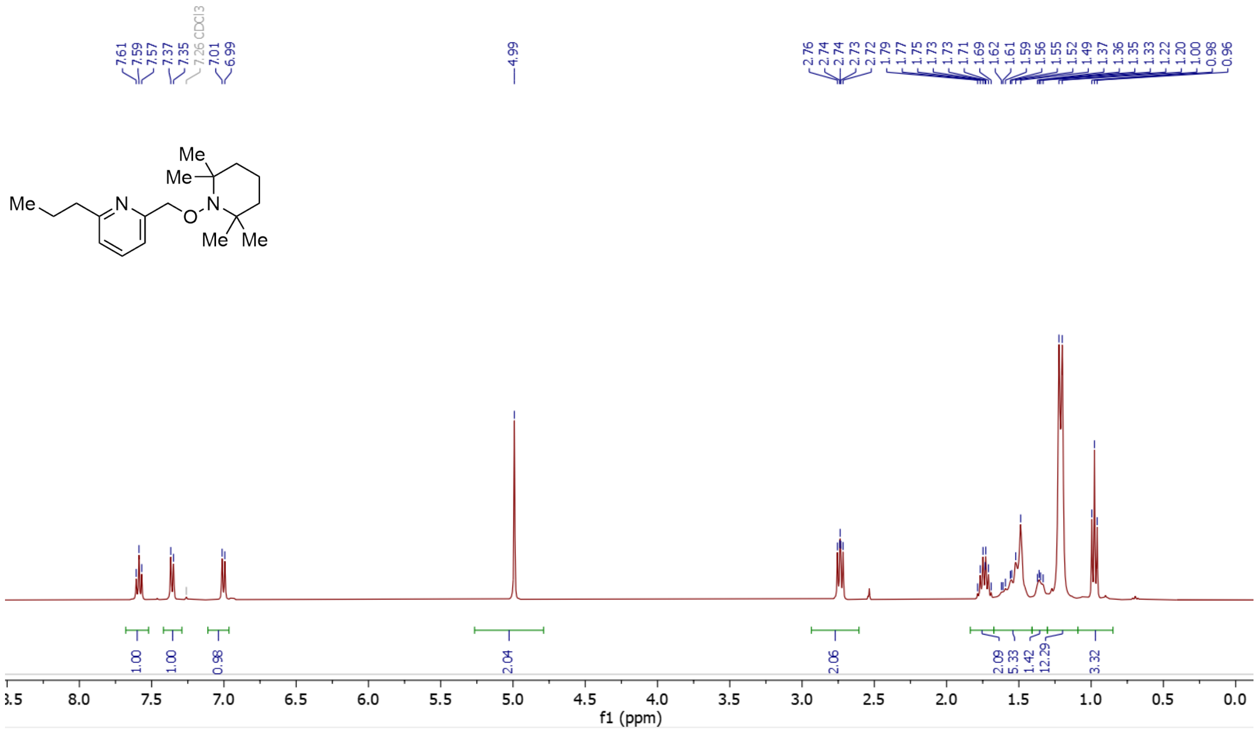

^{1}H **NMR** spectrum of **41, 1° selective** (400 MHz, $CDCl_3$)

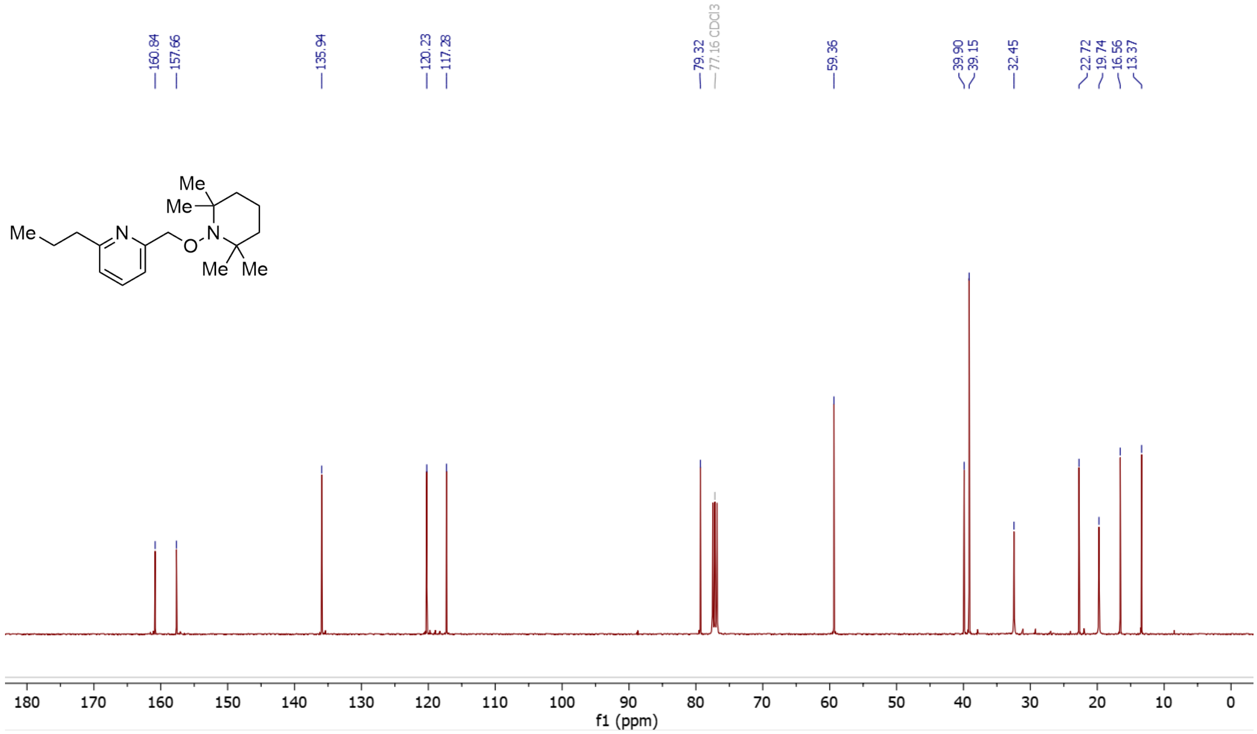

^{13}**C NMR** spectrum of **41, 1° selective** (101 MHz, $CDCl_3$)

2-methyl-6-(1-((2,2,6,6-tetramethylpiperidin-1-yl)oxy)propyl)pyridine (41, 2° selective)

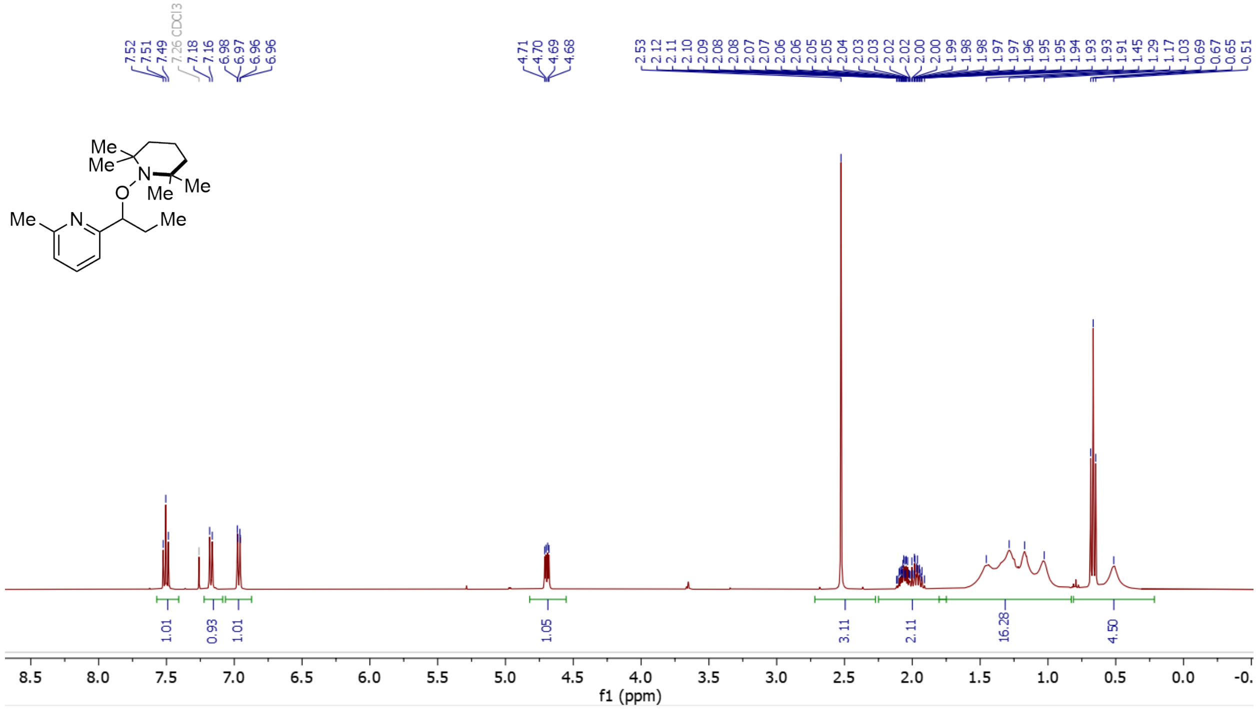

^{1}**H NMR** spectrum of **41, 2° selective** (400 MHz, $CDCl_3$)

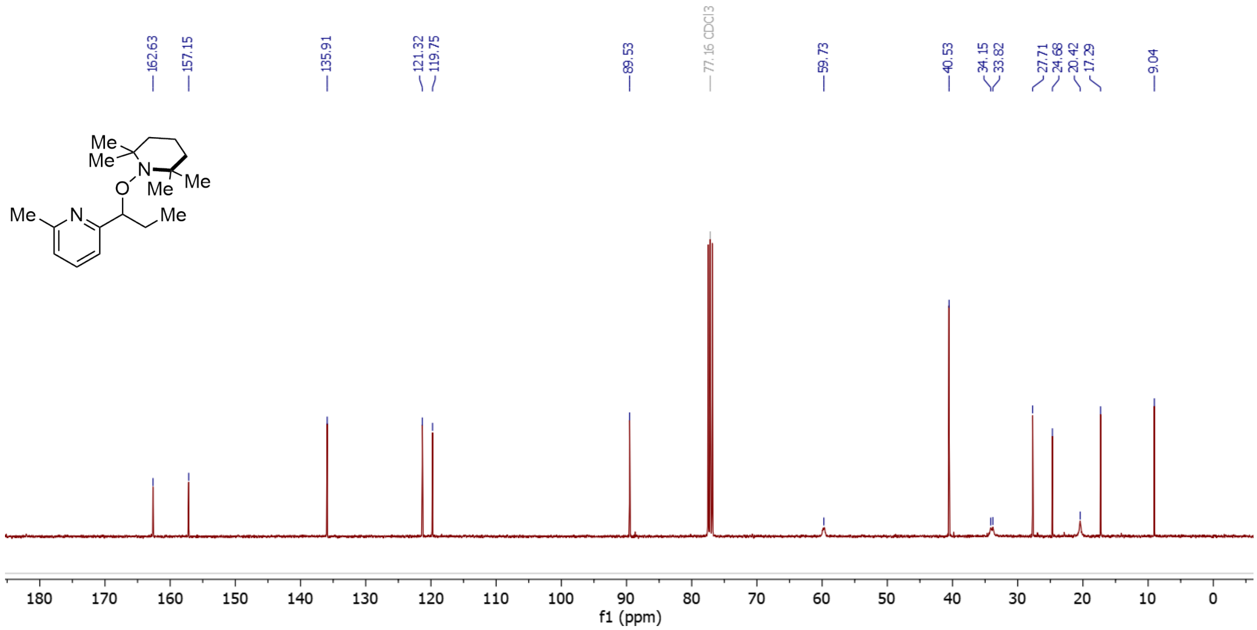

[13]**C NMR** spectrum of **41, 2° selective** (101 MHz, $CDCl_3$)
